# Generation Malawi: a family-based birth cohort investigating physical and mental health conditions in rural and urban Malawi – study protocol

**DOI:** 10.1136/bmjopen-2026-122132

**Published:** 2026-09-15

**Authors:** Wisdom P Nakanga, Robert C Stewart, Jullita Kenala Malava, Providence Nindi, Rebecca Nzawa-Soko, Innocent Nyanjagha, Angella Mainjeni, Thandile Gondwe, Eric Umar, Lucinda Manda-Taylor, Genesis Chorwe-Sungani, Melissa Gladstone, Marko Kerac, Angus MacBeth, Nadine Seward, Noel Patson, McNeil Ngongondo, Don Mathanga, Andrew M McIntosh, Amelia C Crampin, Generation Malawi Collaborator Group, Abena Amoah

**Affiliations:** 1 Malawi Epidemiology and Intervention Research Unit, Lilongwe, Malawi; 2 Institute of Neuroscience and Cardiovascular Research, The University of Edinburgh Division of Psychiatry, Edinburgh, Scotland, UK; 3 London School of Hygiene and Tropical Medicine, London, UK; 4 Kamuzu University of Health Sciences, Blantyre, Southern Region, Malawi; 5 Women and Children’s Health, University of Liverpool Institute of Translational Medicine, Liverpool, UK; 6 Department of Clinical and Health Psychology, School of Health in Social Science, University of Edinburgh, Edinburgh, UK; 7 Malaria Alert Centre, University of Malawi College of Medicine, Blantyre, Southern Region, Malawi; 8 Institute of Health and Wellbeing, University of Glasgow College of Medical Veterinary and Life Sciences, Glasgow, UK

**Keywords:** Africa South of the Sahara, EPIDEMIOLOGY, Family, Antenatal, Longitudinal studies

## Abstract

**Abstract:**

**Introduction:**

Malawi is at a unique time point when infectious disease and undernutrition persist while modern diets, and changes in urbanisation, medical treatments and climate change, non-communicable long-term physical and mental health conditions are emerging. Malawi faces high socioeconomic hardships with 75% of families living in poverty with limited access to basic resources. With these demographic changes, very limited research has been undertaken into the determinants of physical and mental health-related trajectories over time, and in particular the role of prenatal and early life exposures in predetermining long-term conditions in later life.

The study will establish a unique multigenerational birth cohort across contrasting rural and urban settings in Malawi to examine how pregnancy, early-life and intergenerational factors shape long-term mental and physical health

**Methods and analysis:**

Generation Malawi is based within the open, health and demographic surveillance sites in rural (southern Karonga District) and urban (high-density sectors of Area 25, Lilongwe) Malawi. The study seeks to enrol up to 5000 pregnant women aged 16 years and above, and their spouses, during early pregnancy. Pregnant women and their spouses are followed up until the delivery date at which time their baby is recruited into the study. Children and their parents are then followed up at 1 week, 6 weeks, 4, 12, 24 and 36 months after delivery. At each point, we conduct detailed interviewer-led questionnaires, physical measurements and collection of bio-samples for immediate analysis and long-term storage.

The data from this cohort will help us understand the causes of long-term ill health and, in the future, help develop effective interventions to prevent and promptly treat these conditions.

**Ethics and dissemination:**

This study protocol was approved by the National Health Science Research Committee in Malawi (number: 21/01/2653) and the Ethics Committee of the College of Medical, Veterinary and Life Sciences at the University of Glasgow, UK (protocol number: 200190183). The formative work for this study was granted ethical clearance by the College of Medicine Research Ethics Committee (protocol number P.11/19/2865). Results will be presented at relevant conferences and published in peer-reviewed journals.

**STRENGTHS AND LIMITATIONS OF THIS STUDY**

- Longitudinal follow-up with detailed questionnaire data, physical assessment and biological sample collection from mothers, fathers and children enables investigation of biological, environmental, psychosocial determinants of physical and mental health and how this translates to offspring.
- Inclusion of contrasting rural and urban populations enables assessment of the effects of urbanisation, socioeconomic adversity and lifestyle transitions on health outcomes.
- Loss to follow-up over time may introduce selection bias and reduce completeness of outcome data.
- Lower participation and retention among fathers may limit the complete assessment of paternal and intergenerational influences.

## Introduction

 Malawi, similar to other low-income African countries (LI-AC), is at a unique time point when infectious diseases and undernutrition persist while non-communicable long-term physical and mental health conditions are also emerging. This situation is further compounded by rapid urbanisation and climate change, giving rise to increasingly complex health landscapes. In Malawi, 21.4% of the population are undernourished; 33.2% of children under 5 years of age are stunted (chronically undernourished) and 2.8% are wasted (acutely malnourished).^1^ At the same time 27% of adults have a high body mass index and 9% are obese.^2^ This coexistence of undernutrition and rising obesity highlights a dual burden of disease. Limited understanding of the determinants and consequences of long-term physical health conditions, such as cardiovascular disease and type 2 diabetes, as well as mental health conditions (eg, depression and anxiety) limits present opportunities for effective and timely treatment interventions.

Early life exposures are increasingly recognised as important determinants of the development and outcomes of long-term conditions, alongside influences in mid and later life.^3^ Conceptualised in the ‘Developmental Origins of Health And Disease’ framework,^4^ exposure to undernutrition in utero and during infancy has been associated with increased risk of obesity, type 2 diabetes and cardiovascular disease.^5^ There is also evidence that exposures during the prenatal period and early infancy influence later risk of mental health conditions.^6^ For example, prenatal exposure to maternal depression may increase risk of mental health symptoms in childhood and adolescence, independent of shared genetic risk.^7^ Proposed biological pathways linking early life development to later life disease include fetal glucocorticoid overexposure,^8^ epigenetic modifications, inflammatory processes and alterations in the gut microbiome.^9^ Examining the roles of genetic (G) and environmental (E) effects and their interplay (G×E) is crucial for advancing mechanistic understanding.^10^

Despite the growing burden of long-term conditions in LI-AC there are major evidence gaps in understanding the impact of early environmental and genetic interplays on health and development and how this later affects life disease. Findings from high-income or other low- and middle-income countries (LMICs) cannot be assumed to apply to LI-AC where populations experience a high burden of overlapping nutritional, infectious, environmental and psychosocial exposures. To date, no study in LI-AC has integrated data on adult long-term physical and mental health across multiple interacting pathways (environmental, socioeconomic, psychological, nutritional, microbial and inflammatory determinants), from pre-conception through pregnancy and early childhood within a single birth cohort.

The Generation Malawi (GM) study establishes a unique multigenerational family/birth cohort in two very diverse settings in rural and urban Malawi to study the longitudinal course and pregnancy, early-life and intergenerational effects for long-term mental and physical health conditions. The study is nested in health and demographic surveillance systems to facilitate long-term tracking and is linked to multiple other population studies. The study will generate detailed datasets and a biorepository; with both data and biosamples made accessible to external researchers. This paper describes the unique setting, methods, data and biosamples collected, and outlines the processes of data/sample access.

## Methodology

### Study design

Generation Malawi is a multi-generational family/birth cohort nested within wider open population-based cohorts in rural and urban Malawi.

## Study setting

**Figure 1:**
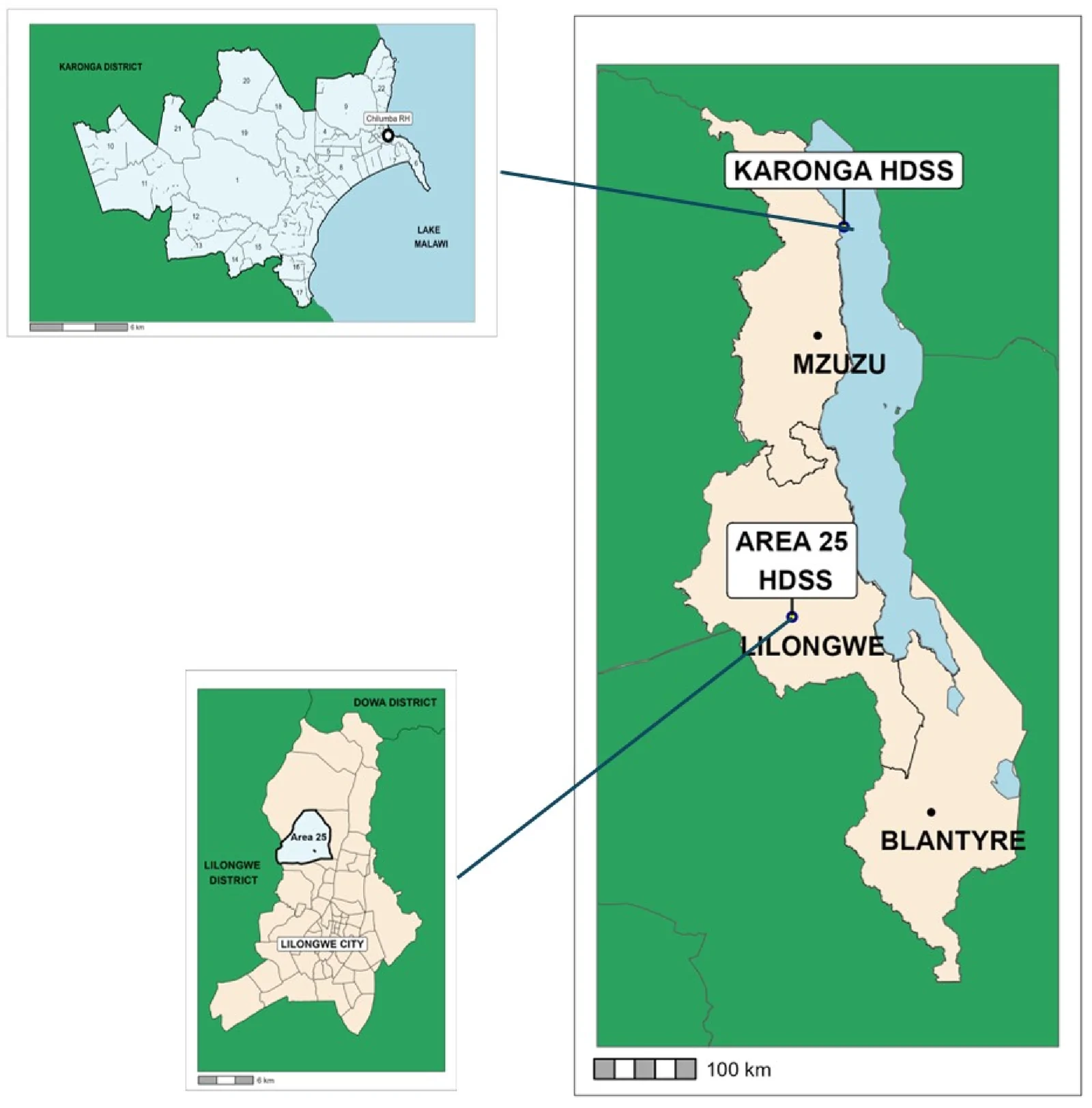
MEIRU HDSS located in rural, Chilumba, Karonga district and urban, Area 25, Lilongwe. HDSS, Health and Demographic Surveillance Site; MEIRU, Malawi Epidemiology and Intervention Research Unit.

The study is recruiting from a rural and an urban site in Malawi: the rural site (population 54,000) is an established Health and Demographic Surveillance Site (HDSS) in southern Karonga District (Northern Region, Malawi) with an area of 135 km^2^; the urban site (current population under surveillance 39 000) is a newly established HDSS in Area 25 of Lilongwe City (Central Region),^11^ with an area of less than 3 km^2^ (figure 1). These two geographically and socioeconomically distinct locations capture highly contrasting rural and urban health and exposures. The rural population in southern Karonga District predominantly consists of subsistence farmers, fisher people and traders. The densely populated and rapidly growing urban population in Lilongwe is socioeconomically mixed with seasonal casual workers, civil servants and professionals and those engaged in small and large businesses. Ethnicity in both sites is black African, almost exclusively Bantu; with the rural area predominantly Tumbuka and the urban population mixed, with Chewa the dominant tribal affiliation.

Generation Malawi, the HDSS and other linked studies in both the rural and urban areas, are implemented by the Malawi Epidemiology and Intervention Research Unit (MEIRU), an independent unit formed as a partnership between the Malawi Ministry of Health, the Kamuzu University of Health Sciences, the London School of Hygiene and Tropical Medicine and the University of Glasgow. The University of Edinburgh leads the UK academic institutional engagement in Generation Malawi. The HDSS and associated population surveys of physical and mental long-term health conditions together form a project running parallel to Generation Malawi, known as Healthy Lives Malawi.

### Sample population

All pregnant women (age 16 years or over) attending their initial antenatal clinic visit ‘booking’ at Chilumba Rural Hospital (CRH, rural site) or Area 25 Health Centre (HC, urban site) and who live within the geographical boundaries of the HDSS sites, are invited to participate, along with their spouses. Infants born to participating women are recruited to the study at birth.

### Main objective

To establish a multigenerational family/birth cohort (nested within the Healthy Lives Malawi open, population-based cohorts) in rural (southern Karonga District) and urban (high-density sectors of Lilongwe Area 25) sites through which to study the longitudinal course, and pregnancy, early-life and intergenerational effects, for long-term mental and physical health conditions in Malawi.

### Specific objectives

1. To measure the incidence, prevalence and prognosis of common mental disorders (depression, anxiety, post-traumatic stress disorder (PTSD), substance misuse) in women presenting to antenatal clinics, and their spouses, over the pregnancy/postnatal period in urban and rural populations.
2. To identify associations of common mental disorders (depression, anxiety, PTSD, substance misuse) in women presenting to antenatal clinics, and their spouses, over the pregnancy/postnatal period in urban and rural populations, with socioeconomic status (SES), stressful life events (including intimate partner violence (IPV)), nutritional status and physical health.
3. To describe the longitudinal patterns of growth, nutrition and development from birth in infants born to these women in rural and urban populations (including the prevalence of stunting, underweight and severe developmental delay/disability/congenital abnormality).
4. To determine the impact of parental mental health on pregnancy and birth outcomes and early child growth and development in urban and rural populations.
5. To determine the effects of covariates (as above) on any association between parental mental health and pregnancy and birth outcomes and early child growth and development in urban and rural populations.
6. To collect a range of biosamples (including blood, mucosal swabs, faeces, urine, hair and breastmilk) from parents and offspring for storage in a biorepository to enable study of the role of genomics, epigenetics, microbiome, stress hormones, inflammation, metabolomics in trajectories and pregnancy, early-life and intergenerational effects, of mental and physical health conditions.
7. To provide a platform for Malawian researchers from MEIRU and the wider research community to extend follow-up of the families and nest substudies further exploring the longitudinal course, and pregnancy, early-life and intergenerational effects, for long-term mental and physical health conditions.

### Sample size determination

We plan to recruit 5000 pregnant women attending their first antenatal clinic (ANC) visit along with their spouses. Accounting for an expected 15% loss before delivery (including 4% multiple births), the resulting birth cohort will be around 4500 infants. The goal is to develop a resource usable for examining various conditions and subgroups; thus, a larger sample enhances future applications. This size is sufficient to accurately estimate disease burden and effects across the entire population and key subgroups such as sex and rural/urban areas. The study has over 90% power to detect an association between depression (prevalence 10%) and a risk factor with an OR of 1.2 in 5000 mothers.

### Study period

Formative activities for the study (see below) commenced in August 2019. Enrolment in the rural site study began in February 2022 and in the urban site in November 2022. Rural recruitment paused in December 2023 but restarted in 2025. The anticipated end of participant enrolment is December 2027. Between 2020 and 2024, progress of the study was severely disrupted by the COVID-19 pandemic, economic challenges facing Malawi (eg, inflation, disrupted fuel availability, foreign exchange restrictions) and other health (eg, cholera outbreaks) and environmental (eg, cyclones) emergencies, with some of these constituting ongoing challenges in Malawi.

### Formative work

#### Patient and public involvement

Before study implementation, a national launch of the programme was conducted to ensure engagement at all levels of the health service, the research community, district authorities, town councils and traditional authorities. Residents were engaged at both an individual and community level in all aspects of the programme. These interactions have been published elsewhere.^12 13^

#### Development of measures

We translated, adapted and piloted a core battery of self-report, interview and observational measures for adult mental and physical health, psychosocial risk factors and child health, growth and development in the two predominant languages spoken in the study sites (Chichewa in Lilongwe and Chitumbuka in Karonga). This process involved translation, expert consensus meetings, focus group discussions and piloting to establish the measures’ acceptability, reliability and validity. We conducted criterion validation of the mental health measures (9-item Patient Health Questionnaire (PHQ-9), 7-item Generalised Anxiety Disorder Scale (GAD-7), Primary Care PTSD Screen for Diagnostic and Statistical Manual of Mental Disorders (DSM)-5 (PC-PTSD-5)) against gold standard diagnostic interviews (Manuscript under review).

#### Establishing clinics and referral pathways

MEIRU has been running non-communicable disease (NCD) clinics for managing diabetes and hypertension, embedded within health facilities at the two study sites (Area 25 HC in Lilongwe and CRH in Karonga) since 2013. Referral pathways to these NCD clinics were integrated into the Generation Malawi procedures. We also set up new mental health clinics at the two sites and established referral pathways for participants identified as having possible mental health or substance use conditions, suicide risk or risk of harm through IPV. The mental health clinics provide assessment, diagnosis, signposting and treatment including ‘Friendship Bench’ Problem Solving Therapy,^14^ counselling for alcohol and substance use problems and prescription of psychotropic medication. When needed, both the NCD and mental health clinics refer to secondary/tertiary care in nearby government hospitals. Protocols for identifying and referring children identified as requiring medical assessment (eg, any anthropometry indicating severe malnutrition, acute illness or developmental disabilities) were also established.

### Study procedures

#### Study contacts

Participants are assessed for eligibility (table 1), recruited and have baseline assessment in the second trimester of pregnancy (or in third trimester if ‘late booker’ for antenatal care). Subsequent study contacts take place in third trimester, at delivery (when infants are recruited to the study) and postnatally at 1 week, 6 weeks, 16 weeks, 12 months, 24 months and 36 months. The schedule of study contacts is shown in table 2.

**Table 1:** Inclusion and exclusion criteria

Inclusion criteria	Age 16 or over. Ultrasound confirmed pregnancy. Resident of Lilongwe Area 25 or southern Karonga HDSS catchment areas. Signed informed consent (for 16–17 year olds who do not meet criteria for being an emancipated minor, consent of parent/guardian required).
Exclusion criteria	Not fluent in Chichewa, Chitumbuka or English. Not planning to remain living in the HDSS areas for at least the next 6 months. Too physically or mentally unwell to give informed consent.

HDSS, Health and Demographic Surveillance Site.

**Table 2:** Summary of contacts

	Antenatal contacts			Postnatal contacts						
	Eligibility	Baseline	3rd trimester	Delivery	Week 1	Week 6	Week 16	Month 12	Month 24	Month 36
Contact timing	First ANC visit (at which fetal USS done)	Soon (usually <1 week) after eligibility contact	34–39 weeks gestation	Within 24 hours of delivery.	At baby check visit (7–9 days postnatal)	At 6-week vaccination	Between 16 and 24 weeks postnatal	Between 12 and 15 months postnatal	Between 24 and 30 months postnatal	Between 36 and 42 months postnatal
Contact place	Facility - ANC (Eligibility for spouses may be done at baseline home visit instead if not present at ANC)	Home (may be seen at place of work or elsewhere if needed)	Facility - ANC	Facility - place of delivery	Facility - postnatal check clinic	Facility - vaccination clinic	Home (may be seen at place of work or elsewhere if needed)	Home	Home (may be seen at place of work or elsewhere if needed)	Home (may be seen at place of work or elsewhere if needed)
Index women	Fetal USS (routine) Eligibility Agreement to home visit	Enrolment Consent interview Anthropometry BP Biosamples	Interview Anthropometry BP Biosamples	Delivery record Interview Anthropometry BP Biosamples		Interview Biosamples	Interview Anthropometry BP		Interview Anthropometry BP Biosamples	Interview Anthropometry BP Biosamples
Spouse	If present - eligibility, agreement to home visit	Eligibility, enrolment/consent Interview Anthropometry BP Biosamples					Interview Anthropometry BP Biosamples		Interview Anthropometry BP Biosamples	Interview Anthropometry BP Biosamples
Child				Enrolment Maternal interview Anthropometry Body composition Biosamples	Maternal interview Anthropometry Body composition Biosamples	Maternal interview Anthropometry Body composition Biosamples	Maternal interview Anthropometry Body composition Biosamples	Maternal interview Anthropometry Biosamples	Maternal interview Anthropometry Biosamples	Maternal interview Anthropometry Biosamples

ANC, antenatal clinic; BP, blood pressure; USS, ultrasound scan.

#### Eligibility assessment at antenatal clinic

Pregnant women attending for their first antenatal appointment (‘booking’) are identified at the Ministry of Health (MOH) Antenatal Clinics in Area 25 HC and CRH. Research staff conduct a short group briefing about the study in the ANC waiting area. The women then proceed through routine MOH ANC procedures including fetal ultrasound scan (USS) dating. (USS scanning was introduced in the clinics as part of the National Institute for Health Research (Global Health Research (GHR) Project: 17/63/08 DIPLOMATIC collaboration).^15^ Women with a confirmed pregnancy and USS determined expected delivery date (EDD) are then seen individually by research staff in a private room and assessed for eligibility using a tablet-based questionnaire programmed using Open Data Kit. Any spouses attending alongside the women are also assessed for eligibility. Eligible individuals who are interested to take part are given a scheduled appointment for a baseline home visit, to take place usually within a week. Women who wish to discuss the study with their spouse at home are given the opportunity to do so, and visit arrangements are subsequently made by phone. Women can choose not to involve their spouse and still participate in the study. Current enrolment of the household in the HDSS is checked and details of potential participants whose household is not yet registered in the HDSS but within the geographical area covered by the HDSS are referred to the HDSS team who make a registration visit (usually within 2 days).

#### Baseline contact at home: consenting, enrolment and baseline data collection (woman and spouse)

Prearranged home visits are made by research fieldworkers working in pairs. Information sheets are read out independently to the woman and spouse (if present at the home) in the language of their choice (English, Chichewa or Chitumbuka), and consent sought. Participants who are willing and able to give informed consent, sign or provide a witnessed thumbprint on the consent form. Participants must provide consent for all procedures to be recruited, including consent for providing baseline biospecimens. They are able to withdraw consent for biosampling at later contacts and still remain in the study.

Any 16–17 year-old assessed as fulfilling criteria for being an ‘emancipated minor’ (defined as living outside family home or having other significant reason not to involve parent/guardian) can give their own informed consent. Those not considered emancipated give assent and require informed consent from parent/legal guardian, as per Malawi research ethics guidelines.

Following consenting, the fieldworker administers the questionnaire in as private a setting as possible, and conducts anthropometry and other physical measurements. A separate biosampler attends the household and conducts biosampling on participating individuals. Baseline information and samples collected are summarised in tables 3 and 4 and online supplemental table 1 and described in more detail below.

**Table 3:** Adult measures

Data	Measure/forms	Contact
Demographics		
Personal details (age, sex, marital status)	Standard MEIRU questionnaire	BL
Individual SES		
Education, occupation, parental education	Standard MEIRU questionnaire	BL
Employment status, personal income	Standard MEIRU questionnaire	BL, M12
Obstetrical/reproductive		
Gestational age/EDD by USS	Pregnancy USS	E
Current pregnancy/postnatal care info	Photo of health passport pages	BL, T3, D, W16, M12, M24, M36
Obstetrical and childbirth history	Standard MEIRU questionnaire	BL
Pregnancy intention/family planning control	Adapted London Measure of Unplanned Pregnancy /DHS questionnaires	BL, (M12, M24, M36 if pregnant)
Delivery record	Photo of delivery+midwife form	D
General health and disability		
History of diagnosed physical and mental disorders. Current medications.	Standard MEIRU questionnaires	BL
HIV and ART	Standard MEIRU questionnaires	BL, M24
Disability	WHODAS (12 item)	BL, M24
Mental health		
Depression, anxiety, somatic symptoms - current	PHQ-9; GAD7; three items from PHQ-15	BL, T3, W16, M24, M36
Lifetime major traumatic event+PTSD screen (current)	Adapted PC-PTSD	BL
Childbirth specific trauma and+PTSD screen (current) (women only)	Adapted PC-PTSD and questions from City Birth Trauma Scale	W16, M24
Lifetime depressive and manic symptoms self report (including if occurred in perinatal period)	Adapted PHQ-2+functioning Q for lifetime, 4 questions adapted from SCID, PHQ	BL
Alcohol (current and past)	AUDIT	BL
Substance use (lifetime)	ASSIST	BL
Substance use (3 months) including alcohol	ASSIST	BL, T3, W16, M24, M36
Cognitive testing		
TMB Digit Symbol, Matrix Reasoning, Visual Paired Associates		BL
Bonding		
Antenatal bonding (women only)	PAI	T3
Bonding with infant	MAI	W16
Risk and resilience		
Neuroticism	EPQ-SF Neuroticism 6 item	BL
Food insecurity	HFIAS	BL, T3, W16, M24, M36
Social support (confiding relationship)	MSPSS+identity of ‘significant other’	BL, T3, W16, M24, M36
Life events	Adapted SHARP questionnaires+ELSQ/CPAS	BL, T3, W16, M24, M36
Intimate partner violence (women only)	Adapted VAWI - WHO multicountry study	BL, W16, M24, M36
Coercive control (women only)	Adapted VAWI - WHO multicountry study	BL, M24, M36
Adverse childhood experiences	Adapted ACE-IQ	W16
Physical measures		
HR, BP		BL, W16, M24
Height, weight and body composition		BL, W16, M24
Mid upper arm circumference		BL, W16, M12, M24

ACE-IQ, Adverse Childhood Experiences - International Questionnaire; ANC, antenatal clinic; ART, antiretroviral therapy; ASSIST, Alcohol, Smoking and Substance Involvement Screening Test; AUDIT, Alcohol Use Disorders Identification Test; BL, baseline/enrolment (home); BP, blood pressure; CPAS, Creative Process Assessment Scale; D, delivery (labour ward - facility); DHS, Demographic and Health Survey; E, eligibility contact (ANC - facility); EDD, expected delivery date; ELSQ, Early Life Stress Questionnaire; EPQ-SF, Eysenck Personality Questionnaire - short form; GAD-7, 7-item Generalised Anxiety Disorder Scale; HFIAS, Household Food Insecurity Access Scale; HR, heart rate; M12, month 12 (home); M24, month 24 (home); M36, month 36 (home); MAI, Maternal Attachment Inventory; MEIRU, Malawi Epidemiology and Intervention Research Unit; MSPSS, Multidimensional Scale of Perceived Social Support; PAI, Prenatal Attachment Inventory; PC, primary care; PHQ-9, 9-item Patient Health Questionnaire; PTSD, post-traumatic stress disorder; SCID, Structured Clinical Interview for DSM ; SES, socioeconomic status; SHARP, sub-Saharan Africa Regional Partnership for Mental Health Capacity Building; T3, third trimester (ANC - facility); TMB, Test My Brain; USS, ultrasound scan; VAWI, Violence Against Women Instrument; W16, week 16 (home); WHODAS, WHO Disability Assessment Schedule.

**Table 4:** Biosamples collected in the Generation Malawi study at the different contacts

Sample	Details	Contact collected
Adult		
Blood serum	· Same day tests: full blood count and random blood glucose. · Serum, plasma and cell pellets aliquots stored at −80°C.	BL, T3, W16, M12, M24, M36
Blood plasma		
Faeces (or rectal swab)	· Diluted, aliquoted and stored at −80°C.	T3
Swabs - nasopharyngeal, oropharynx, skin, vagina	· Collected from women only using appropriate swabs and stored at −80°C.	T3
Hair	· Stored in tin foil at room temperature.	T3, W6, W16
Breast milk	· Expressed into collection tube, for centrifuge and storage at −80°C.	W6, W16
Saliva	· Women and spouses will collect saliva into sampling tubes stored at −80°C.	T3, W6, W16
Placenta	· Four placenta biopsy samples are collected approximately 4 cm from the umbilical cord. These samples are stored in formalin and in RNA protect at −80°C.	D
Infants		
Umbilical cord blood serum	· 16 mL of cord blood will be collected into EDTA and serum test tubes, then taken to the laboratory for centrifuging, dividing serum 5, plasma 3 and cell pellets stored at −80°C.	D
Umbilical cord blood plasma		
Serum blood	· Collected via heel prick into serum tube and on dry blood spots. · Samples aliquoted and frozen at −80°C.	W1, W6, W16
Faeces or rectal swab	· Collected from infants from diapers using a faeces scoop. · Diluted, aliquoted and stored at −80°C.	D, W1, W6, W16
Swabs - nasopharyngeal, oropharynx, skin	· Collected and stored at −80°C.	D, W6, W16
Urine	· Samples are collected in urine bags, pipetted into tubes, then stored at −80 °C.	W1, W6, W16
Hair	· Samples will be stored in tin foil at room temperature.	W6, W16
Saliva	· Infant saliva is collected with salivates placed in the mouth for 1 min, then centrifuged, aliquoted and stored −80°C.	W6, W16

ANC, antenatal clinic; BL, baseline/enrolment (home); D, delivery (labour ward - facility); M12, month 12 (home); M24, month 24 (home); M36, month 36 (home); T3, third trimester (ANC - facility); W1, week 1 (post-natal check - facility); W6, week 6 (under-5 clinic - facility); W16, week 16 (home).

**Table 5:** Child measures

Data	Measure/form	Contact
Childhood illness and vaccination		
Episodes of childhood illness	Direct questionnaire and health passport	W1, W6, W16, M12, M24, M36
Vaccination history	Health passport	W1, W6, W16, M12, M24, M36
HIV/ART	Standard MEIRU questionnaires	W1, W6, W16, M12, M24, M36
Current IMCI illness concern and referral	Standard MEIRU questionnaires	W6, W16, M12, M24, M36
Development		
Motor disability	Prechtls video	W16
Disability	Washington group questionnaire	M24, 36
General development	Malawi development assessment tool	W16, M12, M24, M36
Sleep history	Standard MEIRU questionnaires	W6, W16, M12, M24, M36
Feeding	Standard MEIRU questionnaires	W1, W6, W16, M12, M24, M36
Stimulation	Family care indicators	W16
Language	MacArthur Bates Questionnaire	W16, M24
Behaviour	Strengths and difficulties, START	M36
Cognition	Babyscreen	M24
Cognition	Tangerine EF Touch	M36
Mother-child interaction and adversity		
Family caregiving	Family care indicators	M12, M24<M36
Maternal and child interaction	Video and observation of maternal child interaction	W16, M12, M24, M36
Physical measures		
Height/length	Measuring board/stadiometer	D, W1, W6, W16, M12, M24, M36
Weight	SECA scale	D, W1, W6, W16, M12, M24, M36
Mid upper arm circumference	Measuring tape	D, W1, W6, W16, M12, M24, M36
Mid upper leg circumference	Measuring tape	D, W1, W6, W16, M12, M24, M36
Head circumference	Measuring tape	D, W1, W6, W16, M12, M24, M36
Chest circumference	Measuring tape	D, W1, W6, W16, M12, M24, M36
Body composition	PEAPOD	D, W1, W6, W16

ART, antiretroviral therapy; D, delivery (labour ward - facility); EF, Executive Function; M12, month 12 (home); M24, month 24 (home); M36, month 36 (home); MEIRU, Malawi Epidemiology and Intervention Research Unit; W1, week 1 (post-natal check - facility); W6, week 6 (under-5 clinic - facility); W16, week 16 (home).

#### Third trimester contact at antenatal clinic (woman only)

Participating women attending a routine ANC visit between 34+0 to 37+6 weeks gestation (from March 2025 this was adjusted to 34+0 weeks up to 39+6 weeks) are identified and invited to participate in a second study contact. In the ANC waiting area, women enrolled in the study are identified from GM study stickers in their ‘health passports’ (patient held medical record booklet) and from a participant list. Information and samples collected are summarised in tables 3 and 4 and described in more detail below. Women who do not attend a given third trimester contact (3T) ANC appointment as expected are contacted by phone and encouraged to attend on another date. Non-participation at 3T ANC contact does not preclude ongoing participation in the study.

#### Delivery contact at facility: delivery details and enrolment of child

In the rural site, most women enrolled in the study deliver at CRH, with some deliveries taking place at Karonga District Hospital some 80 km distant (either electively for primiparous women, multiple births or prior obstetrical complications or following emergency referral from CRH). In the urban site, most women deliver at Area 25 HC where full obstetrical services including instrumental deliveries and caesarean section are available.

Maternity staff at all these facilities have been briefed about the study and trained in initial study delivery procedures. The maternity staff identify women in labour who are in the study (from the health passport) and record key delivery information on a paper form including date/time, APGAR scores and birth weight and take cord blood and placental swabs/samples (and meconium if present). Maternity staff then alert the GM research team who conduct the rest of the delivery contact procedures within 24 hours. These procedures include enrolling the child in the study, collecting questionnaire data, child anthropometry (including body composition using PEAPOD air displacement plethysmography) and biosamples (tables 4 and 5). Women who have not delivered by their EDD are contacted by phone. If they have already delivered at a different facility, then the delivery questionnaire is administered by phone (or a home visit arranged) and available information collected. As long as the mother consents to enrolment of the child, the family remains in the study.

#### 1-week postnatal contact at facility baby check appointment (woman and child)

Women are given a routine appointment for initial baby check at 7 days postnatally. Most are given appointments at CRH (rural site) or Area 25 HC (urban site) though some attend at other facilities. For those attending at CRH or Area 25 HC, research staff identify women (and infants) enrolled in the study from health passport and the study register, and conduct a short questionnaire, infant anthropometry (including body composition using PEAPOD) and bio-sampling from infant and mother (tables 4 and 5).

#### 6-week postnatal contact at facility vaccination appointment (woman and child)

Women are given a routine appointment for baby vaccinations at 6 weeks postnatally. As per the initial baby check, most are given appointments at CRH or Area 25 HC though some attend at other facilities. For those attending at CRH or Area 25 HC, research staff identify women (and infants) enrolled in the study from health passport and the study register, and conduct a short questionnaire, infant anthropometry (including body composition using PEAPOD) and biosampling from infant and mother (table 5).

We identified large numbers of missing contacts at week 6 particularly in the rural site, as women were taking their babies to closer health facilities for vaccination. Therefore, from February 2024, to boost participant engagement at this contact point, we arranged to bring mothers/infants who lived far from CRH to the hospital using a study vehicle.

#### 16-week post-natal contact at home (woman, man and child)

The first postnatal home contact takes place at 16 weeks. Visits are prearranged by phone (or in person if phone contact not made). Data are collected from women, spouses and infants including questionnaires, anthropometry and other physical measures, developmental assessments, maternal-child interaction and General Movements Assessment videos as well as biosampling (tables 3–5).

#### 12-month postnatal contact at home (woman and child)

This is a shorter home contact with data collected from woman and child only, with focus on child health, including questionnaires on feeding, sleep, vaccination, anthropometry and other physical measures and biosampling (tables 3–5).

#### 24-month postnatal contact at home (woman, spouse and child)

The third postnatal home contact takes place at 24 months. Visits are prearranged by phone (or in person if phone contact not made). Data is collected from women, spouses and infants including questionnaire, anthropometry and other physical measures and biosampling (tables 3–5).

#### 36-month postnatal contact at home (woman, spouse and child)

The final postnatal home contact takes place at 36 months. Data is collected from women, spouses and infants similar to the previous visits (tables 3–5).

#### Missed contacts, outward migration and withdrawal procedures

Women, spouses and children are retained in the study for as long as we are able to maintain contact and they do not withdraw. If a woman has a miscarriage or stillbirth, she is withdrawn from the study. Similarly, if an enrolled child dies, the mother is withdrawn. Spouses are withdrawn if the woman is no longer in the study. On withdrawal, the reason for withdrawal is recorded. If a participant dies, then the family is approached after a 2-week period, and a verbal autopsy is completed. Data on miscarriages and stillbirths including outcome, timing and key maternal factors are also collected.

Participants who move out of the HDSS areas are followed up through phone calls and invited to attend their scheduled visits at the nearest MEIRU rural or urban sites. Participants still resident in the HDSS areas automatically have vital status updated monthly and annual data capture at the household level.

### Study measures

The variable domains, measures used and the contacts at which they are captured are shown in tables 3 and 5. Individual variables and measures are described in more detail below. Where specific tools are not mentioned, the variables are captured using established MEIRU question items that have been translated and validated in previous studies.

### Adult measures

#### Demographics

We collect comprehensive sociodemographic data including study site, interview language, sex, age, marital status (if polygamous, number of spouses) and ethnic background.

#### Socioeconomic status

We record socioeconomic indicators including participant education, paternal and maternal education, occupation, employment status and personal income.

#### Reproductive history (woman only)

We document reproductive history and current pregnancy details, including gravidity, parity, previous pregnancy complications, number of living children, reason for any child deaths. Details of current pregnancy including fetal USS-dated EDD.

#### Contraception and pregnancy intention (woman only)

Use of contraception prior to falling pregnant; decision-making about contraception, pregnancy intention. Questions adapted from London Measure of Unplanned Pregnancy^16^ and Demographic and Health Survey (DHS) survey.^17^

#### Medical, psychiatric and medication history, including HIV

Ever diagnosed with hypertension, diabetes, asthma, epilepsy and mental health condition. Current medication. For women only, gestational hypertension and gestational diabetes. HIV status and antiretrovirals.

#### Disability

Disability is assessed using the WHO Disability Assessment Schedule 12-item version, a shortened version of the WHO Disability Assessment Schedule V.2.0. This is a general measure of disability associated with both physical and psychological ill health.^18^

#### Mental health: current depression, anxiety, PTSD, somatic issues and suicide risk

Depressive symptoms in the past 2 weeks are measured using the PHQ-9.^19^ A Chichewa version of the PHQ-9 has been adapted and validated in Malawi.^20^ We conducted further adaptation into both Chichewa and Chitumbuka, and criterion validation against DSM-5 diagnosis of major and minor depressive episode (manuscript in preparation).

Anxiety symptoms in past 2 weeks are measured using the GAD-7.^21^ The GAD-7 has been translated into Chichewa and Chitumbuka but not previously validated.^22^ We conducted further adaptation, and criterion validation against DSM-5 diagnosis of Generalised Anxiety Disorder (manuscript in preparation). The PHQ-9 and GAD-7 are widely used and included by Wellcome in a suite of ‘common metrics’ to enhance comparability of results across studies.^23^

The PC-PTSD-5 is an ultra-brief screening tool for lifetime experience of a life-threatening event and post-traumatic stress symptoms in last month.^24^ We conducted adaptation and criterion validation against DSM-5 diagnosis of PTSD (manuscript under review). We also adapted the PC-PTSD-5 for use as a screener for childbirth-related PTSD using questions taken from City Birth Trauma Questionnaire.^25^

Somatic issues associated with psychological distress experienced in past 2 weeks (headache, stomach pain and generalised body pains) were measured with three questions from the PHQ-15.^26^

A 4-question Suicide Risk Screen adapted from The sub-Saharan Africa Regional Partnership for Mental Health Capacity Building (SHARP) study safety protocol is conducted if participant scores >0 on the PHQ-9 suicidal ideation item.^27^

#### Mental health history and neuroticism

Self-report of lifetime possible depressive episode (three items adapted from PHQ-9) and whether this occurred perinatally (women only).

Self-report of lifetime symptoms of possible mania (three items adapted from Structured Clinical Interview for DSM (SCID)^28^ and PHQ-9) and whether this occurred postnatally (women only).

Neuroticism, the tendency to negative emotional states including worry, is an established risk factor for depressive and anxiety disorders.^29^ It is measured using the six neuroticism questions from the revised abbreviated Eysenck Personality Questionnaire.^30^

#### Recent and lifetime alcohol and substance use

The Alcohol Use Disorders Identification Test, a 10-item WHO developed screening tool, is used to measure consumption and harmful use of alcohol. This tool has been adapted and used in several studies in Malawi.^31 32^ We conducted further adaptation including development of a fieldworker tool to evaluate consumption of locally brewed drinks.

Use and harmful use of other substances is screened using two questions from the Alcohol, Smoking and Substance Involvement Screening Test (ASSIST), a screening tool developed by the WHO (accessed at: https://www.who.int/substance_abuse/activities/assist_test/en/).

#### Cognition

Cognitive testing in LI-AC settings such as rural Malawi requires the use of tests that are culturally neutral and can be completed by participants of varying literacy levels. We adapted measures developed by The Many Brains Project (www.ManyBrains.net): *Matrix Reasoning* (attention, perception, cognitive control, working memory); *Digit Symbol Matching* (processing speed, visual short-term memory); and *Visual Paired Associates* (perception, declarative memory, working memory). Tests are self-administered using a tablet computer after a brief orientation by the fieldworker.

#### Bonding: antenatal and postnatal

The antenatal bond of the mother with her unborn child is measured with the Prenatal Attachment Inventory.^33^ We adapted and validated a 19 item version (Manuscript in progress). Maternal and paternal postnatal bonding with the infant is measured with the Maternal Attachment Inventory.^34^

### Psychosocial risk and resilience: social support, food insecurity, intimate partner violence, adverse childhood experiences

Perceived social support is measured using the Multidimensional Scale of Perceived Social Support, a brief measure of the respondent’s perception of the adequacy of support he/she receives from family, friends and ‘significant other’.^35^ A Chichewa version has been previously validated in Malawi.^36^

Food insecurity is measured with the Household Food Insecurity Access Scale.^37^

IPV is measured using the WHO Violence Against Women Instrument. This measures women’s experience of IPV in three domains: psychological, physical and sexual violence.^38^ In addition, questions on coercive control are included at baseline. The questions are administered via an audio tablet-assisted self-interview (ACASI) to ensure confidentiality.^39^

The Adverse Childhood Experiences - International Questionnaire (ACE-IQ) is administered to women and men using an ACASI. It asks about experience of childhood adversity in 13 domains, including exposure to violence, neglect, sexual abuse and family dysfunction. The ACE-IQ had been previously translated into Chichewa and validated and used in Malawi among adolescents.^40^

Stressful life events (including bereavement, severe illness, being a victim of crime and loss of employment) are recorded using questions adapted from the SHARP Study.^27^

### Physical measurements

Adult weight and body fat compositions are measured using OMRON body composition monitor scales. Standing height is measured with a SECA Stadiometer to the nearest 0.1 cm. Mid-upper arm circumference is measured using insertion tapes. Blood pressure is measured using calibrated electronic blood pressure monitors to a standard protocol.

### Biological samples

Biological samples from adult participants include prenatal venepuncture samples and mucosal swabs and postnatal placental sampling, saliva, hair and breast milk. They are listed in table 4, with further details described below.

### Child measures

#### Delivery

Twin or singleton, gestational age at delivery, delivery place, delivery type, APGAR scores, immediate birth weight. Routine delivery record photographed.

#### Child health and nutrition

Vaccination history, episodes of childhood illness, HIV and use of antiretroviral medications. Feeding questionnaire adapted from DHS questionnaire.^41^

#### Sleep

Infant Sleep questionnaire adapted from Brief Infant Sleep Questionnaire – Revised.^42^

#### General development

Malawi Development Assessment Tool assessing the domains of gross motor, fine motor, language and social development^43^ assessed at week 16 (W16), month 24 (M24) and month 36 (M36).

#### Neuromotor disability risk

Assessed at W16 using Prechtl General Movement Assessment.^44^ This involves video recording of the infant lying supine to detect complex ‘fidgety’ movement, absence of which indicates that the infant is at risk of neuromotor disability.

#### Stimulation in the home

This is assessed using the UNICEF-developed Family Care Indicators Questionnaire.^45^

#### Care outside the home

General information on care outside the home is assessed using the adapted Nursery School Attendance questionnaire.

#### Mother–child interaction

At W16 contact, a videoed, semi-structured mother–child interaction procedure is used^46^ that allows coding against the Qualitative Scales of the Observational Ratings of Mother-Child Interaction of the National Institute of Child Health and Human Development scales. Mother and child are recorded interacting face to face for 2 min without any toys or other stimulations (direct interaction). Then, the mother is asked to complete a caregiving activity. Lastly, the mother and child play with a set of simple, age-appropriate toys provided by the researcher for 7 min (free play). Recording is done at the participant’s home using a tablet attached to a tripod.

At W16, M24 and M36 contacts, the Observation of Mother–Child Interaction (OMCI) procedure is used. OMCI is a short observational tool created for the evaluation of parent–child interactions.^47^ Coding is completed by the fieldworker while watching a live 5-minute session of parents interacting with their child over a simple book or another play interaction. OMCI showed validity and reliability in LMIC settings including in sub-Saharan Africa.^48^ It has been adapted and used in Malawi in the neuRocog Exposed inFants ImmuNE (REFINE) study (Gladstone, personal communication). Although coded live, we also video the procedure.^49^

#### Language and communication

At M24 and M36 contacts, child language and communication is assessed using the MacArthur-Bates Communicative Development Inventories: Word and Gesture, a systematic interview to the parent (or caregiver) designed for children 8–18 months of age but which can be extended to 37 months.^50^ In Malawi, the Communicative Development Inventory has demonstrated high sensitivity in detecting developmental changes in children from 12 to 30 months of age.^51^

### Physical measurements

Infant weight (kg) is measured using Tanita Baby weighing scales. Infant length is measured using length boards. Standing height is measured with a SECA Stadiometer to the nearest 0.1 cm. Infant baby head circumference and mid-upper arm circumference are measured using insertion tapes.

The PEAPOD infant body composition system is used to assess infant body composition. This device measures infant body composition, fat and fat-free mass through air displacement plethysmography.^52^

### Biological samples

Biological samples from children include capillary blood sampling, stool, urine and mucosal swabs. They are itemised in table 3 and further details are described below.

### Biosample collection, processing and storage

Biosampling is conducted by trained personnel operating in the health facilities and travelling to participants’ homes. Biosamples at delivery (placental biopsies/swabs, cord blood and meconium if present) are collected by the maternity staff responsible for the delivery. All samples are collected to Standard Operating Procedures (SOPs) with appropriate training. Samples are transported in field cool boxes with icepacks to the laboratory within 4 hours of collection or are stored in monitored facility fridges until transport to laboratory. The SOP for biosample collection and transport (including transport in cool boxes) has been used at MEIRU in these settings for many years, for a wide range of laboratory investigations, including immunological assays, microbiological investigations, DNA and RNA extraction as well as biochemistry and haematology and the quality of the samples is preserved.

On arrival at the laboratory, the biosamples are registered and processed to SOP. Real-time assays (full blood counts and glucose) are conducted, and these results are fed back to participants by field teams. Multiple aliquots are stored in monitored and alarmed −80°C freezers and locations (box, rack and freezer) for each aliquot are documented and tracked in FreezerWorks (rural) and a customised database (urban). Duplicate sets of aliquots are transported to the other (ie, urban or rural) laboratories for secure storage to insure against catastrophic loss in either site. The laboratories have generator back-up and an on-call rota of administrative and laboratory staff to respond to emergencies.

### Management, governance and sharing of data and samples

MEIRU data are held securely to best practices as outlined in Medical Research Council policy and elsewhere.^53^ MEIRU data team ensure that highly detailed longitudinal datasets can be released publicly with minimal disclosure risk. MEIRU already makes large datasets available https://www.meiru.info/data-sets/ to ensure that Malawi populations are fully represented. We share specific datasets to individual researchers and institutions on request and rarely refuse applications. Access is governed through two cohort access committees.

### Linked data

MEIRU stores linked data on several million contacts with hundreds of thousands of participants and associated biosamples from Karonga district dating from 1979 onwards. Later data include an NCD survey 2013–2016 (rural and urban, 28 000 participants, detailed data on cardiometabolic conditions and behavioural risk factors, examinations, biosampling, aliquots stored and accessible). A long-term conditions survey within the Healthy Lives - Malawi study is currently accruing data 2021–2025 (rural and urban, 50 000 participants). These datasets include parents (preconception measures) and grandparents of Generation Malawi infants. Nested studies include kidney disease, stroke, liver disease and more, https://www.meiru.info/data-sets/. All the work is nested in rural and urban health and demographic surveillance sites, with monthly data on births, deaths, verbal autopsies and annual migration and socioeconomic individual and household data.

As of 2024, the data and sample collections are sufficient to promote analysis and sharing. The Data and Biorepository Access Committees, convened as part of the Malawi-led governance of the resource, will make decisions on the availability and security levels for each tranche of data and to consider custom requests for datasets and access to biosamples according to defined criteria. In future requests for data or sample access will be processed through the MEIRU data repository platforms, prior to that launch requests can be made by contacting info@meiru.mw

### Data sharing

Data will be Findable, Accessible, Interoperable and Reusable. Current data sharing procedures involve the recipient signing a MEIRU data use agreement and being provided access to a de-identified unlinkable analysis dataset. In future, we will capitalise on the African Population Cohort Consortium Trusted-Research-Environment-in-a-box platform currently under development, to enable permitted researchers to access data and analytical tools in a ‘safe haven’ without ability to download datasets.

### Sample sharing

The nature and volumes of biosamples collected are balanced between storage of adequate volume for repeated access relating to different studies and avenues of research, either internally or externally generated. The samples constitute a valuable, depletable resource and access is governed by a Biopository Access Committee. Analysis of Generation Malawi samples requires reciprocal sharing of laboratory assay data generated, which then becomes sharable in turn, to maximise the value of the resource.

## Statistical analysis

Baseline characteristics will be summarised using standard descriptive statistics. Continuous variables will be reported as means with SD for normally distributed data, or medians with IQRs for skewed data. Categorical variables will be presented as frequencies and percentages. Unadjusted associations between exposures (eg, SES, rural/urban residency, education) and outcomes (depression, anxiety, PTSD, birth outcomes and infant growth) will be assessed using Pearson’s χ^2^ for categorical variables. Associations between exposures and continuous outcomes will be compared across groups using independent samples t-tests or one-way analysis of variance, as appropriate.

For multivariable analyses, to formalise structural assumptions and mitigate confounding Directed Acyclic Graphs (DAGs) will be developed a priori using expert knowledge and existing literature to map the assumed causal structures of exposures, covariates, mediators and outcomes. DAGs will be used to identify the minimally sufficient adjustment sets required to estimate the total effects of our primary exposures, ensuring adjustment for true confounders while avoiding conditioning on colliders or mediators.

To evaluate the effect of exposures on longitudinal outcomes, while accounting for the correlation of repeated measures within individuals over time, mixed-effects modelling will be applied. Linear mixed-effects models will be used to analyse continuous outcomes, such as infant growth, over follow-up waves. For binary or count outcomes, such as the clinical presence of depression or PTSD, generalised linear mixed models will be applied. All longitudinal models will incorporate exposure-by-time interaction terms to assess whether the rates of change in the outcomes differ according to exposure status and will include random intercepts to account for baseline clustering.

To identify mediating pathways between exposures and outcomes, causal mediation analyses will be conducted. To overcome the limitations of traditional mediation approaches, which are biased in the presence of exposure-induced mediator-outcome confounding, and strict assumptions surrounding the causal order of the mediators and linearity assumptions, we will estimate interventional direct and indirect effects.^54^ Further, we will adapt this approach to account for both time-varying confounders and time-varying mediators across the follow-up waves.^55^

Patterns of missing data will be assessed. If appropriate we will employ multiple imputation by chained equations to generate imputed datasets assuming data is missing at random. Primary multivariable analyses will be conducted on the imputed data, and pooled results will be reported using Rubin’s rules. To assess the robustness of our findings and evaluate potential bias introduced by attrition or non-participation, sensitivity analyses will be performed comparing the imputed results with complete-case analyses.

Analyses will be performed using Stata V.18.0/R V.4.3.0.

## Discussion

### Strengths and limitations

The Generation Malawi study offers a unique and valuable resource for understanding intergenerational and life-course influences on health in a low-income African context, where such data are scarce. Several key strengths enhance the scientific value and sustainability of this cohort. The study is implemented in both urban and rural settings, allowing comparative analyses across diverse socio-environmental contexts. It is nested within an established HDSS, enabling accurate linkage to demographic, morbidity and mortality data, as well as to other ongoing studies. The broad consent for biological sample storage and future use provides opportunities for a wide range of analyses, including genetic, epigenetic and biomarker studies.

The study has several potential limitations. Loss to follow-up over time may introduce selection bias and reduce the completeness of outcome data, particularly for longitudinal analyses. Lower participation and retention among fathers may limit the ability to fully assess paternal contributions and intergenerational influences on child health outcomes. Additionally, recruitment is based on antenatal care attendance and residence within the HDSS catchment areas, which may limit generalisability and introduce selection bias by excluding women who do not access antenatal care or populations living outside the surveillance areas.

### Future strategy and sustainability

Funds permitting, the Generation Malawi has been established with the intention that follow-up should continue for as long as new questions can be addressed and cohort retention is adequate to provide a good and representative sample size of infants (immediate), young children (within the scope of this project) and school-age children and adolescents (within the scope of future applications). These follow-up assessments should be conducted at least annually to ensure ongoing engagement with cohort participants.

Shifting cohort data collection to routine data linkage and low-level cohort maintenance is highly unlikely in the next decade. (1) Infrastructure is lacking: Routine data (births, deaths, health service contacts) in Malawi are at best patchy and often completely absent, owing to under-resourced human resources and infrastructure. (2) Data are of poor quality: Vital registration is incomplete and often excludes the most vulnerable. Furthermore, as we have shown, individuals with long-term conditions are predominantly undiagnosed, with little indication of change.

The future African Population Cohorts Consortium is likely to characterise this cohort as highly valuable, increasing the likelihood of supplementary funding through an African Population Cohorts Consortium (APCC) funder network. Given the interest to date, we predict multiple further requests for associate research to contribute to operational costs (other MEIRU Longitudinal Population Study contributions from awards to date range from 10% to 50% of their cost).

### Plans to collaborate

MEIRU actively shares data and samples across several research networks and is active in a work package within the African Population Cohorts Consortium to develop standardised tools and harmonised datasets. We collaborate with partners in Malawi and the UK on studies exploring the early origins of cardiometabolic risk, the genomics of depression and anxiety, the effects of maternal malaria on infant immunity, the role of gut pathogens in child growth and the role of early-life malnutrition in later-life NCD risk (CHild malnutrition and Adult NCD: Generating Evidence on mechanistic links to inform future policy/practice (CHANGE) project).^56^ MEIRU also contributes to a Wellcome working group on longitudinal mental health data and works closely with international cohorts, including Avon Longitudinal Study of Parents and Children (ALSPAC), Born in Bradford, the Dutch Birth Cohort, Kula Leap, C-Gull (Liverpool) and emerging Ethiopian birth cohorts, to strengthen future collaborations.

## Ethics and dissemination

This study protocol was approved by the National Health Science Research Committee in Malawi (number: 21/01/2653) and the Ethics Committee of the College of Medical Veterinary and Life Sciences at the University of Glasgow, UK (protocol number: 200190183). The formative work for this study was granted ethical clearance by the College of Medicine Research Ethics Committee (protocol number P.11/19/2865).

Findings and interpretations from this study will be made public through policy briefs, seminars, local and international conferences and publications in peer-reviewed open-access journals. As part of the dissemination plans, the findings will be made available locally to different channels, including regional and national meetings of Preventive Health and NCDs and Mental Health at the Ministry of Health.

Through a Wellcome Trust funded public engagement award, we have created a documentary ‘Growing Up in Malawi’ featuring 20 families participating in Generation Malawi. Interviews, film of birth cohort activities and home-life, and family-shot footage of daily life and key events during pregnancy and early infancy are interwoven to the experience of participating in a birth cohort as well as the experience of expecting a new child in rural and urban Malawi (https://www.youtube.com/watch?v=igzj8ixC3ao). In collaboration with the African Alliance of Maternal Mental Health, we have also created maternal mental health advocacy videos featuring women from the birth cohort experiencing mental health conditions who have agreed to share their stories to help others (https://www.youtube.com/watch?v=oiTYNg6hieI).

## Supplementary material

10.1136/bmjopen-2026-122132 online supplemental file 1

## References

[1] Malawi Global hunger index (ghi) - peer-reviewed annual publication designed to comprehensively measure and track hunger at the global, regional, and country levels.

[2] World Obesity Day Atlases (2025). Obesity atlas 2025.

[3] Mandy M, Nyirenda M (2018). Developmental Origins of Health and Disease: the relevance to developing nations. Int Health.

[4] Hoffman DJ, Reynolds RM, Hardy DB (2017). Developmental origins of health and disease: current knowledge and potential mechanisms. Nutr Rev.

[5] Barker DJP (2002). Fetal programming of coronary heart disease. Trends Endocrinol Metab.

[6] MacBeth A, Morales MF, Golds L (2026). Intergenerational transmission of mental health during the first 1,000 days of life. *Nat Rev Psychol*.

[7] Chan JK, Marzuki AA, Vafa S (2024). A systematic review on the relationship between socioeconomic conditions and emotional disorder symptoms during Covid-19: unearthing the potential role of economic concerns and financial strain. BMC Psychol.

[8] Reynolds RM (2013). Glucocorticoid excess and the developmental origins of disease: two decades of testing the hypothesis--2012 Curt Richter Award Winner. Psychoneuroendocrinology.

[9] Borre YE, O’Keeffe GW, Clarke G (2014). Microbiota and neurodevelopmental windows: implications for brain disorders. Trends Mol Med.

[10] von Stumm S, Kandaswamy R, Maxwell J (2023). Gene-environment interplay in early life cognitive development. Intelligence.

[11] Crampin AC, Kayuni N, Amberbir A (2016). Hypertension and diabetes in Africa: design and implementation of a large population-based study of burden and risk factors in rural and urban Malawi. Emerg Themes Epidemiol.

[12] Ndambo MK, Pickersgill M, Bunn C (2023). Maternal mental health research in Malawi: Community and healthcare provider perspectives on acceptability and ethicality. SSM Ment Health.

[13] Ndambo MK, Bunn C, Pickersgill M (2024). Can biosampling really be “non-invasive”? An examination of the socially invasive nature of physically non-invasive biosampling in urban and rural Malawi. Glob Bioeth.

[14] Chibanda D (2017). Reducing the treatment gap for mental, neurological and substance use disorders in Africa: lessons from the Friendship Bench in Zimbabwe. Epidemiol Psychiatr Sci.

[15] von Wissmann B, Wastnedge E, Waters D (2020). Informing prevention of stillbirth and preterm birth in Malawi: development of a minimum dataset for health facilities participating in the DIPLOMATIC collaboration. BMJ Open.

[16] Hall J, Barrett G, Mbwana N (2013). Understanding pregnancy planning in a low-income country setting: validation of the London measure of unplanned pregnancy in Malawi. BMC Pregnancy Childbirth.

[17] National Statistical Office and ICF (2017). Malawi demographic and health survey 2015-16.

[18] Ustün TB, Chatterji S, Kostanjsek N (2010). Developing the World Health Organization Disability Assessment Schedule 2.0. Bull World Health Organ.

[19] Kroenke K, Spitzer RL, Williams JB (2001). The PHQ-9: validity of a brief depression severity measure. J Gen Intern Med.

[20] Udedi M, Pence BW, Stewart RC (2020). Detection and prevalence of depression among adult type 2 diabetes mellitus patients attending non-communicable diseases clinics in Lilongwe, Malawi. *Int J Ment Health Syst*.

[21] Spitzer RL, Kroenke K, Williams JBW (2006). A brief measure for assessing generalized anxiety disorder: the GAD-7. Arch Intern Med.

[22] Barreix M, Barbour K, McCaw-Binns A (2018). Standardizing the measurement of maternal morbidity: Pilot study results. Int J Gynaecol Obstet.

[23] Farber GK, Gage S, Kemmer D (2023). A Collaborative Effort to Establish Common Metrics for Use in Mental Health. JAMA Psychiatry.

[24] Prins A, Bovin MJ, Smolenski DJ (2016). The Primary Care PTSD Screen for DSM-5 (PC-PTSD-5): Development and Evaluation Within a Veteran Primary Care Sample. J Gen Intern Med.

[25] Ayers S, Wright DB, Thornton A (2018). Development of a Measure of Postpartum PTSD: The City Birth Trauma Scale. Front Psychiatry.

[26] Kroenke K, Spitzer RL, Williams JBW (2002). The PHQ-15: validity of a new measure for evaluating the severity of somatic symptoms. Psychosom Med.

[27] Gaynes BN, Akiba CF, Hosseinipour MC (2021). The Sub-Saharan Africa Regional Partnership (SHARP) for Mental Health Capacity-Building Scale-Up Trial: Study Design and Protocol. Psychiatr Serv.

[28] First MB, Williams JBW, Benjamin LS (2015). User’s Guide for the SCID-5-PD (Structured Clinical Interview for DSM-5 Personality Disorder).

[29] Trull TJ, Widiger TA (2013). Dimensional models of personality: the five-factor model and the DSM-5. Dialogues Clin Neurosci.

[30] Francis LJ, Brown LB, Philipchalk R (1992). The development of an abbreviated form of the revised Eysenck personality questionnaire (EPQR-A): Its use among students in England, Canada, the U.S.A. and Australia. Pers Individ Dif.

[31] Lancaster KE, Lungu T, Mmodzi P (2017). The association between substance use and sub-optimal HIV treatment engagement among HIV-infected female sex workers in Lilongwe, Malawi. AIDS Care.

[32] Lancaster KE, MacLean SA, Lungu T (2018). Socioecological Factors Related to Hazardous Alcohol use among Female Sex Workers in Lilongwe, Malawi: A Mixed Methods Study. Substance Use & Misuse.

[33] Muller ME (1993). Development of the Prenatal Attachment Inventory. West J Nurs Res.

[34] Müller ME (1994). A questionnaire to measure mother-to-infant attachment. J Nurs Meas.

[35] Zimet GD, Dahlem NW, Zimet SG (1988). The Multidimensional Scale of Perceived Social Support. J Pers Assess.

[36] Stewart RC, Umar E, Tomenson B (2014). A cross-sectional study of antenatal depression and associated factors in Malawi. *Arch Womens Ment Health*.

[37] Coates J, Swindale A, Bilinsky P (2007). Food and Nutrition Technical Assistance Project Academy for Educational Development.

[38] Jansen H, Watts C, Ellsberg M (2004). Interviewer Training in the WHO Multi-Country Study on Women’s Health and Domestic Violence. Violence Against Women.

[39] Hossain M, Heise L (2017). STRIVE Technical Brief: Measuring Intimate Partner Violence; London School of Hygiene and Tropical Medicine.

[40] Kidman R, Smith D, Piccolo LR (2019). Psychometric evaluation of the Adverse Childhood Experience International Questionnaire (ACE-IQ) in Malawian adolescents. Child Abuse Negl.

[41] (2021). Breastfeeding and complementary feeding.

[42] Sadeh A (2004). A brief screening questionnaire for infant sleep problems: validation and findings for an Internet sample. Pediatrics.

[43] Gladstone M, Lancaster GA, Umar E (2010). The Malawi Developmental Assessment Tool (MDAT): the creation, validation, and reliability of a tool to assess child development in rural African settings. PLoS Med.

[44] Einspieler C, Bos AF, Libertus ME (2016). The General Movement Assessment Helps Us to Identify Preterm Infants at Risk for Cognitive Dysfunction. Front Psychol.

[45] Hamadani JD, Tofail F, Hilaly A (2010). Use of family care indicators and their relationship with child development in Bangladesh. J Health Popul Nutr.

[46] Lotzin A, Lu X, Kriston L (2015). Observational tools for measuring parent-infant interaction: a systematic review. Clin Child Fam Psychol Rev.

[47] Rasheed MA, Yousafzai AK (2015). The development and reliability of an observational tool for assessing mother-child interactions in field studies- experience from Pakistan. Child Care Health Dev.

[48] Bozicevic L, Lucas C, Magai DN (2025). Evaluating caregiver-child interactions in low- and middle-income countries: a systematic review of tools and methods. J Reprod Infant Psychol.

[49] Buchwald AG, Crespo-Llado MM, Maliwichi L (2025). The effect of environment and in-utero HIV exposure on neurodevelopment in infants. AIDS.

[50] Alcock KJ, Rimba K, Holding P (2015). Developmental inventories using illiterate parents as informants: Communicative Development Inventory (CDI) adaptation for two Kenyan languages. J Child Lang.

[51] Prado EL, Phuka J, Ocansey E (2018). A method to develop vocabulary checklists in new languages and their validity to assess early language development. J Health Popul Nutr.

[52] Wiechers C, Kirchhof S, Maas C (2019). Neonatal body composition by air displacement plethysmography in healthy term singletons: a systematic review. BMC Pediatr.

[53] (2023). MRC data sharing policy.

[54] Vansteelandt S, Daniel RM (2017). Interventional Effects for Mediation Analysis with Multiple Mediators. Epidemiology.

[55] Tai AS, Lin SH, Chu YC (2023). Causal Mediation Analysis with Multiple Time-varying Mediators. Epidemiology.

[56] Kerac M, Anujuo K, Lelijveld N (2022). LSHTM Data Compass. Lshtmacuk.

