# Supplementary material for: Generation Malawi: a family-based birth cohort investigating physical and mental health conditions in rural and urban Malawi – study protocol
Source: BMJ Open. 2026 Sep 15;16(9):e122132. doi: 10.1136/bmjopen-2026-122132 (PMC13583881; doi:10.1136/bmjopen-2026-122132)
Supplement: Supplementary file 1 [file bmjopen-2026-122132.reviewer_comments.pdf]

PEER REVIEW HISTORY

BMJ Open publishes all reviews undertaken for accepted manuscripts. Reviewers are asked to complete a checklist review form and are provided with free text boxes to elaborate on their assessment. These free text comments are reproduced below.

ARTICLE DETAILS

Title (Provisional)

Generation Malawi, a family-based birth cohort investigating physical and mental health conditions in rural and urban Malawi: study protocol

Authors

Nakanga, Wisdom; Stewart, Robert C.; Malava, Jullita Kenala; Nindi, Providence; Nzawa-Soko, Rebecca; Nyanjagha, Innocent; Mainjeni, Angella; Gondwe, Thandile; Umar, Eric; Manda-Taylor, Lucinda; Chorwe-Sungani, Genesis; Gladstone, Melissa; Kerac, Marko; MacBeth, Angus; Seward, Nadine; Patson, Noel; Ngongondo, McNeil; Collaborator Group, Generation Malawi; Mathanga, Don; McIntosh, Andrew; Crampin, Amelia C

VERSION 1 - REVIEW

**Reviewer**	**1**
**Name**	**Moron-Duarte, Lina**
**Affiliation**	**Universidade Federal de Pelotas Departmento de Medicina Social, Post-Graduate Program in Epidemiology**
**Date**	**14-Jun-2026**
**COI**	**None**

Although the study is described as a multigenerational family cohort, the design is fundamentally a birth cohort with prenatal recruitment of mothers and fathers. I recommend clarifying the primary unit of follow-up and the conceptual role of parental participants within the study's analytical framework.

The scientific rationale is strong; however, the specific objectives and primary hypotheses are not clearly defined. It would be helpful to identify the priority outcomes, key exposures of interest, and the research questions that will guide future analyses.

Sample size determination: the sample size justification is limited. Although the study plans to recruit approximately 5,000 pregnant women, it would be important to provide formal power calculations or examples of the effect sizes that the study is designed to detect for its main outcomes.

Methodology:

the inclusion of a conceptual framework or causal diagram (DAG) would strengthen understanding of the expected relationships between prenatal exposures, intergenerational factors, and physical and mental health outcomes.

Withdrawal procedures:the protocol states that women experiencing a stillbirth are withdrawn from the study. Given that stillbirth is an important perinatal outcome and that the study collects extensive prenatal information, it would be helpful to justify this decision and clarify whether these events will be included in specific analyses of prenatal risk factors and adverse pregnancy outcomes.

Statistical analysis:The protocol provides a comprehensive description of data collection but limited information regarding the planned analytical approaches. I recommend including a general statistical analysis section describing the methods anticipated for longitudinal data, repeated measures, and intergenerational analyses.

Study measures:given the large number of exposures, biomarkers, and outcomes collected, it would be helpful to indicate how the primary analyses will be prioritized and how the risk of spurious findings arising from multiple comparisons will be addressed.

The current limitations mainly focus on loss to follow-up and paternal participation. I suggest expanding this section to include potential selection biases arising from recruitment based on antenatal care attendance and residence within HDSS areas.

VERSION 1 - AUTHOR RESPONSE

**COMMENTS FROM REVIEWER 1: Dr. Lina Moron-Duarte, Universidade Federal de Pelotas Departmento de Medicina Social**		
1	Although the study is described as a multigenerational family cohort, the design is fundamentally a birth cohort with prenatal recruitment of mothers and fathers. I recommend clarifying the primary unit of follow-up and the conceptual role of parental participants within the study's analytical framework.	Thank you for this important observation. Generation Malawi is a family-based birth cohort with prenatal recruitment, in which pregnant women, their partners, and children born into the cohort are followed longitudinally. While the birth cohort component is centred on the child as the primary offspring generation, parental participants are also followed over time to capture intergenerational, familial, genetic, environmental, and health-related factors.<br>The continued follow-up of both parents and children enables analyses of how parental characteristics and exposures influence child health and development,

		as well as how health trajectories evolve across family members over time.
2	The scientific rationale is strong; however, the specific objectives and primary hypotheses are not clearly defined. It would be helpful to identify the priority outcomes, key exposures of interest, and the research questions that will guide future analyses.	Thank you for the comment. We have added a section detailing the study objectives, with the main objective of establishing a multi-generational family/birth cohort (nested within the Healthy Lives Malawi open, population-based cohorts) in rural (southern Karonga District) and urban (high-density sectors of Lilongwe Area 25) sites, through which to study the longitudinal course and pregnancy, early-life and intergenerational effects for long-term mental and physical health conditions in Malawi. On page 13, we have also included the specific objectives that detail the primary analysis we intend to conduct, including the primary outcomes of interest.
3	Sample size determination: the sample size justification is limited. Although the study plans to recruit approximately 5,000 pregnant women, it would be important to provide formal power calculations or examples of the effect sizes that the study is designed to detect for its main outcomes.	Thank you for this important comment. We have expanded the sample size justification to include example power calculations for key study outcomes. Given the broad range of exposures and outcomes assessed in this longitudinal cohort, the study was not designed to detect a single specific effect size; rather, power will vary depending on the prevalence of the exposures and outcomes under investigation.<br>For example, with a planned sample size of approximately 5000 pregnant women, the study will have >90% power to detect an association between a maternal depression outcome with a prevalence of 10% and a risk factor with an odds ratio of 1.2. Similar power considerations will be assessed for key outcomes as analyses are developed, taking into account outcome prevalence, exposure distribution, and available follow-up data. We have added this clarification to the manuscript on page 14.
4	Methodology: the inclusion of a conceptual framework or causal	Thank you for this helpful comment. We agree that clearly outlining the expected relationships between

	diagram (DAG) would strengthen understanding of the expected relationships between prenatal exposures, intergenerational factors, and physical and mental health outcomes.	prenatal exposures, intergenerational factors, and health outcomes is important. We have strengthened the description of the conceptual relationships underpinning the study in the Study Objectives, Study Measures, and Statistical Analysis sections. These sections now clarify the hypothesised pathways linking maternal, paternal, and early-life exposures to child physical and mental health outcomes, and the planned analytical approaches to account for potential confounding and intergenerational influences.<br>This approach provides a clear framework for interpreting the relationships examined in the study while allowing flexibility as new research questions emerge from this longitudinal cohort.
5	Withdrawal procedures: the protocol states that women experiencing a stillbirth are withdrawn from the study. Given that stillbirth is an important perinatal outcome and that the study collects extensive prenatal information, it would be helpful to justify this decision and clarify whether these events will be included in specific analyses of prenatal risk factors and adverse pregnancy outcomes.	Women are withdrawn from the study in cases of participant death, foetal loss, or child's death. Prior to withdrawal, we conduct a verbal autopsy and an exit interview in all such cases. The information gathered from these interviews will be incorporated into future analyses of prenatal risk factors and adverse pregnancy outcomes. This has been added to the manuscript. The families are offered ongoing support through our mental health clinics and counselling services.
6	Statistical analysis: The protocol provides a comprehensive description of data collection but limited information regarding the planned analytical approaches. I recommend including a general statistical analysis section	Thank you for this comment. We have added a Statistical Analysis section describing the planned analytical approaches, including longitudinal, repeated measures, and intergenerational analyses on page 13.<br>The revised section describes the planned descriptive analyses and statistical methods for evaluating

	describing the methods anticipated for longitudinal data, repeated measures, and intergenerational analyses.	primary and secondary outcomes, including maternal mental health, birth outcomes, infant growth, and associated risk factors. Regression models will be used to examine associations between maternal, infant, and family-level exposures and outcomes while adjusting for potential confounders. For longitudinal data, mixed-effects models or other appropriate approaches will be used to account for repeated measurements and clustering within individuals over time. Intergenerational analyses will assess associations between parental exposures and offspring outcomes, incorporating relevant maternal, paternal, and environmental factors. Missing data will be evaluated and addressed using appropriate methods, including multiple imputation where applicable.<br><br>We also describe how we will develop Directed Acyclic Graphs for each of our analyses to ensure we are accounting for a minimal adjustment set, avoiding the inclusion of covariates such as colliders that can influence our estimates.<br><br>Lastly, we describe our approach to mediation analyses, that describes how we will adapt interventional effects, a robust causal inference based approach to mediation, to account for time-varying mediators and confounders
7	Study measures: given the large number of exposures, biomarkers, and outcomes collected, it would be helpful to indicate how the primary analyses will be prioritized and how the risk of spurious findings arising	Thank you for this important comment. We agree that the Generation Malawi cohort includes a wide range of exposures, biomarkers and outcomes, and that prioritization of analyses is important to reduce the risk of spurious findings from multiple comparisons. The primary analyses will focus on key outcomes and exposures aligned with the main

	from multiple comparisons will be addressed.	study objectives, while secondary and exploratory analyses will be clearly distinguished. Where multiple comparisons are conducted, we will consider appropriate approaches to account for multiple testing. We will also interpret all findings in the context of biological plausibility, consistency across analyses, and replication where possible.<br><br>We will also consider applying for recent approaches to reduce biases associated with testing for multiple moderators, such as causal forests.
8	The current limitations mainly focus on loss to follow-up and paternal participation. I suggest expanding this section to include potential selection biases arising from recruitment based on antenatal care attendance and residence within HDSS areas.	Thank you for this important comment. We have revised the limitations section to expand beyond loss of follow-up and paternal participation. We have included potential selection biases related to recruitment through antenatal care attendance and residence within the HDSS catchment areas, noting that this may affect the representativeness and generalizability of study findings.

VERSION 2 - REVIEW

Reviewer 1

Name Moron-Duarte, Lina

Affiliation Universidade Federal de Pelotas Departmento de Medicina Social, Post-Graduate Program in Epidemiology

Date 11-Aug-2026

COI

The authors have addressed all the requested revisions. I consider the manuscript suitable for publication in its current form.
