# Supplementary material for: Generation Malawi: a family-based birth cohort investigating physical and mental health conditions in rural and urban Malawi – study protocol
Source: BMJ Open. 2026 Sep 15;16(9):e122132. doi: 10.1136/bmjopen-2026-122132 (PMC13583881; doi:10.1136/bmjopen-2026-122132)
Supplement: Supplementary file 2 [file bmjopen-16-9-s001.docx]

# Supplementary table 1 gm baseline questionnaire USER guide

# 1.0 INtroduction

The use of the GM baseline questionnaire is to collect information regarding GM in a standardised way. The reliability of the questionnaire depends on the behaviour of the interviewer, and therefore it is important that the questions are read exactly as they are, and that no non-verbal clues are given.

1.1 **Pre-requisites**
- Interviews should take place where there is minimal disturbance, where both interviewer and subject can be comfortable, and where eye contact and hence the attention of the subject is maintained.
- The interview must not start until when the interviewer has the subject’s full attention.
  1.2 **Standardization**
- Every Field Interviewer must administer every question in the questionnaire to every respondent in the same way. Ensuring consistency minimises variability between interviews and the potential for bias, two factors that can seriously undermine the quality (reliability and validity) of the data gathered in an interviewer-led survey.

Please follow the guidelines listed below to ensure that you are administering the questionnaire in a standardized manner.

2.0 **INTERVIEWING TIPS**
    2.1 **ASKING THE QUESTIONS**

The following is a list of protocols to which you must adhere when administering the GM baseline questionnaire.

- Ask the questions using the exact words. All questions must be read exactly as they appear in the questionnaire; to do otherwise risks invalidating the survey.
- Read the questions slowly. As you become familiar with GM questions, it is tempting to begin reading through the questions more quickly. Remember, however, that this is the first time the respondent has heard these questions. You must read slowly enough to allow the respondent time to understand everything you are asking.
- Ask every question that is specified in the questionnaire. Never presume that the answer to an upcoming question has already been provided by a previous answer. The answer received in the context of one question may not be the same answer in the context of another.
- Read the complete question as displayed. The respondent may interrupt you and answer before having heard the complete question. When this happens, read the question again, making sure the respondent hears the question through to the end. Do not assume a premature response applies to the question as written. You may politely inform the respondent that to do your job you must read the entire question.
- Repeat questions that are misinterpreted or misunderstood by the respondent. The respondent might tell you that he or she did not understand the question, or he or she might look confused when trying to answer the question. The respondent also might give an answer that seems illogical or irrelevant to the question. In any of these circumstances, you should simply repeat the question exactly as it is written. However, if there is no specific guidance provided about the question, you should NOT offer any explanations beyond what is provided in the questionnaire.
- Do not suggest answers to the respondent. As you proceed through the interview, you will come across questions that you might think you know the answers to be based on prior information you have heard. You may feel the urge to suggest answers to the respondent. Resist this urge. Read the question as written. Consistent, nonbiased administration strengthens the validity of the data collected.
- Read introductory and transitional statements as they appear in the questionnaire. These statements often contain instructional material for the respondent.
- Responses must represent the respondent’s own opinions without bias introduced by the Field Interviewer. Do not influence a respondent’s answers with your behavior (that is, with your body language, your attitude, your tone of voice, or any other way). By observing these rules of questionnaire administration, you ensure that the survey is administered to each respondent in the same way and that the responses given by the participant accurately represent his or her experiences and opinions. This practice will guarantee that scientific principles are followed in the administration of the questionnaire and that the data are of the highest quality.
  2.2 **RECORDING THE REPLIES TO THE QUESTIONS**
- For questions with ‘YES’ or ‘NO’ responses only and where there is no option for a ‘DON’T KNOW’ response, if the subject is uncertain of the answer, recorded as ‘NO’.
- If the answer to the question is a number, this should be recorded directly in the spaces provided. Where the answer is a date, this should be selected from the pop-up date displayed on the tablet.
- If an answer of ‘YES’ or ‘NO’ is required and the subject does not understand the question even when repeated, the answer is coded as ‘NO’, (unless a ‘DON’T KNOW’ option is specifically provided).
- Where an answer is required to a quantitative or semi-quantitative question, the subject’s ‘best guess’ may be accepted.
- The interviewer should follow instructions given in the questionnaire regarding which questions to ask according to the subject’s response.
  2.3 **FOLLOWING SKIP PATTERNS**

It is very important not to ask a respondent any questions that are not relevant to his or her situation. For example, you should not read a pregnancy question to a man. In cases where a particular response makes subsequent questions irrelevant, a skip pattern has been provided in the programmed questionnaire to skip to the next appropriate question.

2.4 **TRAINING**

Before starting the survey, the questionnaire and instructions should be studied, and any difficulties discussed. Interviewers must become familiar with the flow of questions. Interviewers should test the questionnaire on 10 or more subjects. These interviews should be witnessed by an experienced person who can identify mistakes or doubtful points that need clarification.

2.5 **PROBING**

Probing is a technique used to help ensure that the answers given by a respondent are as accurate and as complete as possible. Effective probes serve two purposes:

(1) they encourage a respondent to express him or herself completely, and

(2) they help the respondent focus on the specific requirements of the question.

To know when to use a probe, you must be thoroughly familiar with the questionnaire and know the objectives of each question; that is, you must know what is being measured and what constitutes an acceptable response. Otherwise, you will have difficulty judging the ‘adequacy’ of a response. Only neutral or nondirective probes (those that do not influence the respondent) should be used in the GM interview. Some examples of proper probing techniques follow.

- ***Neutral questions or statements***. These probes encourage a respondent to explain further or elaborate on a response without leading or directing the respondent to a particular answer. These must be stated in a neutral or nonchallenging tone. Some examples of neutral probes include the following: – “What do you mean?” – “Please explain that.” – “Which would you say is closest?”
- ***The silent probe***. A timely pause is the easiest and often the most useful type of probe. This pause lets your respondent know that you are expecting or waiting for additional information.
- ***Clarification***. Use clarification probes when you judge the respondent’s answer to be unclear, inconsistent, ambiguous, or contradictory. You must take care, however, not to appear to challenge the respondent. Instead, tactfully express concern over not completely understanding the nature of the response. Some examples include the following: – “I’m not quite sure I understand what you mean by that. Could you tell me a little more?” – “I’m sorry, but a few minutes ago I thought you said [CONTRADICTORY INFORMATION]. Could you clarify this for me?”. Another important section to use this technique is when the participant is using the visual aids, when an interviewer will repeat the option they select (using the word it stands for) to ensure that the participant understands the use of the aid.

|  | **English** | **code** | **English (options)** | **Chichewa** | **Chichewa (options)** | **Tumbuka** | **Tumbuka (options)** | **USER GUIDE** |
| --- | --- | --- | --- | --- | --- | --- | --- | --- |
|  | **SOCIODEMOGRAPHIC DATA** |  |  | **SOCIODEMOGRAPHIC DATA** |  | **SOCIODEMOGRAPHIC DATA** |  |  |
| *STDYPL* | Study site | 1  2 | 1. Karonga  2. Lilongwe | Study site | 1. Karonga  2. Lilongwe | Study site | 1. Karonga  2. Lilongwe | Provide the study site. Only two options available as per enrolment sites for GM. |
| *INTDATE* | Interview date |  |  | Interview date |  | Interview date |  | Enter date interview is taking place. Choose from the tablet. |
| *STID* | Study ID |  |  | Study ID |  | Study ID |  | Enter study identification number. This is the number given to participant on the consent form. Please ensure entered number correctly matches number on the consent form. |
| *STID2* | Re-enter Study ID |  |  | Re-enter Study ID |  | Re-enter Study ID |  | Re- enter study identifier. If the STID do not match with the first entry, you will be prompted to recheck and make sure the STID is correct. |
| *CNO* | Contact number |  |  | Contact number |  | Contact number |  | Enter appropriate contact number as on the stickers. This changes for every completed visit. This will be re-entered to ensure correctness. |
| *GCRS* | CRSNO (For Karonga Only) |  |  | CRSNO (For Karonga Only) |  | CRSNO (For Karonga Only) |  | Record CRS number from field log provided for Karonga site only. The tablet will not allow you to skip this as all participants will have CRS numbers. This is re-entered to ensure correctness. |
| *REPGP* | Reporting group |  |  | Reporting group |  | Reporting group |  | Enter reporting group as appearing on field log. The tablet will not allow you to go to next question if reporting group entered in not two digits. Enter ‘98’ if unknown. |
| *CLUSTER* | Cluster |  |  | Cluster |  | Cluster |  | Enter cluster group as appearing on field log. The tablet will not allow you to go to next question if cluster entered in not two digits. Enter ‘98’ if unknown’. |
| *HHLISTNO* | House Number |  |  | House Number |  | House Number |  | Enter household number as appearing on field log. The tablet will not allow you to go to next question if household number entered in not three digits. Enter ‘9998’ if unknown’ |
| *MEMBNO* | Member Number |  |  | Member Number |  | Member Number |  | Enter member number as appearing on field log. The tablet will not allow you to go to next question if member entered in not two digits. Enter ‘98’ if unknown’ |
| *LHHNO* | Household number (For Lilongwe) |  |  | Household number (For Lilongwe) |  | Household number (For Lilongwe) |  | Record household number for *Lilongwe site* only. This will be re-entered for correctness. The tablet will prompt you to re-enter if household numbers do not match. |
| *NATID* | National identification number |  |  | National identification number |  | National identification number |  | Record the participant’s national identification number. Ask the participant to show you their national ID and record the National ID number.  Interviewer’s hint:  If never registered, record 96  If lost/destroyed, record 97  If registered but not available, record 98 |
| *NOTE_SNAME* | Participant’s name (first name (s), surname) |  |  | Participant’s name (first name (s), surname) |  | Participant’s name (first name (s), surname) |  | Instruction: for Karonga, please write initials only. |
| *PNAMES* | Surname |  |  | Surname |  | Surname |  | Write family surname (reassure the participant on the confidential  nature of this information and that this is only needed for follow up). |
| *PHONE* | Phone number (s)? |  |  | Nambala za foni? |  | Nambala za foni? |  | Record participants’ phone number(s). The computer will prompt you to correctly enter number if phone number entered is invalid.  Interviewer’s hint:  Record 999 if phone number not available. |
| *SEX* | Sex | 0  1 | Male  Female | Mwamuna/Mkazi | Mwamuna  Mkazi | Mwanalume/mwanakazi | Mwanalume  Mwanakazi | Select Male / Female as observed |
| *GDOB* | Is date of birth known? | 1  2  4  8  9 | Full date known  Day unknown  Day and month unknown  Not known at all  Missing | gDob |  | gDob |  | Ask the date of birth of the participant. Select the appropriate option. And enter date as reported. |
| *DECADEBIRTH* | Decade of birth | 3  4  5  6  7  8  9  10  11  12  13  96 | 1910-19  1920-29  1930-39  1940-49  1950-59  1960-69  1970-79  1980-89  1990-99  2000-09  2010-19  Refused to answer | Decade of birth | 1910-19  1920-29  1930-39  1940-49  1950-59  1960-69  1970-79  1980-89  1990-99  2000-09  2010-19  Refused to answer | Decade of birth | 1910-19  1920-29  1930-39  1940-49  1950-59  1960-69  1970-79  1980-89  1990-99  2000-09  2010-19  Refused to answer | For people who cannot remember exact year of birth, use the birth decade estimate |
| *AGEYEAR* | How old are you? |  |  | Muli ndi zaka zingati? |  | Muna vyaka vilinga? |  | Enter age in years. The tablet will prompt you to enter correct years if date of birth and age in years do not match. |
| ***GFAM*** | **YOUR FAMILY AND YOU** |  |  | **YOUR FAMILY AND YOU** |  | **YOUR FAMILY AND YOU** |  |  |
| *MARITAL* | What is your current marital status? | 0  1  2  3  6 | Never married  Married  Divorced/separated  Widowed  Refused to answer | Kodi ndinu wokwatira/wokwatiwa? | Sindinakhalepo pa banja  Ndili pa banja  Banja linatha/tinasiyana  Ndinafeledwa  Refused to answer | Kasi muli mkawilo wuli pa nkhani ya vyanthengwa panyengo yasono? | Nindatengwepo/kutolapo  Nili pa nthengwa  Nthengwa ili kumala/Mphatukano  Nili chokolo  Refused to answer | Record as responded. |
| *GSPOUSE* | Name of spouse |  |  | Chonde pelekani maina a akazi/amuna anu |  | Mazina gha awo muli nawo pa nthwengwa |  | Name of spouse, if married. Also include spouse ID if known. Enter ‘99’ if unknown or not assigned. |
| *SPOUSFA* | A. Name of spouse First name (s) |  |  | A. Dzina la Mkazi/Mwamuna woyamba |  | A. Zina la mwanakazi/mwanalume wakwamba |  |  |
| *SPOUSSA* | Surname |  |  | Surname |  | Surname |  |  |
| *STIDSPA* | Study ID |  |  | Study ID |  | Study ID |  |  |
| *SPOUSENUM* | How many spouses do you have now? |  |  | Muli ndi akazi angati pakali pano? |  | Pala muli pa nthengwa, muli na wanakazi walinga sono? |  | This is asked for male participants. |
| *GSPOUSE1* | Name of spouse |  |  | Chonde pelekani maina a akazi anu |  | Mazina gha awo muli nawo pa nthwengwa |  | Enter Spouse name starting with the recent one. Include study ID for index spouse and ‘99’ for other spouses not in the study. |
| *SPOUSFA* | A. Name of spouse 1 First name (s) |  |  | A. Dzina la Mkazi woyamba |  | A. Zina la mwanakazi wakwamba |  | Spouse 1 |
| *SPOUSSA* | Surname |  |  | Surname |  | Surname |  |  |
| *STIDSPA* | Study ID |  |  | Study ID |  | Study ID |  |  |
| *GSPOUSE2* | Name of spouse |  |  | Chonde pelekani maina a akazi anu |  | Mazina gha awo muli nawo pa nthwengwa |  | Spouse 2 |
| *SPOUSFB* | A. Name of spouse 2 First name (s) |  |  | A. Dzina la Mkazi wachiwiri |  | A. Zina la mwanakazi wachiwiri |  |  |
| *SPOUSSB* | Surname |  |  | Surname |  | Surname |  |  |
| *STIDSPB* | Study ID |  |  | Study ID |  | Study ID |  |  |
| *GSPOUSE3* | Name of spouse |  |  | Chonde pelekani maina a akazi anu |  | Mazina gha awo muli nawo pa nthwengwa |  | Spouse 3 |
| *SPOUSFC* | A. Name of spouse 3 First name (s) |  |  | A. Dzina la Mkazi wachitatu |  | A. Zina la mwanakazi wachitatu |  |  |
| *SPOUSSC* | Surname |  |  | Surname |  | Surname |  |  |
| *STIDSPC* | Study ID |  |  | Study ID |  | Study ID |  |  |
| *GSPOUSE4* | Name of spouse |  |  | Chonde pelekani maina a akazi anu |  | Mazina gha awo muli nawo pa nthwengwa |  | Spouse 4 |
| *SPOUSFD* | A. Name of spouse 4 First name (s) |  |  | A. Dzina la Mkazi wachinayi |  | A. Zina la mwanakazi wachinayi |  |  |
| *SPOUSSD* | Surname |  |  | Surname |  | Surname |  |  |
| *STIDSPD* | Study ID |  |  | Study ID |  | Study ID |  |  |
| *ETHLIST* | Ethnicity |  |  | Ethnicity |  | Ethnicity |  |  |
| *ETHNIC* | What is your ethnic background? | 1  2  3  4  5  6  7  96 | Chewa  Tumbuka  Ngoni  Yao  Lomwe  Ngonde  Other  Refused to answer | Kodi ndinu a mtundu uti? | Chewa  Tumbuka  Ngoni  Yao  Lomwe  Ngonde  Other  Refused to answer | Kasi muli mutundu wuli? | Chewa  Tumbuka  Ngoni  Yao  Lomwe  Ngonde  Other  Refused to answer | Enter ethnic group to which participant belongs to. |
| *ETHNICOTH* | Please specify |  |  | Ngati wina, chonde tchulani |  | Pala mutundu ngunyakhe, phalani mutundu winu |  | Specify if other. |
| *SCHLEVER* | Have you ever been to school? | 0  1  2  6 | Never  Currently at school  Been to school in past  Refused to answer | Kodi muanapitako ku sukulu? | Ayi sindidapiteko  Ndikadapita kusulu  Ndidapitako kusukulu  Refused to answer | Kasi muli kusambilapo sukulu? | Yayi ndindaluteko  Ndichali pa sukulu  Ndili kusambirapo  Refused to answer | Record as responded. |
| *SCHLTYPE* | What is the highest level of education you attended? | 0  1  2  3  6 | None  Primary  Secondary  Tertiary  Refused to answer | Kodi munalekeza pulaimale, sekondale kapena kupitilira apo? | None  Primary  Secondary  Tertiary  Refused to answer | Sukulu muli kusambira kulekezya kalasi wuli? | None  Primary  Secondary  Tertiary  Refused to answer | Record as responded. |
| *SCHLSTD* | At school, what Form/Standard did you complete? | 1  2  3  4  5  6  7  8  9  10  11  12  98  96 | Standard 1  Standard 2  Standard 3  Standard 4  Standard 5  Standard 6  Standard 7  Standard 8  Form 1  Form 2  Form 3  Form 4  Unknown  Refused to answer | Kusukulu, kodi munamaliza folomu/sitandade yanji? | Standard 1  Standard 2  Standard 3  Standard 4  Standard 5  Standard 6  Standard 7  Standard 8  Form 1  Form 2  Form 3  Form 4  Unknown  Refused to answer | Kalasi ya primary panji ya sekondale iyo mukamalizga? | Standard 1  Standard 2  Standard 3  Standard 4  Standard 5  Standard 6  Standard 7  Standard 8  Form 1  Form 2  Form 3  Form 4  Unknown  Refused to answer | Record as responded, |
| *EDQUAL* | What is the highest educational qualification acquired? | 0  1  2  3  4  5  6  7  8  9  96 | None  PSLC  JCE  MSCE/GSCE  A-levels  University Certificate  University Diploma  University Degree  Post-Graduate Degree  Other  Refused to answer | Kodi satifiketi yaikulu yasukulu yomwe muli nayo ndi iti? | None  PSLC  JCE  MSCE/GSCE  A-levels  University Certificate  University Diploma  University Degree  Post-Graduate Degree  Other  Refused to answer | Kasi satifiketi yinu yikulu ni vichi? | None  PSLC  JCE  MSCE/GSCE  A-levels  University Certificate  University Diploma  University Degree  Post-Graduate Degree  Other  Refused to answer | This refers to the highest qualification/school certificate the participant ACQUIRED (i.e., passed the final examination at that level). For example, a person attended a few months of the first year of secondary school but did not complete the year, record “PSLC”. Circle appropriate response. The question is intended to obtain the information on which CERTIFICATE the person has. |
| *FSCHL* | Did your father go to school? | 1  0  8  6 | Yes  No  Don't Know  Refused to answer | Kodi bambo anu anapitako ku sukulu? | Eya  Ayi  Sindikudziwa  Refused to answer | Kasi wa dada winu wali kusambirapo? | Enya  Yayi  Nkhumanya yayi  Refused to answer | Record the appropriate response regardless of whether the father is alive or not. |
| *FSCHLHGHSTLVL* | What was the highest level he attended? | 0  1  2  3  4  8  6 | None  Primary  Secondary  Tertiary  Higher  Unknown  Refused to answer | Kodi analekeza pulaimale, sekondale kapena kupitilira apo? | None  Primary  Secondary  Tertiary  Higher  Unknown  Refused to answer | Kasi bakalekezgela ku primary, secondary,panji kujumphizga apo? | None  Primary  Secondary  Tertiary  Higher  Unknown  Refused to answer | This seeks to understand the level of school the participant’s father ATTENDED (Regardless of whether that level was completed). |
| *MCHL* | Did your mother go to school? | 1  0  8  6 | Yes  No  Don't Know  Refused to answer | Kodi mai anu anapitako ku sukulu ? | Eya  Ayi  Sindikudziwa  Refused to answer | Kasi wa mama winu walikusambirapo? | Enya  Yayi  Nkhumanya yayi  Refused to answer | Record the appropriate response regardless of whether the mother is alive or not. |
| *MSCHLHGHSTLVL* | What was the highest level she attended? | 0  1  2  3  4  8  6 | None  Primary  Secondary  Tertiary  Higher  Unknown  Refused to answer | Kodi analekeza pulaimale, sekondale kapena kupitilira apo | None  Primary  Secondary  Tertiary  Higher  Unknown  Refused to answer | Kasi bakalekezgela ku primary, secondary, panji kujumphizga apo? | None  Primary  Secondary  Tertiary  Higher  Unknown  Refused to answer | This seeks to understand the level of school the participant’s father ATTENDED (Regardless of whether that level was completed). |
| *EMPTYP* | What type of employment do you have? | 0  1  2  3  4  6 | Not employed  Full-time student  Piecework/irregular wage  Self-employed  Regular monthly or weekly wage/salary  Refused to answer | Kodi mumagwira ntchito yanji yomwe imakupezetsani ndalama pa moyo wanu wa tsiku ndi tsiku? | Not employed  Full-time student  Piecework/irregular wage  Self-employed  Regular monthly or weekly wage/salary  Refused to answer | Kasi mukugwira ntchito ya mtundu wuli iyo mukusangilapo ndalama pa umoyo winu wa zuba na zuba? | Not employed  Full-time student  Piecework/irregular wage  Self-employed  Regular monthly or weekly wage/salary  Refused to answer | Record as responded. |
| *OCC* | What is your main occupation? | 0  12  13  14  25  31  32  34  35  36  43  44  53  54  55  56  57  58  98  95  96 | No occupation  Teachers  Health professionals  Clergy  Commercial farmer  Desk, shop or official outdoor job  Small trader/store  Bartender, waiter  Sales assistant  Army/police  Crafts and related manual skills  Driver/assistant driver  Subsistence farmer  Herdsman  Fisherman  Labourer  Security guard/watchman  Domestic worker  Unknown  Other  Refused to answer | Kodi mumachita chiyani pa moyo wanu watsiku ndi tsiku? | No occupation  Teachers  Health professionals  Clergy  Commercial farmer  Desk, shop or official outdoor jobs  Small trader/store  Bartender, waiter  Sales assistant  Army/police  Crafts and related manual skills  Driver/assistant driver  Subsistence farmer  Herdsman  Fisherman  Labourer  Security guard/watchman  Domestic worker  Unknown  Other  Refused to answer | Kasi mukuchita vichi pa umoyo winu wa zuba na zuba? | No occupation  Teachers  Health professionals  Clergy  Commercial farmer  Desk, shop or official outdoor jobs  Small trader/store  Bartender, waiter  Sales assistant  Army/police  Crafts and related manual skills  Driver/assistant driver  Subsistence farmer  Herdsman  Fisherman  Labourer  Security guard/watchman  Domestic worker  Unknown  Other  Refused to answer | Record as responded. |
| *OTHEROCC* | If others, specify |  |  | Ngati pali zina, chonde nenani |  | Pala ni vinyakhe, niphalirani |  | Specify if other. |
| *IND* | In which industry do you work? | 0  11  12  23  30  48  51  54  61  62  63  64  65  73  74  75  95  96 | No sub-category available  Agriculture & forestry  Fishing  Food/Drink processing & sales  Mining, manufacturing, construction & energy  Transport, electricity, water, Gas, Telephone  Petty, retail, and wholesale  Financial  Health  Personal & Domestic  Government, security, public administration & defence  Education and pastoral  Information, entertainment, arts, and recreation  Real estate  Professional, scientific, and technical services  Administration & support services activities  Other  Refused to answer | Mumagwira ntchito yokhudzana ndichani? | No sub-category available  Agriculture & forestry  Fishing  Food/Drink processing & sales  Mining, manufacturing, construction & energy  Transport, electricity, water, Gas, Telephone  Petty, retail, and wholesale  Financial  Health  Personal & Domestic  Government, security, public administration & defence  Education and pastoral  Information, entertainment, arts, and recreation  Real estate  Professional, scientific, and technical services  Administration & support services activities  Other  Refused to answer | Mukugwira ntchito yakukhwaskana na vichi? | No sub-category available  Agriculture & forestry  Fishing  Food/Drink processing & sales  Mining, manufacturing, construction & energy  Transport, electricity, water, Gas, Telephone  Petty, retail, and wholesale  Financial  Health  Personal & Domestic  Government, security, public administration & defence  Education and pastoral  Information, entertainment, arts, and recreation  Real estate  Professional, scientific, and technical services  Administration & support services activities  Other  Refused to answer | Record as responded. |
| *OTHERIND* | If other, specify |  |  | Ngati pali zina, chonde nenani |  | Pala ni vinyakhe, niphalirani |  | Specify if other industry. |
| *WORKTYPE* | Do you work full-time, part-time, or seasonally? | 0  1  2  3  96 | Not applicable  Full time  Part-time  Seasonal  Refused to answer | Kodi ntchitoyi mumayigwira nthawi zones (full-time), ya nthawi zina/mwa apo ndi apo (part-time) kapena imayendera nyengo (seasonally)? | Not applicable  Full time  Part-time  Seasonal  Refused to answer | Kasi mukugwira ntchito nyengo zose (fulltime), nyengo zinyake (part-time) panji mwa pa nyengo (seasonally)? | Not applicable  Full time  Part-time  Seasonal  Refused to answer | Record as responded. |
| *MOUNCOM* | What was your personal income last month? | 1  2  3  4  5  8  6 | MK0.00-MK49 999.99.  MK50 000.00-MK99 999.99.  MK100 000.00- MK199 999.99.  MK200 000-MK499 999.99.  MK500 000 and above  Unknown  Refused to answer | Kodi munapeza ndalama zingati mwezi watha? | MK0.00-MK49 999.99.  MK50 000.00-MK99 999.99.  MK100 000.00- MK199 999.99.  MK200 000-MK499 999.99.  MK500 000 and above  Unknown  Refused to answer | Mwezi wamala mukasanga ndalama zilinga? | MK0.00-MK49 999.99.  MK50 000.00-MK99 999.99.  MK100 000.00- MK199 999.99.  MK200 000-MK499 999.99.  MK500 000 and above  Unknown  Refused to answer | Record as responded. |
| ***GMAT*** | **MATERNITY** |  |  | **MATERNITY** |  | **MATERNITY** |  |  |
| *PREG_INTRO* | Now I am going to ask you some questions about pregnancies and childbirth. First I will take photos of pages from your health passport. |  |  | Tsopano ndikufunsani mafunso ena okhudzana ndi mimba komanso kubereka. Choyamba ndijambula zithunzi za mubukhu lanu lachipatala |  | Sono nimufumbeninge mafumbo yakukhwaskana navya nthumbo na kubaba. Chakwamba nijambulenge vithuzi vya mubuku linu lachipatala. |  | Read note exactly as written. Ask participants to show you their antenatal health passport(s). |
| *PHOTO_NOTE* | Instruction to fieldworker: Photograph Antenatal Care Pages from Maternal Health Passport. Ask if woman has any previous health passports containing details of pregnancies/deliveries. If yes, photograph those records. |  |  | Instruction to fieldworker: Photograph Antenatal Care Pages from Maternal Health Passport. Ask if woman has any previous health passports containing details of pregnancies/deliveries. If yes, photograph those records. |  | Instruction to fieldworker: Photograph Antenatal Care Pages from Maternal Health Passport. Ask if woman has any previous health passports containing details of pregnancies/deliveries. If yes, photograph those records. |  | Take a picture of all antenatal pages (middle pages) in the passport. |
| *RPHOTOMAT* | Photo of record |  |  | Photo of record |  | Photo of record |  |  |
| *RECPHOTOMAT* | Take photo of record |  |  | Take photo of record |  | Take photo of record |  |  |
| *PHOTOTAKENMAT* | Was photo/s taken? | 1  2  3 | Photo/s taken  Photo/s not taken as no Health Passport  Photo/s not taken for other reason | Was photo/s taken? | Photo/s taken  Photo/s not taken as no Health Passport  Photo/s not taken for other reason | Was photo/s taken? | Photo/s taken  Photo/s not taken as no Health Passport  Photo/s not taken for other reason | Record as responded. |
| *PHOTOTAKENMATOTH* | Specify… |  |  | Specify… |  | Specify… |  | Specify if photo was not taken for any other reason apart from no health passport |
| *PREGNUM* | How many pregnancies have you had before this one, including those that ended in miscarriage or stillbirth? |  |  | Kodi munatengapo mimba zingati m'mbuyomu kupatulako iyiyi, kuphatikizapo zomwe munapititsa padera kapena kubereka mwana wakufa? |  | Kasi mulikutolapo nthumbo zilinga pambele mundatole nthumbo iyi, kusazgilapo na nthumbo izo zikanangika panyake mukababa mwana wakufwa? |  | Record the number of pregnancies the woman has had, including miscarriages and still births. |
| *PREG_NOTE* | I would like to ask you about each of your pregnancies, including those that ended in miscarriage or stillbirth. |  |  | Ndikufuna ndikufunseni mafunso pa mimba ina iliyonse mwakhala mukutenga kuphatikizapo ndi zomwe munapitisa padela kapena kubereka mwana wakufa. |  | Nkhukhumba kumufumbani za nthumbo yiliyose iyo mukatolapo, kusazgilapo na nthumbo izo zikanangika panyake mukababa mwana wakufwa. |  | Read note as written. These questions ask about each pregnancy as reported above. |
| *PREGNUM_NOTE* | The following questions are about pregnancy ${preggroup}: |  |  | Mafunso otsatirawa ndiokhudzana ndi mimba ${preggroup}: |  | Mafumbo aya yalimusi umu yakukhwaskana na nthumbo yinu ${preggroup}: |  | This will be read for |
| *AGEPREG* | How old were you when you had pregnancy ${preggroup}? |  |  | Munali ndi zaka zingati pamene munatenga mimba ${preggroup}? |  | Mukaŵa na vyaka vilinga apo mukapoka nthumbo ya ${preggroup} ? |  | Record age in years. |
| *PREGOUTCOME* | What was the outcome of the pregnancy? | 1  2  3  4  5  6 | Live birth  Miscarriage (loss of fetus before 28 weeks gestation)  Stillbirth (baby born dead after 28 weeks gestation)  Multiple birth with both live and stillborn babies  Induced abortion  Refused to answer | Kodi zotsatira za mimba zinali bwanji? | Ndinabeleka mwana wamoyo  Mimba inachoka yokha miyezi seveni isanakwane.  Nthayo (ndinabeleka mwana wakufa kale mimba itadusa miyezi seven)  Ndinabeleka mapasa mwana wina anabadwa wamoyo ndipo wina anabadwa wakufa  Mimba inachotsedwa  Refused to answer | Wumalilo wa nthumbo yinu wukawa wakuti wuli? | Mwana wakababika wamoyo  Nthumbo yikafuma yekha (yikafuma pambele yindakwane miyezi seveni)  Kubaba mwana wakufwa kale (kubaba mwana wakufwa miyezi seven yajumpha kale)  Nkhababa mileza, mwana munyakhe wakababika wamoyo ndipo munyakhe wakababika wakufwa  Kufuma kwa nthumbo mwakujipangiska  Refused to answer | Record as responded. |
| *GESTPREGOUTC_NOTE* | At how many months gestation did this happen? |  |  | Inachoka iri ndi miyezi ingati? |  | Yikafuma pa miyezi ilinga? |  | Record in months, if miscarriage. |
| *MNTHSGEST* | Months |  |  | Months |  | Months |  |  |
| *NOTE_DOB* | Is the date of delivery known? | 1  2  4  8  9 | Full date known  Day unknown  Day and month unknown  Not known at all  Missing | Tsiku lobereka? |  | Dazi la kubabila? |  | Record date of delivery if not a miscarriage. Date is recorded based on response. The tablet will prompt you to re-enter date if date entered is invalid. |
| *DATEDELIVDD* | Day |  |  | Day |  | Day |  |  |
| *DATEDELIVMM* | Month |  |  | Month |  | Month |  |  |
| *DATEDELIVYY* | Year |  |  | Year |  | Year |  |  |
| *GESTDELIV* | What was the gestation at delivery? |  |  | Kodi mimba inali ndi miyezi ingati pamene mumabereka? |  | Kasi nthumbo yikaŵa na miyezi yilinga apo mukababanga? |  | Record in months. Record ‘98’ if unknown. |
| *GESTATDELIVMNTHS* | Months |  |  | Months |  | Months |  |  |
| *GESTATDELIVSLCT* | If gestation not known, was it early, at term, or post term? | 1  2  3  8  6 | Early  At term  Post term  Don't Know  Refused to answer | If gestation not known, was it early, at term, or post term? | Ndinabereka masiku asanakwane  Ndinabereka masiku atakwana  Ndinabereka masiku atapitilira  Sindikudziwa  Refused to answer | If gestation not known, was it early, at term, or post term? | Nkhababa mazuba yakubabila yandakwane yayi  Nkhababa munyengo yakhe  Nkhababa mumazuba yakubabikila yajumpha  Nkhumanya yayi  Refused to answer | Record as responded if gestation age unknown. |
| *DELIVMETHD* | What was the method of delivery? | 1  2  3  4  5  6 | SVD  Breech  Instrumental  Caesarean not in labour  Caesarean in labour  Refused to answer | Mwana anabadwa munjira yanji? | Anabadwa kuzela njira ya bambobwinobwino  Anatsogoza matako kapena miyendo  Anagwiritsira ntchito zitsulo  Ndinaberekera opereshoni mimba isanawawe  Ndinaberekera opaleshoni mimba itawawa  Refused to answer | Mwana wakababika nthowa wuli? | Nkhababa kwizila munthowa ya wanakazi (makola waka)  Mwana wakandangizga vigugu panji malundi)  Wakagwiliska ntchito visulu  Nkhababila chimayi, pambele nthumbo yindauke  Nkhababila chimayi nthumbo ikawa yauka  Refused to answer | Record as responded. |
| *BIRTHPL* | Where did you give birth? | 1  2  3  4  5  6  7  96 | Primary Health Centre  Rural Hospital  Referral hospital  In transit  At home with TBA  At home without TBA  Other  Refused to answer | Munaberekera kuti? | Chipatala chaching’ono (Primary Health Centre)  Chipatala chachikuluko (Rural hospital)  Chipatala chachikulu chotumizirako anthu (Referral hospital)  Ndinabelekera pa njila  Panyumba koma ndi mzamba  Panyumba koma popanda mzamba  Kwina  Refused to answer | Mukababila kochi? | Chipatala chichoko (Primary Health Centre)  Chipatala chachikuluko (Rural hospital)  Chipatala chikulu chakutumilako wanthu (Referral hospital)  Nkhababila munthowa  Nkhababila panyumba na mzamba  Nkhababila panyumba kwambula mzamba  Kunyakhe  Refused to answer | Record as responded. |
| *BIRTHPLOTH* | If other, specify |  |  | Ngati pali zina, chonde nenani |  | Pala ni vinyakhe, niphalirani |  | Specify if other. |
| *COMPLDURPREG* | Did you have complications during this pregnancy, delivery or afterwards? Complications include heavy bleeding, pre-eclampsia, gestational diabetes, or anything that needed an operation, medical treatment or extra time in hospital. | 1  0  8  6 | Yes  No  Don't Know  Refused to answer | Kodi munali ndi zobvuta pamene munali oyembekezera, pobereka kapena mutabereka? Zobvuta monga kutaya magazi kwambiri, kuthamanaga magazi, matenda a shuga kapena vuto lirilonse lofunika opareshoni, mankhwala kapena kukhalitsa nthawi yayitali mchipatala. | Eya  Ayi  Sindikudziwa  Refused to answer | Kasi mukawa namawunonono apo mukaŵa na nthumbo iyi, pakubaba panji mwati mwababa? Maunonono nge kuthika ndopa chomene, kutchimbila kwa ndopa, nthenda ya shuga panji unonono uliwose wakukhumbika opereshoni, wovwili wa mankhwala panji kukhaliska nyengo yitali muchipatala. | Enya  Yayi  Nkhumanya yayi  Refused to answer | Record as responded. |
| *COMPL_NOTE* | Which of the following complications did you have during this pregnancy, delivery or afterwards? |  |  | Kodi munakumana ndi zobvuta zotsatilazi pa nthawi ya mimba, mutabeleka kapena mutangochila? |  | Kasi mukakumana namaunono yakulondezga aya pa nyengo ya nthumbo, pakuchila panji mwati mwachila? |  | If yes or does not know any complications, read out the complications below. |
| *COMPL_LABELS* |  | 1  0  8  6 | Yes  No  Don't Know  Refused to answer |  | Eya  Ayi  Sindikudziwa  Refused to answer |  | Enya  Yayi  Nkhumanya yayi  Refused to answer | Record as responded. |
| *COMPBLEEDBEF* | Heavy bleeding before birth | 1  0  8  6 | Yes  No  Don't Know  Refused to answer | Ndimataya magazi mochuluka ndisanabereke. | Eya  Ayi  Sindikudziwa  Refused to answer | Nkhathikanga ndopa chomene pambele ndindababe… | Enya  Yayi  Nkhumanya yayi  Refused to answer | Record as responded. |
| *COMPBLEEDAFT* | Heavy bleeding after birth | 1  0  8  6 | Yes  No  Don't Know  Refused to answer | Ndimataya magazi mochuluka nditabereka. | Eya  Ayi  Sindikudziwa  Refused to answer | Nkhathikanga ndopa chomene ndati ndababa. | Enya  Yayi  Nkhumanya yayi  Refused to answer | Record as responded. |
| *COMPTEAR* | Severe vaginal tear after childbirth | 1  0  8  6 | Yes  No  Don't Know  Refused to answer | Njila yodusamo mwana pobeleka yinavulazidwa kwambili nditabeleka… | Eya  Ayi  Sindikudziwa  Refused to answer | Nthowa ya kubabila yikakeluka chomene ndati ndababa… | Enya  Yayi  Nkhumanya yayi  Refused to answer | Record as responded. |
| *COMPFISTULA* | Fistula | 1  0  8  6 | Yes  No  Don't Know  Refused to answer | Fisitula (Kubowoka pakati panjila yochilira ndi njila yachimbudzi-kuchucha mikodzo kapena chimbuzi kudzera munjira yo chilira). | Eya  Ayi  Sindikudziwa  Refused to answer | Fisitula (kudoloka pakati pa nthowa ya kubabila nakuchigugu kuthika matuzi panji chimbuzi). | Enya  Yayi  Nkhumanya yayi  Refused to answer | Record as responded. |
| *COMPFISTULAREP* | HAS THE FISTULA BEEN REPAIRED? | 1  0  8  6 | Yes  No  Don't Know  Refused to answer | KODI FISITULAYI ANAPANGA OPARESHONI? | Eya  Ayi  Sindikudziwa  Refused to answer | KASI FISITULA IYI BAKAPANGA OPARESHONI? | Enya  Yayi  Nkhumanya yayi  Refused to answer | If yes to above, record as responded. |
| *COMPDIAB* | Diabetes in pregnancy | 1  0  8  6 | Yes  No  Don't Know  Refused to answer | Ndinali ndi bvuto la nthenda ya shuga pamene ndinali ndi mimba. | Eya  Ayi  Sindikudziwa  Refused to answer | Nkhaŵa na suzgo la shuga pa nyengo ya nthumbo. | Enya  Yayi  Nkhumanya yayi  Refused to answer | Record as responded. |
| *COMPBP* | Pre-eclampsia (high blood pressure) | 1  0  8  6 | Yes  No  Don't Know  Refused to answer | Ndinali ndi bvuto la kuthamanga kwambiri kwa magazi pamene ndinali ndi mimba. | Eya  Ayi  Sindikudziwa  Refused to answer | Nkhaŵa na suzgo la kutchimbila chomene kwa ndopa pa nyengo ya nthumbo. | Enya  Yayi  Nkhumanya yayi  Refused to answer | Record as responded. |
| *COMPECLAMP* | Eclampsia (fits due to high blood pressure) | 1  0  8  6 | Yes  No  Don't Know  Refused to answer | Ndimakomoka chifukwa chakuthamanga kwambiri kwamagazi. | Eya  Ayi  Sindikudziwa  Refused to answer | Kunyutuka chifukwa chakutchimbila chomene kwa ndopa. | Enya  Yayi  Nkhumanya yayi  Refused to answer | Record as responded. |
| *COMPINF* | Infection during or after pregnancy | 1  0  8  6 | Yes  No  Don't Know  Refused to answer | Ndinadwala( ndikamva nthupi kutentha ndipo adotolo adandipasa makhwala) pamene ndinali ndipakati/nditangochira | Eya  Ayi  Sindikudziwa  Refused to answer | Nkhalwala( thupi kotcha ndipo adokotala wakanipasa makhwala) panyengo ya nthumbo panji nati nachila. | Enya  Yayi  Nkhumanya yayi  Refused to answer | Record as responded. |
| *COMPPROM* | Waters broke more than 1 week before delivery | 1  0  8  6 | Yes  No  Don't Know  Refused to answer | Ndinayamba kutaya madzi kutatsara sabata imodzi ndisanachire | Eya  Ayi  Sindikudziwa  Refused to answer | Nkhayamba kuthika maji sabata yimoza pambele nindachile | Enya  Yayi  Nkhumanya yayi  Refused to answer | Record as responded. |
| *COMPLOTHSPEC* | Other, specify |  |  | Zina, longosolani |  | Vinyakhe, longosolani |  | Specify if other. |
| *MULTIPLEPREG* | How many babies resulted from this pregnancy? | 1  2  3  6 | One  Two  Three  Refused to answer | Kodi munabereka ana angati pa mimba imeneyi? | Mmodzi  Awiri  Atatu  Refused to answer | Kasi mukababa bana balinga nthumbo iyi? | Yumoza  Bawiri  Batatu  Refused to answer | Record as responded, if pregnancy was not a miscarriage. |
| *NUMSTLBRN* | How many babies were stillborn? | 1  2  6 | One  Two  Refused to answer | Kodi ndi ana angati omwe anabadwa atafa kale? | Mmodzi  Awiri  Refused to answer | Kasi ni bana balinga awo wakababika bakufwa kale? | Yumoza  Bawiri  Refused to answer | Record as responded. |
| *BRNALVE_NOTE* | The following questions are about the baby (babies) who were born alive from this pregnancy |  |  | Mafunso otsatirawa ndiokhudzana ndi mwana/ana omwe anabadwa amoyo pamimba iyi |  | Mafumbo yakulondezga aya yakukhwaskana na mwana/bana abo bakababika bamoyo panthumbo iyi |  | This question is for live babies born from the pregnancy being inquired about. |
| *SINGSEX* | What sex was the child? | 0  1 | Male  Female | Mwana anali wam’muna kapena wam’kazi? | Mwamuna  Mkazi | Mwana akaŵa msepuka panji msungwana? | Mwanalume  Mwanakazi | Record as responded. |
| *SINGALIVE* | Is the child still alive? | 1  0  8  6 | Yes  No  Don't Know  Refused to answer | Kodi mwana akadali ndi moyo? | Eya  Ayi  Sindikudziwa  Refused to answer | Kasi mwana wachali wamoyo? | Enya  Yayi  Nkhumanya yayi  Refused to answer | Record as responded. |
| *SINGAGEATDEATH* | How old was the child when he/she died? |  |  | Mwana anamwalira ali wamkulu bwanji? |  | Mwana akafwa mlala wuli? |  | If child dead, record as responded either in days, months or years. |
| *SINGAGEATDEATHDD* | Days |  |  | Masiku |  | Madazi |  |  |
| *SINGAGEATDEATHMM* | Months |  |  | Mwiyezi |  | Miyezi |  |  |
| *SINGAGEATDEATHYY* | Years |  |  | Zaka |  | Vyaka |  |  |
| *SINGCAUSEDEATH* | What was the cause of death? |  |  | Anamwalira ndi chiyani? |  | Icho chikapangiska kuti mwana wafwe ntchivichi? |  | Record in a few key words as described by participant. |
| *SINGDISAB* | Does/Did the child have a congenital anomaly, handicap/disability? | 1  0  8  6 | Yes  No  Don't Know  Refused to answer | Kodi anali kapena ali ndi chilema kapena ndiwolumala? | Eya  Ayi  Sindikudziwa  Refused to answer | Kasi mwana aakawa panji wali nachilema panji wakupendela? | Enya  Yayi  Nkhumanya yayi  Refused to answer | Record as responded. |
| *SINGDISABSPEC* | Specify… |  |  | Longosolani… |  | Longosolani… |  | Specify abnormality if yes. |
| *MULTIPLSEX1* | What sex was child 1? | 0  1 | Male  Female | Mwana woyamba kubadwayo anali wam’muna kapena wam’kazi? | Mwamuna  Mkazi | Mwana wakwamba kubabika akaŵa mwanalume panji mwanakazi? | Mwanalume  Mwanakazi | This is question is for first child in a multiple pregnancy. |
| *MULTIPL1LALIVE* | Is child 1 still alive? | 1  0  8  6 | Yes  No  Don't Know  Refused to answer | Mwana woyamba kubadwa, kodi akadali ndi moyo? | Eya  Ayi  Sindikudziwa  Refused to answer | Mwana wakwamba kubabika, kasi wachali wamoyo? | Enya  Yayi  Nkhumanya yayi  Refused to answer | Record as responded. |
| *MULTIPL1AGEATDEATH* | How old was child 1 when he/she died? |  |  | Mwana woyamba kubadwayo anamwalira ali wamkulu bwanji? |  | Mwana wakwamba kubabika akafwa mlala uli? |  | If child dead, record age in either days, months or years. |
| *MULTIPL1AGEATDEATHDD* | Days |  |  | Masiku |  | Madazi |  |  |
| *MULTIPL1AGEATDEATHMM* | Months |  |  | Mwiyezi |  | Miyezi |  |  |
| *MULTIPL1AGEATDEATHYY* | Years |  |  | Zaka |  | Vyaka |  |  |
| *MULTIPL1CAUSEDEATH* | What was the cause of death? |  |  | Anamwalira ndi chiyani? |  | Icho chikapangiska kuti mwana wafwe ntchivichi? |  | Record in a few key words as described by participant. |
| *MULTIPL1DISAB* | Does/Did child 1 have a congenital anomaly, handicap/disability? | 1  0  8  6 | Yes  No  Don't Know  Refused to answer | Kodi anali kapena ali ndi chilema kapena ndiwolumala? | Eya  Ayi  Sindikudziwa  Refused to answer | Kasi mwana aakawa panji wali nachilema panji wakupendela? | Enya  Yayi  Nkhumanya yayi  Refused to answer | Record as responded. |
| *MULTIPL1DISABSPEC* | Specify… |  |  | Longosolani… |  | Longosolani… |  | Specify abnormality if yes. |
| *MULTIPLSEX2* | What sex was child 2? | 0  1 | Male  Female | Mwana wachiwiri kubadwayo anali wam’muna kapena wam’kazi? | Mwamuna  Mkazi | Mwana wachibili kubabika akaŵa mwanalume panji mwanakazi? | Mwanalume  Mwanakazi | This is question is for second child in a multiple pregnancy. |
| *MULTIPL2LALIVE* | Is child 2 still alive? | 1  0  8  6 | Yes  No  Don't Know  Refused to answer | Kodi mwana wachiwiri kubadwa, akadali ndi moyo? | Eya  Ayi  Sindikudziwa  Refused to answer | Kasi mwana wachibili kubabika, wachali wamoyo? | Enya  Yayi  Nkhumanya yayi  Refused to answer | Record as responded. |
| *MULTIPL2AGEATDEATH* | How old was child 2 when he/she died? |  |  | Mwana wachiwiri kubadwayo anamwalira ali wamkulu bwanji? |  | Mwana wachibili kubabika akafwa mlala uli? |  | If child dead, record age in either days, months or years. |
| *MULTIPL2AGEATDEATHDD* | Days |  |  | Masiku |  | Madazi |  |  |
| *MULTIPL2AGEATDEATHMM* | Months |  |  | Mwiyezi |  | Miyezi |  |  |
| *MULTIPL2AGEATDEATHYY* | Years |  |  | Zaka |  | Vyaka |  |  |
| *MULTIPL2CAUSEDEATH* | What was the cause of death of child 2? |  |  | Anamwalira ndi chiyani? |  | Icho chikapangiska kuti mwana wafwe nchivichi? |  | Record in a few key words as described by participant. |
| *MULTIPL2DISAB* | Does/Did child 2 have a congenital anomaly, handicap/disability? | 1  0  8  6 | Yes  No  Don't Know  Refused to answer | Kodi anali kapena ali ndi chilema kapena ndiwolumala? | Eya  Ayi  Sindikudziwa  Refused to answer | Kasi mwana aakawa panji wali nachilema panji wakupendela? | Enya  Yayi  Nkhumanya yayi  Refused to answer | Record as responded. |
| *MULTIPL2DISABSPEC* | Specify… |  |  | Longosolani… |  | Longosolani… |  | Specify abnormality if yes. |
| *MULTIPLSEX3* | What sex was child 3? | 0  1 | Male  Female | Mwana wachitatu kubadwayo anali wam’muna kapena wam’kazi? | Mwamuna  Mkazi | Mwana wachitatu kubabika akaŵa mwanalume panji mwanakazi? | Mwanalume  Mwanakazi | This is question is for third child in a multiple pregnancy. |
| *MULTIPL3LALIVE* | Is child 3 still alive? | 1  0  8  6 | Yes  No  Don't Know  Refused to answer | Kodi mwana wachitatu kubadwa, akadali ndi moyo? | Eya  Ayi  Sindikudziwa  Refused to answer | Kasi mwana wachitatu kubabika, wachali wamoyo? | Enya  Yayi  Nkhumanya yayi  Refused to answer | Record as responded. |
| *MULTIPL3AGEATDEATH* | How old was child 3 when he/she died? |  |  | Mwana wachitatu kubadwayo anamwalira ali wamkulu bwanji? |  | Mwana wachitatu kubabika akafwa mlala uli? |  | If child dead, record age in either days, months or years. |
| *MULTIPL3AGEATDEATHDD* | Days |  |  | Masiku |  | Madazi |  |  |
| *MULTIPL3AGEATDEATHMM* | Months |  |  | Mwiyezi |  | Miyezi |  |  |
| *MULTIPL3AGEATDEATHYY* | Years |  |  | Zaka |  | Vyaka |  |  |
| *MULTIPL3CAUSEDEATH* | What was the cause of death of child 3? |  |  | Anamwalira ndi chiyani? |  | Icho chikapangiska kuti mwana wafwe nchivichi? |  | Record in a few key words as described by participant. |
| *MULTIPL3DISAB* | Does/Did child 3 have a congenital anomaly, handicap/disability? | 1  0  8  6 | Yes  No  Don't Know  Refused to answer | Kodi anali kapena ali ndi chilema kapena ndiwolumala? | Eya  Ayi  Sindikudziwa  Refused to answer | Kasi mwana aakawa panji wali nachilema panji wakupendela? | Enya  Yayi  Nkhumanya yayi  Refused to answer | Record as responded. |
| *MULTIPL3DISABSPEC* | Specify… |  |  | Longosolani… |  | Longosolani… |  | Specify abnormality if yes. |
| ***PREGINTENT*** | **PREGNANCY INTENTION/REPRODUCTIVE AUTONOMY** |  |  | **KHUMBO LAKUTENGA MIMBA/KUZIPANGIRA ZIGANIZO PA NKHANI YAUCHEMBERE** |  | **CHILATO/KHUMBILO PAKUTOLA NTHUMBO/WANANGWA UWO MUNTHU AKUŴA NAWO PA NKHANI YA KUCHEMBEZGA** |  |  |
| *PREGINTENT_NOTE* | The following questions ask about your use of contraception before you became pregnant WITH YOUR CURRENT PREGNANCY, your wish and intention to get pregnant. |  |  | Mafunso otsatirawa akufunsa momwe inu munkagwiritsira ntchito njila zakulera musanatenge mimba, chikhumbokhumbo ndi kufuna kwanu kuti mutenge mimba. |  | Mafumbo yakulondezga aya yakufumba umo imwe mukagwiliskilanga nchito nthowa za vyakulela pambele mundapoke nthumbo, khumbiro na chilato chinu pakutola nthumbo. |  | Read note to participant exactly as written. |
| *CONTRACEP* | In the month before you became pregnant which of the following was true regarding contraception? | 1  2  3  4  6 | I/we were not using contraception  I/we were using contraception, but not on every occasion  I/we always used contraception, but knew that the method had failed (i.e. broke, moved, came off, came out, not worked etc) at least once  I/we always used contraception  Refused to answer | Mumwezi wakumbuyo musanatenge mimba, kodi ndiziti mwaziganizo zotsatirazi zomwe zikufotoza chilungamo pa nkhani ya zakulela? | Ine/ife sitimagwiritsa ntchito njira ina iliyose yakulera  Ine/ife timagwiritsa ntchito njila yakulera koma osati nthawi zonse  Ine/ife nthawi zonse timagwiritsa njira yakulera koma ndinadziwa/tinadziwa kuti inakanika kugwira ntchito yake (inaphulika, inasuntha, inachoka etc)  Ine/ife nthawi zonse timagwiritsa ntchito njira yakulera  Refused to answer | Mu mwezi uwo mukawamo pambele mundapoke nthumbo kasi ndi fundo nji yakulondezgapo iyo njaunenesko mukulongosola vya nthowa zakulela? | Ine/ise tikagwiliskapo nthowa yiliyose yayi ya vyakulela  Ine/ise tikagwiliskapo nthowa ya vyakulela kweni mwanyengo zonse yayi  Ine/ise nyengo zose tikagwiliskanga nthowa ya vyakulela kweni nkhamanya kuti yikatondekapo (nge kusweka, kusunkhunyuka, kushezgeka, kuleka kugwila nchito etc.)  Ine/ise nyengo zose tikagwiliskanga nchito nthowa ya vyakulela  Refused to answer | Record as responded. |
| *CONTDEC* | Would you say that using contraception is mainly your decision, mainly your (husband's/partner's) decision, or do you both decide together? | 1  2  3  6 | Mainly respondent  Mainly husband/partner  Joint decision  Refused to answer | Kodi munganene kuti kugwiritsa ntchito njira yakulera ndi ganizo lanu, lamwamuna wanu kapena nonse awiri pamodzi? | Ganizo langa nthawi zambiri  Ganizo la mwamuna/ chibwenzi wanga nthawi zambiri  Ganizo la tonsefe awiri  Refused to answer | Kasi mungayowoya kuti kugwiliska nchito nthowa ya vyakulela ni chigamulo cha imwe mwekha, cham’fumu winu pela panji mose mwawawili pamoza? | Ntchigamulo chane nyengo zinandi  Ntchigamulo cha m’fumu/chibwezi chane nyengo zinandi  Ntchigamulo chatose wawili pamoza  Refused to answer | Record as responded. |
| *PREGWISH* | Just before you became pregnant, which of the following was true regarding your wish to get pregnant? | 1  2  3  6 | I wanted to get pregnant  I did not want to get pregnant  I had no strong feelings either way  Refused to answer | Musanatenge mimba, ndichiganizo chiti paziganizo zotsatirazi chomwe chikukamba zowona pakufuna kwanu potenga mimba? | Ndinachita kufuna kutenga mimba  Sindinafune kutenga mimba  Ndinali pakatikati  Refused to answer | Pambele mundapoke nthumbo, ndi fundo nji yakulondezgapo iyo yikuyowoya unenesko za khumbo linu ilo mukaba nalo pakupoka nthumbo? | Nkhakhumba kupoka nthumbo  Nkhakhumba yayi kupoka nthumbo  Nkhawa waka pakatikati  Refused to answer | Record as responded. |
| *PREGINTENTMEN_NOTE* | The following question asks about your wish and intention for your wife to get pregnant. |  |  | Funso lotsatirali lifunsa zokhudzana ndi chikhumbokhumbo ndi kufuna kwanu kuti akazi anu atenge mimba. |  | Fumbo lakulondezga ili likufumba vyakukhwaskana na khumbiro na chilato chinu chakuti bawoli winu bapoke nthumbo. |  | *For men only. Read note as written.* |
| *PREGWISHMEN* | Just before your wife became pregnant, which of the following was true regarding your wish for her to get pregnant? | 1  2  3  6 | I wanted her to get pregnant  I did not want her to get pregnant  I had no strong feelings either way  Refused to answer | Akazi anu asanatenge mimba, ndichiganizo chiti paziganizo zotsatirazi chomwe chikukamba zowona pakufuna kwanu kuti atenge mimba? | Ndinachita kufuna kuti atenge mimba  Sindinafune kuti atenge mimba  Ndinali pakatikati  Refused to answer | Pambele bawoli winu bandapoke nthumbo, ndi fundo nji pazakulondezgapo izi iyo yikuyowoya wunenesko wa zakhumbo linu ilo mukaba nalo kuti bapoke nthumbo? | Nkhakhumba kuti bapoke nthumbo  Nkhakhumba yayi kuti bapoke nthumbo  Nkhawa waka pakatikati  Refused to answer | Record as responded. |
| ***GMHM*** | **MEDICAL HISTORY & MEDICATION** |  |  | **MEDICAL HISTORY & MEDICATION** |  | **MEDICAL HISTORY & MEDICATION** |  |  |
| *MEDHIST_INTRO* | The following questions are about health conditions that have been diagnosed by a health professional. I will also check the information in your health passport |  |  | Mafunso otsatirawa ndiokhudza matenda omwe anakupezani nawo ogwira ntchito ya zaumoyo. Ndionatso uthenga wa mubuku lanu lakuchipatala. |  | Mafumbo yakulondezga aya ngakukhwaskana na matenda ayo wakamusangani nayo wakugwira ntchito ya vyaumoyo. Niwonengeso wanduzi wa mubuku linu lakuchipatala. |  | Read note to participant and inform them that their health passport will be needed. |
| *DIABP* | Have you ever been diagnosed with high blood pressure by a doctor or other health professional? | 1  0  8  6 | Yes  No  Don't Know  Refused to answer | Kodi munayamba mwapezedwapo ndi nthenda yothamanga magazi ndi adotolo kapena anthu ena a zachipatala? | Eya  Ayi  Sindikudziwa  Refused to answer | Kasi muli kupimikapo nawadokotala panji wa vyawumoyo kuti muli na matenda ya kuchimbila kwa ndopa? | Enya  Yayi  Nkhumanya yayi  Refused to answer | Record as responed.  Note: this question is asking for high blood pressure confirmed by a **‘Professional’** not by field interviewers in previous MEIRU surveys. The interviewer can check in the health passport to confirm any diagnoses. |
| *BPW* | Blood Pressure **for women** |  |  | Blood Pressure for women |  | Blood Pressure for women |  |  |
| *BPWHENPREG* | Did the high blood pressure only happen when you were pregnant or also at other times? | 1  2  8  6 | Only when pregnant  Also at other times  Don't know  Refused to answer | Kodi kuthamanga kwa magazi kudachitika nthawi yomwe mudali ndi mimba yokha kapena nthawi inaso? | Pamene mudali ndi mimba pokha  Nthawi inanso  Don't know  Refused to answer | Kasi kuchimbila chomene kwa ndopa kukachitika pa nyengo iyo mukaba nathumbo pera panyakhe pakabaso nyengo inyakhe? | Apo mukaba na thumbo pekha  Nyengo yinyakheso  Don't know  Refused to answer | *For women only. Record as responded.* |
| *REMDBP* | Are you on regular medication for high blood pressure? | 1  0  8  6 | Yes  No  Don't Know  Refused to answer | Kodi mukulandira mankhwala a nthenda yothamanga magazi? | Eya  Ayi  Sindikudziwa  Refused to answer | Kasi muli pamankhwala ya matenda yokutchimbila kwa ndopa? | Enya  Yayi  Nkhumanya yayi  Refused to answer | Record as responded. |
| *BP_MEDS* | *BP medication* |  |  | *BP medication* |  | *BP medication* |  |  |
| *NOTE_BPNOM* | If yes, name of medication (s) |  |  | Ngati eya, chonde tchulani dzina la mankhwalawo |  | Pala enya, chonde zunulani zina lamankhwala? |  | If on medication,select medication from list below. Interviewer can also check in the book to confirm medications. |
| *BPHCT* | A. Hydrochlorthiazide (HCT) | 1  0  8  6 | Yes  No  Don't Know  Refused to answer | A. Hydrochlorthiazide (HCT) | Eya  Ayi  Sindikudziwa  Refused to answer | A. Hydrochlorthiazide (HCT) | Enya  Yayi  Nkhumanya yayi  Refused to answer | Record as responded. |
| *BPAML* | B. Amlodipine | 1  0  8  6 | Yes  No  Don't Know  Refused to answer | B. Amlodipine | Eya  Ayi  Sindikudziwa  Refused to answer | B. Amlodipine | Enya  Yayi  Nkhumanya yayi  Refused to answer | Record as responded. |
| *BPBIF* | C. Nifedipine | 1  0  8  6 | Yes  No  Don't Know  Refused to answer | C. Nifedipine | Eya  Ayi  Sindikudziwa  Refused to answer | C. Nifedipine | Enya  Yayi  Nkhumanya yayi  Refused to answer | Record as responded. |
| *BPENA* | D. Enalopril | 1  0  8  6 | Yes  No  Don't Know  Refused to answer | D. Enalopril | Eya  Ayi  Sindikudziwa  Refused to answer | D. Enalopril | Enya  Yayi  Nkhumanya yayi  Refused to answer | Record as responded. |
| *BPCAP* | E. Captopril/Lisinopril | 1  0  8  6 | Yes  No  Don't Know  Refused to answer | E. Captopril/Lisinopril | Eya  Ayi  Sindikudziwa  Refused to answer | E. Captopril/Lisinopril | Enya  Yayi  Nkhumanya yayi  Refused to answer | Record as responded. |
| *BPATE* | F. Atenolol | 1  0  8  6 | Yes  No  Don't Know  Refused to answer | F. Atenolol | Eya  Ayi  Sindikudziwa  Refused to answer | F. Atenolol | Enya  Yayi  Nkhumanya yayi  Refused to answer | Record as responded. |
| *BPPRO* | G. Propranolol | 1  0  8  6 | Yes  No  Don't Know  Refused to answer | G. Propranolol | Eya  Ayi  Sindikudziwa  Refused to answer | G. Propranolol | Enya  Yayi  Nkhumanya yayi  Refused to answer | Record as responded. |
| *BPMDP* | H. Methyldopa/Aldomet | 1  0  8  6 | Yes  No  Don't Know  Refused to answer | H. Methyldopa/Aldomet | Eya  Ayi  Sindikudziwa  Refused to answer | H. Methyldopa/Aldomet | Enya  Yayi  Nkhumanya yayi  Refused to answer | Record as responded. |
| *OTHBP1* | I. Other, specify [1] |  |  | I. Ena, tchulani [1] |  | I. Ena, tchulani [1] |  | Specify if other. |
| *OTHBP2* | J. Other, specify [ 2] |  |  | J. Ena, tchulani [ 2] |  | J. Ena, tchulani [ 2] |  | Specify if other. |
| *OTHBP3* | K. Other, specify [3] |  |  | K. Ena, tchulani [3] |  | K. Ena, tchulani [3] |  | Specify if other. |
| *DIAB_GROUP* | *Diabetes* |  |  | *Diabetes* |  | *Diabetes* |  |  |
| *DIADIAB* | Have you been diagnosed with diabetes by a doctor or other health professional? | 1  0  8  6 | Yes  No  Don't Know  Refused to answer | Kodi munayambapo mwapezedwa ndi nthenda ya shuga ndi adotolo kapena azachipatala ena? | Eya  Ayi  Sindikudziwa  Refused to answer | Kasi muli kupimiskapo nakusangika na matenda ya shuga na badokotala panji wa vya umoyo banyakhe? | Enya  Yayi  Nkhumanya yayi  Refused to answer | Record as responed.  Note: this question is asking for diabetes pressure confirmed by a **‘Professional’** not by field interviewers in previous MEIRU surveys. The interviewer can check in the health passport to confirm any diagnoses. |
| *TYPDIAB* | What type of diabetes is it? | 1  2  8  6 | Type 1  Type 2  Don’t know  Refused to answer | Ndimtundu wanji wa matenda a shuga? | Type 1  Type 2  Don’t know  Refused to answer | Mukasangika na mtundu uli wa matenda ya shuga? | Type 1  Type 2  Don’t know  Refused to answer | If yes to above, record as appropriate. Interviewer can also check in health passport to confirm this. |
| *DIAW* | For diabetic **women only** |  |  | For diabetic **women only** |  | For diabetic **women only** |  |  |
| *DIAWHENPREG* | Did the diabetes only happen when you were pregnant or also at other times? | 1  2  8  6 | Only when pregnant  Also at other times  Don't know  Refused to answer | Kodi munadwara matenda a shunga nthawi yomwe mudali ndi mimba yokha kapena nthawi inaso? | Pamene mudali ndi mimba pokha  Nthawi inanso  Don't know  Refused to answer | Kasi mukalwara matenda ya shunga pa nyengo iyo mukaba nathumbo pera panyakhe pakabaso nyengo inyakhe? | Apo mukaba na thumbo pekha  Nyengo yinyakheso  Don't know  Refused to answer | *For women only. Record as responded.* |
| *DIAW* |  |  |  |  |  |  |  |  |
| *REMDDIA* | Are you on regular medication for diabetes? | 1  0  8  6 | Yes  No  Don't Know  Refused to answer | Kodi mukulandira mankhwala a nthenda yashuga? | Eya  Ayi  Sindikudziwa  Refused to answer | Kasi muli pa mankhwala ya nthenda ya shuga? | Enya  Yayi  Nkhumanya yayi  Refused to answer | Record as responded. |
| *DIAB_MEDS* | Diabetes medication |  |  | Diabetes medication |  | Diabetes medication |  | If on medication, select medication from list below. Interviewer can also check in the book to confirm medications. |
| *NOTE_NODIAM* | If yes, name of medication (s) |  |  | Ngati eya, chonde tchulani dzina la mankhwalawo |  | Pala enya, chonde zunulani zina lamankhwala? |  | Record as responded. |
| *DBMET* | A. Metformin | 1  0  8  6 | Yes  No  Don't Know  Refused to answer | A. Metformin | Eya  Ayi  Sindikudziwa  Refused to answer | A. Metformin | Enya  Yayi  Nkhumanya yayi  Refused to answer | Record as responded. |
| *DBGLI* | B. Glibenclamide | 1  0  8  6 | Yes  No  Don't Know  Refused to answer | B. Glibenclamide | Eya  Ayi  Sindikudziwa  Refused to answer | B. Glibenclamide | Enya  Yayi  Nkhumanya yayi  Refused to answer | Record as responded. |
| *DBLEN* | C. Lente | 1  0  8  6 | Yes  No  Don't Know  Refused to answer | C. Lente | Eya  Ayi  Sindikudziwa  Refused to answer | C. Lente | Enya  Yayi  Nkhumanya yayi  Refused to answer | Record as responded. |
| *DBADR* | D. Actrapid | 1  0  8  6 | Yes  No  Don't Know  Refused to answer | D. Actrapid | Eya  Ayi  Sindikudziwa  Refused to answer | D. Actrapid | Enya  Yayi  Nkhumanya yayi  Refused to answer | Record as responded. |
| *DBACL* | E. Acraphane | 1  0  8  6 | Yes  No  Don't Know  Refused to answer | E. Acraphane | Eya  Ayi  Sindikudziwa  Refused to answer | E. Acraphane | Enya  Yayi  Nkhumanya yayi  Refused to answer | Record as responded. |
| *DBPRO* | F. Protaphane | 1  0  8  6 | Yes  No  Don't Know  Refused to answer | F. Protaphane | Eya  Ayi  Sindikudziwa  Refused to answer | F. Protaphane | Enya  Yayi  Nkhumanya yayi  Refused to answer | Record as responded. |
| *DBNOV* | G. Novomix 30 | 1  0  8  6 | Yes  No  Don't Know  Refused to answer | G. Novomix 30 | Eya  Ayi  Sindikudziwa  Refused to answer | G. Novomix 30 | Enya  Yayi  Nkhumanya yayi  Refused to answer | Record as responded. |
| *OTHDB* | H. Other, specify [1] |  |  | H. Ena, tchulani [1] |  | H. Ena, tchulani [1] |  | Specify if other. |
| *OTHDB2* | I. Other, specify [2] |  |  | I. Ena, tchulani [2] |  | I. Ena, tchulani [2] |  | Specify if other. |
| *OTHDB3* | J. Other, specify [3] |  |  | J. Ena, tchulani [3] |  | J. Ena, tchulani [3] |  | Specify if other. |
| *ASTH* | *Asthma* |  |  | *Asthma* |  | *Asthma* |  |  |
| *ASTCK* | Have you ever been diagnosed with asthma by a doctor or other health professional? | 1  0  8  6 | Yes  No  Don't Know  Refused to answer | Kodi munayamba mwapezedwapo ndi nthenda ya mphumu ndi adotolo kapena azachipatala ena? | Eya  Ayi  Sindikudziwa  Refused to answer | Kasi muli kupimiskapo nakusangika na matenda ya asima na badokotala panji wa vya umoyo banyakhe | Enya  Yayi  Nkhumanya yayi  Refused to answer | Record as responed.  Note: this question is asking for asthma confirmed by a **‘Professional’** not by field interviewers in previous MEIRU surveys. The interviewer can check in the health passport to confirm any diagnoses. |
| *ASTHMED* | Are you on regular medication for asthma? | 1  0  8  6 | Yes  No  Don't Know  Refused to answer | Kodi mukulandira mankhwala a mphumu? | Eya  Ayi  Sindikudziwa  Refused to answer | Kasi muli pa mankhwala ya asima? | Enya  Yayi  Nkhumanya yayi  Refused to answer | Record as responded. |
| *NMASMED* | If yes, name of medication |  |  | Ngati eya, chonde tchulani dzina la mankhwala |  | Pala enya, chonde zunulani zina lamankhwala? |  | If on Asthma medication, select medication from list below. Interviewer can also check in the book to confirm medications. |
| *THEOPHYLLINE* | A. Theophylline | 1  0  8  6 | Yes  No  Don't Know  Refused to answer | A. Theophylline | Eya  Ayi  Sindikudziwa  Refused to answer | A. Theophylline | Enya  Yayi  Nkhumanya yayi  Refused to answer | Record as responded. |
| *AMINOPHYLLINE* | B. Aminophylline | 1  0  8  6 | Yes  No  Don't Know  Refused to answer | B. Aminophylline | Eya  Ayi  Sindikudziwa  Refused to answer | B. Aminophylline | Enya  Yayi  Nkhumanya yayi  Refused to answer | Record as responded. |
| *SALBUTAMOL* | C. Salbutamol | 1  0  8  6 | Yes  No  Don't Know  Refused to answer | C. Salbutamol | Eya  Ayi  Sindikudziwa  Refused to answer | C. Salbutamol | Enya  Yayi  Nkhumanya yayi  Refused to answer | Record as responded. |
| *SALMETEROL* | D. Salmeterol | 1  0  8  6 | Yes  No  Don't Know  Refused to answer | D. Salmeterol | Eya  Ayi  Sindikudziwa  Refused to answer | D. Salmeterol | Enya  Yayi  Nkhumanya yayi  Refused to answer | Record as responded. |
| *IPRATROPIUMBROMIDE* | E. Ipratropium bromide | 1  0  8  6 | Yes  No  Don't Know  Refused to answer | E. Ipratropium bromide | Eya  Ayi  Sindikudziwa  Refused to answer | E. Ipratropium bromide | Enya  Yayi  Nkhumanya yayi  Refused to answer | Record as responded. |
| *SODIUMCROMOGLYCATE* | F. Sodium cromoglycate | 1  0  8  6 | Yes  No  Don't Know  Refused to answer | F. Sodium cromoglycate | Eya  Ayi  Sindikudziwa  Refused to answer | F. Sodium cromoglycate | Enya  Yayi  Nkhumanya yayi  Refused to answer | Record as responded. |
| *PREDNISOLONE* | G. Prednisolone | 1  0  8  6 | Yes  No  Don't Know  Refused to answer | G. Prednisolone | Eya  Ayi  Sindikudziwa  Refused to answer | G. Prednisolone | Enya  Yayi  Nkhumanya yayi  Refused to answer | Record as responded. |
| *OTHERASTHMAMED* | H. Other, specify |  |  | H. Other, specify |  | H. Other, specify |  | Specify if other. |
| *EPI_GROUP* | *Epilepsy* |  |  | *Epilepsy* |  | *Epilepsy* |  |  |
| *DIAEPI* | Have you ever been diagnosed with epilepsy/seizures by a doctor or other health professional? | 1  0  8  6 | Yes  No  Don't Know  Refused to answer | Kodi munayamba mwapezedwapo ndi nthenda yakugwa khunyu/kukomokakomoka ndi adotolo kapena anthu ena a zachipatala? | Eya  Ayi  Sindikudziwa  Refused to answer | Kasi muli kusangikapo na nthenda ya vizilisi /chakumutu/njili nawadokotala panji wa vyaumoyo waliyose? | Enya  Yayi  Nkhumanya yayi  Refused to answer | Record as responed.  Note: this question is asking for asthma confirmed by a **‘Professional’** not by field interviewers in previous MEIRU surveys. The interviewer can check in the health passport to confirm any diagnoses. |
| *MEDEPI* | Are you on regular medication for Epilepsy/seizures? | 1  0  8  6 | Yes  No  Don't Know  Refused to answer | Kodi mukulandira mankhwala a nthenda yakugwa khunyu/ kukomokakomoka? | Eya  Ayi  Sindikudziwa  Refused to answer | Kasi mukumwa mankhwala ya vizilisi /chakumutu/njili? | Enya  Yayi  Nkhumanya yayi  Refused to answer | Record as responded. |
| *EPI_MEDS* | Epilepsy medication |  |  | Epilepsy medication |  | Epilepsy medication |  | If on Asthma medication, select medication from list below. Interviewer can also check in the book to confirm medications. |
| *NOTE_REMEDEPI* | If yes, name of medication (s) |  |  | Ngati eya, chonde tchulani dzina la mankhwalawo |  | Pala enya, chonde zunulani zina lamankhwala? |  | Record as responded. |
| *MEDPH* | A. Phenobarbitone/ Pheno | 1  0  8  6 | Yes  No  Don't Know  Refused to answer | A. Phenobarbitone/ Pheno | Eya  Ayi  Sindikudziwa  Refused to answer | A. Phenobarbitone/ Pheno | Enya  Yayi  Nkhumanya yayi  Refused to answer | Record as responded. |
| *MEDCARB* | B. Carbamazapine/Carba/CBZ | 1  0  8  6 | Yes  No  Don't Know  Refused to answer | B. Carbamazapine/Carba/CBZ | Eya  Ayi  Sindikudziwa  Refused to answer | B. Carbamazapine/Carba/CBZ | Enya  Yayi  Nkhumanya yayi  Refused to answer | Record as responded. |
| *MEDPNY* | C. Phenytoin | 1  0  8  6 | Yes  No  Don't Know  Refused to answer | C. Phenytoin | Eya  Ayi  Sindikudziwa  Refused to answer | C. Phenytoin | Enya  Yayi  Nkhumanya yayi  Refused to answer | Record as responded. |
| *MED* | D. Sodium Valproate | 1  0  8  6 | Yes  No  Don't Know  Refused to answer | D. Sodium Valproate | Eya  Ayi  Sindikudziwa  Refused to answer | D. Sodium Valproate | Enya  Yayi  Nkhumanya yayi  Refused to answer | Record as responded. |
| *OTHERMEDEP1* | G. Other, specify [1] |  |  | G. Other, specify [1] |  | G. Other, specify [1] |  | Specify if other. |
| *OTHERMEDEPI2* | H. Other, specify [2] |  |  | H. Other, specify [2] |  | H. Other, specify [2] |  | Specify if other. |
| *OTHERMEDEPI3* | I. Other, specify [3] |  |  | I. Other, specify [3] |  | I. Other, specify [3] |  | Specify if other. |
| *MENT_GROUP* | *Mental health* |  |  | *Mental health* |  | *Mental health* |  |  |
| *DIAMENT* | Have you ever been diagnosed with a mental health problem by a doctor or other health professional? | 1  0  8  6 | Yes  No  Don't Know  Refused to answer | Kodi munayamba mwapezedwapo ndi nthenda ya maganizo angwiro ndi adotolo kapena anthu ena a zachipatala? | Eya  Ayi  Sindikudziwa  Refused to answer | Kasi muli kusangikapo na nthenda yakukola maghanoghano yawemi nabadokotala panji wa vyaumoyo waliyose? | Enya  Yayi  Nkhumanya yayi  Refused to answer | Record as responded.  Note: this question is asking for asthma confirmed by a **‘Professional’** not by field interviewers in previous MEIRU surveys. The interviewer can check in the health passport to confirm any diagnoses. |
| *MENT_TYPE* | Type of mental health problem |  |  | Type of mental health problem |  | Type of mental health problem |  |  |
| *NOTE_MENTYP* | What was the name of the mental health problem? |  |  | Kodi dzina la nthenda ya maganizo angwiro yomwe munapezeka nayo ndichiyani? |  | Kasi zina la nthenda yakukola maghanoghano yawemi yikaŵa vichi? |  | Select the mental health problem from list below. Interviewer can also check in health passport book to confirm. |
| *DEP* | A. Depression | 1  0  8  6 | Yes  No  Don't Know  Refused to answer | A. Depression | Eya  Ayi  Sindikudziwa  Refused to answer | A. Depression | Enya  Yayi  Nkhumanya yayi  Refused to answer | Record as responded. |
| *ANX* | B. Anxiety | 1  0  8  6 | Yes  No  Don't Know  Refused to answer | B. Anxiety | Eya  Ayi  Sindikudziwa  Refused to answer | B. Anxiety | Enya  Yayi  Nkhumanya yayi  Refused to answer | Record as responded. |
| *PSYC* | C. Psychosis/Schizophrenia ("Schizo") | 1  0  8  6 | Yes  No  Don't Know  Refused to answer | C. Psychosis/Schizophrenia ("Schizo") | Eya  Ayi  Sindikudziwa  Refused to answer | C. Psychosis/Schizophrenia ("Schizo") | Enya  Yayi  Nkhumanya yayi  Refused to answer | Record as responded. |
| *BIP* | D. Bipolar Affective Disorder/Mania | 1  0  8  6 | Yes  No  Don't Know  Refused to answer | D. Bipolar Affective Disorder/Mania | Eya  Ayi  Sindikudziwa  Refused to answer | D. Bipolar Affective Disorder/Mania | Enya  Yayi  Nkhumanya yayi  Refused to answer | Record as responded. |
| *ALCOD* | E. Alcohol dependence/harmful use | 1  0  8  6 | Yes  No  Don't Know  Refused to answer | E. Alcohol dependence/harmful use | Eya  Ayi  Sindikudziwa  Refused to answer | E. Alcohol dependence/harmful use | Enya  Yayi  Nkhumanya yayi  Refused to answer | Record as responded. |
| *OTHERD* | F. Other substance dependence/harmful use | 1  0  8  6 | Yes  No  Don't Know  Refused to answer | F. Other substance dependence/harmful use | Eya  Ayi  Sindikudziwa  Refused to answer | F. Other substance dependence/harmful use | Enya  Yayi  Nkhumanya yayi  Refused to answer | Record as responded. |
| *OTHERDMENT1* | G. Other, specify [1] |  |  | G. Other, specify [1] |  | G. Other, specify [1] |  | Specify if other. |
| *OTHERDMENT2* | H. Other, specify [2] |  |  | H. Other, specify [2] |  | H. Other, specify [2] |  | Specify if other. |
| *OTHERDMENT3* | I. Other, specify [3] |  |  | I. Other, specify [3] |  | I. Other, specify [3] |  | Specify if other. |
| *REGMEDMENT* | Are you on regular medication for a mental health problem? | 1  0  8  6 | Yes  No  Don't Know  Refused to answer | Kodi mukulandira mankhwala a nthenda ya maganizo angwiro? | Eya  Ayi  Sindikudziwa  Refused to answer | Kasi mukupokela mankhwala ya nthenda yakukola maghanoghano yawemi? | Enya  Yayi  Nkhumanya yayi  Refused to answer | Record as responded. |
| *MENT_MEDS* | Mental health medication |  |  | Mental health medication |  | Mental health medication |  |  |
| *NOTE_MENNOM* | If yes, name of medication (s) |  |  | Ngati eya, chonde tchulani dzina la mankhwalawo |  | Pala enya, chonde zunulani zina lamankhwala? |  | If on mental health medication, select medication from list below. Interviewer can also check in the book to confirm medications. |
| *MEDCH* | A. Chlorpromazine/ CPZ | 1  0  8  6 | Yes  No  Don't Know  Refused to answer | A. Chlorpromazine/ CPZ | Eya  Ayi  Sindikudziwa  Refused to answer | A. Chlorpromazine/ CPZ | Enya  Yayi  Nkhumanya yayi  Refused to answer | Record as responded. |
| *MEDHAL* | B. Haloperidol/ Haldol | 1  0  8  6 | Yes  No  Don't Know  Refused to answer | B. Haloperidol/ Haldol | Eya  Ayi  Sindikudziwa  Refused to answer | B. Haloperidol/ Haldol | Enya  Yayi  Nkhumanya yayi  Refused to answer | Record as responded. |
| *MEDAMI* | C. Amitryptyline (AMT) | 1  0  8  6 | Yes  No  Don't Know  Refused to answer | C. Amitryptyline (AMT) | Eya  Ayi  Sindikudziwa  Refused to answer | C. Amitryptyline (AMT) | Enya  Yayi  Nkhumanya yayi  Refused to answer | Record as responded. |
| *MEDFLUO* | D. Fluoxetine | 1  0  8  6 | Yes  No  Don't Know  Refused to answer | D. Fluoxetine | Eya  Ayi  Sindikudziwa  Refused to answer | D. Fluoxetine | Enya  Yayi  Nkhumanya yayi  Refused to answer | Record as responded. |
| *MEDCARBZPN* | E. Carbamazapine/Carba/CBZ | 1  0  8  6 | Yes  No  Don't Know  Refused to answer | E. Carbamazapine/Carba/CBZ | Eya  Ayi  Sindikudziwa  Refused to answer | E. Carbamazapine/Carba/CBZ | Enya  Yayi  Nkhumanya yayi  Refused to answer | Record as responded. |
| *MEDSOD* | F. Sodium Valproate/Na+ Valporate | 1  0  8  6 | Yes  No  Don't Know  Refused to answer | F. Sodium Valproate/Na+ Valporate | Eya  Ayi  Sindikudziwa  Refused to answer | F. Sodium Valproate/Na+ Valporate | Enya  Yayi  Nkhumanya yayi  Refused to answer | Specify if other |
| *OTHERMEDMENT1* | G. Other, specify [1] |  |  | G. Other, specify [1] |  | G. Other, specify [1] |  | Specify if other. |
| *OTHERMEDMENT2* | H. Other, specify [2] |  |  | H. Other, specify [2] |  | H. Other, specify [2] |  | Specify if other. |
| *OTHERMEDMENT3* | I. Other, specify [3] |  |  | I. Other, specify [3] |  | I. Other, specify [3] |  | Specify if other. |
| ***HIV_GROUP*** | **HIV and ART** |  |  | **HIV and ART** |  | **HIV and ART** |  |  |
| *EVERTEST* | Have you ever been tested for HIV? | 1  0  6 | Yes  No  Refused to answer | Kodi munayezedwako HIV? | Eya  Ayi  Sanayankhe  Refused to answer | Kasi mulikupimikapo HIV? | Enya  Yayi  Ndakana kuphala  Refused to answer | Record as responded. |
| *RECENTTESTDT_NOTE* | is the date of most recent test known? | 1  2  4  8  9 | Full date known  Day unknown  Day and month unknown  Not known at all  Missing | Kodi mudayezetsa liti komaliza? |  | Kasi mukapimika pawuli kawumaliro? |  | Ask the date of most recent test. Select the appropriate option and enter date as reported.  The tablet will promt you to re-nter date if date entered is invalid. |
| *HIVTESTDTDD* | Day |  |  | Day |  | Day |  |  |
| *HIVTESTDTMM* | Month |  |  | Month |  | Month |  |  |
| *HIVTESTDTYY* | Year |  |  | Year |  | Year |  |  |
| *RCNTRESULT* | What was the most recent result? | 0  1  2  6 | Negative  Positive  Participant was not given result  Refused to answer | Kodi zotsatira za kuyezedwa kwanu komalidza zinali zotani? | Negative  Positive  Participant was not given result  Refused to answer | Kasi ma lizati yakupimika kwinu kwa wumaliro yakawa yakuti wuli? | Negative  Positive  Participant was not given result  Refused to answer | Record as responded. |
| *HIVDOC* | FIELDWORKER: Test result documentation seen or verbal report? | 1  2 | Documentation seen  Verbal report | FIELDWORKER: Test result documentation seen or verbal report? | Documentation seen  Verbal report | FIELDWORKER: Test result documentation seen or verbal report? | Documentation seen  Verbal report | Record as responded. |
| *FRSTPOSTEST* | Was this the first time you tested positive? | 1  0  6 | Yes  No  Refused to answer | Kodi uku kunali kupezeka koyamba pozitivi? | Eya  Ayi  Refused to answer | Kasi uku kukaba kusangika kwakwamba pozitivi? | Enya  Yayi  Refused to answer | If yes, record if this was the first time testing positive. |
| *AGE1STTESTPOS* | What age were you when you first tested positive? |  |  | Kodi munali ndi zaka zingati pamene mudapezeka pozitivi? |  | Kasi mukaba na vyaka vilinga apo mukasangika pozitivi? |  | If no to above, record when age the participant tested positive (in years). |
| *ARVCURR* | Are you on antiretroviral therapy for HIV? | 1  0  6 | Yes  No  Refused to answer | Kodi mukumwa mankhwala a ARV (mankhwala a HIV)? | Eya  Ayi  Sanayankhe  Refused to answer | Kasi mukumwa mankhwala ya ARV (Mankhwala a HIV)? | Enya  Yayi  Ndakana kuphala  Refused to answer | Record as responded. |
| *ARVCURRSTRT* | If yes, how long ago did you start? |  |  | Ngati eya, munayamba liti kumwa mankhwalawa? |  | Pala enya, mukamba pauli? |  | If yes, record when ARV was started. In months/years. Enter ‘9998’ if unknown. |
| *ARVCURRMNTHS* | Months |  |  | Months |  | Months |  |  |
| *ARVCURRYRS* | Years |  |  | Years |  | Years |  |  |
| *ARVEVER* | Have you ever taken antiretroviral therapy? | 1  0  2  6 | Yes  No  No disclosure  Refused to answer | Kodi munamwako ma ARV (mankhwala a HIV)? | Eya  Ayi  Sanayankhe  Refused to answer | Muli kumwapo ma ARV (Mankhwala a HIV)? | Enya  Yayi  Ndakana kuphala  Refused to answer | If no to taking ARV, record whether taken ARV before. Record as responded. |
| *ARVEVERTRTMNT* | If yes, for how long did you take the treatment? |  |  | Ngati eya, munamwa mankhwalawa kwa nthawi yaitali bwanji? |  | Pala enya, nkhwanyengo yitali wuli mwakhala mukupokela mankhwala aya? |  | if yes, record for how long ARVs were taken in months/years. Enter ‘9998’ if unknown. |
| *ARVEVERMNTHS* | Months |  |  | Months |  | Months |  |  |
| *ARVEVERYRS* | Years |  |  | Years |  | Years |  |  |
| ***GFUNCT*** | **FUNCTIONING** |  |  | **FUNCTIONING** |  | **FUNCTIONING** |  |  |
| *WHODAS12* | WHODAS 12 |  |  | WHODAS 12 |  | WHODAS 12 |  |  |
| *WHODAS_NOTE* | GIVE THE WHODAS VISUAL AID TO THE PARTICIPANT  The following questions are about difficulties people have because of health conditions.  By health condition I mean diseases or illnesses, or other health problems that may be short or long lasting; injuries; mental or emotional problems; and problems with alcohol or drugs.  Remember to keep all of your health problems in mind as you answer the questions. When I ask you about difficulties in doing an activity think about: • Increased effort  • Discomfort or pain  • Slowness  • Changes in the way you do the activity.  Think back over the past 30 days and answer these questions, thinking about how much difficulty you had doing the following activities. The choices are as follows [POINT ON VISUAL AID]: No difficulty, Mild difficulty, Moderate difficulty; Severe difficulty; Extreme difficulty (or you cannot do the activity at all) |  |  | GIVE THE WHODAS VISUAL AID TO THE PARTICIPANT Mafunso otsatirawa ndiokhuza mavuto omwe anthu amakhala nawo chifukwa cha mavuto azaumoyo. Ndikati mavuto azaumoyo ndikutanthauza matenda kapena mavuto ena a zaummoyo omwe anthu amakhala nawo nthawi yayifupi kapena yayitali; kuvulala; mavuto a maganizo angwiro kapena kukhudzika mumtima; komanso mavuto amowa kapena mankhwala ozunguza ubongo.  Ganizirani za mavuto azaumoyo anu onse pamene mukuyankha mafunsowa. Ndikakufunsani za mavuto omwe mumakumana nawo pomwe mukuchita zinthu, ganizirani izi: • Kuchita kulimbana nazo pochita zinthu • Kusamva bwino kapena ululu • Kuchita zinthu motchedwa • Kusintha mmene mumachitira zinthu Ganizani za m’mbuyo masiku makumi atatu (30) apitawa poyankha mafunsowa. Ganizirani momwe mumavutikira kapena ngati munapezapo vuto kupanga zinthu zotsatirazi. Mayankho ali chonchi [POINT ON VISUAL AID]: Panalibe kuvutika; Ndimavutika pang’ono pokha; Ndimavutika mochulukirapo; Ndimavutika mochuluka, Ndimavutika mochuluka kwambiri kapena ndimakanikiratu kuchita zinthu. |  | GIVE THE WHODAS VISUAL AID TO THE PARTICIPANT Mafumbo yakulondezga aya yakukwaskana na maunonono ayo banthu wakusangana nayo chifukwa cha masuzgo ya vyaumoyo. Masuzgo ya vyaumoyo nkhung’anamula maulwali panji masuzgo yanyakhe ya vyaumoyo ayo yangawa ya kanyengo kachoko panji nyengo yitali, yakuvulazika, yamubongo/kaghanaghanilo panji ya kapulikilo kamumtima; namasuzgo yakukwaskana na mowa panji mankhwala yakunanga bongo.  Kumbukani kughanaghana vya masuzgo yinu yose ya vyaumoyo apo mukuzgola mafumbo aya. Pala nafumba fumbo lakukhwaskana na maunonono pala mukuchita vinthu mughanaghanenge vya ivyi: • Kuchita kujikakamizga pakuchita mlimo • Kupulika makola yayi panji kupulika ulwilwi • Kuchita vinthu mwakutchedwa • Kusintha mwakuyana naumo mukuchitilanga vinthu Ghanaghanani vya kumanyuma mumwezi panji mmazuba (30) ayo yajumpha nakuzgola mafumbo aya, mughanaghane naumo vikawila vinono kupanga vinthu vakulondezga ivi. Mazgolo yali nthena; [POINT ON VISUAL AID]: Unonono pakawavya, vinonono pachoko waka, vinononoko, vinonono chomene, vinonono nkhanila (mwakujumphizga)/kutondekelathu. |  | Explain to the participant that you will ask him/her questions about difficulties people have because of health conditions they have.  Read the introductory statement as slowly as possible and exactly as written in the tablet.  Inquire if there any questions from the introductory statement.  The WHODAS sections requires the use of the visual aid, however the interviewer will assess and decide whether the participant will be able to use the aid or not.  If the guide is going to be used, the interviewer guide the participant on how to use the visual aid options using the visual aid interviewers script. The interviewer has to cross-check (by randomly echoing the option said when a participant selects an option) to ensure the participants understands how to use the aid.  If the aid will not be used, the interviewer will read out the options to the participant after every question. |
| *DIFFSTAND* | In the past 30 days, how much difficulty did you have in standing for long periods such as 30 minutes? | 1  2  3  4  5  6 | None  Mild  Moderate  Severe  Extreme or cannot do  Refused to answer | Mumasiku 30 apitawa, mumavutika bwanji kuyima kwanthawi yayitali ngati mphindi 30? | Panalibe kuvutika  Ndimavutika pang’ono pokha  Ndimavutika mochulukirapo  Ndimavutika mochuluka  Ndimavutika mochuluka kwambiri kapena kukanika  Refused to answer | Mumazuba (30) ayo yajumpha, vikaba vinonono wuli kumilira kwa nyengo yitali nge ma miniti 30? | Unonono pakawavya  Vinonono pachoko waka  Vinononoko  Vinonono chomene  Vinonono nkhanila panji nkhatondekelatu  Refused to answer | Record as responded. Read out options if not using visual aid. |
| *DIFFTKCARE* | In the past 30 days, how much difficulty did you have in taking care of your household responsibilities? | 1  2  3  4  5  6 | None  Mild  Moderate  Severe  Extreme or cannot do  Refused to answer | Mumasiku 30 apitawa, mumavutika bwanji kukwaniritsa udindo wanu pakhomo? | Panalibe kuvutika  Ndimavutika pang’ono pokha  Ndimavutika mochulukirapo  Ndimavutika mochuluka  Ndimavutika mochuluka kwambiri kapena kukanika  Refused to answer | Mumazuba (30) ayo yajumpha, vikaba vinonono wuli kukwaniriska udindo winu panyumba? | Unonono pakawavya  Vinonono pachoko waka  Vinononoko  Vinonono chomene  Vinonono nkhanila panji nkhatondekelatu  Refused to answer | Record as responded. Read out options if not using visual aid. |
| *DIFFLEARN* | In the past 30 days, how much difficulty did you have in learning a new task, for example, learning how to get to a new place? | 1  2  3  4  5  6 | None  Mild  Moderate  Severe  Extreme or cannot do  Refused to answer | Mumasiku 30 apitawa, mumavutika bwanji kuphunzila zinthu zatsopano monga kupita umalo achilendo? | Panalibe kuvutika  Ndimavutika pang’ono pokha  Ndimavutika mochulukirapo  Ndimavutika mochuluka  Ndimavutika mochuluka kwambiri kapena kukanika  Refused to answer | Mumazuba (30) ayo yajumpha, vikaba vinonono wuli kusambira vinthu vya sono nge kuluta ku malo yachilendo? | Unonono pakawavya  Vinonono pachoko waka  Vinononoko  Vinonono chomene  Vinonono nkhanila panji nkhatondekelatu  Refused to answer | Record as responded. Read out options if not using visual aid. |
| *DIFFJOINCOMACT* | In the past 30 days, how much of a problem did you have in joining in community activities (for example, festivities, religious or other activities) in the same way as anyone else can? | 1  2  3  4  5  6 | None  Mild  Moderate  Severe  Extreme or cannot do  Refused to answer | Mumasiku 30 apitawa mumavutika bwanji kuti mu zitenga nawo mbali pazinthu zochitika kumene mumakhala (ngati zisangaralo, zochitika zaku mpingo kapena zina) ngati mmene amapangira anthu ena? | Panalibe kuvutika  Ndimavutika pang’ono pokha  Ndimavutika mochulukirapo  Ndimavutika mochuluka  Ndimavutika mochuluka kwambiri kapena kukanika  Refused to answer | Mumazuba (30) ayo yajumpha, vikaba vinonono wuli kuti mutole nawo lwande pa vinthu vyakuchitika mukaya (nge vyavikondwelelo, vyakutchalitchi, panji vinyake) nga umo wakupangira wanthu wanyakhe? | Unonono pakawavya  Vinonono pachoko waka  Vinononoko  Vinonono chomene  Vinonono nkhanila panji nkhatondekelatu  Refused to answer | Record as responded. Read out options if not using visual aid. |
| *EMOTAFFCT* | In the past 30 days, how much have you been emotionally affected by your health condition? | 1  2  3  4  5  6 | None  Mild  Moderate  Severe  Extreme or cannot do  Refused to answer | Mumasiku 30 apitawa mumtima mwanu munakhudzika bwanji ndi umoyo wanu? | Panalibe kuvutika  Ndimavutika pang’ono pokha  Ndimavutika mochulukirapo  Ndimavutika mochuluka  Ndimavutika mochuluka kwambiri kapena kukanika  Refused to answer | Mumazuba (30) ayo yajumpha, kasi mwakhwaskika wuli mumtima winu na kabilo kavyaumoyo winu? | Unonono pakawavya  Vinonono pachoko waka  Vinononoko  Vinonono chomene  Vinonono nkhanila panji nkhatondekelatu  Refused to answer | Record as responded. Read out options if not using visual aid. |
| *DIFFCONCENT* | In the past 30 days, how much difficulty did you have in concentrating on doing something for ten minutes? | 1  2  3  4  5  6 | None  Mild  Moderate  Severe  Extreme or cannot do  Refused to answer | Mumasiku 30 apitawa, mumavutika bwanji kupanga zithu mokhazikika/modekha kwa mphindi khumi (10)? | Panalibe kuvutika  Ndimavutika pang’ono pokha  Ndimavutika mochulukirapo  Ndimavutika mochuluka  Ndimavutika mochuluka kwambiri kapena kukanika  Refused to answer | Mumazuba (30) ayo yajumpha, vikaba vinonono wuli kupanga vinthu mwakukhazgikika kwa maminiti 10? | Unonono pakawavya  Vinonono pachoko waka  Vinononoko  Vinonono chomene  Vinonono nkhanila panji nkhatondekelatu  Refused to answer | Record as responded. Read out options if not using visual aid. |
| *DIFFWALKLNGDIST* | In the past 30 days, how much difficulty did you have in walking a long distance such as a kilometre [or equivalent]? | 1  2  3  4  5  6 | None  Mild  Moderate  Severe  Extreme or cannot do  Refused to answer | Mumasiku 30 apitawa, mumavutika bwanji kuyenda pansi ulendo wawutali ngati kilomita imodzi (kapena ulendo ofanana nawo)? | Panalibe kuvutika  Ndimavutika pang’ono pokha  Ndimavutika mochulukirapo  Ndimavutika mochuluka  Ndimavutika mochuluka kwambiri kapena kukanika  Refused to answer | Mumazuba (30) ayo yajumpha, vikawa vinonono wuli kwenda pasi ulendo utali ngati kutalika kwa kilomita yimoza (panji ulendo wakuyana nawo)? | Unonono pakawavya  Vinonono pachoko waka  Vinononoko  Vinonono chomene  Vinonono nkhanila panji nkhatondekelatu  Refused to answer | Record as responded. Read out options if not using visual aid. |
| *DIFFWSHBODY* | In the past 30 days, how much difficulty did you have in washing your whole body? | 1  2  3  4  5  6 | None  Mild  Moderate  Severe  Extreme or cannot do  Refused to answer | Mumasiku 30 apitawa, mumavutika bwanji kusamba thupi lanu lonse? | Panalibe kuvutika  Ndimavutika pang’ono pokha  Ndimavutika mochulukirapo  Ndimavutika mochuluka  Ndimavutika mochuluka kwambiri kapena kukanika  Refused to answer | Mumazuba (30) ayo yajumpha, vikaba vinonono wuli kugeza thupi linu lose? | Unonono pakawavya  Vinonono pachoko waka  Vinononoko  Vinonono chomene  Vinonono nkhanila panji nkhatondekelatu  Refused to answer | Record as responded. Read out options if not using visual aid. |
| *DIFFGETDRESS* | In the past 30 days, how much difficulty did you have in getting dressed? | 1  2  3  4  5  6 | None  Mild  Moderate  Severe  Extreme or cannot do  Refused to answer | Mumasiku 30 apitawa, mumavutika bwanji kuvala zovala? | Panalibe kuvutika  Ndimavutika pang’ono pokha  Ndimavutika mochulukirapo  Ndimavutika mochuluka  Ndimavutika mochuluka kwambiri kapena kukanika  Refused to answer | Mumazuba (30) ayo yajumpha, vikaba vinonono wuli kuvwala vyakuvwala? | Unonono pakawavya  Vinonono pachoko waka  Vinononoko  Vinonono chomene  Vinonono nkhanila panji nkhatondekelatu  Refused to answer | Record as responded. Read out options if not using visual aid. |
| *DIFFDEALPPL* | In the past 30 days, how much difficulty did you have in dealing with people you do not know? | 1  2  3  4  5  6 | None  Mild  Moderate  Severe  Extreme or cannot do  Refused to answer | Mumasiku 30 apitawa, mumavutika bwanji kutakataka ndi anthu osawaziwa? | Panalibe kuvutika  Ndimavutika pang’ono pokha  Ndimavutika mochulukirapo  Ndimavutika mochuluka  Ndimavutika mochuluka kwambiri kapena kukanika  Refused to answer | Mumazuba (30) ayo yajumpha, vikaba vinonono wuli kusangana na kuyowoya na wanthu wambula kuwamanya? | Unonono pakawavya  Vinonono pachoko waka  Vinononoko  Vinonono chomene  Vinonono nkhanila panji nkhatondekelatu  Refused to answer | Record as responded. Read out options if not using visual aid. |
| *DIFFMAINTAINFRND* | In the past 30 days, how much difficulty did you have in maintaining a friendship? | 1  2  3  4  5  6 | None  Mild  Moderate  Severe  Extreme or cannot do  Refused to answer | Mumasiku 30 apitawa, mumavutika bwanji kupitiriza ubale ndi anzanu? | Panalibe kuvutika  Ndimavutika pang’ono pokha  Ndimavutika mochulukirapo  Ndimavutika mochuluka  Ndimavutika mochuluka kwambiri kapena kukanika  Refused to answer | Mumazuba (30) ayo yajumpha, vikaba vinonono wuli kulutirizga ubale na wanyinu? | Unonono pakawavya  Vinonono pachoko waka  Vinononoko  Vinonono chomene  Vinonono nkhanila panji nkhatondekelatu  Refused to answer | Record as responded. Read out options if not using visual aid. |
| *DIFFDAYTODAY* | In the past 30 days, how much difficulty did you have in your day-to-day work/school? | 1  2  3  4  5  6 | None  Mild  Moderate  Severe  Extreme or cannot do  Refused to answer | Mumasiku 30 apitawa, mumavutika bwanji ndi ntchito kapena sukulu yanu ya tsiku ndi tsiku? | Panalibe kuvutika  Ndimavutika pang’ono pokha  Ndimavutika mochulukirapo  Ndimavutika mochuluka  Ndimavutika mochuluka kwambiri kapena kukanika  Refused to answer | Mumazuba (30) ayo yajumpha, vikaba vinonono wuli kupanga vinthu vinu vya zuba na zuba nge kugwila ntchito panji kuluta kusukulu? | Unonono pakawavya  Vinonono pachoko waka  Vinononoko  Vinonono chomene  Vinonono nkhanila panji nkhatondekelatu  Refused to answer | Record as responded. Read out options if not using visual aid. |
| *WHODAS12* | WHODAS 12 |  |  | WHODAS 12 |  | WHODAS 12 |  |  |
| *WHODAS_NOTE* | GIVE THE WHODAS VISUAL AID TO THE PARTICIPANT  The following questions are about difficulties people have because of health conditions.  By health condition I mean diseases or illnesses, or other health problems that may be short or long lasting; injuries; mental or emotional problems; and problems with alcohol or drugs.  Remember to keep all of your health problems in mind as you answer the questions. When I ask you about difficulties in doing an activity think about: • Increased effort  • Discomfort or pain  • Slowness  • Changes in the way you do the activity.  Think back over the past 30 days and answer these questions, thinking about how much difficulty you had doing the following activities. The choices are as follows [POINT ON VISUAL AID]: No difficulty, Mild difficulty, Moderate difficulty; Severe difficulty; Extreme difficulty (or you cannot do the activity at all) |  |  | GIVE THE WHODAS VISUAL AID TO THE PARTICIPANT Mafunso otsatirawa ndiokhuza mavuto omwe anthu amakhala nawo chifukwa cha mavuto azaumoyo. Ndikati mavuto azaumoyo ndikutanthauza matenda kapena mavuto ena a zaummoyo omwe anthu amakhala nawo nthawi yayifupi kapena yayitali; kuvulala; mavuto a maganizo angwiro kapena kukhudzika mumtima; komanso mavuto amowa kapena mankhwala ozunguza ubongo.  Ganizirani za mavuto azaumoyo anu onse pamene mukuyankha mafunsowa. Ndikakufunsani za mavuto omwe mumakumana nawo pomwe mukuchita zinthu, ganizirani izi: • Kuchita kulimbana nazo pochita zinthu • Kusamva bwino kapena ululu • Kuchita zinthu motchedwa • Kusintha mmene mumachitira zinthu Ganizani za m’mbuyo masiku makumi atatu (30) apitawa poyankha mafunsowa. Ganizirani momwe mumavutikira kapena ngati munapezapo vuto kupanga zinthu zotsatirazi. Mayankho ali chonchi [POINT ON VISUAL AID]: Panalibe kuvutika; Ndimavutika pang’ono pokha; Ndimavutika mochulukirapo; Ndimavutika mochuluka, Ndimavutika mochuluka kwambiri kapena ndimakanikiratu kuchita zinthu. |  | GIVE THE WHODAS VISUAL AID TO THE PARTICIPANT Mafumbo yakulondezga aya yakukwaskana na maunonono ayo banthu wakusangana nayo chifukwa cha masuzgo ya vyaumoyo. Masuzgo ya vyaumoyo nkhung’anamula maulwali panji masuzgo yanyakhe ya vyaumoyo ayo yangawa ya kanyengo kachoko panji nyengo yitali, yakuvulazika, yamubongo/kaghanaghanilo panji ya kapulikilo kamumtima; namasuzgo yakukwaskana na mowa panji mankhwala yakunanga bongo.  Kumbukani kughanaghana vya masuzgo yinu yose ya vyaumoyo apo mukuzgola mafumbo aya. Pala nafumba fumbo lakukhwaskana na maunonono pala mukuchita vinthu mughanaghanenge vya ivyi: • Kuchita kujikakamizga pakuchita mlimo • Kupulika makola yayi panji kupulika ulwilwi • Kuchita vinthu mwakutchedwa • Kusintha mwakuyana naumo mukuchitilanga vinthu Ghanaghanani vya kumanyuma mumwezi panji mmazuba (30) ayo yajumpha nakuzgola mafumbo aya, mughanaghane naumo vikawila vinono kupanga vinthu vakulondezga ivi. Mazgolo yali nthena; [POINT ON VISUAL AID]: Unonono pakawavya, vinonono pachoko waka, vinononoko, vinonono chomene, vinonono nkhanila (mwakujumphizga)/kutondekelathu. |  | Explain to the participant that you will ask him/her questions about difficulties people have because of health conditions they have.  Read the introductory statement as slowly as possible and exactly as written in the tablet.  Inquire if there any questions from the introductory statement.  The WHODAS sections requires the use of the visual aid, however the interviewer will assess and decide whether the participant will be able to use the aid or not.  If the guide is going to be used, the interviewer guide the participant on how to use the visual aid options using the visual aid interviewers script. The interviewer has to cross-check (by randomly echoing the option said when a participant selects an option) to ensure the participants understands how to use the aid.  If the aid will not be used, the interviewer will read out the options to the participant after every question. |
| *DIFFSTAND* | In the past 30 days, how much difficulty did you have in standing for long periods such as 30 minutes? | 1  2  3  4  5  6 | None  Mild  Moderate  Severe  Extreme or cannot do  Refused to answer | Mumasiku 30 apitawa, mumavutika bwanji kuyima kwanthawi yayitali ngati mphindi 30? | Panalibe kuvutika  Ndimavutika pang’ono pokha  Ndimavutika mochulukirapo  Ndimavutika mochuluka  Ndimavutika mochuluka kwambiri kapena kukanika  Refused to answer | Mumazuba (30) ayo yajumpha, vikaba vinonono wuli kumilira kwa nyengo yitali nge ma miniti 30? | Unonono pakawavya  Vinonono pachoko waka  Vinononoko  Vinonono chomene  Vinonono nkhanila panji nkhatondekelatu  Refused to answer | Record as responded. Read out options if not using visual aid. |
| *DIFFTKCARE* | In the past 30 days, how much difficulty did you have in taking care of your household responsibilities? | 1  2  3  4  5  6 | None  Mild  Moderate  Severe  Extreme or cannot do  Refused to answer | Mumasiku 30 apitawa, mumavutika bwanji kukwaniritsa udindo wanu pakhomo? | Panalibe kuvutika  Ndimavutika pang’ono pokha  Ndimavutika mochulukirapo  Ndimavutika mochuluka  Ndimavutika mochuluka kwambiri kapena kukanika  Refused to answer | Mumazuba (30) ayo yajumpha, vikaba vinonono wuli kukwaniriska udindo winu panyumba? | Unonono pakawavya  Vinonono pachoko waka  Vinononoko  Vinonono chomene  Vinonono nkhanila panji nkhatondekelatu  Refused to answer | Record as responded. Read out options if not using visual aid. |
| *DIFFLEARN* | In the past 30 days, how much difficulty did you have in learning a new task, for example, learning how to get to a new place? | 1  2  3  4  5  6 | None  Mild  Moderate  Severe  Extreme or cannot do  Refused to answer | Mumasiku 30 apitawa, mumavutika bwanji kuphunzila zinthu zatsopano monga kupita umalo achilendo? | Panalibe kuvutika  Ndimavutika pang’ono pokha  Ndimavutika mochulukirapo  Ndimavutika mochuluka  Ndimavutika mochuluka kwambiri kapena kukanika  Refused to answer | Mumazuba (30) ayo yajumpha, vikaba vinonono wuli kusambira vinthu vya sono nge kuluta ku malo yachilendo? | Unonono pakawavya  Vinonono pachoko waka  Vinononoko  Vinonono chomene  Vinonono nkhanila panji nkhatondekelatu  Refused to answer | Record as responded. Read out options if not using visual aid. |
| *DIFFJOINCOMACT* | In the past 30 days, how much of a problem did you have in joining in community activities (for example, festivities, religious or other activities) in the same way as anyone else can? | 1  2  3  4  5  6 | None  Mild  Moderate  Severe  Extreme or cannot do  Refused to answer | Mumasiku 30 apitawa mumavutika bwanji kuti mu zitenga nawo mbali pazinthu zochitika kumene mumakhala (ngati zisangaralo, zochitika zaku mpingo kapena zina) ngati mmene amapangira anthu ena? | Panalibe kuvutika  Ndimavutika pang’ono pokha  Ndimavutika mochulukirapo  Ndimavutika mochuluka  Ndimavutika mochuluka kwambiri kapena kukanika  Refused to answer | Mumazuba (30) ayo yajumpha, vikaba vinonono wuli kuti mutole nawo lwande pa vinthu vyakuchitika mukaya (nge vyavikondwelelo, vyakutchalitchi, panji vinyake) nga umo wakupangira wanthu wanyakhe? | Unonono pakawavya  Vinonono pachoko waka  Vinononoko  Vinonono chomene  Vinonono nkhanila panji nkhatondekelatu  Refused to answer | Record as responded. Read out options if not using visual aid. |
| *EMOTAFFCT* | In the past 30 days, how much have you been emotionally affected by your health condition? | 1  2  3  4  5  6 | None  Mild  Moderate  Severe  Extreme or cannot do  Refused to answer | Mumasiku 30 apitawa mumtima mwanu munakhudzika bwanji ndi umoyo wanu? | Panalibe kuvutika  Ndimavutika pang’ono pokha  Ndimavutika mochulukirapo  Ndimavutika mochuluka  Ndimavutika mochuluka kwambiri kapena kukanika  Refused to answer | Mumazuba (30) ayo yajumpha, kasi mwakhwaskika wuli mumtima winu na kabilo kavyaumoyo winu? | Unonono pakawavya  Vinonono pachoko waka  Vinononoko  Vinonono chomene  Vinonono nkhanila panji nkhatondekelatu  Refused to answer | Record as responded. Read out options if not using visual aid. |
| *DIFFCONCENT* | In the past 30 days, how much difficulty did you have in concentrating on doing something for ten minutes? | 1  2  3  4  5  6 | None  Mild  Moderate  Severe  Extreme or cannot do  Refused to answer | Mumasiku 30 apitawa, mumavutika bwanji kupanga zithu mokhazikika/modekha kwa mphindi khumi (10)? | Panalibe kuvutika  Ndimavutika pang’ono pokha  Ndimavutika mochulukirapo  Ndimavutika mochuluka  Ndimavutika mochuluka kwambiri kapena kukanika  Refused to answer | Mumazuba (30) ayo yajumpha, vikaba vinonono wuli kupanga vinthu mwakukhazgikika kwa maminiti 10? | Unonono pakawavya  Vinonono pachoko waka  Vinononoko  Vinonono chomene  Vinonono nkhanila panji nkhatondekelatu  Refused to answer | Record as responded. Read out options if not using visual aid. |
| *DIFFWALKLNGDIST* | In the past 30 days, how much difficulty did you have in walking a long distance such as a kilometre [or equivalent]? | 1  2  3  4  5  6 | None  Mild  Moderate  Severe  Extreme or cannot do  Refused to answer | Mumasiku 30 apitawa, mumavutika bwanji kuyenda pansi ulendo wawutali ngati kilomita imodzi (kapena ulendo ofanana nawo)? | Panalibe kuvutika  Ndimavutika pang’ono pokha  Ndimavutika mochulukirapo  Ndimavutika mochuluka  Ndimavutika mochuluka kwambiri kapena kukanika  Refused to answer | Mumazuba (30) ayo yajumpha, vikawa vinonono wuli kwenda pasi ulendo utali ngati kutalika kwa kilomita yimoza (panji ulendo wakuyana nawo)? | Unonono pakawavya  Vinonono pachoko waka  Vinononoko  Vinonono chomene  Vinonono nkhanila panji nkhatondekelatu  Refused to answer | Record as responded. Read out options if not using visual aid. |
| *DIFFWSHBODY* | In the past 30 days, how much difficulty did you have in washing your whole body? | 1  2  3  4  5  6 | None  Mild  Moderate  Severe  Extreme or cannot do  Refused to answer | Mumasiku 30 apitawa, mumavutika bwanji kusamba thupi lanu lonse? | Panalibe kuvutika  Ndimavutika pang’ono pokha  Ndimavutika mochulukirapo  Ndimavutika mochuluka  Ndimavutika mochuluka kwambiri kapena kukanika  Refused to answer | Mumazuba (30) ayo yajumpha, vikaba vinonono wuli kugeza thupi linu lose? | Unonono pakawavya  Vinonono pachoko waka  Vinononoko  Vinonono chomene  Vinonono nkhanila panji nkhatondekelatu  Refused to answer | Record as responded. Read out options if not using visual aid. |
| *DIFFGETDRESS* | In the past 30 days, how much difficulty did you have in getting dressed? | 1  2  3  4  5  6 | None  Mild  Moderate  Severe  Extreme or cannot do  Refused to answer | Mumasiku 30 apitawa, mumavutika bwanji kuvala zovala? | Panalibe kuvutika  Ndimavutika pang’ono pokha  Ndimavutika mochulukirapo  Ndimavutika mochuluka  Ndimavutika mochuluka kwambiri kapena kukanika  Refused to answer | Mumazuba (30) ayo yajumpha, vikaba vinonono wuli kuvwala vyakuvwala? | Unonono pakawavya  Vinonono pachoko waka  Vinononoko  Vinonono chomene  Vinonono nkhanila panji nkhatondekelatu  Refused to answer | Record as responded. Read out options if not using visual aid. |
| *DIFFDEALPPL* | In the past 30 days, how much difficulty did you have in dealing with people you do not know? | 1  2  3  4  5  6 | None  Mild  Moderate  Severe  Extreme or cannot do  Refused to answer | Mumasiku 30 apitawa, mumavutika bwanji kutakataka ndi anthu osawaziwa? | Panalibe kuvutika  Ndimavutika pang’ono pokha  Ndimavutika mochulukirapo  Ndimavutika mochuluka  Ndimavutika mochuluka kwambiri kapena kukanika  Refused to answer | Mumazuba (30) ayo yajumpha, vikaba vinonono wuli kusangana na kuyowoya na wanthu wambula kuwamanya? | Unonono pakawavya  Vinonono pachoko waka  Vinononoko  Vinonono chomene  Vinonono nkhanila panji nkhatondekelatu  Refused to answer | Record as responded. Read out options if not using visual aid. |
| *DIFFMAINTAINFRND* | In the past 30 days, how much difficulty did you have in maintaining a friendship? | 1  2  3  4  5  6 | None  Mild  Moderate  Severe  Extreme or cannot do  Refused to answer | Mumasiku 30 apitawa, mumavutika bwanji kupitiriza ubale ndi anzanu? | Panalibe kuvutika  Ndimavutika pang’ono pokha  Ndimavutika mochulukirapo  Ndimavutika mochuluka  Ndimavutika mochuluka kwambiri kapena kukanika  Refused to answer | Mumazuba (30) ayo yajumpha, vikaba vinonono wuli kulutirizga ubale na wanyinu? | Unonono pakawavya  Vinonono pachoko waka  Vinononoko  Vinonono chomene  Vinonono nkhanila panji nkhatondekelatu  Refused to answer | Record as responded. Read out options if not using visual aid. |
| *GFUNCT* |  |  |  |  |  |  |  |  |
| *COGTEST* | *[NOTE TO FIELDWORKER] Please now administer the cognitive testing* |  |  | *[NOTE TO FIELDWORKER] Please now administer the cognitive testing* |  | *[NOTE TO FIELDWORKER] Please now administer the cognitive testing* |  |  |
| **GMH** | **MENTAL HEALTH** |  |  | **MENTAL HEALTH** |  | **MENTAL HEALTH** |  |  |
| *INTRO* | I am now going to ask you some questions about how you have been thinking and feeling during the past two weeks.   There are 4 possible answers for each question:   “Not at all”, means you have not had the symptom at all in the last 2 weeks. “Sometimes”, means you have had the symptom but less than half the days in the past two weeks. “Usually”, means you have had the symptom on more than half the days but not all the time in the past two weeks. Finally, “Almost always” means you have had the symptom every day, or almost every day.   [IF YOU THINK THAT THE PARTICIPANT WOULD BENEFIT FROM USING THE WATER GLASSES VISUAL AID, SHOW IT TO THE PARTICIPANT AND GIVE THE FOLLOWING INSTRUCTIONS]  [Point at empty glass] “Not at all”, means you have not had the symptom at all in the last 2 weeks. [Point at 1/3 full glass] “Sometimes”, means you have had the symptom but less than half the days in the past two weeks. [Point at 2/3 full glass]. “Usually”, means you have had the symptom on more than half the days but not all the time in the past two weeks. [Point at full glass], Finally “Almost always” means you have had the symptom every day, or almost every day. |  |  | Pano ndikufunsani mafunso okhudzana ndi momwe mwakhala mukuganizira ndi kumvera mu mtima mwanu mu masabata awiri apitawa.   Pali mayankho anayi pafunso lina lililonse:    Yankho loti “Palibe” likutanthauza kuti simunakhalepo ndi vuto limene mwafunsidwa mu masabata awiri apitawa. Yankho loti “Nthawi zina” likutanthauza kuti mwakhala ndi vutoli koma kochepera theka (hafu) la masiku a masabata awiri apitawa. Yankho loti “Kawirikawiri” likutanthauza kuti mwakhala ndi vutoli koposera theka (hafu) la masabata awiri apitawa, koma osati tisku ndi tsiku. Yankho lomalizira ndi loti “Pafupifupi nthawi zonse”, limene likutanthauza kuti mwakhala ndi vuto limene mwafunsidwali tsiku lililonse, kapena pafupifupi tsiku lilonse.   [IF YOU THINK THAT THE PARTICIPANT WOULD BENEFIT FROM USING THE WATER GLASSES VISUAL AID, SHOW IT TO THE PARTICIPANT AND GIVE THE FOLLOWING INSTRUCTIONS]  [Point at empty glass] Yankho loti “Palibe” likutanthauza kuti simunakhalepo ndi vuto limene mwafunsidwa mu masabata awiri apitawa. [Point at 1/3 full glass]. Yankho loti “Nthawi zina” likutanthauza kuti mwakhala ndi vutoli koma kochepera theka (hafu) la masiku a masabata awiri apitawa. [Point at 2/3 full glass] Yankho loti “Kawirikawiri” likutanthauza kuti mwakhala ndi vutoli koposera theka (hafu) la masabata awiri apitawa, koma osati tisku ndi tsiku. [Point at full glass] Yankho lomalizira ndi loti “Pafupifupi nthawi zonse”, limene likutanthauza kuti mwakhala ndi vuto limene mwafunsidwali tsiku lililonse, kapena pafupifupi tsiku lilonse. |  | Sono nikufumbaninge mafumbo yakukhwaskana na umo mwakhala mukughanaghanira na kupulikira mu mtima winu mu masabata yawiri aya yajumpha.   Pali mazgolo yanayi pafumbo lililose:  Zgolo lakuti “Palije” likungánamula kuti mundawepo na suzgo ilo mwafumbika mu masabata yawiri yajumpha. Zgolo lakuti “Nyengo zinyakhe” likungánamula kuti mwawa na sugzo ili kweni kwakuchepera hafu ya mazuwa ya mu masabata yawiri yajumpha. Zgolo lakuti “Kawirikawiri” likungánamula kuti mwawa na sugzo ili kwakujumpha hafu ya masabata yawiri yajumpha, kweni dazi na dazi yayi. Zgolo lakuti “Pafupifupi nyengo zose” likungánamula kuti mwawa na suzgo ilo mwafumbika kwa dazi lililose, panji pafupifupi dazi lililose.   [IF YOU THINK THAT THE PARTICIPANT WOULD BENEFIT FROM USING THE WATER GLASSES VISUAL AID, SHOW IT TO THE PARTICIPANT AND GIVE THE FOLLOWING INSTRUCTIONS]  [Point at empty glass] Zgolo lakuti “Palije” likungánamula kuti mundawepo na suzgo ilo mwafumbika mu masabata yawiri yajumpha. [Point at 1/3 full glass]. Zgolo lakuti “Nyengo zinyakhe” likungánamula kuti mwawa na sugzo ili kweni kwakuchepera hafu ya mazuwa ya mu masabata yawiri yajumpha. [Point at 2/3 full glass]. Zgolo lakuti “Kawirikawiri” likungánamula kuti mwawa na sugzo ili kwakujumpha hafu ya masabata yawiri yajumpha, kweni dazi na dazi yayi. [Point at full glass] Zgolo lakuti “Pafupifupi nyengo zose” likungánamula kuti mwawa na suzgo ilo mwafumbika kwa dazi lililose, panji pafupifupi dazi lililose. |  | This section asks participants on mental health symptoms they have been experiencing in the past two weeks. The interviewer will also assess whether the participant can use the water glass visual aids and explain it to them as written in the introductory script. If the interviewer thinks the participant will not benefit from using the visual aid, (s)he should read out the four options to the participant.  Each question has a note of an example of how a question should be rephrased if the participant does not understand it. Interviewer should read this slowly and as exactly as written |
| *PHQ9* | PHQ9 |  |  | PHQ9 |  | PHQ9 |  |  |
| *FEELDOWN* | During the past two weeks, how much have you been feeling down, depressed, or hopeless? | 0  1  2  3  6 | Not at all  Sometimes  Usually  Almost Always  Refused to answer | Mu masabata awiri apitawa, ndi mochuluka bwanji pamene mwakhala osasangalala, wokhumudwa, kapena opanda chiyembekezo? | Palibe  Nthawi zina  Kawirikawiri  Pafupifupi nthawi zonse  Refused to answer | Mu masabata yawiri ayo yajumpha, nkhanandi uli ako mwawa wambula kukondwa, kukwebeka, panji kuwavya chigomezgo? | Palije  Nyengo zinyakhe  Kawirikawiri  Pafupifupi nyengo zose  Refused to answer | Record as responded. Read out the options if not using the visual aid. |
| *FEELDOWN_NOTE* | [IF THE PARTICIPANT IS NOT SURE WHAT THE QUESTION MEANS, READ THIS EXAMPLE AS IT IS WRITTEN]  Sometimes a person feels miserable because of something in their life or even for no clear reason; or feels that nothing they do will make life better. How much has this happened to you in the last 2 weeks? |  |  | [IF THE PARTICIPANT IS NOT SURE WHAT THE QUESTION MEANS, READ THIS EXAMPLE AS IT IS WRITTEN]  Nthawi zina munthu amatha kukhala wokhumudwa chifukwa cha chinthu china m’moyo mwake kapenanso opanda chifukwa chimene; komanso amatha kukhala ndi maganizo akuti palibe chomwe angachite kuti akometse moyo wake. Ndi mochuluka bwanji zimenezi zinakuchitikirani mu masabata awiri apitawa? |  | [IF THE PARTICIPANT IS NOT SURE WHAT THE QUESTION MEANS, READ THIS EXAMPLE AS IT IS WRITTEN]  Ngengo zinyake munthu wakujipulika wambula kukondwa na ivyo vikuchitika pa moyo wakhe nanga pangaba chifukwa chambula kupulikwika pakunji palije icho bangachita chakuti chipangiske moyo wabo wube makora. Nkhanandi uli ivi vyamuchitikiranipo mumasabata yawiri ayo yajumpha? |  | Read note if participant does not understand question. |
| *ANHEDONIA* | During the past two weeks, how much have you had little interest or pleasure in doing things? | 0  1  2  3  6 | Not at all  Sometimes  Usually  Almost Always  Refused to answer | Mu masabata awiri apitawa, ndi mochuluka bwanji pamene mwakhala ndi chidwi chochepa kapena osasangalatsidwa pochita zinthu? | Palibe  Nthawi zina  Kawirikawiri  Pafupifupi nthawi zonse  Refused to answer | Mu masabata yawiri ayo yajumpha, nkhanandi uli ako mwawa wa kunweka kwa pachoko panji kukhala wambula kusangwa pakuchita vinthu? | Palije  Nyengo zinyakhe  Kawirikawiri  Pafupifupi nyengo zose  Refused to answer | Record as responded. Read out the options if not using the visual aid. |
| *ANHEDONIA_NOTE* | [IF THE PARTICIPANT IS NOT SURE WHAT THE QUESTION MEANS, READ THIS EXAMPLE AS IT IS WRITTEN]   Sometimes a person gets no pleasure or has no interest when doing things such as cooking, working or meeting with friends, even though he/she used to enjoy those things. How much has this happened to you in the last 2 weeks? |  |  | [IF THE PARTICIPANT IS NOT SURE WHAT THE QUESTION MEANS, READ THIS EXAMPLE AS IT IS WRITTEN]  Nthawi zina munthu amatha kukhala opanda chidwi kapena osasangalatsidwa pochita zinthu monga kuphika, kugwira ntchito kapena kukumana ndi anzake, ngakhale m’mbuyomu anali kusangalatsidwa nazo. Ndi mochuluka bwanji zimenezi zinakuchitikirani mu masabata awiri apitawa? |  | [IF THE PARTICIPANT IS NOT SURE WHAT THE QUESTION MEANS, READ THIS EXAMPLE AS IT IS WRITTEN]   Nyengo zinyake munthu wakuwa wambula kusangwa panji wambula kunweka navinthu ivyo wakatemwanga kupanga ngati kuphika, kugwira tchito panji kuchezga na wabwezi, nangauli kale wakasanguluskikanga navinthu vyeneivyi. Nkhanandi uli ivi vyamuchitikiranipo mumasabata yawiri ayo yajumpha? |  | Read note if participant does not understand question. |
| *PROBSLEEP* | During the past two weeks, how much have you had trouble falling or staying asleep, or sleeping too much? | 0  1  2  3  6 | Not at all  Sometimes  Usually  Almost Always  Refused to answer | Mu masabata awiri apitawa, ndi mochuluka bwanji pamene mwakhala mukuvutika kupeza tulo kapena kugona mosakwanira, kapenanso kugona kwambiri? | Palibe  Nthawi zina  Kawirikawiri  Pafupifupi nthawi zonse  Refused to answer | Mu masabata yawiri ayo yajumpha, nkhanandi uli ako mwawa wa kutondeka kusanga tulo panji kugona mwambula kukwanira, panji kugona chomene? | Palije  Nyengo zinyakhe  Kawirikawiri  Pafupifupi nyengo zose  Refused to answer | Record as responded. Read out the options if not using the visual aid. |
| *PROBSLEEP_NOTE* | [IF THE PARTICIPANT IS NOT SURE WHAT THE QUESTION MEANS, READ THIS EXAMPLE AS IT IS WRITTEN]   Sometimes a person tries to go to sleep but cannot, or wakes often even if he/she is tired, or sleeps for longer than he/she would normally do. How much has this happened to you in the last 2 weeks? |  |  | [IF THE PARTICIPANT IS NOT SURE WHAT THE QUESTION MEANS, READ THIS EXAMPLE AS IT IS WRITTEN]  Nthawi zina munthu amayesera kugona koma tulo osabwera kapena kumangozizimuka pafupifupafupi angakhale iye ali wotopa, kapena kugona nthawi yaitali modabwitsa. Ndi mochuluka bwanji zimenezi zinakuchitikirani mu masabata awiri apitawa? |  | [IF THE PARTICIPANT IS NOT SURE WHAT THE QUESTION MEANS, READ THIS EXAMPLE AS IT IS WRITTEN]   Nyengo zinyake munthu wangakhumba kuti wakagone kweni nakusoba tulo, panji wakuwukawuka apo wagona nanga wangaba kuti wavuka, panji wakugona mwakujumphizga umo wakugonera nyengozose. Nkhanandi uli ivi vyamuchitikiranipo mumasabata yawiri ayo yajumpha? |  | Read note if participant does not understand question. |
| *TIRED* | During the past two weeks, how much have you been feeling tired, fatigued or having little energy? | 0  1  2  3  6 | Not at all  Sometimes  Usually  Almost Always  Refused to answer | Mu masabata awiri apitawa, ndi mochuluka bwanji pamene mwakhala mukumva kutopa, kufowoka kapena kuchepa mphamvu? | Palibe  Nthawi zina  Kawirikawiri  Pafupifupi nthawi zonse  Refused to answer | Mu masabata yawiri ayo yajumpha, nkhanandi uli ako mwawa wa kuvuka, wakulema, panji kuchepelwa nkhongono? | Palije  Nyengo zinyakhe  Kawirikawiri  Pafupifupi nyengo zose  Refused to answer | Record as responded. Read out the options if not using the visual aid. |
| *TIRED_NOTE* | [IF THE PARTICIPANT IS NOT SURE WHAT THE QUESTION MEANS, READ THIS EXAMPLE AS IT IS WRITTEN]    Sometimes a person feels particularly tired and weak and finds everything an effort to do, even if he/she has had rest. How much has this happened to you in the last 2 weeks? |  |  | [IF THE PARTICIPANT IS NOT SURE WHAT THE QUESTION MEANS, READ THIS EXAMPLE AS IT IS WRITTEN]  Nthawi zina munthu amakhala wotopa ndi wofooka okuti amapeza china chili chonse chakuti achite kukhala kulimbana nacho kuti amalizitse angakhale iye wapumula mokwanira. Ndi mochuluka bwanji zimenezi zinakuchitikirani mu masabata awiri apitawa? |  | [IF THE PARTICIPANT IS NOT SURE WHAT THE QUESTION MEANS, READ THIS EXAMPLE AS IT IS WRITTEN]    Nyengo zinyakhe munthu wakuwa wakuvuka nakufooka mwakuti wakusanga chilichose chakuti wachite chikuwa chinonono kumalizgiska, nanga wuli iyo wapumula mwakukwanila. Nkhanandi uli ivi vyamuchitikiranipo mumasabata yawiri ayo yajumpha? |  | Read note if participant does not understand question. |
| *POORAPP* | During the past two weeks, how much have you had poor appetite or been overeating? | 0  1  2  3  6 | Not at all  Sometimes  Usually  Almost Always  Refused to answer | Mu masabata awiri apitawa, ndi mochuluka bwanji pamene mwakhala osowa chilakolako cha chakudya kapena kumadya kwambiri? | Palibe  Nthawi zina  Kawirikawiri  Pafupifupi nthawi zonse  Refused to answer | Mu masabata yawiri ayo yajumpha, nkhanandi uli ako mwawa wa kuwavya khumbilo la chakurya panji kurya mwakujumphizga? | Palije  Nyengo zinyakhe  Kawirikawiri  Pafupifupi nyengo zose  Refused to answer | Record as responded. Read out the options if not using the visual aid. |
| *POORAPP_NOTE* | [IF THE PARTICIPANT IS NOT SURE WHAT THE QUESTION MEANS, READ THIS EXAMPLE AS IT IS WRITTEN]   Sometimes a person finds that, even though there is food available to eat at home, they are not interested in eating it. Or they may eat more than usual because of how they are feeling emotionally. How much has this happened to you in the last 2 weeks? |  |  | [IF THE PARTICIPANT IS NOT SURE WHAT THE QUESTION MEANS, READ THIS EXAMPLE AS IT IS WRITTEN]   Nthawi zina munthu amakhala opanda chilakolako choti adye angakhale chakudya chilipo panyumba. Kapenanso nthawi zina amadya kwambiri kuposa mmene achitira nthawi zonse chifukwa cha mmene akumvera mumtima. Ndi mochuluka bwanji zimenezi zinakuchitikirani mu masabata awiri apitawa? |  | [IF THE PARTICIPANT IS NOT SURE WHAT THE QUESTION MEANS, READ THIS EXAMPLE AS IT IS WRITTEN]   Nyengo zinyake munthu wangaba na vyakulya vyakuti walye panyumba, kweni wakusoba khumbiro lakuti walye. Panji wakulya mwakujumphizga mulingo wanyengozose chifukwa cha umo wakujipulikira mumtima. Nkhanandi uli ivi vyamuchitikiranipo mumasabata yawiri ayo yajumpha? |  | Read note if participant does not understand question. |
| *FEELBAD* | During the past two weeks, how much have you been feeling bad about yourself—or that you are a failure or have let yourself or your family down? | 0  1  2  3  6 | Not at all  Sometimes  Usually  Almost Always  Refused to answer | Mu masabata awiri apitawa, ndi mochuluka bwanji pamene mwakhala musakumva bwino mu mtima za inu mwini - kapena kuziona kuti ndinu olephera kapena kuzikhumudwitsa kapena kukhumudwitsa banja lanu? | Palibe  Nthawi zina  Kawirikawiri  Pafupifupi nthawi zonse  Refused to answer | Mu masabata yawiri ayo yajumpha, nkhanandi uli ako mwanguwa kwambula kujipulika makola mumtima vya imwe mwawene, mwajiwona kuti ndimwe wambula kwenelera, panji kuti muli wakutondeka, panji kuti mwajikhozga soni mwekha panji kukhozga soni banja linu? | Palije  Nyengo zinyakhe  Kawirikawiri  Pafupifupi nyengo zose  Refused to answer | Record as responded. Read out the options if not using the visual aid. |
| *FEELBAD_NOTE* | [IF THE PARTICIPANT IS NOT SURE WHAT THE QUESTION MEANS, READ THIS EXAMPLE AS IT IS WRITTEN]   Sometimes a person feels worthless, blames themselves for all their problems, or feels that they have disappointed people they care about. How much has this happened to you in the last 2 weeks? |  |  | [IF THE PARTICIPANT IS NOT SURE WHAT THE QUESTION MEANS, READ THIS EXAMPLE AS IT IS WRITTEN]   Nthawi zina munthu amakhala ndi maganizo akuti iye ndiwopanda phindu ndipo amaziloza chala iye mwini chifukwa cha mavuto ake onse, kapenanso amatha kukhala ndi maganizo akuti wakhumudwitsa anthu a pamtima pake. Ndi mochuluka bwanji zimenezi zinakuchitikirani mu masabata awiri apitawa? |  | [IF THE PARTICIPANT IS NOT SURE WHAT THE QUESTION MEANS, READ THIS EXAMPLE AS IT IS WRITTEN]    Nyengo zinyake munthu wakujiwona wambula phindu, kujitundura yekha pa masuzgo yose agho wakusangananayo, pakunji wakujipulika kuti wakhubazga banthu awo bakupweleleka na iyo. Nkhanandi uli ivi vyamuchitikiranipo mumasabata yawiri ayo yajumpha? |  | Read note if participant does not understand question. |
| *CONCENTRATE* | During the past two weeks, how much have you had trouble concentrating on things, such as reading, talking with friends, finishing tasks, or attending a meeting? | 0  1  2  3  6 | Not at all  Sometimes  Usually  Almost Always  Refused to answer | Mu masabata awiri apitawa, ndi mochuluka bwanji pamene mwakhala ovutika kukhazikitsa maganizo anu pa chinthu chimodzi monga kuwerenga, kucheza ndi anzanu, kumalizitsa ntchito imene mwayiyamba kapena kutenga mbali mu zokambirana? | Palibe  Nthawi zina  Kawirikawiri  Pafupifupi nthawi zonse  Refused to answer | Mu masabata yawiri ayo yajumpha, nkhanandi uli ako mwawa wakutondeka kuwika maghanoghano yinu ghamoza pavinthu panji kutondeka kufiska chinthu icho mwachiyamba kuchita ngati kuwerenga, kuyowoya na wabwezi, kumalizga zintchito, panji kutolapo lwande pa nkhumano? | Palije  Nyengo zinyakhe  Kawirikawiri  Pafupifupi nyengo zose  Refused to answer | Record as responded. Read out the options if not using the visual aid. |
| *CONCENTRATE_NOTE* | [IF THE PARTICIPANT IS NOT SURE WHAT THE QUESTION MEANS, READ THIS EXAMPLE AS IT IS WRITTEN]   Sometimes a person finds that they cannot complete tasks that they normally would because their mind wanders or they are thinking about other things. How much has this happened to you in the last 2 weeks? |  |  | [IF THE PARTICIPANT IS NOT SURE WHAT THE QUESTION MEANS, READ THIS EXAMPLE AS IT IS WRITTEN]   Nthawi zina munthu amapezeka kuti akulephera kumalizitsa ntchito zakuti m’mbuyomu amakwaniritsa chifukwa cholephera kukhazikitsa ma maganizo ake pamodzi kapenanso chifukwa chakuti akuganizira zinthu zina. Ndi mochuluka bwanji zimenezi zinakuchitikirani mu masabata awiri apitawa? |  | [IF THE PARTICIPANT IS NOT SURE WHAT THE QUESTION MEANS, READ THIS EXAMPLE AS IT IS WRITTEN]    Nyengo zinyake munthu wangatondeka kumaliziska milimo iyo nyengozose wakamalizganga makola waka chifukwa cha kutimbanizgika mukaghanaghaniro panji kutimbanizgika chifukwa chakughanaghana vinthu vinyake. Nkhanandi uli ivi vyamuchitikiranipo mumasabata yawiri ayo yajumpha? |  | Read note if participant does not understand question. |
| *SLOW* | During the past two weeks, how much have you been moving or speaking so slowly that other people could have noticed? | 0  1  2  3  6 | Not at all  Sometimes  Usually  Almost Always  Refused to answer | Mu masabata awiri apitawa, ndi mochuluka bwanji pamene mwakhala mukuyenda kapena kulankhula mwa pang’onopang’ono moti anthu ena anazindikira zimenezi? | Palibe  Nthawi zina  Kawirikawiri  Pafupifupi nthawi zonse  Refused to answer | Mu masabata yawiri ayo yajumpha, nkhanandi uli ako mwayendanga panji mwayowoyanga mwapachokopachoko chomene kuti mpaka wanthu wanyakhe bakamubowozgani? | Palije  Nyengo zinyakhe  Kawirikawiri  Pafupifupi nyengo zose  Refused to answer | Record as responded. Read out the options if not using the visual aid. |
| *SLOW_NOTE* | [IF THE PARTICIPANT IS NOT SURE WHAT THE QUESTION MEANS, READ THIS EXAMPLE AS IT IS WRITTEN]   Sometimes a person will walk or talk much more slowly than normal because of how they are feeling emotionally. How much has this happened to you in the last 2 weeks? |  |  | [IF THE PARTICIPANT IS NOT SURE WHAT THE QUESTION MEANS, READ THIS EXAMPLE AS IT IS WRITTEN]   Nthawi zina munthu amayenda kapena kuyankhula pang’onopang’ono chifukwa chammene akumvera mumtima. Ndi mochuluka bwanji zimenezi zinakuchitikirani mu masabata awiri apitawa? |  | [IF THE PARTICIPANT IS NOT SURE WHAT THE QUESTION MEANS, READ THIS EXAMPLE AS IT IS WRITTEN]   Nyengo zinyake munthu wangendanga panji kuyowoya mwapachokopachoko chomene kuluska nyengo zose chifukwa cha umo wakujipilikira mumtima. Nkhanandi uli ivi vyamuchitikiranipo mumasabata yawiri ayo yajumpha? |  | Read note if participant does not understand question. |
| *RESTLESSDEP* | During the past two weeks, how much have you been so fidgety or restless that you have been moving around a lot more than usual? | 0  1  2  3  6 | Not at all  Sometimes  Usually  Almost Always  Refused to answer | Mu masabata awiri apitawa, ndi mochuluka bwanji pamene mwakhala ndi mtima woti siunali mmalo mwakuti mwakhala mukumangoyendayenda koposa mmene mumachitira masiku onse? | Palibe  Nthawi zina  Kawirikawiri  Pafupifupi nthawi zonse  Refused to answer | Mu masabata yawiri ayo yajumpha, nkhanandi uli ako mtima winu ukawa mmalo mwakhe yaye mwakuti mwendendekanga mwakuluska nyengo zose? | Palije  Nyengo zinyakhe  Kawirikawiri  Pafupifupi nyengo zose  Refused to answer | Record as responded. Read out the options if not using the visual aid. |
| *RESTLESSDEP_NOTE* | [IF THE PARTICIPANT IS NOT SURE WHAT THE QUESTION MEANS, READ THIS EXAMPLE AS IT IS WRITTEN]   Sometimes a person is so worried or sad that they cannot relax and they move about much more than usual. How much has this happened to you in the last 2 weeks? |  |  | [IF THE PARTICIPANT IS NOT SURE WHAT THE QUESTION MEANS, READ THIS EXAMPLE AS IT IS WRITTEN]   Nthawi zina munthu amakhala` wodandaula kapena wokhumudwa kwambiri ndipo mtima wake siukhala mmalo motero kuti amangoyendayenda/amangoti zungulizunguli. Ndi mochuluka bwanji zimenezi zinakuchitikirani mu masabata awiri apitawa? |  | [IF THE PARTICIPANT IS NOT SURE WHAT THE QUESTION MEANS, READ THIS EXAMPLE AS IT IS WRITTEN]    Nyengo zinyake munthu wakudandaula panji kuba na chitima mwakuti wakutondeka kufwasa kweniso kwendendekanga chomene kuluska nyengo zose. Nkhanandi uli ivi vyamuchitikiranipo mumasabata yawiri ayo yajumpha? |  | Read note if participant does not understand question. |
| *SUICIDE* | During the past two weeks, how much have you had thoughts that you would be better off dead or of hurting yourself in some way? | 0  1  2  3  6 | Not at all  Sometimes  Usually  Almost Always  Refused to answer | Mu masabata awiri apitawa, ndi mochuluka bwanji pamene mwakhala ndi maganizo akuti kuli bwino mungofa kapena mungozivulaza nokha mwanjira ina yake? | Palibe  Nthawi zina  Kawirikawiri  Pafupifupi nthawi zonse  Refused to answer | Mu masabata yawiri ayo yajumpha, nkhanandi uli ako mwawa na maghanoghano ghakuti chingawa makola kufwa panji kujipweteka mwekha mu nthowa yinyakhe? | Palije  Nyengo zinyakhe  Kawirikawiri  Pafupifupi nyengo zose  Refused to answer | Record as responded. Read out the options if not using the visual aid. |
| *SUICIDE_NOTE* | [IF THE PARTICIPANT IS NOT SURE WHAT THE QUESTION MEANS, READ THIS EXAMPLE AS IT IS WRITTEN]   Sometimes a person feels so down that he/she has thoughts that life is no longer worth living and he/she thinks about ending his/her life or hurting him/herself for example using poison or hanging. How much has this happened to you in the last 2 weeks? |  |  | [IF THE PARTICIPANT IS NOT SURE WHAT THE QUESTION MEANS, READ THIS EXAMPLE AS IT IS WRITTEN]   Nthawi zina munthu amakhala osasangalala ndipo amakhala ndi maganizo akuti moyo ulibe phindu ndipo amaganizanso zozipha kapena zozivulaza iye mwini mwachitsanzo pogwiritsa ntchito poyizoni kapena kuzikhweza/kuzimangilirira. Ndi mochuluka bwanji zimenezi zinakuchitikirani mu masabata awiri apitawa? |  | [IF THE PARTICIPANT IS NOT SURE WHAT THE QUESTION MEANS, READ THIS EXAMPLE AS IT IS WRITTEN]  Nyengo zinyake munthu wangaba wambula kukondwa ndipo akuba na maghanoghano yakuti moyo wakhe ngwambula kwenelera panji kubaso namaghanoghano yakujipweteka/kujikoma pakugwiriska ntchito vinthu ngati poyizoni panji kujikaka. Nkhanandi uli ivi vyamuchitikiranipo mumasabata yawiri ayo yajumpha? |  | Read note if participant does not understand question. |
| *DIFFPROBSNOTE* | [DO NOT USE THE VISUAL AID FOR THIS QUESTION] |  |  | [DO NOT USE THE VISUAL AID FOR THIS QUESTION] |  | [DO NOT USE THE VISUAL AID FOR THIS QUESTION] |  |  |
| *DIFFICULTYPROBS* | In the last 2 weeks, how difficult have these problems made it for you to do your work, take care of things at home or get along with other people [READ THE OPTIONS]? | 0  1  2  3  6 | Not difficult at all  Somewhat difficult  Very difficult  Extremely difficult  Refused to answer | Mu masabata awiri apitawa, kodi mabvuto amenewa alimbitsa bwanji kagwiridwe kanu ka ntchito, kasamaliridwe kanu ka zinthu pakhomo, kapena ubale wanu ndi anthu: [READ THE OPTIONS]? | Sindinavutikepo  Pang’ono pokha  Mochuluka  Mochuluka kwambiri  Refused to answer | Mu masabata yawiri ayo yajumpha, kasi masuzgo aya yiza na maunonono wuli pa umo mukugwirira ntchito, kupwererera vinthu pa nyumba, panji ubale winu na banthu: [READ THE OPTIONS]? | Unonono pakawavya  Pachoko waka  Chomene  Chomene nkhanila  Refused to answer | Do not use the visual aid for this question. |
| *DIFFICULTYPROBS_NOTE* | [IF THE PARTICIPANT IS NOT SURE WHAT THE QUESTION MEANS, READ THIS EXAMPLE AS IT IS WRITTEN]    Sometimes when a person has problems like feeling depressed, tired or hopeless, the person cannot do their usual activities. For you, in the last 2 weeks, how difficult have you found it to do your normal activities: Not difficult at all; Somewhat difficult; Very difficult or Extremely difficult? |  |  | [IF THE PARTICIPANT IS NOT SURE WHAT THE QUESTION MEANS, READ THIS EXAMPLE AS IT IS WRITTEN]   Nthawi zina munthu amakhala ndi mavuto monga kukhumudwa, kutopa kapenanso kupanda chiyembekezo, mwakuti munthuyo sangathe kuchita ntchito za moyo wake wa tsiku ndi tsiku. Inuyo mu masabata awiri apitawa, zimakuvutani bwanji kuchita ntchito za moyo wanu wa tsiku ndi tsiku: Sindinavutikepo; Pang’ono pokha; Mochuluka; Mochuluka kwambiri? |  | [IF THE PARTICIPANT IS NOT SURE WHAT THE QUESTION MEANS, READ THIS EXAMPLE AS IT IS WRITTEN]   Nyengo zinyake pala munthu wanamasuzgo ngati kuba wakukwebeka, wakuvuka panji wambula chigomezgo, wangatondeka kukwaniska kugwira tchito izo wakakwaniskanga nyengo zose. Kasi imwe, mumasabata yawiri ayo yajumpha, mwasanga maunonono uli ayo yamutondeskani kugwira ntchito ngati umo mukuchitira nyengo zose: Unonono pakawavya; Pachoko waka; Chomene; Chomene nkhanila? |  |  |
| ***GAD7*** | **GAD7** |  |  | **GAD7** |  | **GAD7** |  |  |
| *NERVOUS* | Over the last two weeks, how much have you been feeling nervous, anxious, or on edge? | 0  1  2  3  6 | Not at all  Sometimes  Usually  Almost Always  Refused to answer | Mu masabata awiri apitawa, ndi mochuluka bwanji pamene mu mtima mwanu mwakhala ndimantha, nkhawa, kapena kusowa mtendele? | Palibe  Nthawi zina  Kawirikawiri  Pafupifupi nthawi zonse  Refused to answer | Mu masabata yawiri ayo yajumpha, nkhanandi uli ako mu mtima mwinu mwanguwa na wofi, wa kudandaula, panji wambula mtende? | Palije  Nyengo zinyakhe  Kawirikawiri  Pafupifupi nyengo zose  Refused to answer | Record as responded. Read out the options if not using the visual aid. |
| *NERVOUS_NOTE* | [IF THE PARTICIPANT IS NOT SURE WHAT THE QUESTION MEANS, READ THIS EXAMPLE AS IT IS WRITTEN]    Sometimes a person feels fearful even if there is no danger, or worries that something bad will happen to him/herself or family? How much has this happened to you in the last 2 weeks? |  |  | [IF THE PARTICIPANT IS NOT SURE WHAT THE QUESTION MEANS, READ THIS EXAMPLE AS IT IS WRITTEN]   Nthawi zina munthu amakhala ndi mantha ngakhale palibe choopsa chilichonse, kapenanso amakhala wodandaula kuti china chake choyipa chimuchitikira iye kapena banja lake. Ndi mochuluka bwanji zimenezi zinakuchitikirani mu masabata awiri apitawa? |  | [IF THE PARTICIPANT IS NOT SURE WHAT THE QUESTION MEANS, READ THIS EXAMPLE AS IT IS WRITTEN]    Nyengo zinyake munthu wakuba na wofi kwambula chakofya chilichose panji kudandaula kuti viheni vitimuchitikilenge iyo panji bapabanja lakhe? Nkhanandi uli ivi vyamuchitikiranipo mumasabata yawiri ayo yajumpha? |  | Read note if participant does not understand question. |
| *WORRYING* | Over the last two weeks, how much have you not been able to stop or control worrying? | 0  1  2  3  6 | Not at all  Sometimes  Usually  Almost Always  Refused to answer | Mu masabata awiri apitawa, ndi mochuluka bwanji pamene mwakhala olephera kusiya kapena kuchepetsa kudandaula? | Palibe  Nthawi zina  Kawirikawiri  Pafupifupi nthawi zonse  Refused to answer | Mu masabata yawiri ayo yajumpha, nkhanandi uli ako mwawa wa kutondeka kuleka panji kuchepeska kudandaula? | Palije  Nyengo zinyakhe  Kawirikawiri  Pafupifupi nyengo zose  Refused to answer | Record as responded. Read out the options if not using the visual aid. |
| *WORRYING_NOTE* | [IF THE PARTICIPANT IS NOT SURE WHAT THE QUESTION MEANS, READ THIS EXAMPLE AS IT IS WRITTEN]   Sometimes a person cannot stop having worrying thoughts even if he/she normally could do so. How much has this happened to you in the last 2 weeks? |  |  | [IF THE PARTICIPANT IS NOT SURE WHAT THE QUESTION MEANS, READ THIS EXAMPLE AS IT IS WRITTEN]   Nthawi zina munthu amalephera kusiya kukhala ndi maganizo odandaula ngakhale iye ali ndi kuthekera kosiya kudandaulako. Ndi mochuluka bwanji zimenezi zinakuchitikirani mu masabata awiri apitawa? |  | [IF THE PARTICIPANT IS NOT SURE WHAT THE QUESTION MEANS, READ THIS EXAMPLE AS IT IS WRITTEN]   Nyengo zinyake munthu wakutondeka kujilekeska maghanoghano gha kudandaula nanga wangayezeska theula. Nkhanandi uli ivi vyamuchitikiranipo mumasabata yawiri ayo yajumpha? |  | Read note if participant does not understand question. |
| *WORRYDIFF* | Over the last two weeks, how much have you been worrying too much about different things? | 0  1  2  3  6 | Not at all  Sometimes  Usually  Almost Always  Refused to answer | Mu masabata awiri apitawa, ndi mochuluka bwanji mwakhala odandaula kwambiri za zinthu zosiyanasiyana? | Palibe  Nthawi zina  Kawirikawiri  Pafupifupi nthawi zonse  Refused to answer | Mu masabata yawiri ayo yajumpha, nkhanandi uli ako mwanguwa wakudandaula mwakujumphizga kukhwaskana na vinthu vyakupambanapambana? | Palije  Nyengo zinyakhe  Kawirikawiri  Pafupifupi nyengo zose  Refused to answer | Record as responded. Read out the options if not using the visual aid. |
| *WORRYDIFF_NOTE* | [IF THE PARTICIPANT IS NOT SURE WHAT THE QUESTION MEANS, READ THIS EXAMPLE AS IT IS WRITTEN]   Sometimes a person worries about many things such as family, health, money, future plans, all at the same time. How much has this happened to you in the last 2 weeks? |  |  | [IF THE PARTICIPANT IS NOT SURE WHAT THE QUESTION MEANS, READ THIS EXAMPLE AS IT IS WRITTEN]   Nthawi zina munthu amadandaula za zinthu zambiri monga banja, umoyo, ndalama, mapulani amtsogolo, zonsezi panthawi imodzi. Ndi mochuluka bwanji zimenezi zinakuchitikirani mu masabata awiri apitawa? |  | [IF THE PARTICIPANT IS NOT SURE WHAT THE QUESTION MEANS, READ THIS EXAMPLE AS IT IS WRITTEN]   Nyengo zinyake munthu wakudandaula kukhwaskana na vinthu vinadi ngati banja, moyo, ndalama, mapulani yamunthazi kweni vyose ivyi panyengo yimoza. Nkhanandi uli ivi vyamuchitikiranipo mumasabata yawiri ayo yajumpha? |  | Read note if participant does not understand question. |
| *PROBRELAX* | Over the last two weeks, how much have you had trouble relaxing? | 0  1  2  3  6 | Not at all  Sometimes  Usually  Almost Always  Refused to answer | Mu masabata awiri apitawa, ndi mochuluka bwanji mwakhala mukuvutika kuti mukhale omasuka? | Palibe  Nthawi zina  Kawirikawiri  Pafupifupi nthawi zonse  Refused to answer | Mu masabata yawiri ayo yajumpha, nkhanandi uli ako mwanguwa wambula kufwasa mu mtima? | Palije  Nyengo zinyakhe  Kawirikawiri  Pafupifupi nyengo zose  Refused to answer | Record as responded. Read out the options if not using the visual aid. |
| *PROBRELAX_NOTE* | [IF THE PARTICIPANT IS NOT SURE WHAT THE QUESTION MEANS, READ THIS EXAMPLE AS IT IS WRITTEN]   Sometimes a person cannot rest even though they do not have work to do. How much has this happened to you in the last 2 weeks? |  |  | [IF THE PARTICIPANT IS NOT SURE WHAT THE QUESTION MEANS, READ THIS EXAMPLE AS IT IS WRITTEN]   Nthawi zina munthu amalephera kukhazikika ngakhale palibe ntchito yomutanganidwitsa. Ndi mochuluka bwanji zimenezi zinakuchitikirani mu masabata awiri apitawa? |  | [IF THE PARTICIPANT IS NOT SURE WHAT THE QUESTION MEANS, READ THIS EXAMPLE AS IT IS WRITTEN]   Nyengo zinyake munthu wakutondeka kujipumuzga nanga pangaba kuti palije ntchito iyo bakugwira. Nkhanandi uli ivi vyamuchitikiranipo mumasabata yawiri ayo yajumpha? |  | Read note if participant does not understand question. |
| *RESTLESSANX* | Over the last two weeks, how much have you been so restless that it is hard to sit still? | 0  1  2  3  6 | Not at all  Sometimes  Usually  Almost Always  Refused to answer | Mu ma sabata awiri apitawa, ndi mochuluka bwanji pamene mtima wanu siunali mmalo mwake mwakuti mumalephera kukhazikika malo amodzi? | Palibe  Nthawi zina  Kawirikawiri  Pafupifupi nthawi zonse  Refused to answer | Mu masabata yawiri ayo yajumpha, nkhanandi uli ako mtima winu wanguwa mmalo mwakhe yayi mwakuti mwatondekanga kuti mukhazikike pa malo yamoza? | Palije  Nyengo zinyakhe  Kawirikawiri  Pafupifupi nyengo zose  Refused to answer | Record as responded. Read out the options if not using the visual aid. |
| *RESTLESSANX_NOTE* | [IF THE PARTICIPANT IS NOT SURE WHAT THE QUESTION MEANS, READ THIS EXAMPLE AS IT IS WRITTEN]  Sometimes a person is so worried or sad that they cannot relax and they move about much more than usual. How much has this happened to you in the last 2 weeks? |  |  | [IF THE PARTICIPANT IS NOT SURE WHAT THE QUESTION MEANS, READ THIS EXAMPLE AS IT IS WRITTEN]   Nthawi zina munthu amakhala` wodandaula kapena wokhumudwa kwambiri ndipo mtima wake siukhala mmalo motero kuti amangoyendayenda/amangoti zungulizunguli. Ndi mochuluka bwanji zimenezi zinakuchitikirani mu masabata awiri apitawa? |  | [IF THE PARTICIPANT IS NOT SURE WHAT THE QUESTION MEANS, READ THIS EXAMPLE AS IT IS WRITTEN]   Nyengo zinyake munthu wakudandaula panji kuba na chitima mwakuti wakutondeka kufwasa kweniso kwendendekanga chomene kuluska nyengo zose. Nkhanandi uli ivi vyamuchitikiranipo mumasabata yawiri ayo yajumpha? |  | Read note if participant does not understand question. |
| *IRRITABLE* | Over the last two weeks, how much have you felt annoyed or irritable? | 0  1  2  3  6 | Not at all  Sometimes  Usually  Almost Always  Refused to answer | Mu ma sabata awiri apitawa, ndi mochuluka bwanji mwakhala wokwiya mosavuta? | Palibe  Nthawi zina  Kawirikawiri  Pafupifupi nthawi zonse  Refused to answer | Mu masabata yawiri ayo yajumpha, nkhanandi uli ako mwakalipanga panji mwakwiyanga mwaluwiro? | Palije  Nyengo zinyakhe  Kawirikawiri  Pafupifupi nyengo zose  Refused to answer | Record as responded. Read out the options if not using the visual aid. |
| *IRRITABLE_NOTE* | [IF THE PARTICIPANT IS NOT SURE WHAT THE QUESTION MEANS, READ THIS EXAMPLE AS IT IS WRITTEN]   Sometimes a person is easily angered by small actions of their family or friends. How much has this happened to you in the last 2 weeks? |  |  | [IF THE PARTICIPANT IS NOT SURE WHAT THE QUESTION MEANS, READ THIS EXAMPLE AS IT IS WRITTEN]   Nthawi zina munthu sachedwa kukwiyisidwa ndi tizinthu ting’onoting’ono tomwe banja lake kapena anzake achita. Ndi mochuluka bwanji zimenezi zinakuchitikirani mu masabata awiri apitawa? |  | [IF THE PARTICIPANT IS NOT SURE WHAT THE QUESTION MEANS, READ THIS EXAMPLE AS IT IS WRITTEN]   Nyengo zinyake munthu wakukwiyanga lubilo na vichito vya banja lakhe panji wabwezi. Nkhanandi uli ivi vyamuchitikiranipo mumasabata yawiri ayo yajumpha? |  | Read note if participant does not understand question. |
| *AFRAID* | Over the last two weeks, how much have you felt afraid as if something awful might happen? | 0  1  2  3  6 | Not at all  Sometimes  Usually  Almost Always  Refused to answer | Mu ma sabata awiri apitawa, ndi mochuluka bwanji pamene mwakhala ndi mantha mu mtima mwanu kuti chinachake choopsa chikhoza kuchitika? | Palibe  Nthawi zina  Kawirikawiri  Pafupifupi nthawi zonse  Refused to answer | Mu masabata yawiri ayo yajumpha, nkhanandi uli ako mwapulika wofi mu mtima nganipala chinyakhe chakofya chingachitika? | Palije  Nyengo zinyakhe  Kawirikawiri  Pafupifupi nyengo zose  Refused to answer | Record as responded. Read out the options if not using the visual aid. |
| *AFRAID_NOTE* | [IF THE PARTICIPANT IS NOT SURE WHAT THE QUESTION MEANS, READ THIS EXAMPLE AS IT IS WRITTEN]   Sometimes a person feels fearful that something bad will happen to him/herself or family? How much has this happened to you in the last 2 weeks? |  |  | [IF THE PARTICIPANT IS NOT SURE WHAT THE QUESTION MEANS, READ THIS EXAMPLE AS IT IS WRITTEN]   Nthawi zina munthu amakhala ndi mantha kuti chinthu china chake choyipa chimuchitikira iye mwini kapenanso banja lake. Ndi mochuluka bwanji zimenezi zinakuchitikirani mu masabata awiri apitawa? |  | [IF THE PARTICIPANT IS NOT SURE WHAT THE QUESTION MEANS, READ THIS EXAMPLE AS IT IS WRITTEN]   Nyengo zinyake munthu wakuba nawofyi kuti chinthu chinyake chiheni chitimuchitikilenge iyo panji banja lakhe. Nkhanandi uli ivi vyamuchitikiranipo mumasabata yawiri ayo yajumpha? |  | Read note if participant does not understand question. |
| ***SOMATIC*** | **Somatic items** |  |  | **Somatic items** |  | **Somatic items** |  |  |
| *HEADACHE* | During the past 2 weeks, how much have you had a headache? | 0  1  2  3  6 | Not at all  Sometimes  Usually  Almost Always  Refused to answer | Mu masabata awiri apitawa, ndi mochuluka bwanji mwakhala mukumva kupweteka mutu? | Palibe  Nthawi zina  Kawirikawiri  Pafupifupi nthawi zonse  Refused to answer | Mu masabata yawiri ayo yajumpha, nkhanandi uli ako mwapulika kuwawa kwa mutu? | Palije  Nyengo zinyakhe  Kawirikawiri  Pafupifupi nyengo zose  Refused to answer | Record as responded. Read out the options if not using the visual aid. |
| *STOMACHPROB* | During the past 2 weeks, how much have you had stomach problems? | 0  1  2  3  6 | Not at all  Sometimes  Usually  Almost Always  Refused to answer | Mu masabata awiri apitawa, ndi mochuluka bwanji mwakhala ndi mavuto a mmimba? | Palibe  Nthawi zina  Kawirikawiri  Pafupifupi nthawi zonse  Refused to answer | Mu masabata yawiri ayo yajumpha, nkhanandi uli ako mwawa na masugzo ya mnthumbo? | Palije  Nyengo zinyakhe  Kawirikawiri  Pafupifupi nyengo zose  Refused to answer | Record as responded. Read out the options if not using the visual aid. |
| *BODYPAINS* | During the past 2 weeks, how much have you had general body pains? | 0  1  2  3  6 | Not at all  Sometimes  Usually  Almost Always  Refused to answer | Mu masabata awiri apitawa, ndi mochuluka bwanji mwakhala ndi vuto lakuphwanya kwa mthupi? | Palibe  Nthawi zina  Kawirikawiri  Pafupifupi nthawi zonse  Refused to answer | Mu masabata yawiri ayo yajumpha, nkhanandi uli ako mwawa na masuzgo ya kuphwanya mthupi? | Palije  Nyengo zinyakhe  Kawirikawiri  Pafupifupi nyengo zose  Refused to answer | Record as responded. Read out the options if not using the visual aid. |
| ***PC-PTSD*** | **PC-PTSD** |  |  | **PC-PTSD** |  | **PC-PTSD** |  |  |
| *EVNTSLST* | [STOP USING VISUAL AID]   Sometimes things happen to people that are unusually or especially frightening, horrible, or traumatic. For example:   • a serious accident or fire • a physical or sexual assault or abuse • [WOMEN ONLY] a very difficult childbirth when you thought that you or the baby would die • an earthquake or flood • a war • seeing someone be killed or seriously injured • having a loved one die through homicide or suicide |  |  | [STOP USING VISUAL AID]   Nthawi zina zinthu zodabwitsa, zoopsya kwambiri, zoyipa, komanso zowawitsa mtima zimawachitikira anthu. Zinthu monga: • Ngozi yowopsya kapena moto • Kumenyedwa, nkhanza zogonana kapena nkhanza ya mtundu wina uliwonse • [WOMEN ONLY] nthawi yovuta kwambiri yakubereka yomwe munaganizirapo kuti inu kapena mwana wanu amwalira • chivomerezi kapena kusefukira kwa madzi • Nkhondo • kuwona winawake akuphedwa kapena atavulara kwambiri • kukhala ndi wachibale yemwe anazipha yekha kapena kuphedwa ndi anthu ena |  | [STOP USING VISUAL AID]   Nyengo zinyakhe vinthu vyakuziziswa, vyakofya chomene, viheni, kweniso vyakuwawiska mtima vikuwachitikira wanthu. Vinthu nge:  • Ngozi yakofya panji moto • Kutimbika panji kukorereka panji nkhaza zakugonana na nkhanza zinyakhe • [WOMEN ONLY] kusuzgika chomene pa nyengo ya kubaba apo mukaghanaghana kuti imwe panji mwana winu wafwenge • chindindindi panji kusefukira kwa maji • nkhondo • Kuona munthu wakukomeka panji wakupwetekeka chomene • Kuwa na m'bale wakuti walikujikoma yekha panji kukomeka na wanthu wanyakhe |  | These questions ask about past trauma. Take the visual aid from the participant. Read out the introductory speech as written. |
| *EXPEVNT* | Have you ever experienced this kind of traumatic event? | 1  0  8  6 | Yes  No  Don't Know  Refused to answer | Kodi zinthu monga izi zinakuchitikiranipo? | Eya  Ayi  Sindikudziwa  Refused to answer | Kasi vinthu nge ivi vilikumchitikiranipo? | Enya  Yayi  Nkhumanya yayi  Refused to answer | Record as responded. |
| *EVNTSNT_LABELS* | What traumatic event or events happened to you? | 1  0  8  6 | Yes  No  Don't Know  Refused to answer | Kodi zinachitika ndi chani? | Eya  Ayi  Sindikudziwa  Refused to answer | Kasi chikachitika ntchivichi? | Enya  Yayi  Nkhumanya yayi  Refused to answer | This question is asked if the participant has experienced trauma before. Ask and select from the list of questions below. |
| *ACCFIRE* | a serious accident or fire | 1  0  8  6 | Yes  No  Don't Know  Refused to answer | ngozi yowopsya kapena moto | Eya  Ayi  Sindikudziwa  Refused to answer | ngozi yakofya panji moto | Enya  Yayi  Nkhumanya yayi  Refused to answer | Record as responded. |
| *ABUSE* | a physical or sexual assault or abuse | 1  0  8  6 | Yes  No  Don't Know  Refused to answer | kumenyedwa, nkhanza zogonana kapena nkhanza ya mtundu wina uliwonse | Eya  Ayi  Sindikudziwa  Refused to answer | kutimbika, panji nkhaza zakugonana na nkhanza zinyakhe | Enya  Yayi  Nkhumanya yayi  Refused to answer | Record as responded. |
| *DIFBRTH* | a very difficult childbirth when you thought that you or the baby would die | 1  0  8  6 | Yes  No  Don't Know  Refused to answer | nthawi yovuta kwambiri yakubereka yomwe munaganizirapo kuti inu kapena mwana wanu amwalira | Eya  Ayi  Sindikudziwa  Refused to answer | kusuzgika chomene pa nyengo ya kubaba apo mukaghanaghana kuti imwe panji mwana winu wafwenge | Enya  Yayi  Nkhumanya yayi  Refused to answer | Record as responded. |
| *EQUAKFLD* | an earthquake or flood | 1  0  8  6 | Yes  No  Don't Know  Refused to answer | chivomerezi kapena kusefukira kwa madzi | Eya  Ayi  Sindikudziwa  Refused to answer | chindindindi panji kusefukira kwa maji | Enya  Yayi  Nkhumanya yayi  Refused to answer | Record as responded. |
| *WAR* | a war | 1  0  8  6 | Yes  No  Don't Know  Refused to answer | nkhondo | Eya  Ayi  Sindikudziwa  Refused to answer | nkhondo | Enya  Yayi  Nkhumanya yayi  Refused to answer | Record as responded. |
| *KILLEDINJUR* | seeing someone be killed or seriously injured | 1  0  8  6 | Yes  No  Don't Know  Refused to answer | kuwona winawake akuphedwa kapena atavulara kwambiri | Eya  Ayi  Sindikudziwa  Refused to answer | kuwona munthu wakukomeka panji wakupwetekeka chomene | Enya  Yayi  Nkhumanya yayi  Refused to answer | Record as responded. |
| *HSCIDE* | having a loved one die through homicide or suicide | 1  0  8  6 | Yes  No  Don't Know  Refused to answer | kukhala ndi wachibale yemwe anazipha yekha kapena kuphedwa ndi anthu ena | Eya  Ayi  Sindikudziwa  Refused to answer | Kuwa na m’bale wakuti walikujikoma yekha panji kukomeka na wanthu wanyakhe | Enya  Yayi  Nkhumanya yayi  Refused to answer | Record as responded. |
| *EVNTOTH* | other | 1  0  8  6 | Yes  No  Don't Know  Refused to answer | zina | Eya  Ayi  Sindikudziwa  Refused to answer | vinyakhe | Enya  Yayi  Nkhumanya yayi  Refused to answer | Record if other. |
| *EVNTOTHSPEC* | other, specify |  |  | ngati pali zina tchulani |  | vinyakhe longosolani |  | Specify if other. |
| ***EVENTS*** |  |  |  |  |  |  |  | The following questions are asked if a participant has experienced any traumatic event from the previous section. |
| *NIGHTMRS* | In the past month, have you had nightmares about the event(s) or thought about the event(s) when you did not want to? | 1  0  8  6 | Yes  No  Don't Know  Refused to answer | Mu mwezi wathawu, mwakhalapo ndi maloto kapena maganizo oyipa komanso obwerezabwereza okhudzana ndi zomwe zinakuchitikirani ngakhale inu simumafuna kuganizira zimenezo? | Eya  Ayi  Sindikudziwa  Refused to answer | Mu mwezi wajumpha uwu, mwakhalapo na maloto panji maghanoghano yaheni ya icho/ivyo vikamuchitikirani nanga uli imwe mukukhumba yayi kuvighanaghanira? | Enya  Yayi  Nkhumanya yayi  Refused to answer | Record as responded. |
| *AVOID* | In the past month, have you tried hard not to think about the event(s) or went out of your way to avoid situations that reminded you of the event(s)? | 1  0  8  6 | Yes  No  Don't Know  Refused to answer | Mu mwezi wathawu, mwakhala mukudziletsa kwambiri kuganizira zomwe zinachitika kapena kuyesayesa kuzemba zinthu, malo, kapena zochitika zomwe zingakukumbutseni za zomwe zinakuchitikirani? | Eya  Ayi  Sindikudziwa  Refused to answer | Mu mwezi wajumpha uwu, mwayezgapo chomene kujikanizga kughanaghanira vya ivyo vikachitika panji kuyezgeska kugwentha malo panji vinthu ivyo vikumukumbuskani ivo vikachitika? | Enya  Yayi  Nkhumanya yayi  Refused to answer | Record as responded. |
| *ONGRD* | In the past month, have you been constantly on guard, watchful, or easily startled? | 1  0  8  6 | Yes  No  Don't Know  Refused to answer | Mu mwezi wathawu, ndikawirikawiri mwakhala ndi mantha, tcheru ngati chinachake chichitika, kapena odzidzimutsika mosavuta? | Eya  Ayi  Sindikudziwa  Refused to answer | Mu mwezi wajumpha uwu, mwakhala wa tcheru chomene kweniso wakuchiluskika mwaluwiro? | Enya  Yayi  Nkhumanya yayi  Refused to answer | Record as responded. |
| *NUMB* | In the past month, have you felt numb or detached from people, activities, or your surroundings? | 1  0  8  6 | Yes  No  Don't Know  Refused to answer | Mu mwezi wathawu, mwakhala mukumva mumtima mwanu ngati kuti palibe kulumikizana ndi anthu, zochitika, kepana malo omwe muli? | Eya  Ayi  Sindikudziwa  Refused to answer | Mu mwezi wajumpha uwu, mwapulikapo ngati kuti thupi lati waka zii kweniso kupulika ngati mwatalikirana na wanthu, vakuchitika vya mdera linu, kweniso malo ayo mukukhala? | Enya  Yayi  Nkhumanya yayi  Refused to answer | Record as responded. |
| *GUILTY* | In the past month, have you felt guilty or unable to stop blaming yourself or others for the event(s) or any problems the event(s) may have caused? | 1  0  8  6 | Yes  No  Don't Know  Refused to answer | Mu mwezi wathawu, mwakhala mu kuziona olakwa komanso kukanika kusiya kuziona inu kapena ena kuti ndi olakwa chifukwa cha zomwe zinakuchitikirani kapena mavuto amene abwera chifukwa cha zimenezi? | Eya  Ayi  Sindikudziwa  Refused to answer | Mu mwezi wajumpha uwu, mwakhala mukujiyeruzga panji kutondeka kuleka kujiyeruzga mwawene panji wanthu wanyakhe chifukwa cha ivyo vikachitika panji masuzgo ayo yiza chifukwa cha ivi? | Enya  Yayi  Nkhumanya yayi  Refused to answer | Record as responded. |
| ***SUICIDESCREEN*** | **Suicide Screening** |  |  |  |  |  |  |  |
| *SUICIDERISKNOTE* | Read the following to participant: You said that, in the last two weeks, you have had thoughts that life is not worth living or of hurting yourself in some way. I am sorry to hear that. I would like to ask you a few more questions about those thoughts. |  |  | Read the following to participant: Munanena kuti mu sabata ziwiri zapitazi, mwakhalapo ndi maganizo akuti kulibwino mungofa kapena ofuna kuzivulaza nokha munjira ina yake. Ndine okhudzidwa kumva choncho. Ndikufuna ndikufunseni mafunso ena ochepa okhudza maganizo amenewa. |  | Read the following to participant: Mwanguyowoya kuti mu sabata ziwiri zajumpha, mwawapo na maghanoghano yakuti chingaba makola kufwa panji kujipweteka mwekha mu nthowa yinyakhe. Vyanikhwaska kuti vili nthena. Nkhukhumba kumufumbani mafumbo yanyakhe ghachoko ghakukhwaskana na maghanoghano agha. |  | These questions aim to screen participants at risk of suicide. Suicide screening is done for participants who have responded to have had thoughts that they would be better off dead or thoughts of hurting themselves in some way. Read the note to the participant as in the script. |
| *SUICIDERISK1* | Can I check, in the last 2 weeks have you had these thoughts many times or just once or twice? | 1  0  6 | Many times  Once or twice  Refused to answer | Mu masabata awiri apitawa, maganizowa mwakhala nawo kangati: kambiri, kapena "kamodzi kapena kawiri"? | Many times  Once or twice  Refused to answer | Mu masabata yaŵiri ayo yajumpha, kasi maghanoghano aya mwawanagho kalinga: kanandi, panji "kamoza panji kawiri"? | Many times  Once or twice  Refused to answer | Record as responded. |
| *SUICIDERISK2* | In the last 2 weeks, have you made any plans or considered a method that you might use to end your life or harm yourself? | 1  0  6 | Yes  No  Refused to answer | Mu masabata awiri apitawa, munapangako dongosolo kapena kulingalira njira yofuna kuzipha kapena kuzivulaza? | Eya  Ayi  Refused to answer | Mu masabata yaŵiri ayo yajumpha, kasi muli kupangapo nthowa yiliyose yakujikomera panji kughanaghana nthowa iyo mungagwiriska ntchito kuti mujipweteke mwaŵene? | Enya  Yayi  Refused to answer | Record as responded. |
| *SUICIDERISK3* | Have you ever attempted to harm yourself or end your life? | 1  0  6 | Yes  No  Refused to answer | Munayamba mwayeserapo kuzipweteka nokha kapena kufuna kuzipha nokha? | Eya  Ayi  Refused to answer | Kasi mulikuyezgapo kujipweteka mwabene panji kujikoma mwekha? | Enya  Yayi  Refused to answer | Record as responded. |
| *SUICIDERISK4* | Do you think there is any risk that you might harm yourself or end your life now or in the near future? | 1  0  6 | Yes  No  Refused to answer | Kodi mukuwona ngati muli pachiwopsyezo choti mutha kuzipha kapena kuzivulaza nokha pakali pano kapena posachedwapa? | Eya  Ayi  Refused to answer | Kasi mukughanaghana kuti ulipo ulwani wakuti mungajikoma panji mungajipweteka mwabene pa nyengo yasono panji mwasonosono? | Enya  Yayi  Refused to answer | Record as responded. |
| *CONTINT* | Read the following to the participant: Because you have had some thoughts that you would be better off dead or of hurting yourself in some way, at the end of the questionnaire, I will explain how you can get help and support. Do you feel able to continue with the rest of the interview first? | 1  0 | Yes  No | Read the following to the participant: Chifukwa chikuti mwakhalapo ndi maganizo akuti kulibwino mungofa kapena ofuna kuzivulaza nokha munjira ina yake, pamapeto pamafunsowa ndikufotokozerani za momwe mungapezere chithandizo. Kodi pakali pano mutha kupiliza kuyankha mafunso otsarawa? | Eya  Ayi | Read the following to the participant: Chifukwa chikuti mwawapo na maghanoghano yakuti chingaba makola kufwa panji kujipweteka mwekha mu nthowa yinyakhe, pawumaliro pa mafumbo aya ndimulongosoleraninge umo mungasangila wovwiri. Kasi tingalutilira kumufumbani mafumbo yakhalako? | Enya  Yayi | This note is for participants that are at risk of suicide. Read note exactly as written and inquire if participant is willing to continue with interview. If yes, the tablet will remind you to carryout appropriate actions at the end of the interview. If not, skip to safety action as described in “SUICIDESAFETY_NOTE “ |
| *CONTSKIP* | IF YES, continue with interview. You will be reminded by the tablet to carry out the appropriate actions at the end of the interview. IF NO, skip to safety action section. |  |  | IF YES, continue with interview. You will be reminded by the tablet to carry out the appropriate actions at the end of the interview. IF NO, skip to safety action section. |  | IF YES, continue with interview. You will be reminded by the tablet to carry out the appropriate actions at the end of the interview. IF NO, skip to safety action section. |  |  |
| *SSINTRO* | Now I am going to ask you some questions about the past. Don’t answer about how you have felt in the last month, but think about ANY time in your life before that. |  |  | Pano ndikufunsani mafunso okhudza nthawi ya m’mbuyomu. Musayankhe za mmene mumamvera mwezi watha koma muganizire za nthawi ya m’mbuyo m’moyo wanu kuposera mwezi watha. |  | Sono ndikufumbaninge mafumbo yanyake yakukhwaskana na nyengo yakumanyuma. Mungazgolanga yayi umo mwangujipulikira mumwezi umoza uwo wamala kweni mughanaghanire zanyengo yakumanyuma mumoyo winu kujumpha mwezi wamala. |  | These questions are about past events. Read note exactly as written. Emphasize to the participant that these questions are about ANY time in their life. |
| **GMH** | **MENTAL HEALTH** |  |  | **MENTAL HEALTH** |  | **MENTAL HEALTH** |  |  |
| *INTRO* | I am now going to ask you some questions about how you have been thinking and feeling during the past two weeks.   There are 4 possible answers for each question:   “Not at all”, means you have not had the symptom at all in the last 2 weeks. “Sometimes”, means you have had the symptom but less than half the days in the past two weeks. “Usually”, means you have had the symptom on more than half the days but not all the time in the past two weeks. Finally, “Almost always” means you have had the symptom every day, or almost every day.   [IF YOU THINK THAT THE PARTICIPANT WOULD BENEFIT FROM USING THE WATER GLASSES VISUAL AID, SHOW IT TO THE PARTICIPANT AND GIVE THE FOLLOWING INSTRUCTIONS]  [Point at empty glass] “Not at all”, means you have not had the symptom at all in the last 2 weeks. [Point at 1/3 full glass] “Sometimes”, means you have had the symptom but less than half the days in the past two weeks. [Point at 2/3 full glass]. “Usually”, means you have had the symptom on more than half the days but not all the time in the past two weeks. [Point at full glass], Finally “Almost always” means you have had the symptom every day, or almost every day. |  |  | Pano ndikufunsani mafunso okhudzana ndi momwe mwakhala mukuganizira ndi kumvera mu mtima mwanu mu masabata awiri apitawa.   Pali mayankho anayi pafunso lina lililonse:    Yankho loti “Palibe” likutanthauza kuti simunakhalepo ndi vuto limene mwafunsidwa mu masabata awiri apitawa. Yankho loti “Nthawi zina” likutanthauza kuti mwakhala ndi vutoli koma kochepera theka (hafu) la masiku a masabata awiri apitawa. Yankho loti “Kawirikawiri” likutanthauza kuti mwakhala ndi vutoli koposera theka (hafu) la masabata awiri apitawa, koma osati tisku ndi tsiku. Yankho lomalizira ndi loti “Pafupifupi nthawi zonse”, limene likutanthauza kuti mwakhala ndi vuto limene mwafunsidwali tsiku lililonse, kapena pafupifupi tsiku lilonse.   [IF YOU THINK THAT THE PARTICIPANT WOULD BENEFIT FROM USING THE WATER GLASSES VISUAL AID, SHOW IT TO THE PARTICIPANT AND GIVE THE FOLLOWING INSTRUCTIONS]  [Point at empty glass] Yankho loti “Palibe” likutanthauza kuti simunakhalepo ndi vuto limene mwafunsidwa mu masabata awiri apitawa. [Point at 1/3 full glass]. Yankho loti “Nthawi zina” likutanthauza kuti mwakhala ndi vutoli koma kochepera theka (hafu) la masiku a masabata awiri apitawa. [Point at 2/3 full glass] Yankho loti “Kawirikawiri” likutanthauza kuti mwakhala ndi vutoli koposera theka (hafu) la masabata awiri apitawa, koma osati tisku ndi tsiku. [Point at full glass] Yankho lomalizira ndi loti “Pafupifupi nthawi zonse”, limene likutanthauza kuti mwakhala ndi vuto limene mwafunsidwali tsiku lililonse, kapena pafupifupi tsiku lilonse. |  | Sono nikufumbaninge mafumbo yakukhwaskana na umo mwakhala mukughanaghanira na kupulikira mu mtima winu mu masabata yawiri aya yajumpha.   Pali mazgolo yanayi pafumbo lililose:  Zgolo lakuti “Palije” likungánamula kuti mundawepo na suzgo ilo mwafumbika mu masabata yawiri yajumpha. Zgolo lakuti “Nyengo zinyakhe” likungánamula kuti mwawa na sugzo ili kweni kwakuchepera hafu ya mazuwa ya mu masabata yawiri yajumpha. Zgolo lakuti “Kawirikawiri” likungánamula kuti mwawa na sugzo ili kwakujumpha hafu ya masabata yawiri yajumpha, kweni dazi na dazi yayi. Zgolo lakuti “Pafupifupi nyengo zose” likungánamula kuti mwawa na suzgo ilo mwafumbika kwa dazi lililose, panji pafupifupi dazi lililose.   [IF YOU THINK THAT THE PARTICIPANT WOULD BENEFIT FROM USING THE WATER GLASSES VISUAL AID, SHOW IT TO THE PARTICIPANT AND GIVE THE FOLLOWING INSTRUCTIONS]  [Point at empty glass] Zgolo lakuti “Palije” likungánamula kuti mundawepo na suzgo ilo mwafumbika mu masabata yawiri yajumpha. [Point at 1/3 full glass]. Zgolo lakuti “Nyengo zinyakhe” likungánamula kuti mwawa na sugzo ili kweni kwakuchepera hafu ya mazuwa ya mu masabata yawiri yajumpha. [Point at 2/3 full glass]. Zgolo lakuti “Kawirikawiri” likungánamula kuti mwawa na sugzo ili kwakujumpha hafu ya masabata yawiri yajumpha, kweni dazi na dazi yayi. [Point at full glass] Zgolo lakuti “Pafupifupi nyengo zose” likungánamula kuti mwawa na suzgo ilo mwafumbika kwa dazi lililose, panji pafupifupi dazi lililose. |  | This section asks participants on mental health symptoms they have been experiencing in the past two weeks. The interviewer will also assess whether the participant can use the water glass visual aids and explain it to them as written in the introductory script. If the interviewer thinks the participant will not benefit from using the visual aid, (s)he should read out the four options to the participant.  Each question has a note of an example of how a question should be rephrased if the participant does not understand it. Interviewer should read this slowly and as exactly as written |
| *PHQ9* | PHQ9 |  |  | PHQ9 |  | PHQ9 |  |  |
| *FEELDOWN* | During the past two weeks, how much have you been feeling down, depressed, or hopeless? | 0  1  2  3  6 | Not at all  Sometimes  Usually  Almost Always  Refused to answer | Mu masabata awiri apitawa, ndi mochuluka bwanji pamene mwakhala osasangalala, wokhumudwa, kapena opanda chiyembekezo? | Palibe  Nthawi zina  Kawirikawiri  Pafupifupi nthawi zonse  Refused to answer | Mu masabata yawiri ayo yajumpha, nkhanandi uli ako mwawa wambula kukondwa, kukwebeka, panji kuwavya chigomezgo? | Palije  Nyengo zinyakhe  Kawirikawiri  Pafupifupi nyengo zose  Refused to answer | Record as responded. Read out the options if not using the visual aid. |
| *FEELDOWN_NOTE* | [IF THE PARTICIPANT IS NOT SURE WHAT THE QUESTION MEANS, READ THIS EXAMPLE AS IT IS WRITTEN]  Sometimes a person feels miserable because of something in their life or even for no clear reason; or feels that nothing they do will make life better. How much has this happened to you in the last 2 weeks? |  |  | [IF THE PARTICIPANT IS NOT SURE WHAT THE QUESTION MEANS, READ THIS EXAMPLE AS IT IS WRITTEN]  Nthawi zina munthu amatha kukhala wokhumudwa chifukwa cha chinthu china m’moyo mwake kapenanso opanda chifukwa chimene; komanso amatha kukhala ndi maganizo akuti palibe chomwe angachite kuti akometse moyo wake. Ndi mochuluka bwanji zimenezi zinakuchitikirani mu masabata awiri apitawa? |  | [IF THE PARTICIPANT IS NOT SURE WHAT THE QUESTION MEANS, READ THIS EXAMPLE AS IT IS WRITTEN]  Ngengo zinyake munthu wakujipulika wambula kukondwa na ivyo vikuchitika pa moyo wakhe nanga pangaba chifukwa chambula kupulikwika pakunji palije icho bangachita chakuti chipangiske moyo wabo wube makora. Nkhanandi uli ivi vyamuchitikiranipo mumasabata yawiri ayo yajumpha? |  | Read note if participant does not understand question. |
| *ANHEDONIA* | During the past two weeks, how much have you had little interest or pleasure in doing things? | 0  1  2  3  6 | Not at all  Sometimes  Usually  Almost Always  Refused to answer | Mu masabata awiri apitawa, ndi mochuluka bwanji pamene mwakhala ndi chidwi chochepa kapena osasangalatsidwa pochita zinthu? | Palibe  Nthawi zina  Kawirikawiri  Pafupifupi nthawi zonse  Refused to answer | Mu masabata yawiri ayo yajumpha, nkhanandi uli ako mwawa wa kunweka kwa pachoko panji kukhala wambula kusangwa pakuchita vinthu? | Palije  Nyengo zinyakhe  Kawirikawiri  Pafupifupi nyengo zose  Refused to answer | Record as responded. Read out the options if not using the visual aid. |
| *ANHEDONIA_NOTE* | [IF THE PARTICIPANT IS NOT SURE WHAT THE QUESTION MEANS, READ THIS EXAMPLE AS IT IS WRITTEN]   Sometimes a person gets no pleasure or has no interest when doing things such as cooking, working or meeting with friends, even though he/she used to enjoy those things. How much has this happened to you in the last 2 weeks? |  |  | [IF THE PARTICIPANT IS NOT SURE WHAT THE QUESTION MEANS, READ THIS EXAMPLE AS IT IS WRITTEN]  Nthawi zina munthu amatha kukhala opanda chidwi kapena osasangalatsidwa pochita zinthu monga kuphika, kugwira ntchito kapena kukumana ndi anzake, ngakhale m’mbuyomu anali kusangalatsidwa nazo. Ndi mochuluka bwanji zimenezi zinakuchitikirani mu masabata awiri apitawa? |  | [IF THE PARTICIPANT IS NOT SURE WHAT THE QUESTION MEANS, READ THIS EXAMPLE AS IT IS WRITTEN]   Nyengo zinyake munthu wakuwa wambula kusangwa panji wambula kunweka navinthu ivyo wakatemwanga kupanga ngati kuphika, kugwira tchito panji kuchezga na wabwezi, nangauli kale wakasanguluskikanga navinthu vyeneivyi. Nkhanandi uli ivi vyamuchitikiranipo mumasabata yawiri ayo yajumpha? |  | Read note if participant does not understand question. |
| *PROBSLEEP* | During the past two weeks, how much have you had trouble falling or staying asleep, or sleeping too much? | 0  1  2  3  6 | Not at all  Sometimes  Usually  Almost Always  Refused to answer | Mu masabata awiri apitawa, ndi mochuluka bwanji pamene mwakhala mukuvutika kupeza tulo kapena kugona mosakwanira, kapenanso kugona kwambiri? | Palibe  Nthawi zina  Kawirikawiri  Pafupifupi nthawi zonse  Refused to answer | Mu masabata yawiri ayo yajumpha, nkhanandi uli ako mwawa wa kutondeka kusanga tulo panji kugona mwambula kukwanira, panji kugona chomene? | Palije  Nyengo zinyakhe  Kawirikawiri  Pafupifupi nyengo zose  Refused to answer | Record as responded. Read out the options if not using the visual aid. |
| *PROBSLEEP_NOTE* | [IF THE PARTICIPANT IS NOT SURE WHAT THE QUESTION MEANS, READ THIS EXAMPLE AS IT IS WRITTEN]   Sometimes a person tries to go to sleep but cannot, or wakes often even if he/she is tired, or sleeps for longer than he/she would normally do. How much has this happened to you in the last 2 weeks? |  |  | [IF THE PARTICIPANT IS NOT SURE WHAT THE QUESTION MEANS, READ THIS EXAMPLE AS IT IS WRITTEN]  Nthawi zina munthu amayesera kugona koma tulo osabwera kapena kumangozizimuka pafupifupafupi angakhale iye ali wotopa, kapena kugona nthawi yaitali modabwitsa. Ndi mochuluka bwanji zimenezi zinakuchitikirani mu masabata awiri apitawa? |  | [IF THE PARTICIPANT IS NOT SURE WHAT THE QUESTION MEANS, READ THIS EXAMPLE AS IT IS WRITTEN]   Nyengo zinyake munthu wangakhumba kuti wakagone kweni nakusoba tulo, panji wakuwukawuka apo wagona nanga wangaba kuti wavuka, panji wakugona mwakujumphizga umo wakugonera nyengozose. Nkhanandi uli ivi vyamuchitikiranipo mumasabata yawiri ayo yajumpha? |  | Read note if participant does not understand question. |
| *TIRED* | During the past two weeks, how much have you been feeling tired, fatigued or having little energy? | 0  1  2  3  6 | Not at all  Sometimes  Usually  Almost Always  Refused to answer | Mu masabata awiri apitawa, ndi mochuluka bwanji pamene mwakhala mukumva kutopa, kufowoka kapena kuchepa mphamvu? | Palibe  Nthawi zina  Kawirikawiri  Pafupifupi nthawi zonse  Refused to answer | Mu masabata yawiri ayo yajumpha, nkhanandi uli ako mwawa wa kuvuka, wakulema, panji kuchepelwa nkhongono? | Palije  Nyengo zinyakhe  Kawirikawiri  Pafupifupi nyengo zose  Refused to answer | Record as responded. Read out the options if not using the visual aid. |
| *TIRED_NOTE* | [IF THE PARTICIPANT IS NOT SURE WHAT THE QUESTION MEANS, READ THIS EXAMPLE AS IT IS WRITTEN]    Sometimes a person feels particularly tired and weak and finds everything an effort to do, even if he/she has had rest. How much has this happened to you in the last 2 weeks? |  |  | [IF THE PARTICIPANT IS NOT SURE WHAT THE QUESTION MEANS, READ THIS EXAMPLE AS IT IS WRITTEN]  Nthawi zina munthu amakhala wotopa ndi wofooka okuti amapeza china chili chonse chakuti achite kukhala kulimbana nacho kuti amalizitse angakhale iye wapumula mokwanira. Ndi mochuluka bwanji zimenezi zinakuchitikirani mu masabata awiri apitawa? |  | [IF THE PARTICIPANT IS NOT SURE WHAT THE QUESTION MEANS, READ THIS EXAMPLE AS IT IS WRITTEN]    Nyengo zinyakhe munthu wakuwa wakuvuka nakufooka mwakuti wakusanga chilichose chakuti wachite chikuwa chinonono kumalizgiska, nanga wuli iyo wapumula mwakukwanila. Nkhanandi uli ivi vyamuchitikiranipo mumasabata yawiri ayo yajumpha? |  | Read note if participant does not understand question. |
| *POORAPP* | During the past two weeks, how much have you had poor appetite or been overeating? | 0  1  2  3  6 | Not at all  Sometimes  Usually  Almost Always  Refused to answer | Mu masabata awiri apitawa, ndi mochuluka bwanji pamene mwakhala osowa chilakolako cha chakudya kapena kumadya kwambiri? | Palibe  Nthawi zina  Kawirikawiri  Pafupifupi nthawi zonse  Refused to answer | Mu masabata yawiri ayo yajumpha, nkhanandi uli ako mwawa wa kuwavya khumbilo la chakurya panji kurya mwakujumphizga? | Palije  Nyengo zinyakhe  Kawirikawiri  Pafupifupi nyengo zose  Refused to answer | Record as responded. Read out the options if not using the visual aid. |
| *POORAPP_NOTE* | [IF THE PARTICIPANT IS NOT SURE WHAT THE QUESTION MEANS, READ THIS EXAMPLE AS IT IS WRITTEN]   Sometimes a person finds that, even though there is food available to eat at home, they are not interested in eating it. Or they may eat more than usual because of how they are feeling emotionally. How much has this happened to you in the last 2 weeks? |  |  | [IF THE PARTICIPANT IS NOT SURE WHAT THE QUESTION MEANS, READ THIS EXAMPLE AS IT IS WRITTEN]   Nthawi zina munthu amakhala opanda chilakolako choti adye angakhale chakudya chilipo panyumba. Kapenanso nthawi zina amadya kwambiri kuposa mmene achitira nthawi zonse chifukwa cha mmene akumvera mumtima. Ndi mochuluka bwanji zimenezi zinakuchitikirani mu masabata awiri apitawa? |  | [IF THE PARTICIPANT IS NOT SURE WHAT THE QUESTION MEANS, READ THIS EXAMPLE AS IT IS WRITTEN]   Nyengo zinyake munthu wangaba na vyakulya vyakuti walye panyumba, kweni wakusoba khumbiro lakuti walye. Panji wakulya mwakujumphizga mulingo wanyengozose chifukwa cha umo wakujipulikira mumtima. Nkhanandi uli ivi vyamuchitikiranipo mumasabata yawiri ayo yajumpha? |  | Read note if participant does not understand question. |
| *FEELBAD* | During the past two weeks, how much have you been feeling bad about yourself—or that you are a failure or have let yourself or your family down? | 0  1  2  3  6 | Not at all  Sometimes  Usually  Almost Always  Refused to answer | Mu masabata awiri apitawa, ndi mochuluka bwanji pamene mwakhala musakumva bwino mu mtima za inu mwini - kapena kuziona kuti ndinu olephera kapena kuzikhumudwitsa kapena kukhumudwitsa banja lanu? | Palibe  Nthawi zina  Kawirikawiri  Pafupifupi nthawi zonse  Refused to answer | Mu masabata yawiri ayo yajumpha, nkhanandi uli ako mwanguwa kwambula kujipulika makola mumtima vya imwe mwawene, mwajiwona kuti ndimwe wambula kwenelera, panji kuti muli wakutondeka, panji kuti mwajikhozga soni mwekha panji kukhozga soni banja linu? | Palije  Nyengo zinyakhe  Kawirikawiri  Pafupifupi nyengo zose  Refused to answer | Record as responded. Read out the options if not using the visual aid. |
| *FEELBAD_NOTE* | [IF THE PARTICIPANT IS NOT SURE WHAT THE QUESTION MEANS, READ THIS EXAMPLE AS IT IS WRITTEN]   Sometimes a person feels worthless, blames themselves for all their problems, or feels that they have disappointed people they care about. How much has this happened to you in the last 2 weeks? |  |  | [IF THE PARTICIPANT IS NOT SURE WHAT THE QUESTION MEANS, READ THIS EXAMPLE AS IT IS WRITTEN]   Nthawi zina munthu amakhala ndi maganizo akuti iye ndiwopanda phindu ndipo amaziloza chala iye mwini chifukwa cha mavuto ake onse, kapenanso amatha kukhala ndi maganizo akuti wakhumudwitsa anthu a pamtima pake. Ndi mochuluka bwanji zimenezi zinakuchitikirani mu masabata awiri apitawa? |  | [IF THE PARTICIPANT IS NOT SURE WHAT THE QUESTION MEANS, READ THIS EXAMPLE AS IT IS WRITTEN]    Nyengo zinyake munthu wakujiwona wambula phindu, kujitundura yekha pa masuzgo yose agho wakusangananayo, pakunji wakujipulika kuti wakhubazga banthu awo bakupweleleka na iyo. Nkhanandi uli ivi vyamuchitikiranipo mumasabata yawiri ayo yajumpha? |  | Read note if participant does not understand question. |
| *CONCENTRATE* | During the past two weeks, how much have you had trouble concentrating on things, such as reading, talking with friends, finishing tasks, or attending a meeting? | 0  1  2  3  6 | Not at all  Sometimes  Usually  Almost Always  Refused to answer | Mu masabata awiri apitawa, ndi mochuluka bwanji pamene mwakhala ovutika kukhazikitsa maganizo anu pa chinthu chimodzi monga kuwerenga, kucheza ndi anzanu, kumalizitsa ntchito imene mwayiyamba kapena kutenga mbali mu zokambirana? | Palibe  Nthawi zina  Kawirikawiri  Pafupifupi nthawi zonse  Refused to answer | Mu masabata yawiri ayo yajumpha, nkhanandi uli ako mwawa wakutondeka kuwika maghanoghano yinu ghamoza pavinthu panji kutondeka kufiska chinthu icho mwachiyamba kuchita ngati kuwerenga, kuyowoya na wabwezi, kumalizga zintchito, panji kutolapo lwande pa nkhumano? | Palije  Nyengo zinyakhe  Kawirikawiri  Pafupifupi nyengo zose  Refused to answer | Record as responded. Read out the options if not using the visual aid. |
| *CONCENTRATE_NOTE* | [IF THE PARTICIPANT IS NOT SURE WHAT THE QUESTION MEANS, READ THIS EXAMPLE AS IT IS WRITTEN]   Sometimes a person finds that they cannot complete tasks that they normally would because their mind wanders or they are thinking about other things. How much has this happened to you in the last 2 weeks? |  |  | [IF THE PARTICIPANT IS NOT SURE WHAT THE QUESTION MEANS, READ THIS EXAMPLE AS IT IS WRITTEN]   Nthawi zina munthu amapezeka kuti akulephera kumalizitsa ntchito zakuti m’mbuyomu amakwaniritsa chifukwa cholephera kukhazikitsa ma maganizo ake pamodzi kapenanso chifukwa chakuti akuganizira zinthu zina. Ndi mochuluka bwanji zimenezi zinakuchitikirani mu masabata awiri apitawa? |  | [IF THE PARTICIPANT IS NOT SURE WHAT THE QUESTION MEANS, READ THIS EXAMPLE AS IT IS WRITTEN]    Nyengo zinyake munthu wangatondeka kumaliziska milimo iyo nyengozose wakamalizganga makola waka chifukwa cha kutimbanizgika mukaghanaghaniro panji kutimbanizgika chifukwa chakughanaghana vinthu vinyake. Nkhanandi uli ivi vyamuchitikiranipo mumasabata yawiri ayo yajumpha? |  | Read note if participant does not understand question. |
| *SLOW* | During the past two weeks, how much have you been moving or speaking so slowly that other people could have noticed? | 0  1  2  3  6 | Not at all  Sometimes  Usually  Almost Always  Refused to answer | Mu masabata awiri apitawa, ndi mochuluka bwanji pamene mwakhala mukuyenda kapena kulankhula mwa pang’onopang’ono moti anthu ena anazindikira zimenezi? | Palibe  Nthawi zina  Kawirikawiri  Pafupifupi nthawi zonse  Refused to answer | Mu masabata yawiri ayo yajumpha, nkhanandi uli ako mwayendanga panji mwayowoyanga mwapachokopachoko chomene kuti mpaka wanthu wanyakhe bakamubowozgani? | Palije  Nyengo zinyakhe  Kawirikawiri  Pafupifupi nyengo zose  Refused to answer | Record as responded. Read out the options if not using the visual aid. |
| *SLOW_NOTE* | [IF THE PARTICIPANT IS NOT SURE WHAT THE QUESTION MEANS, READ THIS EXAMPLE AS IT IS WRITTEN]   Sometimes a person will walk or talk much more slowly than normal because of how they are feeling emotionally. How much has this happened to you in the last 2 weeks? |  |  | [IF THE PARTICIPANT IS NOT SURE WHAT THE QUESTION MEANS, READ THIS EXAMPLE AS IT IS WRITTEN]   Nthawi zina munthu amayenda kapena kuyankhula pang’onopang’ono chifukwa chammene akumvera mumtima. Ndi mochuluka bwanji zimenezi zinakuchitikirani mu masabata awiri apitawa? |  | [IF THE PARTICIPANT IS NOT SURE WHAT THE QUESTION MEANS, READ THIS EXAMPLE AS IT IS WRITTEN]   Nyengo zinyake munthu wangendanga panji kuyowoya mwapachokopachoko chomene kuluska nyengo zose chifukwa cha umo wakujipilikira mumtima. Nkhanandi uli ivi vyamuchitikiranipo mumasabata yawiri ayo yajumpha? |  | Read note if participant does not understand question. |
| *RESTLESSDEP* | During the past two weeks, how much have you been so fidgety or restless that you have been moving around a lot more than usual? | 0  1  2  3  6 | Not at all  Sometimes  Usually  Almost Always  Refused to answer | Mu masabata awiri apitawa, ndi mochuluka bwanji pamene mwakhala ndi mtima woti siunali mmalo mwakuti mwakhala mukumangoyendayenda koposa mmene mumachitira masiku onse? | Palibe  Nthawi zina  Kawirikawiri  Pafupifupi nthawi zonse  Refused to answer | Mu masabata yawiri ayo yajumpha, nkhanandi uli ako mtima winu ukawa mmalo mwakhe yaye mwakuti mwendendekanga mwakuluska nyengo zose? | Palije  Nyengo zinyakhe  Kawirikawiri  Pafupifupi nyengo zose  Refused to answer | Record as responded. Read out the options if not using the visual aid. |
| *RESTLESSDEP_NOTE* | [IF THE PARTICIPANT IS NOT SURE WHAT THE QUESTION MEANS, READ THIS EXAMPLE AS IT IS WRITTEN]   Sometimes a person is so worried or sad that they cannot relax and they move about much more than usual. How much has this happened to you in the last 2 weeks? |  |  | [IF THE PARTICIPANT IS NOT SURE WHAT THE QUESTION MEANS, READ THIS EXAMPLE AS IT IS WRITTEN]   Nthawi zina munthu amakhala` wodandaula kapena wokhumudwa kwambiri ndipo mtima wake siukhala mmalo motero kuti amangoyendayenda/amangoti zungulizunguli. Ndi mochuluka bwanji zimenezi zinakuchitikirani mu masabata awiri apitawa? |  | [IF THE PARTICIPANT IS NOT SURE WHAT THE QUESTION MEANS, READ THIS EXAMPLE AS IT IS WRITTEN]    Nyengo zinyake munthu wakudandaula panji kuba na chitima mwakuti wakutondeka kufwasa kweniso kwendendekanga chomene kuluska nyengo zose. Nkhanandi uli ivi vyamuchitikiranipo mumasabata yawiri ayo yajumpha? |  | Read note if participant does not understand question. |
| *SUICIDE* | During the past two weeks, how much have you had thoughts that you would be better off dead or of hurting yourself in some way? | 0  1  2  3  6 | Not at all  Sometimes  Usually  Almost Always  Refused to answer | Mu masabata awiri apitawa, ndi mochuluka bwanji pamene mwakhala ndi maganizo akuti kuli bwino mungofa kapena mungozivulaza nokha mwanjira ina yake? | Palibe  Nthawi zina  Kawirikawiri  Pafupifupi nthawi zonse  Refused to answer | Mu masabata yawiri ayo yajumpha, nkhanandi uli ako mwawa na maghanoghano ghakuti chingawa makola kufwa panji kujipweteka mwekha mu nthowa yinyakhe? | Palije  Nyengo zinyakhe  Kawirikawiri  Pafupifupi nyengo zose  Refused to answer | Record as responded. Read out the options if not using the visual aid. |
| *SUICIDE_NOTE* | [IF THE PARTICIPANT IS NOT SURE WHAT THE QUESTION MEANS, READ THIS EXAMPLE AS IT IS WRITTEN]   Sometimes a person feels so down that he/she has thoughts that life is no longer worth living and he/she thinks about ending his/her life or hurting him/herself for example using poison or hanging. How much has this happened to you in the last 2 weeks? |  |  | [IF THE PARTICIPANT IS NOT SURE WHAT THE QUESTION MEANS, READ THIS EXAMPLE AS IT IS WRITTEN]   Nthawi zina munthu amakhala osasangalala ndipo amakhala ndi maganizo akuti moyo ulibe phindu ndipo amaganizanso zozipha kapena zozivulaza iye mwini mwachitsanzo pogwiritsa ntchito poyizoni kapena kuzikhweza/kuzimangilirira. Ndi mochuluka bwanji zimenezi zinakuchitikirani mu masabata awiri apitawa? |  | [IF THE PARTICIPANT IS NOT SURE WHAT THE QUESTION MEANS, READ THIS EXAMPLE AS IT IS WRITTEN]  Nyengo zinyake munthu wangaba wambula kukondwa ndipo akuba na maghanoghano yakuti moyo wakhe ngwambula kwenelera panji kubaso namaghanoghano yakujipweteka/kujikoma pakugwiriska ntchito vinthu ngati poyizoni panji kujikaka. Nkhanandi uli ivi vyamuchitikiranipo mumasabata yawiri ayo yajumpha? |  | Read note if participant does not understand question. |
| *DIFFPROBSNOTE* | [DO NOT USE THE VISUAL AID FOR THIS QUESTION] |  |  | [DO NOT USE THE VISUAL AID FOR THIS QUESTION] |  | [DO NOT USE THE VISUAL AID FOR THIS QUESTION] |  |  |
| *DIFFICULTYPROBS* | In the last 2 weeks, how difficult have these problems made it for you to do your work, take care of things at home or get along with other people [READ THE OPTIONS]? | 0  1  2  3  6 | Not difficult at all  Somewhat difficult  Very difficult  Extremely difficult  Refused to answer | Mu masabata awiri apitawa, kodi mabvuto amenewa alimbitsa bwanji kagwiridwe kanu ka ntchito, kasamaliridwe kanu ka zinthu pakhomo, kapena ubale wanu ndi anthu: [READ THE OPTIONS]? | Sindinavutikepo  Pang’ono pokha  Mochuluka  Mochuluka kwambiri  Refused to answer | Mu masabata yawiri ayo yajumpha, kasi masuzgo aya yiza na maunonono wuli pa umo mukugwirira ntchito, kupwererera vinthu pa nyumba, panji ubale winu na banthu: [READ THE OPTIONS]? | Unonono pakawavya  Pachoko waka  Chomene  Chomene nkhanila  Refused to answer | Do not use the visual aid for this question. |
| *DIFFICULTYPROBS_NOTE* | [IF THE PARTICIPANT IS NOT SURE WHAT THE QUESTION MEANS, READ THIS EXAMPLE AS IT IS WRITTEN]    Sometimes when a person has problems like feeling depressed, tired or hopeless, the person cannot do their usual activities. For you, in the last 2 weeks, how difficult have you found it to do your normal activities: Not difficult at all; Somewhat difficult; Very difficult or Extremely difficult? |  |  | [IF THE PARTICIPANT IS NOT SURE WHAT THE QUESTION MEANS, READ THIS EXAMPLE AS IT IS WRITTEN]   Nthawi zina munthu amakhala ndi mavuto monga kukhumudwa, kutopa kapenanso kupanda chiyembekezo, mwakuti munthuyo sangathe kuchita ntchito za moyo wake wa tsiku ndi tsiku. Inuyo mu masabata awiri apitawa, zimakuvutani bwanji kuchita ntchito za moyo wanu wa tsiku ndi tsiku: Sindinavutikepo; Pang’ono pokha; Mochuluka; Mochuluka kwambiri? |  | [IF THE PARTICIPANT IS NOT SURE WHAT THE QUESTION MEANS, READ THIS EXAMPLE AS IT IS WRITTEN]   Nyengo zinyake pala munthu wanamasuzgo ngati kuba wakukwebeka, wakuvuka panji wambula chigomezgo, wangatondeka kukwaniska kugwira tchito izo wakakwaniskanga nyengo zose. Kasi imwe, mumasabata yawiri ayo yajumpha, mwasanga maunonono uli ayo yamutondeskani kugwira ntchito ngati umo mukuchitira nyengo zose: Unonono pakawavya; Pachoko waka; Chomene; Chomene nkhanila? |  |  |
| ***GAD7*** | **GAD7** |  |  | **GAD7** |  | **GAD7** |  |  |
| *NERVOUS* | Over the last two weeks, how much have you been feeling nervous, anxious, or on edge? | 0  1  2  3  6 | Not at all  Sometimes  Usually  Almost Always  Refused to answer | Mu masabata awiri apitawa, ndi mochuluka bwanji pamene mu mtima mwanu mwakhala ndimantha, nkhawa, kapena kusowa mtendele? | Palibe  Nthawi zina  Kawirikawiri  Pafupifupi nthawi zonse  Refused to answer | Mu masabata yawiri ayo yajumpha, nkhanandi uli ako mu mtima mwinu mwanguwa na wofi, wa kudandaula, panji wambula mtende? | Palije  Nyengo zinyakhe  Kawirikawiri  Pafupifupi nyengo zose  Refused to answer | Record as responded. Read out the options if not using the visual aid. |
| *NERVOUS_NOTE* | [IF THE PARTICIPANT IS NOT SURE WHAT THE QUESTION MEANS, READ THIS EXAMPLE AS IT IS WRITTEN]    Sometimes a person feels fearful even if there is no danger, or worries that something bad will happen to him/herself or family? How much has this happened to you in the last 2 weeks? |  |  | [IF THE PARTICIPANT IS NOT SURE WHAT THE QUESTION MEANS, READ THIS EXAMPLE AS IT IS WRITTEN]   Nthawi zina munthu amakhala ndi mantha ngakhale palibe choopsa chilichonse, kapenanso amakhala wodandaula kuti china chake choyipa chimuchitikira iye kapena banja lake. Ndi mochuluka bwanji zimenezi zinakuchitikirani mu masabata awiri apitawa? |  | [IF THE PARTICIPANT IS NOT SURE WHAT THE QUESTION MEANS, READ THIS EXAMPLE AS IT IS WRITTEN]    Nyengo zinyake munthu wakuba na wofi kwambula chakofya chilichose panji kudandaula kuti viheni vitimuchitikilenge iyo panji bapabanja lakhe? Nkhanandi uli ivi vyamuchitikiranipo mumasabata yawiri ayo yajumpha? |  | Read note if participant does not understand question. |
| *WORRYING* | Over the last two weeks, how much have you not been able to stop or control worrying? | 0  1  2  3  6 | Not at all  Sometimes  Usually  Almost Always  Refused to answer | Mu masabata awiri apitawa, ndi mochuluka bwanji pamene mwakhala olephera kusiya kapena kuchepetsa kudandaula? | Palibe  Nthawi zina  Kawirikawiri  Pafupifupi nthawi zonse  Refused to answer | Mu masabata yawiri ayo yajumpha, nkhanandi uli ako mwawa wa kutondeka kuleka panji kuchepeska kudandaula? | Palije  Nyengo zinyakhe  Kawirikawiri  Pafupifupi nyengo zose  Refused to answer | Record as responded. Read out the options if not using the visual aid. |
| *WORRYING_NOTE* | [IF THE PARTICIPANT IS NOT SURE WHAT THE QUESTION MEANS, READ THIS EXAMPLE AS IT IS WRITTEN]   Sometimes a person cannot stop having worrying thoughts even if he/she normally could do so. How much has this happened to you in the last 2 weeks? |  |  | [IF THE PARTICIPANT IS NOT SURE WHAT THE QUESTION MEANS, READ THIS EXAMPLE AS IT IS WRITTEN]   Nthawi zina munthu amalephera kusiya kukhala ndi maganizo odandaula ngakhale iye ali ndi kuthekera kosiya kudandaulako. Ndi mochuluka bwanji zimenezi zinakuchitikirani mu masabata awiri apitawa? |  | [IF THE PARTICIPANT IS NOT SURE WHAT THE QUESTION MEANS, READ THIS EXAMPLE AS IT IS WRITTEN]   Nyengo zinyake munthu wakutondeka kujilekeska maghanoghano gha kudandaula nanga wangayezeska theula. Nkhanandi uli ivi vyamuchitikiranipo mumasabata yawiri ayo yajumpha? |  | Read note if participant does not understand question. |
| *WORRYDIFF* | Over the last two weeks, how much have you been worrying too much about different things? | 0  1  2  3  6 | Not at all  Sometimes  Usually  Almost Always  Refused to answer | Mu masabata awiri apitawa, ndi mochuluka bwanji mwakhala odandaula kwambiri za zinthu zosiyanasiyana? | Palibe  Nthawi zina  Kawirikawiri  Pafupifupi nthawi zonse  Refused to answer | Mu masabata yawiri ayo yajumpha, nkhanandi uli ako mwanguwa wakudandaula mwakujumphizga kukhwaskana na vinthu vyakupambanapambana? | Palije  Nyengo zinyakhe  Kawirikawiri  Pafupifupi nyengo zose  Refused to answer | Record as responded. Read out the options if not using the visual aid. |
| *WORRYDIFF_NOTE* | [IF THE PARTICIPANT IS NOT SURE WHAT THE QUESTION MEANS, READ THIS EXAMPLE AS IT IS WRITTEN]   Sometimes a person worries about many things such as family, health, money, future plans, all at the same time. How much has this happened to you in the last 2 weeks? |  |  | [IF THE PARTICIPANT IS NOT SURE WHAT THE QUESTION MEANS, READ THIS EXAMPLE AS IT IS WRITTEN]   Nthawi zina munthu amadandaula za zinthu zambiri monga banja, umoyo, ndalama, mapulani amtsogolo, zonsezi panthawi imodzi. Ndi mochuluka bwanji zimenezi zinakuchitikirani mu masabata awiri apitawa? |  | [IF THE PARTICIPANT IS NOT SURE WHAT THE QUESTION MEANS, READ THIS EXAMPLE AS IT IS WRITTEN]   Nyengo zinyake munthu wakudandaula kukhwaskana na vinthu vinadi ngati banja, moyo, ndalama, mapulani yamunthazi kweni vyose ivyi panyengo yimoza. Nkhanandi uli ivi vyamuchitikiranipo mumasabata yawiri ayo yajumpha? |  | Read note if participant does not understand question. |
| *PROBRELAX* | Over the last two weeks, how much have you had trouble relaxing? | 0  1  2  3  6 | Not at all  Sometimes  Usually  Almost Always  Refused to answer | Mu masabata awiri apitawa, ndi mochuluka bwanji mwakhala mukuvutika kuti mukhale omasuka? | Palibe  Nthawi zina  Kawirikawiri  Pafupifupi nthawi zonse  Refused to answer | Mu masabata yawiri ayo yajumpha, nkhanandi uli ako mwanguwa wambula kufwasa mu mtima? | Palije  Nyengo zinyakhe  Kawirikawiri  Pafupifupi nyengo zose  Refused to answer | Record as responded. Read out the options if not using the visual aid. |
| *PROBRELAX_NOTE* | [IF THE PARTICIPANT IS NOT SURE WHAT THE QUESTION MEANS, READ THIS EXAMPLE AS IT IS WRITTEN]   Sometimes a person cannot rest even though they do not have work to do. How much has this happened to you in the last 2 weeks? |  |  | [IF THE PARTICIPANT IS NOT SURE WHAT THE QUESTION MEANS, READ THIS EXAMPLE AS IT IS WRITTEN]   Nthawi zina munthu amalephera kukhazikika ngakhale palibe ntchito yomutanganidwitsa. Ndi mochuluka bwanji zimenezi zinakuchitikirani mu masabata awiri apitawa? |  | [IF THE PARTICIPANT IS NOT SURE WHAT THE QUESTION MEANS, READ THIS EXAMPLE AS IT IS WRITTEN]   Nyengo zinyake munthu wakutondeka kujipumuzga nanga pangaba kuti palije ntchito iyo bakugwira. Nkhanandi uli ivi vyamuchitikiranipo mumasabata yawiri ayo yajumpha? |  | Read note if participant does not understand question. |
| *RESTLESSANX* | Over the last two weeks, how much have you been so restless that it is hard to sit still? | 0  1  2  3  6 | Not at all  Sometimes  Usually  Almost Always  Refused to answer | Mu ma sabata awiri apitawa, ndi mochuluka bwanji pamene mtima wanu siunali mmalo mwake mwakuti mumalephera kukhazikika malo amodzi? | Palibe  Nthawi zina  Kawirikawiri  Pafupifupi nthawi zonse  Refused to answer | Mu masabata yawiri ayo yajumpha, nkhanandi uli ako mtima winu wanguwa mmalo mwakhe yayi mwakuti mwatondekanga kuti mukhazikike pa malo yamoza? | Palije  Nyengo zinyakhe  Kawirikawiri  Pafupifupi nyengo zose  Refused to answer | Record as responded. Read out the options if not using the visual aid. |
| *RESTLESSANX_NOTE* | [IF THE PARTICIPANT IS NOT SURE WHAT THE QUESTION MEANS, READ THIS EXAMPLE AS IT IS WRITTEN]  Sometimes a person is so worried or sad that they cannot relax and they move about much more than usual. How much has this happened to you in the last 2 weeks? |  |  | [IF THE PARTICIPANT IS NOT SURE WHAT THE QUESTION MEANS, READ THIS EXAMPLE AS IT IS WRITTEN]   Nthawi zina munthu amakhala` wodandaula kapena wokhumudwa kwambiri ndipo mtima wake siukhala mmalo motero kuti amangoyendayenda/amangoti zungulizunguli. Ndi mochuluka bwanji zimenezi zinakuchitikirani mu masabata awiri apitawa? |  | [IF THE PARTICIPANT IS NOT SURE WHAT THE QUESTION MEANS, READ THIS EXAMPLE AS IT IS WRITTEN]   Nyengo zinyake munthu wakudandaula panji kuba na chitima mwakuti wakutondeka kufwasa kweniso kwendendekanga chomene kuluska nyengo zose. Nkhanandi uli ivi vyamuchitikiranipo mumasabata yawiri ayo yajumpha? |  | Read note if participant does not understand question. |
| *IRRITABLE* | Over the last two weeks, how much have you felt annoyed or irritable? | 0  1  2  3  6 | Not at all  Sometimes  Usually  Almost Always  Refused to answer | Mu ma sabata awiri apitawa, ndi mochuluka bwanji mwakhala wokwiya mosavuta? | Palibe  Nthawi zina  Kawirikawiri  Pafupifupi nthawi zonse  Refused to answer | Mu masabata yawiri ayo yajumpha, nkhanandi uli ako mwakalipanga panji mwakwiyanga mwaluwiro? | Palije  Nyengo zinyakhe  Kawirikawiri  Pafupifupi nyengo zose  Refused to answer | Record as responded. Read out the options if not using the visual aid. |
| *IRRITABLE_NOTE* | [IF THE PARTICIPANT IS NOT SURE WHAT THE QUESTION MEANS, READ THIS EXAMPLE AS IT IS WRITTEN]   Sometimes a person is easily angered by small actions of their family or friends. How much has this happened to you in the last 2 weeks? |  |  | [IF THE PARTICIPANT IS NOT SURE WHAT THE QUESTION MEANS, READ THIS EXAMPLE AS IT IS WRITTEN]   Nthawi zina munthu sachedwa kukwiyisidwa ndi tizinthu ting’onoting’ono tomwe banja lake kapena anzake achita. Ndi mochuluka bwanji zimenezi zinakuchitikirani mu masabata awiri apitawa? |  | [IF THE PARTICIPANT IS NOT SURE WHAT THE QUESTION MEANS, READ THIS EXAMPLE AS IT IS WRITTEN]   Nyengo zinyake munthu wakukwiyanga lubilo na vichito vya banja lakhe panji wabwezi. Nkhanandi uli ivi vyamuchitikiranipo mumasabata yawiri ayo yajumpha? |  | Read note if participant does not understand question. |
| *AFRAID* | Over the last two weeks, how much have you felt afraid as if something awful might happen? | 0  1  2  3  6 | Not at all  Sometimes  Usually  Almost Always  Refused to answer | Mu ma sabata awiri apitawa, ndi mochuluka bwanji pamene mwakhala ndi mantha mu mtima mwanu kuti chinachake choopsa chikhoza kuchitika? | Palibe  Nthawi zina  Kawirikawiri  Pafupifupi nthawi zonse  Refused to answer | Mu masabata yawiri ayo yajumpha, nkhanandi uli ako mwapulika wofi mu mtima nganipala chinyakhe chakofya chingachitika? | Palije  Nyengo zinyakhe  Kawirikawiri  Pafupifupi nyengo zose  Refused to answer | Record as responded. Read out the options if not using the visual aid. |
| *AFRAID_NOTE* | [IF THE PARTICIPANT IS NOT SURE WHAT THE QUESTION MEANS, READ THIS EXAMPLE AS IT IS WRITTEN]   Sometimes a person feels fearful that something bad will happen to him/herself or family? How much has this happened to you in the last 2 weeks? |  |  | [IF THE PARTICIPANT IS NOT SURE WHAT THE QUESTION MEANS, READ THIS EXAMPLE AS IT IS WRITTEN]   Nthawi zina munthu amakhala ndi mantha kuti chinthu china chake choyipa chimuchitikira iye mwini kapenanso banja lake. Ndi mochuluka bwanji zimenezi zinakuchitikirani mu masabata awiri apitawa? |  | [IF THE PARTICIPANT IS NOT SURE WHAT THE QUESTION MEANS, READ THIS EXAMPLE AS IT IS WRITTEN]   Nyengo zinyake munthu wakuba nawofyi kuti chinthu chinyake chiheni chitimuchitikilenge iyo panji banja lakhe. Nkhanandi uli ivi vyamuchitikiranipo mumasabata yawiri ayo yajumpha? |  | Read note if participant does not understand question. |
| ***SOMATIC*** | **Somatic items** |  |  | **Somatic items** |  | **Somatic items** |  |  |
| *HEADACHE* | During the past 2 weeks, how much have you had a headache? | 0  1  2  3  6 | Not at all  Sometimes  Usually  Almost Always  Refused to answer | Mu masabata awiri apitawa, ndi mochuluka bwanji mwakhala mukumva kupweteka mutu? | Palibe  Nthawi zina  Kawirikawiri  Pafupifupi nthawi zonse  Refused to answer | Mu masabata yawiri ayo yajumpha, nkhanandi uli ako mwapulika kuwawa kwa mutu? | Palije  Nyengo zinyakhe  Kawirikawiri  Pafupifupi nyengo zose  Refused to answer | Record as responded. Read out the options if not using the visual aid. |
| *STOMACHPROB* | During the past 2 weeks, how much have you had stomach problems? | 0  1  2  3  6 | Not at all  Sometimes  Usually  Almost Always  Refused to answer | Mu masabata awiri apitawa, ndi mochuluka bwanji mwakhala ndi mavuto a mmimba? | Palibe  Nthawi zina  Kawirikawiri  Pafupifupi nthawi zonse  Refused to answer | Mu masabata yawiri ayo yajumpha, nkhanandi uli ako mwawa na masugzo ya mnthumbo? | Palije  Nyengo zinyakhe  Kawirikawiri  Pafupifupi nyengo zose  Refused to answer | Record as responded. Read out the options if not using the visual aid. |
| *BODYPAINS* | During the past 2 weeks, how much have you had general body pains? | 0  1  2  3  6 | Not at all  Sometimes  Usually  Almost Always  Refused to answer | Mu masabata awiri apitawa, ndi mochuluka bwanji mwakhala ndi vuto lakuphwanya kwa mthupi? | Palibe  Nthawi zina  Kawirikawiri  Pafupifupi nthawi zonse  Refused to answer | Mu masabata yawiri ayo yajumpha, nkhanandi uli ako mwawa na masuzgo ya kuphwanya mthupi? | Palije  Nyengo zinyakhe  Kawirikawiri  Pafupifupi nyengo zose  Refused to answer | Record as responded. Read out the options if not using the visual aid. |
| ***PC-PTSD*** | **PC-PTSD** |  |  | **PC-PTSD** |  | **PC-PTSD** |  |  |
| *EVNTSLST* | [STOP USING VISUAL AID]   Sometimes things happen to people that are unusually or especially frightening, horrible, or traumatic. For example:   • a serious accident or fire • a physical or sexual assault or abuse • [WOMEN ONLY] a very difficult childbirth when you thought that you or the baby would die • an earthquake or flood • a war • seeing someone be killed or seriously injured • having a loved one die through homicide or suicide |  |  | [STOP USING VISUAL AID]   Nthawi zina zinthu zodabwitsa, zoopsya kwambiri, zoyipa, komanso zowawitsa mtima zimawachitikira anthu. Zinthu monga: • Ngozi yowopsya kapena moto • Kumenyedwa, nkhanza zogonana kapena nkhanza ya mtundu wina uliwonse • [WOMEN ONLY] nthawi yovuta kwambiri yakubereka yomwe munaganizirapo kuti inu kapena mwana wanu amwalira • chivomerezi kapena kusefukira kwa madzi • Nkhondo • kuwona winawake akuphedwa kapena atavulara kwambiri • kukhala ndi wachibale yemwe anazipha yekha kapena kuphedwa ndi anthu ena |  | [STOP USING VISUAL AID]   Nyengo zinyakhe vinthu vyakuziziswa, vyakofya chomene, viheni, kweniso vyakuwawiska mtima vikuwachitikira wanthu. Vinthu nge:  • Ngozi yakofya panji moto • Kutimbika panji kukorereka panji nkhaza zakugonana na nkhanza zinyakhe • [WOMEN ONLY] kusuzgika chomene pa nyengo ya kubaba apo mukaghanaghana kuti imwe panji mwana winu wafwenge • chindindindi panji kusefukira kwa maji • nkhondo • Kuona munthu wakukomeka panji wakupwetekeka chomene • Kuwa na m'bale wakuti walikujikoma yekha panji kukomeka na wanthu wanyakhe |  | These questions ask about past trauma. Take the visual aid from the participant. Read out the introductory speech as written. |
| *EXPEVNT* | Have you ever experienced this kind of traumatic event? | 1  0  8  6 | Yes  No  Don't Know  Refused to answer | Kodi zinthu monga izi zinakuchitikiranipo? | Eya  Ayi  Sindikudziwa  Refused to answer | Kasi vinthu nge ivi vilikumchitikiranipo? | Enya  Yayi  Nkhumanya yayi  Refused to answer | Record as responded. |
| *EVNTSNT_LABELS* | What traumatic event or events happened to you? | 1  0  8  6 | Yes  No  Don't Know  Refused to answer | Kodi zinachitika ndi chani? | Eya  Ayi  Sindikudziwa  Refused to answer | Kasi chikachitika ntchivichi? | Enya  Yayi  Nkhumanya yayi  Refused to answer | This question is asked if the participant has experienced trauma before. Ask and select from the list of questions below. |
| *ACCFIRE* | a serious accident or fire | 1  0  8  6 | Yes  No  Don't Know  Refused to answer | ngozi yowopsya kapena moto | Eya  Ayi  Sindikudziwa  Refused to answer | ngozi yakofya panji moto | Enya  Yayi  Nkhumanya yayi  Refused to answer | Record as responded. |
| *ABUSE* | a physical or sexual assault or abuse | 1  0  8  6 | Yes  No  Don't Know  Refused to answer | kumenyedwa, nkhanza zogonana kapena nkhanza ya mtundu wina uliwonse | Eya  Ayi  Sindikudziwa  Refused to answer | kutimbika, panji nkhaza zakugonana na nkhanza zinyakhe | Enya  Yayi  Nkhumanya yayi  Refused to answer | Record as responded. |
| *DIFBRTH* | a very difficult childbirth when you thought that you or the baby would die | 1  0  8  6 | Yes  No  Don't Know  Refused to answer | nthawi yovuta kwambiri yakubereka yomwe munaganizirapo kuti inu kapena mwana wanu amwalira | Eya  Ayi  Sindikudziwa  Refused to answer | kusuzgika chomene pa nyengo ya kubaba apo mukaghanaghana kuti imwe panji mwana winu wafwenge | Enya  Yayi  Nkhumanya yayi  Refused to answer | Record as responded. |
| *EQUAKFLD* | an earthquake or flood | 1  0  8  6 | Yes  No  Don't Know  Refused to answer | chivomerezi kapena kusefukira kwa madzi | Eya  Ayi  Sindikudziwa  Refused to answer | chindindindi panji kusefukira kwa maji | Enya  Yayi  Nkhumanya yayi  Refused to answer | Record as responded. |
| *WAR* | a war | 1  0  8  6 | Yes  No  Don't Know  Refused to answer | nkhondo | Eya  Ayi  Sindikudziwa  Refused to answer | nkhondo | Enya  Yayi  Nkhumanya yayi  Refused to answer | Record as responded. |
| *KILLEDINJUR* | seeing someone be killed or seriously injured | 1  0  8  6 | Yes  No  Don't Know  Refused to answer | kuwona winawake akuphedwa kapena atavulara kwambiri | Eya  Ayi  Sindikudziwa  Refused to answer | kuwona munthu wakukomeka panji wakupwetekeka chomene | Enya  Yayi  Nkhumanya yayi  Refused to answer | Record as responded. |
| *HSCIDE* | having a loved one die through homicide or suicide | 1  0  8  6 | Yes  No  Don't Know  Refused to answer | kukhala ndi wachibale yemwe anazipha yekha kapena kuphedwa ndi anthu ena | Eya  Ayi  Sindikudziwa  Refused to answer | Kuwa na m’bale wakuti walikujikoma yekha panji kukomeka na wanthu wanyakhe | Enya  Yayi  Nkhumanya yayi  Refused to answer | Record as responded. |
| *EVNTOTH* | other | 1  0  8  6 | Yes  No  Don't Know  Refused to answer | zina | Eya  Ayi  Sindikudziwa  Refused to answer | vinyakhe | Enya  Yayi  Nkhumanya yayi  Refused to answer | Record if other. |
| *EVNTOTHSPEC* | other, specify |  |  | ngati pali zina tchulani |  | vinyakhe longosolani |  | Specify if other. |
| ***EVENTS*** |  |  |  |  |  |  |  | The following questions are asked if a participant has experienced any traumatic event from the previous section. |
| *NIGHTMRS* | In the past month, have you had nightmares about the event(s) or thought about the event(s) when you did not want to? | 1  0  8  6 | Yes  No  Don't Know  Refused to answer | Mu mwezi wathawu, mwakhalapo ndi maloto kapena maganizo oyipa komanso obwerezabwereza okhudzana ndi zomwe zinakuchitikirani ngakhale inu simumafuna kuganizira zimenezo? | Eya  Ayi  Sindikudziwa  Refused to answer | Mu mwezi wajumpha uwu, mwakhalapo na maloto panji maghanoghano yaheni ya icho/ivyo vikamuchitikirani nanga uli imwe mukukhumba yayi kuvighanaghanira? | Enya  Yayi  Nkhumanya yayi  Refused to answer | Record as responded. |
| *AVOID* | In the past month, have you tried hard not to think about the event(s) or went out of your way to avoid situations that reminded you of the event(s)? | 1  0  8  6 | Yes  No  Don't Know  Refused to answer | Mu mwezi wathawu, mwakhala mukudziletsa kwambiri kuganizira zomwe zinachitika kapena kuyesayesa kuzemba zinthu, malo, kapena zochitika zomwe zingakukumbutseni za zomwe zinakuchitikirani? | Eya  Ayi  Sindikudziwa  Refused to answer | Mu mwezi wajumpha uwu, mwayezgapo chomene kujikanizga kughanaghanira vya ivyo vikachitika panji kuyezgeska kugwentha malo panji vinthu ivyo vikumukumbuskani ivo vikachitika? | Enya  Yayi  Nkhumanya yayi  Refused to answer | Record as responded. |
| *ONGRD* | In the past month, have you been constantly on guard, watchful, or easily startled? | 1  0  8  6 | Yes  No  Don't Know  Refused to answer | Mu mwezi wathawu, ndikawirikawiri mwakhala ndi mantha, tcheru ngati chinachake chichitika, kapena odzidzimutsika mosavuta? | Eya  Ayi  Sindikudziwa  Refused to answer | Mu mwezi wajumpha uwu, mwakhala wa tcheru chomene kweniso wakuchiluskika mwaluwiro? | Enya  Yayi  Nkhumanya yayi  Refused to answer | Record as responded. |
| *NUMB* | In the past month, have you felt numb or detached from people, activities, or your surroundings? | 1  0  8  6 | Yes  No  Don't Know  Refused to answer | Mu mwezi wathawu, mwakhala mukumva mumtima mwanu ngati kuti palibe kulumikizana ndi anthu, zochitika, kepana malo omwe muli? | Eya  Ayi  Sindikudziwa  Refused to answer | Mu mwezi wajumpha uwu, mwapulikapo ngati kuti thupi lati waka zii kweniso kupulika ngati mwatalikirana na wanthu, vakuchitika vya mdera linu, kweniso malo ayo mukukhala? | Enya  Yayi  Nkhumanya yayi  Refused to answer | Record as responded. |
| *GUILTY* | In the past month, have you felt guilty or unable to stop blaming yourself or others for the event(s) or any problems the event(s) may have caused? | 1  0  8  6 | Yes  No  Don't Know  Refused to answer | Mu mwezi wathawu, mwakhala mu kuziona olakwa komanso kukanika kusiya kuziona inu kapena ena kuti ndi olakwa chifukwa cha zomwe zinakuchitikirani kapena mavuto amene abwera chifukwa cha zimenezi? | Eya  Ayi  Sindikudziwa  Refused to answer | Mu mwezi wajumpha uwu, mwakhala mukujiyeruzga panji kutondeka kuleka kujiyeruzga mwawene panji wanthu wanyakhe chifukwa cha ivyo vikachitika panji masuzgo ayo yiza chifukwa cha ivi? | Enya  Yayi  Nkhumanya yayi  Refused to answer | Record as responded. |
| ***SUICIDESCREEN*** | **Suicide Screening** |  |  |  |  |  |  |  |
| *SUICIDERISKNOTE* | Read the following to participant: You said that, in the last two weeks, you have had thoughts that life is not worth living or of hurting yourself in some way. I am sorry to hear that. I would like to ask you a few more questions about those thoughts. |  |  | Read the following to participant: Munanena kuti mu sabata ziwiri zapitazi, mwakhalapo ndi maganizo akuti kulibwino mungofa kapena ofuna kuzivulaza nokha munjira ina yake. Ndine okhudzidwa kumva choncho. Ndikufuna ndikufunseni mafunso ena ochepa okhudza maganizo amenewa. |  | Read the following to participant: Mwanguyowoya kuti mu sabata ziwiri zajumpha, mwawapo na maghanoghano yakuti chingaba makola kufwa panji kujipweteka mwekha mu nthowa yinyakhe. Vyanikhwaska kuti vili nthena. Nkhukhumba kumufumbani mafumbo yanyakhe ghachoko ghakukhwaskana na maghanoghano agha. |  | These questions aim to screen participants at risk of suicide. Suicide screening is done for participants who have responded to have had thoughts that they would be better off dead or thoughts of hurting themselves in some way. Read the note to the participant as in the script. |
| *SUICIDERISK1* | Can I check, in the last 2 weeks have you had these thoughts many times or just once or twice? | 1  0  6 | Many times  Once or twice  Refused to answer | Mu masabata awiri apitawa, maganizowa mwakhala nawo kangati: kambiri, kapena "kamodzi kapena kawiri"? | Many times  Once or twice  Refused to answer | Mu masabata yaŵiri ayo yajumpha, kasi maghanoghano aya mwawanagho kalinga: kanandi, panji "kamoza panji kawiri"? | Many times  Once or twice  Refused to answer | Record as responded. |
| *SUICIDERISK2* | In the last 2 weeks, have you made any plans or considered a method that you might use to end your life or harm yourself? | 1  0  6 | Yes  No  Refused to answer | Mu masabata awiri apitawa, munapangako dongosolo kapena kulingalira njira yofuna kuzipha kapena kuzivulaza? | Eya  Ayi  Refused to answer | Mu masabata yaŵiri ayo yajumpha, kasi muli kupangapo nthowa yiliyose yakujikomera panji kughanaghana nthowa iyo mungagwiriska ntchito kuti mujipweteke mwaŵene? | Enya  Yayi  Refused to answer | Record as responded. |
| *SUICIDERISK3* | Have you ever attempted to harm yourself or end your life? | 1  0  6 | Yes  No  Refused to answer | Munayamba mwayeserapo kuzipweteka nokha kapena kufuna kuzipha nokha? | Eya  Ayi  Refused to answer | Kasi mulikuyezgapo kujipweteka mwabene panji kujikoma mwekha? | Enya  Yayi  Refused to answer | Record as responded. |
| *SUICIDERISK4* | Do you think there is any risk that you might harm yourself or end your life now or in the near future? | 1  0  6 | Yes  No  Refused to answer | Kodi mukuwona ngati muli pachiwopsyezo choti mutha kuzipha kapena kuzivulaza nokha pakali pano kapena posachedwapa? | Eya  Ayi  Refused to answer | Kasi mukughanaghana kuti ulipo ulwani wakuti mungajikoma panji mungajipweteka mwabene pa nyengo yasono panji mwasonosono? | Enya  Yayi  Refused to answer | Record as responded. |
| *CONTINT* | Read the following to the participant: Because you have had some thoughts that you would be better off dead or of hurting yourself in some way, at the end of the questionnaire, I will explain how you can get help and support. Do you feel able to continue with the rest of the interview first? | 1  0 | Yes  No | Read the following to the participant: Chifukwa chikuti mwakhalapo ndi maganizo akuti kulibwino mungofa kapena ofuna kuzivulaza nokha munjira ina yake, pamapeto pamafunsowa ndikufotokozerani za momwe mungapezere chithandizo. Kodi pakali pano mutha kupiliza kuyankha mafunso otsarawa? | Eya  Ayi | Read the following to the participant: Chifukwa chikuti mwawapo na maghanoghano yakuti chingaba makola kufwa panji kujipweteka mwekha mu nthowa yinyakhe, pawumaliro pa mafumbo aya ndimulongosoleraninge umo mungasangila wovwiri. Kasi tingalutilira kumufumbani mafumbo yakhalako? | Enya  Yayi | This note is for participants that are at risk of suicide. Read note exactly as written and inquire if participant is willing to continue with interview. If yes, the tablet will remind you to carryout appropriate actions at the end of the interview. If not, skip to safety action as described in “SUICIDESAFETY_NOTE “ |
| *CONTSKIP* | IF YES, continue with interview. You will be reminded by the tablet to carry out the appropriate actions at the end of the interview. IF NO, skip to safety action section. |  |  | IF YES, continue with interview. You will be reminded by the tablet to carry out the appropriate actions at the end of the interview. IF NO, skip to safety action section. |  | IF YES, continue with interview. You will be reminded by the tablet to carry out the appropriate actions at the end of the interview. IF NO, skip to safety action section. |  |  |
| *SSINTRO* | Now I am going to ask you some questions about the past. Don’t answer about how you have felt in the last month, but think about ANY time in your life before that. |  |  | Pano ndikufunsani mafunso okhudza nthawi ya m’mbuyomu. Musayankhe za mmene mumamvera mwezi watha koma muganizire za nthawi ya m’mbuyo m’moyo wanu kuposera mwezi watha. |  | Sono ndikufumbaninge mafumbo yanyake yakukhwaskana na nyengo yakumanyuma. Mungazgolanga yayi umo mwangujipulikira mumwezi umoza uwo wamala kweni mughanaghanire zanyengo yakumanyuma mumoyo winu kujumpha mwezi wamala. |  | These questions are about past events. Read note exactly as written. Emphasize to the participant that these questions are about ANY time in their life. |
| **GMH** | **MENTAL HEALTH** |  |  | **MENTAL HEALTH** |  | **MENTAL HEALTH** |  |  |
| *INTRO* | I am now going to ask you some questions about how you have been thinking and feeling during the past two weeks.   There are 4 possible answers for each question:   “Not at all”, means you have not had the symptom at all in the last 2 weeks. “Sometimes”, means you have had the symptom but less than half the days in the past two weeks. “Usually”, means you have had the symptom on more than half the days but not all the time in the past two weeks. Finally, “Almost always” means you have had the symptom every day, or almost every day.   [IF YOU THINK THAT THE PARTICIPANT WOULD BENEFIT FROM USING THE WATER GLASSES VISUAL AID, SHOW IT TO THE PARTICIPANT AND GIVE THE FOLLOWING INSTRUCTIONS]  [Point at empty glass] “Not at all”, means you have not had the symptom at all in the last 2 weeks. [Point at 1/3 full glass] “Sometimes”, means you have had the symptom but less than half the days in the past two weeks. [Point at 2/3 full glass]. “Usually”, means you have had the symptom on more than half the days but not all the time in the past two weeks. [Point at full glass], Finally “Almost always” means you have had the symptom every day, or almost every day. |  |  | Pano ndikufunsani mafunso okhudzana ndi momwe mwakhala mukuganizira ndi kumvera mu mtima mwanu mu masabata awiri apitawa.   Pali mayankho anayi pafunso lina lililonse:    Yankho loti “Palibe” likutanthauza kuti simunakhalepo ndi vuto limene mwafunsidwa mu masabata awiri apitawa. Yankho loti “Nthawi zina” likutanthauza kuti mwakhala ndi vutoli koma kochepera theka (hafu) la masiku a masabata awiri apitawa. Yankho loti “Kawirikawiri” likutanthauza kuti mwakhala ndi vutoli koposera theka (hafu) la masabata awiri apitawa, koma osati tisku ndi tsiku. Yankho lomalizira ndi loti “Pafupifupi nthawi zonse”, limene likutanthauza kuti mwakhala ndi vuto limene mwafunsidwali tsiku lililonse, kapena pafupifupi tsiku lilonse.   [IF YOU THINK THAT THE PARTICIPANT WOULD BENEFIT FROM USING THE WATER GLASSES VISUAL AID, SHOW IT TO THE PARTICIPANT AND GIVE THE FOLLOWING INSTRUCTIONS]  [Point at empty glass] Yankho loti “Palibe” likutanthauza kuti simunakhalepo ndi vuto limene mwafunsidwa mu masabata awiri apitawa. [Point at 1/3 full glass]. Yankho loti “Nthawi zina” likutanthauza kuti mwakhala ndi vutoli koma kochepera theka (hafu) la masiku a masabata awiri apitawa. [Point at 2/3 full glass] Yankho loti “Kawirikawiri” likutanthauza kuti mwakhala ndi vutoli koposera theka (hafu) la masabata awiri apitawa, koma osati tisku ndi tsiku. [Point at full glass] Yankho lomalizira ndi loti “Pafupifupi nthawi zonse”, limene likutanthauza kuti mwakhala ndi vuto limene mwafunsidwali tsiku lililonse, kapena pafupifupi tsiku lilonse. |  | Sono nikufumbaninge mafumbo yakukhwaskana na umo mwakhala mukughanaghanira na kupulikira mu mtima winu mu masabata yawiri aya yajumpha.   Pali mazgolo yanayi pafumbo lililose:  Zgolo lakuti “Palije” likungánamula kuti mundawepo na suzgo ilo mwafumbika mu masabata yawiri yajumpha. Zgolo lakuti “Nyengo zinyakhe” likungánamula kuti mwawa na sugzo ili kweni kwakuchepera hafu ya mazuwa ya mu masabata yawiri yajumpha. Zgolo lakuti “Kawirikawiri” likungánamula kuti mwawa na sugzo ili kwakujumpha hafu ya masabata yawiri yajumpha, kweni dazi na dazi yayi. Zgolo lakuti “Pafupifupi nyengo zose” likungánamula kuti mwawa na suzgo ilo mwafumbika kwa dazi lililose, panji pafupifupi dazi lililose.   [IF YOU THINK THAT THE PARTICIPANT WOULD BENEFIT FROM USING THE WATER GLASSES VISUAL AID, SHOW IT TO THE PARTICIPANT AND GIVE THE FOLLOWING INSTRUCTIONS]  [Point at empty glass] Zgolo lakuti “Palije” likungánamula kuti mundawepo na suzgo ilo mwafumbika mu masabata yawiri yajumpha. [Point at 1/3 full glass]. Zgolo lakuti “Nyengo zinyakhe” likungánamula kuti mwawa na sugzo ili kweni kwakuchepera hafu ya mazuwa ya mu masabata yawiri yajumpha. [Point at 2/3 full glass]. Zgolo lakuti “Kawirikawiri” likungánamula kuti mwawa na sugzo ili kwakujumpha hafu ya masabata yawiri yajumpha, kweni dazi na dazi yayi. [Point at full glass] Zgolo lakuti “Pafupifupi nyengo zose” likungánamula kuti mwawa na suzgo ilo mwafumbika kwa dazi lililose, panji pafupifupi dazi lililose. |  | This section asks participants on mental health symptoms they have been experiencing in the past two weeks. The interviewer will also assess whether the participant can use the water glass visual aids and explain it to them as written in the introductory script. If the interviewer thinks the participant will not benefit from using the visual aid, (s)he should read out the four options to the participant.  Each question has a note of an example of how a question should be rephrased if the participant does not understand it. Interviewer should read this slowly and as exactly as written |
| *PHQ9* | PHQ9 |  |  | PHQ9 |  | PHQ9 |  |  |
| *FEELDOWN* | During the past two weeks, how much have you been feeling down, depressed, or hopeless? | 0  1  2  3  6 | Not at all  Sometimes  Usually  Almost Always  Refused to answer | Mu masabata awiri apitawa, ndi mochuluka bwanji pamene mwakhala osasangalala, wokhumudwa, kapena opanda chiyembekezo? | Palibe  Nthawi zina  Kawirikawiri  Pafupifupi nthawi zonse  Refused to answer | Mu masabata yawiri ayo yajumpha, nkhanandi uli ako mwawa wambula kukondwa, kukwebeka, panji kuwavya chigomezgo? | Palije  Nyengo zinyakhe  Kawirikawiri  Pafupifupi nyengo zose  Refused to answer | Record as responded. Read out the options if not using the visual aid.  Read note if participant does not understand question. |
| *FEELDOWN_NOTE* | [IF THE PARTICIPANT IS NOT SURE WHAT THE QUESTION MEANS, READ THIS EXAMPLE AS IT IS WRITTEN]  Sometimes a person feels miserable because of something in their life or even for no clear reason; or feels that nothing they do will make life better. How much has this happened to you in the last 2 weeks? |  |  | [IF THE PARTICIPANT IS NOT SURE WHAT THE QUESTION MEANS, READ THIS EXAMPLE AS IT IS WRITTEN]  Nthawi zina munthu amatha kukhala wokhumudwa chifukwa cha chinthu china m’moyo mwake kapenanso opanda chifukwa chimene; komanso amatha kukhala ndi maganizo akuti palibe chomwe angachite kuti akometse moyo wake. Ndi mochuluka bwanji zimenezi zinakuchitikirani mu masabata awiri apitawa? |  | [IF THE PARTICIPANT IS NOT SURE WHAT THE QUESTION MEANS, READ THIS EXAMPLE AS IT IS WRITTEN]  Ngengo zinyake munthu wakujipulika wambula kukondwa na ivyo vikuchitika pa moyo wakhe nanga pangaba chifukwa chambula kupulikwika pakunji palije icho bangachita chakuti chipangiske moyo wabo wube makora. Nkhanandi uli ivi vyamuchitikiranipo mumasabata yawiri ayo yajumpha? |  |  |
| *ANHEDONIA* | During the past two weeks, how much have you had little interest or pleasure in doing things? | 0  1  2  3  6 | Not at all  Sometimes  Usually  Almost Always  Refused to answer | Mu masabata awiri apitawa, ndi mochuluka bwanji pamene mwakhala ndi chidwi chochepa kapena osasangalatsidwa pochita zinthu? | Palibe  Nthawi zina  Kawirikawiri  Pafupifupi nthawi zonse  Refused to answer | Mu masabata yawiri ayo yajumpha, nkhanandi uli ako mwawa wa kunweka kwa pachoko panji kukhala wambula kusangwa pakuchita vinthu? | Palije  Nyengo zinyakhe  Kawirikawiri  Pafupifupi nyengo zose  Refused to answer | Record as responded. Read out the options if not using the visual aid. |
| *ANHEDONIA_NOTE* | [IF THE PARTICIPANT IS NOT SURE WHAT THE QUESTION MEANS, READ THIS EXAMPLE AS IT IS WRITTEN]   Sometimes a person gets no pleasure or has no interest when doing things such as cooking, working or meeting with friends, even though he/she used to enjoy those things. How much has this happened to you in the last 2 weeks? |  |  | [IF THE PARTICIPANT IS NOT SURE WHAT THE QUESTION MEANS, READ THIS EXAMPLE AS IT IS WRITTEN]  Nthawi zina munthu amatha kukhala opanda chidwi kapena osasangalatsidwa pochita zinthu monga kuphika, kugwira ntchito kapena kukumana ndi anzake, ngakhale m’mbuyomu anali kusangalatsidwa nazo. Ndi mochuluka bwanji zimenezi zinakuchitikirani mu masabata awiri apitawa? |  | [IF THE PARTICIPANT IS NOT SURE WHAT THE QUESTION MEANS, READ THIS EXAMPLE AS IT IS WRITTEN]   Nyengo zinyake munthu wakuwa wambula kusangwa panji wambula kunweka navinthu ivyo wakatemwanga kupanga ngati kuphika, kugwira tchito panji kuchezga na wabwezi, nangauli kale wakasanguluskikanga navinthu vyeneivyi. Nkhanandi uli ivi vyamuchitikiranipo mumasabata yawiri ayo yajumpha? |  | Read note if participant does not understand question. |
| *PROBSLEEP* | During the past two weeks, how much have you had trouble falling or staying asleep, or sleeping too much? | 0  1  2  3  6 | Not at all  Sometimes  Usually  Almost Always  Refused to answer | Mu masabata awiri apitawa, ndi mochuluka bwanji pamene mwakhala mukuvutika kupeza tulo kapena kugona mosakwanira, kapenanso kugona kwambiri? | Palibe  Nthawi zina  Kawirikawiri  Pafupifupi nthawi zonse  Refused to answer | Mu masabata yawiri ayo yajumpha, nkhanandi uli ako mwawa wa kutondeka kusanga tulo panji kugona mwambula kukwanira, panji kugona chomene? | Palije  Nyengo zinyakhe  Kawirikawiri  Pafupifupi nyengo zose  Refused to answer | Record as responded. Read out the options if not using the visual aid. |
| ***PASTDEP*** | **Past Depression** |  |  | **Past Depression** |  | **Past Depression** |  |  |
| *FEELDOWN1MO* | Have you ever had a time in the past, before the last 1 month, when you felt down, depressed, or hopeless, lasting for two weeks or more? | 1  0  8  6 | Yes  No  Don't Know  Refused to answer | Munakhalapo ndi nthawi m’mbuyomu kupatula mwezi umodzi wathawu yomwe munakhala okhumudwa, a nkhawa, komanso opanda chiyembekezo kwa ma sabata awiri kapena kuposera? | Eya  Ayi  Sindikudziwa  Refused to answer | Kasi mumanyuma umu, kupatula mwezi wamala muli kuwapo na nyengo yakuti mukawa bakukwebeka, bakukwenyerera, kweniso bambula chigomezgo kwa masabata yawiri panji kujumpha? | Enya  Yayi  Nkhumanya yayi  Refused to answer | Record as responded. |
| *ANHEDONIA1MO* | Have you ever had a time in the past, before the last 1 month, when you lost interest in most things like hobbies, work, or activities that usually give you pleasure, lasting for two weeks or more? | 1  0  8  6 | Yes  No  Don't Know  Refused to answer | Munakhalapo ndi nthawi m’mbuyomu kupatula mwezi umodzi wathawu yomwe munasoweka chidwi mu zinthu zambiri zomwe mumakonda monga masewero, ntchito, kapena zinthu zoti zimakusangalatsani, kwa nthawi yokwana masabata awiri kapena kuposera? | Eya  Ayi  Sindikudziwa  Refused to answer | Mulikuwapo na nyengo mumanyuma umu kupatula mwezi wamala yakuti mukasoweka kunweka pakuchita vinthu vinandi vyakuti mukatemwanga kuchita, vinthu nge masewero, ntchito, na vyakuchita vinyake vyakuti vikumukondweskani, kwa masabata yawiri panji kujumpha? | Enya  Yayi  Nkhumanya yayi  Refused to answer | Record as responded. |
| *DURPREG* | Did you have an episode like the one you just mentioned during a previous pregnancy? | 1  0  8  6 | Yes  No  Don't Know  Refused to answer | Kodi zinthu ngati zimene ndakufunsani pamwambazi zinakuchitikiranipo pa mimba zam’mbuyomu? | Eya  Ayi  Sindikudziwa  Refused to answer | Kasi vinthu nge ivi tadumbilana pachanya apo vili kumuchitikiranipo pa nthumbo za mumanyuma? | Enya  Yayi  Nkhumanya yayi  Refused to answer | *For women only:* This question will be asked to women who said yes to any of the questions above. Record as responded. |
| *FEWMOBIRTH* | Did you have an episode like the one you just mentioned within a few months of giving birth? | 1  0  8  6 | Yes  No  Don't Know  Refused to answer | Kodi zinthu ngati zimene ndakufunsani pa mwambazi zinakuchitikiranipo patatha miyezi yochepa mutangobereka? | Eya  Ayi  Sindikudziwa  Refused to answer | Kasi vinthu nge ivi tadumbirana pachanya ivi vili kumuchitikiranipo pa miyezi ichoko mwati mwababa waka? | Enya  Yayi  Nkhumanya yayi  Refused to answer | *For women only:* This question will be asked to women who said yes to any of the questions above. Record as responded. |
| *PASTDEPDIF* | When you had the episode you just mentioned, how difficult was it for you to do your work, take care of things at home or get along with other people? [READ THE OPTIONS]? | 0  1  2  3  6 | Not difficult at all  Somewhat difficult  Very difficult  Extremely difficult  Refused to answer | Kodi nthawi imene zinthu ndakufunsani pamwambazi zinachitika, munabvutika bwanji kugwira ntchito zanu, kusamala pakhomo kapena kukhala bwino ndi anthu ena [READ THE OPTIONS]? | Sindinavutikepo  Pang’ono pokha  Mochuluka  Mochuluka kwambiri  Refused to answer | Panyengo iyo ivi mwazunuila vikamuchitikilani, Kasi vinthu ivi vikiza na maunonono wuli pa umo mukugwirira ntchito, kupwererera vinthu pa nyumba, panji ubale winu na wanthu [READ THE OPTIONS]? | Nindasuzgikepo  Pachoko waka  Nkhasuzgika chomene  Nkhasuzgika chomene nkhanira  Refused to answer | This question will be asked to anyone who said yes to any of the questions above. Read out the options and record as responded. |
| *PASTMANIA* | **Past possible manic symptoms** |  |  | **Past possible manic symptoms** |  | **Past possible manic symptoms** |  |  |
| *NOTSELFEVER* | Has there ever been a period of time when you were feeling so good, “high,” excited, or “on top of the world” that your family or friends thought you were not your normal self? | 1  0  8  6 | Yes  No  Don't Know  Refused to answer | Inalipo nthawi inayake mbuyomo yoti inu mumamva kukondwa kwambiri ngati kuti mwapambana kwambiri mumoyo wanu mwakuti abale anu kapena anzanu amaona ngati kuti simuli bwino kapena simuli monga momwe mumakhalira nthawi zonse? | Eya  Ayi  Sindikudziwa  Refused to answer | Yilikuwapo nyengo yakuti imwe mukapulikanga kukondwa chomene, kusangwa, nge kuti mwadangira chomene mumoyo winu mwakuti wabale panji wanyinu wakaonanga kuti muli umo mukubira nyengo zose yayi? | Enya  Yayi  Nkhumanya yayi  Refused to answer | Record as responded. |
| *IRRITABLEEVER* | Have you ever had a period of time when you were feeling irritable, angry, or short-tempered for most of the day, every day, for at least several days, so that your family or friends thought you were not your normal self? | 1  0  8  6 | Yes  No  Don't Know  Refused to answer | Munakhalapo ndi nthawi yomwe mumakwiya mosavuta kapena kupsa mtima msanga pafupifupi tsiku lonse, tsiku liri lonse, kwa masiku ambiri, mwakuti abale anu kapena anzanu amaona ngati kuti simuli bwino kapena simuli monga momwe mumakhalira nthawi zonse? | Eya  Ayi  Sindikudziwa  Refused to answer | Mulikuwapo na nyengo iyo mukakalipanga panji mukakwiyanga mwaluwiro pafupifupi zuwa lose, zuwa lili lose, kwa mazuwa yanandi, mwakuti wabale panji wanyinu wakawonanga kuti muli umo mukubira nyengo zose yayi? | Enya  Yayi  Nkhumanya yayi  Refused to answer | Record as responded. |
| *STRTAFTBIRTH* | Did you have an episode like the one you just mentioned that started soon after giving birth? | 1  0  8  6 | Yes  No  Don't Know  Refused to answer | Kodi zinthu ngati zimene ndakufunsani pamwambapo zinakuchitikiranipo mutangobereka kumene? | Eya  Ayi  Sindikudziwa  Refused to answer | Kasi vinthu nge ivi tadumbilana pacha apo vikamuchitikilanipo mu nyengo yakuti mwababa waka? | Enya  Yayi  Nkhumanya yayi  Refused to answer | *For women only:* This question will be asked to women who said yes to any of the questions above. Record as responded. |
| *PASTMANIADIF* | When you had the episode you just mentioned, how difficult was it for you to do your work, take care of things at home or get along with other people? [READ THE OPTIONS]? | 0  1  2  3  6 | Not difficult at all  Somewhat difficult  Very difficult  Extremely difficult  Refused to answer | Kodi nthawi imene zinthu ndakufunsani pamwambazi zinachitika, munabvutika bwanji kugwira ntchito zanu, kusamala pakhomo kapena kukhala bwino ndi anthu ena [READ THE OPTIONS]? | Sindinavutikepo  Pang’ono pokha  Mochuluka  Mochuluka kwambiri  Refused to answer | Panyengo iyo ivi mwazunuila vikamuchitikilani, Kasi vinthu ivi vikiza na maunonono wuli pa umo mukugwirira ntchito, kupwererera vinthu pa nyumba, panji ubale winu na wanthu [READ THE OPTIONS]? | Nindasuzgikepo  Pachoko waka  Nkhasuzgika chomene  Nkhasuzgika chomene nkhanira  Refused to answer | This question will be asked to anyone who said yes to any of the questions above. Read out the options and record as responded. |
| ***PASTDEP*** | **Past Depression** |  |  | **Past Depression** |  | **Past Depression** |  |  |
| *FEELDOWN1MO* | Have you ever had a time in the past, before the last 1 month, when you felt down, depressed, or hopeless, lasting for two weeks or more? | 1  0  8  6 | Yes  No  Don't Know  Refused to answer | Munakhalapo ndi nthawi m’mbuyomu kupatula mwezi umodzi wathawu yomwe munakhala okhumudwa, a nkhawa, komanso opanda chiyembekezo kwa ma sabata awiri kapena kuposera? | Eya  Ayi  Sindikudziwa  Refused to answer | Kasi mumanyuma umu, kupatula mwezi wamala muli kuwapo na nyengo yakuti mukawa bakukwebeka, bakukwenyerera, kweniso bambula chigomezgo kwa masabata yawiri panji kujumpha? | Enya  Yayi  Nkhumanya yayi  Refused to answer | Record as responded. |
| ***ASSISTQ1*** | **ASSIST Q 1** |  |  | **ASSIST Q 1** |  | **ASSIST Q 1** |  |  |
| *ASSISTQ1_INTRO* | I am now going to ask you some questions about alcohol, tobacco products and other drugs. Some of these substances may be prohibited by law, but please be assured that all information we will collect will be kept private and confidential. |  |  | Ndikufunsani mafunso okhudzana ndi mowa, fodya, ndi mankhwala ena.    Ena mwa mankhwala antha kukhala oletsedwa, chonde dziwani kuti uthenga omwe utengedwe ukasungida motetezedwa ndi mwa chinsinsi. |  | Sono ndimufumbaninge mafumbo yakukhwaskana na mowa, hona, na mankhwala yanyakhe.   Yanyakhe mwa mankhwala aya yangaba yakukanizgika, chonde manyani kuti wanduzi wose uwo utolekenge wamusungika mwa kuvikilirika na mwa chisisi. |  | Read note exactly as written. Emphasize to participants about confidentiality of the information collected. If participants agree to taking any substance, confirm if they understand what the substance is. |
| *ALCEVER* | In your life, have you ever used alcohol? Examples of alcoholic beverages are bottled beer (Carlsberg), spirits, chibuku and locally brewed alcohol. | 1  0  8  6 | Yes  No  Don't Know  Refused to answer | Mu moyo wanu, munamwako mowa? Mu gulu la zokumwa zoledzeletsa (mowa), muli mowa wa m’botolo (monga Carlsberg, castel) mowa wa masacheti, maspiriti, wayini, Chibuku, kachasu, mowa wina wophikidwa mmudzi ndi mowa ina yosiyanasiyana. | Eya  Ayi  Sindikudziwa  Refused to answer | Mu moyo winu, muli kumwapo moba? Mu gulu la vyakumwa vyakulowereska (moba), muli moba wa ma botolo (nge Carlsberg, Castel), moba wa masacheti, masipiriti, wayini, Chibuku, kachasu, moba wakuphikika mchikaya, na moba inyakhe yakupambanapambana. | Enya  Yayi  Nkhumanya yayi  Refused to answer | Record as responded. |
| *TOBACEVER* | In your life, have you ever used tobacco products (e.g. cigarettes, chewing tobacco, kuber, cigars, labu etc.)? | 1  0  8  6 | Yes  No  Don't Know  Refused to answer | Mu moyo wanu munagwiritsapo ntchito zopangidwa kuchoka ku fodya (monga ndundu, fodya wotafuna/fodya wamphuno, kyuba, chikololo (paipi), chingambwe ndi zina zotero)? | Eya  Ayi  Sindikudziwa  Refused to answer | Pamoyo winu, kasi mulikugwiliskapo ntchito vyakupangika kufuma ku hona (nge ndudu, hona wakusumba, hona wamphuno, ngilimbo, kyuba, chikololo, chingambwe na vinyake)? | Enya  Yayi  Nkhumanya yayi  Refused to answer | Record as responded. |
| *CANNABISEVER* | In your life, have you ever used cannabis (marijuana, pot, grass, hash etc.)? | 1  0  8  6 | Yes  No  Don't Know  Refused to answer | Mu moyo wanu, munagwiritsapo ntchito chamba ndi zinthu zomwe zimapangidwa kuchokera ku chamba? | Eya  Ayi  Sindikudziwa  Refused to answer | Pamoyo winu, kasi mulikugwiliskapo ntchito chamba na vyakupangika kufuma ku chamba? | Enya  Yayi  Nkhumanya yayi  Refused to answer | Record as responded. |
| *COCAINEEVER* | In your life, have you ever used cocaine (coke, crack, etc.)? | 1  0  8  6 | Yes  No  Don't Know  Refused to answer | Mu moyo wanu, munagwiritsapo ntchito kokeni? | Eya  Ayi  Sindikudziwa  Refused to answer | Pamoyo winu, kasi mulikugwiliskapo ntchito kokeni? | Enya  Yayi  Nkhumanya yayi  Refused to answer | Record as responded. |
| *AMPHETEVER* | In your life, have you ever used amphetamine type stimulants? Names of these drugs include speed, ecstasy and tik. | 1  0  8  6 | Yes  No  Don't Know  Refused to answer | Mu moyo wanu, munagwiritsapo ntchito mankhwala ochangamutsa? Maina a makhwala awa ndimonga speed, ecstasy and tik. | Eya  Ayi  Sindikudziwa  Refused to answer | Pamoyo winu, kasi mulikugwiliskapo ntchito mankhwala yakuchentchemuska munthu? Mazina ya makhwala aya ningati speed, ecstasy and tik. | Enya  Yayi  Nkhumanya yayi  Refused to answer | Record as responded. |
| *INHALANTEVER* | In your life, have you ever used inhalants (glue, petrol, etc.)? | 1  0  8  6 | Yes  No  Don't Know  Refused to answer | Mu moyo wanu, munagwiritsapo ntchito zofwenthera (monga glue, petrol ndi zina zotero)? | Eya  Ayi  Sindikudziwa  Refused to answer | Pamoyo winu, kasi mulikugwiliskapo ntchito vyakukhweba (nge glue, petrol na vinyakhe)? | Enya  Yayi  Nkhumanya yayi  Refused to answer | Record as responded. |
| *SEDATIVEEVER* | In your life, have you ever used sedatives or sleeping pills more than you were prescribed by a doctor (e.g Valium or Diazepam)? | 1  0  8  6 | Yes  No  Don't Know  Refused to answer | Mu moyo wanu, munagwiritsapo ntchito mankhwala ogonetsa kuposera mulingo womwe anakulemberani adotolo (monga Valium kapena kuti Diazepam)? | Eya  Ayi  Sindikudziwa  Refused to answer | Pamoyo winu, kasi mulikugwiliskapo ntchito mankhwala yakwiziska tulo mwakujumphizga mulingo uwo wakamulembelani badokotala (nge Valium panji Diazepam)? | Enya  Yayi  Nkhumanya yayi  Refused to answer | Record as responded. |
| *HALLUCEVER* | In your life, have you ever used hallucinogens (magic mushrooms etc.)? | 1  0  8  6 | Yes  No  Don't Know  Refused to answer | Mu moyo wanu, munagwiritsapo ntchito mankhwala ena opangisa kuona zinthu zomwe anthu ena sakuziwona ndi maso awo (bowa wa poyizoni ndi zina zotero)? | Eya  Ayi  Sindikudziwa  Refused to answer | Pamoyo winu, kasi mulikugwiliskapo ntchito mankhwala yanyake ayo yakuwoneska vinthu vyakuti banthu banyakhe bakuviwona yayi na maso (vikanje na vinyakhe)? | Enya  Yayi  Nkhumanya yayi  Refused to answer | Record as responded. |
| *OPIOIDEVER* | In your life, have you ever used illegal opiods (e.g. heroin) or other opiods (e.g. morphine, pethidine, methadone, codeine, cough syrup with codeine etc) for reasons other than prescription, or taken them more frequently or at higher doses than prescribed by a doctor? | 1  0  8  6 | Yes  No  Don't Know  Refused to answer | Mu moyo wanu, munagwiritsapo ntchito mankhwala oletsedwa a heroin, kapena mankhwala amphamvu oletsa ululu waukulu monga morphine, pethidine, methadone, codeine, cough syrup wophatikizako codeine ndi zina zotero pa zifukwa zina kupatula zomwe anakulemberani adotolo kapena kumwa mowirikiza kapenanso kuposera mulingo omwe munapatsidwa? | Eya  Ayi  Sindikudziwa  Refused to answer | Pamoyo winu, kasi mulikugwiliskapo ntchito mankhwala yakukanizgika ya heroin panyakhe mankhwala yankhongono yakulekeska wurwirwi wukulu nge morphine, pethidine, methadone, codeine, cough syrup wakuskazikako codeine na vinyakhe pavifukwa vinyakhe vyakuperekeka na badokotala yayi, panji para mukamwa mankhwala aya mwakujumphizga ndondomeko? | Enya  Yayi  Nkhumanya yayi  Refused to answer | Record as responded. |
| *OTHEVER* | In your life, have you ever used any other illegal drug or used drugs for reasons other than prescription, or taken them more frequently or at higher doses than prescribed by a doctor? | 1  0  8  6 | Yes  No  Don't Know  Refused to answer | Mu moyo wanu, munagwiritsapo ntchito mankhwala ena aliwonse oletsedwa kapena kugwiritsa ntchito mankhwala pa zifukwa zina kupatula zomwe anakulemberani adotolo kapena kumwa mowirikiza kapenanso kuposera mulingo omwe munapatsidwa? | Eya  Ayi  Sindikudziwa  Refused to answer | Pamoyo winu, mulikugwiliskapo ntchito mankhwala yanyake yaliyose yakukanizgika panji kugwiliska mankhwala pavifukwa vinyakhe vyakuperekeka na badokotala yayi, panji para mukamwa mankhwala aya mwakujumphizga ndondomeko? | Enya  Yayi  Nkhumanya yayi  Refused to answer | Record as responded. |
| *OTHEVERSPEC* | If so, what was it? |  |  | Ngati eya, mankhwala anji? |  | Para enya, mankhwala uli? |  | Specify any other drug used if yes to above. |
| ***AUDIT*** | **AUDIT** |  |  | **AUDIT** |  | **AUDIT** |  |  |
| *INTRO* | Now I am going to ask you some questions about your use of alcoholic beverages during this past 12 months |  |  | Pano ndikufunsani mafunso okhudzana ndi kamwedwe kanu ka zokumwa zoledzeretsa mu m'miyezi 12 yapitayi. |  | Sono ndimufumbaninge mafumbo yakukhwaskana na kwamwelo kinu ka vyakumwa vyakulowereska mu miyezi 12 yajumpha iyi. |  | These questions are asked to respondents who accepted ever taking alcohol in their life. Read out note as written to participant. |
| *ALCFREQ* | During this past year how often did you have a drink containing alcohol? | 0  1  2  3  4  6 | Never  Monthly or less  2 to 4 times a month  2 to 3 times a week  4 or more times a week  Refused to answer | Mu m'miyezi 12 yapitayi ndi kowilikiza bwanji pamene mumamwa chakumwa cholezeletsa? | Palibe  Kamodzi pamwezi kapena kucheperapo  Kawiri kufikira kanayi pa mwezi  Kawiri kufikira katatu pa sabata  Kanayi kapena koposera apo pa sabata  Refused to answer | Mu miyezi 12 yajumpha iyi, nkhanandi wuli ako mukamwanga chakumwa chakuloweleska? | Palije  Kamoza pa mwezi panji kucheperapo  Kawiri kufikira kanayi pa mwezi  Kawiri panji katatu pa sabata  Kanayi panji kujumpha apo pa sabata  Refused to answer | Record as responded. |
| *DRINKTYP_1* | On a typical day when you are drinking, what is the main type of alcohol that you drink? | 1  2  3  4  5  6  7  96 | Local beer (chipumu, khangala, chikokeyani, masese)  Local fruit wine "Wayini"  Kachasu  Chibuku  Bottled beer or cider (e.g. Carlsberg, Castel, Savannah)  Industrial/imported spirit (rum, whisky, gin, vodka etc)  Imported wine  Refused to answer | Pa tsiku pamene mukumwa mowa, ndimtundu wanji wa mowa womwe mumakonda kumwa? | Local beer (chipumu, khangala, chikokeyani, masese)  Local fruit wine "Wayini"  Kachasu  Chibuku  Bottled beer or cider (e.g. Carlsberg, Castel, Savannah)  Industrial/imported spirit (rum, whisky, gin, vodka etc)  Imported wine  Refused to answer | Pa zuba ilo mukumwa moba, kasi nimoba wamutundu uli uwo mukutemwa kumwa? | Local beer (chipumu, khangala, chikokeyani, masese)  Local fruit wine "Wayini"  Kachasu  Chibuku  Bottled beer or cider (e.g. Carlsberg, Castel, Savannah)  Industrial/imported spirit (rum, whisky, gin, vodka etc)  Imported wine  Refused to answer | Record as responded. If a participants mentions more than one drink, inquire which one of these is the does the participant drink on a typical day. |
| *DRINKTYPICDAY_1* | How much of this do you drink on a typical day? [REFER TO STANDARD DRINK TABLE] |  |  | Kodi mumamwa mowa wochuluka bwanji watundu umenewu pa tsiku? [REFER TO STANDARD DRINK TABLE] |  | Kasi mukumwa moba unandi uli wamutundu uwu pa zuba? [REFER TO STANDARD DRINK TABLE] |  | Refer to standard drink table to convert to intake to a standard drink. Use “GM ASSIST/AUDIT FORM USER GUIDE” on how to use standard drink table. |
| *ALCOTH1_2* | In addition to ${drinktyp_1_str}, do you also drink another type of alcohol on the same typical day? | 1  0  6 | Yes  No  Refused to answer | Moonjezerapo ${drinktyp_1_str}, mumamwanso mtundu wina wa mowa pa tsiku lomweri? | Eya  Ayi  Refused to answer | Mwakusazgilapo ${drinktyp_1_str}, mkumwaso mtundu unyakhe wamoba pa zuba leneliri? | Enya  Yayi  Refused to answer | Ask if in addition to the typical drink, participant takes another type of drink. |
| *DRINKTYP_2* | What type of alcohol is it? | 1  2  3  4  5  6  7  96 | Local beer (chipumu, khangala, chikokeyani, masese)  Local fruit wine "Wayini"  Kachasu  Chibuku  Bottled beer or cider (e.g. Carlsberg, Castel, Savannah)  Industrial/imported spirit (rum, whisky, gin, vodka etc)  Imported wine  Refused to answer | Kodi ndi mowa wanji? | Local beer (chipumu, khangala, chikokeyani, masese)  Local fruit wine "Wayini"  Kachasu  Chibuku  Bottled beer or cider (e.g. Carlsberg, Castel, Savannah)  Industrial/imported spirit (rum, whisky, gin, vodka etc)  Imported wine  Refused to answer | Kasi ni moba wuli? | Local beer (chipumu, khangala, chikokeyani, masese)  Local fruit wine "Wayini"  Kachasu  Chibuku  Bottled beer or cider (e.g. Carlsberg, Castel, Savannah)  Industrial/imported spirit (rum, whisky, gin, vodka etc)  Imported wine  Refused to answer | Record as responded. |
| *DRINKTYPICDAY_2* | How much of this do you drink on the same typical day? [REFER TO STANDARD DRINK TABLE] |  |  | Kodi mumamwa mowa wochuluka bwanji watundu umenewu pa tsiku lomweri? [REFER TO STANDARD DRINK TABLE] |  | Kasi mukumwa moba unandi uli wamutundu uwu pa zuba leneliri? [REFER TO STANDARD DRINK TABLE] |  | Refer to standard drink table to convert to intake to a standard drink. Use “GM ASSIST/AUDIT FORM USER GUIDE” on how to use standard drink table. |
| *ALCOTH2_3* | In addition to ${drinktyp_1_str} and ${drinktyp_2_str}, do you also drink another type of alcohol on the same typical day? | 1  0  6 | Yes  No  Refused to answer | Moonjera pa ${drinktyp_1_str} ndi ${drinktyp_2_str}, mumamwanso mtundu wina wa mowa pa tsiku lomweri? | Eya  Ayi  Refused to answer | Mwakusazgilapo pa ${drinktyp_1_str} na ${drinktyp_2_str}, mkumwaso mtundu unyakhe wamoba pa zuba leneliri? | Enya  Yayi  Refused to answer | Ask if in addition to the typical drink, participant takes another type of drink. |
| *DRINKTYP_3* | What type of alcohol is it? | 1  2  3  4  5  6  7  96 | Local beer (chipumu, khangala, chikokeyani, masese)  Local fruit wine "Wayini"  Kachasu  Chibuku  Bottled beer or cider (e.g. Carlsberg, Castel, Savannah)  Industrial/imported spirit (rum, whisky, gin, vodka etc)  Imported wine  Refused to answer | Kodi ndi mowa wanji? | Local beer (chipumu, khangala, chikokeyani, masese)  Local fruit wine "Wayini"  Kachasu  Chibuku  Bottled beer or cider (e.g. Carlsberg, Castel, Savannah)  Industrial/imported spirit (rum, whisky, gin, vodka etc)  Imported wine  Refused to answer | Kasi ni moba wuli? | Local beer (chipumu, khangala, chikokeyani, masese)  Local fruit wine "Wayini"  Kachasu  Chibuku  Bottled beer or cider (e.g. Carlsberg, Castel, Savannah)  Industrial/imported spirit (rum, whisky, gin, vodka etc)  Imported wine  Refused to answer | Record as responded. |
| *DRINKTYPICDAY_3* | How much of this do you drink on the same typical day? [REFER TO STANDARD DRINK TABLE] |  |  | Kodi mumamwa mowa wochuluka bwanji watundu umenewu pa tsiku lomweri? [REFER TO STANDARD DRINK TABLE] |  | Kasi mukumwa moba unandi uli wamutundu uwu pa zuba leneliri? [REFER TO STANDARD DRINK TABLE] |  | Refer to standard drink table to convert to intake to a standard drink. Use “GM ASSIST/AUDIT FORM USER GUIDE” on how to use standard drink table. |
| *SUMMARY_NOTE* | Total number of "standard drinks" on a typical day:  ${stddrinktotal}   [If incorrect, go back and change previous answers.] |  |  | Total number of "standard drinks" on a typical day  ${stddrinktotal}   [If incorrect, go back and change previous answers] |  | Total number of "standard drinks" on a typical day  ${stddrinktotal}  [If incorrect, go back and change previous answers.] |  | The tablet will calculate total number of standard drinks based on intake. |
| *ALC6FREQ1* | In the last year, have there been some occasions when you have had at a lot of alcohol. I mean at least 5 bottles of Carlsberg, OR a 200ml bottle of kachasu/sprit OR 2 cartons of chibuku OR 2.5 bottles of “wayini” OR almost 1 bottle of imported wine OR a large number of drinks of different kinds? [NOTE: IF PERSON DOESN’T UNDERSTAND THEN REFER SPECIFICALLY TO THE TYPE(S) OF DRINK THAT THE PERSON SAID THAT THEY DRINK TYPICALLY] | 1  0  6 | Yes  No  Refused to answer | Mu miyezi khumi ndi iwiri yapitayi, panali nthawi zina pomwe mukamwa zakumwa zolezeletsa zambiri panthawi imodzi? Ndikuthanthauza kumwa mabotolo 5 kapena kupotsera apa a Carlsberg, kapena botololo la 200 ml la kachasu/sipiriti, makatoni awiri a chibuku, kapena mabotolo awiri ndi hafu ''wayini'' kapena pafupifupi botolo limodzi la wayini wopangidwa kuja kapena zakumwa zambiri zolezeletsa zosiyanasiyana? [NOTE: IF PERSON DOESN’T UNDERSTAND THEN REFER SPECIFICALLY TO THE TYPE(S) OF DRINK THAT THE PERSON SAID THAT THEY DRINK TYPICALLY] | Eya  Ayi  Refused to answer | Mu miyezi 12 yajumpha iyi pakaba nyengo izo mukamwa vyakumwa vyakuloweleska vinandi panyengo imoza? Nkhung'anamula kumwa mabotolo 5 ya Carlsberg panji kujumpha, panyakhe botolo la 200ml la kachasu/masipiriti, panji makatoni yawiri ya chiBuku, panji mabotolo ya wiri na hafu ya ''wayini'' panji pafupifupi botolo limoza la wayini wakupangika kuwalo panji kumwa vyakumwa vyakuloweleska vyakupAmbanapambana vinandi? [NOTE: IF PERSON DOESN’T UNDERSTAND THEN REFER SPECIFICALLY TO THE TYPE(S) OF DRINK THAT THE PERSON SAID THAT THEY DRINK TYPICALLY] | Enya  Yayi  Refused to answer | Read as written. Refer to their typical drink if they do not understand question. |
| *ALC6FREQ2* | How often do you do this? | 1  2  3  4  6 | Less than monthly (occasionally)  Monthly (1-3 times per month)  Weekly (1-3 times per week)  Daily or almost daily (4 or more times per week)  Refused to answer | Izi mumapanga mowirikiza bwanji? | Kochepera mwezi ndi mwezi (mwapanthawi)  Mwezi ndi mwezi (kamodzi fukikira katatu pa mwezi)  Sabata ndi sabata(kamozi kufikira katatu pa sabata)  Tsiku ndi tsiku kapena pafupifupi tsiku lili lonse (kanai kapena kupotsera apo pa sabata)  Refused to answer | Ivi mukuchitanga mwapafupipafupi uli? | Kuchepera mwezi na mwezi (panyengo)  Mwezi na mwezi (kamoza kufikira katatu pa mwezi)  Sabata na sabata (kamoza kufikira katatu pa sabata)  Zuwa na zuwa panji pafupifupi zuwa lililose (kanayi panji kujumphizga apa pa sabata)  Refused to answer | Questions below are asked to participants who agreed to taking alcohol in excess. Record as responded. |
| *ALCNOSTOP* | How often during the last year have you found that you were not able to stop drinking once you had started? | 0  1  2  3  4  6 | Never  Less than monthly (occasionally)  Monthly (1-3 times per month)  Weekly (1-3 times per week)  Daily or almost daily (4 or more times per week)  Refused to answer | Ndimowirikiza bwanji mu m'miyezi 12 yapitayi pamene mumapezeka kuti mukangoyamba kumwa zakumwa zoledzeretsa simumatha kusiya? | Palibe  Kochepera mwezi ndi mwezi (mwapanthawi)  Mwezi ndi mwezi (kamodzi fukikira katatu pa mwezi)  Sabata ndi sabata(kamozi kufikira katatu pa sabata)  Tsiku ndi tsiku kapena pafupifupi tsiku lili lonse (kanai kapena kupotsera apo pa sabata)  Refused to answer | Mu miyezi 12 yajumpha iyi, nkhanandi wuli apomukasanga kuti pala mwayamba kumwa mukatondekanga kulekelezga? | Palije  Kuchepera mwezi na mwezi (panyengo)  Mwezi na mwezi (kamoza kufikira katatu pa mwezi)  Sabata na sabata (kamoza kufikira katatu pa sabata)  Zuwa na zuwa panji pafupifupi zuwa lililose (kanayi panji kujumphizga apa pa sabata)  Refused to answer | Record as responded. |
| *ALCFAILEXP* | How often during the last year have you failed to do what was normally expected from you because of drinking? | 0  1  2  3  4  6 | Never  Less than monthly (occasionally)  Monthly (1-3 times per month)  Weekly (1-3 times per week)  Daily or almost daily (4 or more times per week)  Refused to answer | Ndimowirikiza bwanji, mu m'miyezi 12 yapitayi pamene munalephera kuchita zimene mumayenera kuchita nthawi zonse chifukwa cha kumwa kwanu kwa mowa? | Palibe  Kochepera mwezi ndi mwezi (mwapanthawi)  Mwezi ndi mwezi (kamodzi fukikira katatu pa mwezi)  Sabata ndi sabata(kamozi kufikira katatu pa sabata)  Tsiku ndi tsiku kapena pafupifupi tsiku lili lonse (kanai kapena kupotsera apo pa sabata)  Refused to answer | Mu miyezi 12 yajumpha iyi, nkhanandi wuli apo mukatondekanga kupanga ivyo ŵanthu ŵakukhazga kufuma kwa imwe chifukwa cha kumwa kwinu kwa moba? | Palije  Kuchepera mwezi na mwezi (panyengo)  Mwezi na mwezi (kamoza kufikira katatu pa mwezi)  Sabata na sabata (kamoza kufikira katatu pa sabata)  Zuwa na zuwa panji pafupifupi zuwa lililose (kanayi panji kujumphizga apa pa sabata)  Refused to answer | Record as responded. |
| *ALCMORN* | How often during the last year have you needed a first drink in the morning to get yourself going after a heavy drinking session? | 0  1  2  3  4  6 | Never  Less than monthly (occasionally)  Monthly (1-3 times per month)  Weekly (1-3 times per week)  Daily or almost daily (4 or more times per week)  Refused to answer | Ndimowirikiza bwanji, mu m'miyezi 12 yapitayi pamene choyambirira m’mawa mumafuna kumwa chakumwa choledzeretsa kuti muthe kukwaniritsa kuchita zinthu chifukwa dzulo lake munamwa kwambiri? | Palibe  Kochepera mwezi ndi mwezi (mwapanthawi)  Mwezi ndi mwezi (kamodzi fukikira katatu pa mwezi)  Sabata ndi sabata(kamozi kufikira katatu pa sabata)  Tsiku ndi tsiku kapena pafupifupi tsiku lili lonse (kanai kapena kupotsera apo pa sabata)  Refused to answer | Mu miyezi 12 yajumpha iyi, nkhanandi wuli apo mukakhumbanga chakumwa chakuloweleska chakwamba namlenji kuti mukwaniliske kuchita vinthu chifukwa chakuti mayilo yakhe mukamwa chomene? | Palije  Kuchepera mwezi na mwezi (panyengo)  Mwezi na mwezi (kamoza kufikira katatu pa mwezi)  Sabata na sabata (kamoza kufikira katatu pa sabata)  Zuwa na zuwa panji pafupifupi zuwa lililose (kanayi panji kujumphizga apa pa sabata)  Refused to answer | Record as responded. |
| *ALCGUILT* | How often during the last year have you had a feeling of guilt or remorse after drinking? | 0  1  2  3  4  6 | Never  Less than monthly (occasionally)  Monthly (1-3 times per month)  Weekly (1-3 times per week)  Daily or almost daily (4 or more times per week)  Refused to answer | Ndimowirikiza bwanji, mu m'miyezi 12 yapitayi pamene munamva kutsutsika kapena kudzimvera chisoni pamapeto pa kumwa? | Palibe  Kochepera mwezi ndi mwezi (mwapanthawi)  Mwezi ndi mwezi (kamodzi fukikira katatu pa mwezi)  Sabata ndi sabata(kamozi kufikira katatu pa sabata)  Tsiku ndi tsiku kapena pafupifupi tsiku lili lonse (kanai kapena kupotsera apo pa sabata)  Refused to answer | Mu miyezi 12 yajumpha iyi, nkhanandi wuli apo mukaŵapo na kapulikiro kakuti mwananga panji kujiyeruzga pamanyuma pakumwa? | Palije  Kuchepera mwezi na mwezi (panyengo)  Mwezi na mwezi (kamoza kufikira katatu pa mwezi)  Sabata na sabata (kamoza kufikira katatu pa sabata)  Zuwa na zuwa panji pafupifupi zuwa lililose (kanayi panji kujumphizga apa pa sabata)  Refused to answer | Record as responded. |
| *ALCREMEM* | How often during the last year have you been unable to remember what happened the night before because you had been drinking? | 0  1  2  3  4  6 | Never  Less than monthly (occasionally)  Monthly (1-3 times per month)  Weekly (1-3 times per week)  Daily or almost daily (4 or more times per week)  Refused to answer | Ndimowirikiza bwanji, mu m'miyezi 12 yapitayi pamene simumatha kukumbukira zimene zinachitika usiku wathawo chifukwa munali mutamwa mowa? | Palibe  Kochepera mwezi ndi mwezi (mwapanthawi)  Mwezi ndi mwezi (kamodzi fukikira katatu pa mwezi)  Sabata ndi sabata(kamozi kufikira katatu pa sabata)  Tsiku ndi tsiku kapena pafupifupi tsiku lili lonse (kanai kapena kupotsera apo pa sabata)  Refused to answer | Mu miyezi 12 yajumpha iyi, nkhanandi wuli apo mukatondeka kukumbuka ivyo vikachitika mu usiku wamala chifukwa mukaŵa kuti mukamwa moba? | Palije  Kuchepera mwezi na mwezi (panyengo)  Mwezi na mwezi (kamoza kufikira katatu pa mwezi)  Sabata na sabata (kamoza kufikira katatu pa sabata)  Zuwa na zuwa panji pafupifupi zuwa lililose (kanayi panji kujumphizga apa pa sabata)  Refused to answer | Record as responded. |
| *ALCPASTPRESNTNOTE* | Now I am going to ask you some more questions related to your use of alcoholic beverages in the PAST or PRESENT. If you no longer use alcohol at all, please think back to your past use of alcohol and answer the next questions based on that. |  |  | Tsopano ndikufunsani mafunso ena wokhudzana ndi kumwa mowa kwanu M’MBUYOMU kapena PAKALI PANO. Ngati pakali pano simumamwanso wowa, ganizirani m’mbuyomu pamene mumamwa mowa ndi kuyankha mafunso wotsatira potengera zimenezo. |  | Sono ndimfumbaninge mafumbo yakukhwaskana naumo mukamwelanga moba mu nyengo za KUMASINDA panji KWASONO. Usange muli kulekelathu, chonde ghanaghanani umo mukamwelanga moba kale na kuzgola mafumbo yakulondezgapo kugwiriska ntchito vyenevivyo. |  | Read note exactly as written. Remind participant that this includes a time when they used to take alcohol, if they no longer take alcohol now. |
| *ALCINJ* | Have you or someone else been injured as a result of your drinking? | 0  2  4  6 | No  Yes, but not in the last year  Yes, during the last year  Refused to answer | Kodi inu kapena wina wake anavulalapo chifukwa cha kumwa mowa kwanu? | Ayi  Inde, koma osati mu chaka chathachi  Inde, mu chaka chathachi  Refused to answer | Kasi imwe panji munthu munyakhe walikupwetekekapo chifukwa cha kumwa moba kwinu? | Yayi  Enya, kweni chaka chamala yayi  Enya, mu chaka chamala ichi  Refused to answer | Record as responded. |
| *ALCCONCERN* | Has a relative or friend or a doctor or another health worker been concerned about your drinking or suggested you cut down? | 0  2  4  6 | No  Yes, but not in the last year  Yes, during the last year  Refused to answer | Kodi wachibale kapena mzanu kapena dokotala kapena wina aliyense wogwira ntchito ya zaumoyo anakhudzidwapo ndi kumwa kwanu kwa mowa kapena ananenapo kuti muchepetse? | Ayi  Inde, koma osati mu chaka chathachi  Inde, mu chaka chathachi  Refused to answer | Kasi mubali panji mubwezi panji dokotala panji wakugwira ntchito za vyaumoyo munyakhe wali kukhwaskikapo na kumwa kwinu kwa moba panji kusachizga kuti muchepeske? | Yayi  Enya, kweni chaka chamala yayi  Enya, mu chaka chamala ichi  Refused to answer | Record as responded. |
| ***ASSISTQ2*** | **ASSIST Q 2** |  |  | **ASSIST Q 2** |  | **ASSIST Q 2** |  | These questions are asked to respondents who accepted ever taking any substance in their life. Read out note as written to participant. Remind participant that you are asking questions of substance use in ­the past three months only |
| *ASSISTQ2_INTRO_M* | I am now going to ask you about your use of substances in the past three months only. |  |  | Tsopano ndikufunsani mafunso okhuzana ndi mankhwala ozunguza bongo omwe mwakhala mukugwiritsa ntchito miyezi itatu yathayi. |  | Sono ndimufumbaninge mafumbo yakukhwaskana na mankhwala yakunanga wongo ayo mwagwiriska ntchito mu miyezi yitatu yajumpha. |  |  |
| *ALC3MON* | This is another question about alcohol. In the past three months, how often have you used Alcohol? | 0  1  2  3  4  6 | Never  Once or Twice  Monthly (1-3 TIMES PER MONTH)  Weekly (1-3 TIMES PER WEEK)  Daily or almost daily (4 OR MORE TIMES A WEEK)  Refused to answer | Ili ndifunso lina lokhuza mowa. Miyezi itatu yathayi, ndimowirikiza bwanji pamene munagwiritsa ntchito mowa? | Palibe  Kamodzi kapena kawiri  Mwezi ndi mwezi (kamodzi fukikira katatu pa mwezi)  Sabata ndi sabata (kamozi kufikira katatu pa sabata)  Tsiku ndi tsiku kapena pafupifupi tsiku lili lonse (kanai kapena kupotsera apo pa sabata)  Refused to answer | Ili nifumbo linyakhe lokhwaskana na mowa.Mu miyezi yitatu yajumpha, nkhanandi wuli apo mukagwiliska ntchito moba? | Palije  Kamoza panji kawiri  Mwezi na mwezi (kamoza kufikira katatu pa mwezi)  Sabata na sabata (kamoza kufikira katatu pa sabata)  Zuwa na zuwa panji pafupifupi zuwa lililose (kanayi panji kujumphizga apa pa sabata)  Refused to answer | Record as responded. Read out options to participants. |
| *TOBAC3MON* | In the past three months, how often have you used Tobacco products (cigarettes, chewing tobacco, kuber, cigars, labu etc.)? | 0  1  2  3  4  6 | Never  Once or Twice  Monthly (1-3 TIMES PER MONTH)  Weekly (1-3 TIMES PER WEEK)  Daily or almost daily (4 OR MORE TIMES A WEEK)  Refused to answer | Mu miyezi itatu yathayi, ndimowirikiza bwanji pamene munagwiritsa ntchito zopangidwa kuchoka ku fodya (monga ndundu, fodya wotafuna/fodya wamphuno, kyuba, chikololo (paipi), chingambwe ndi zina zotero)? | Palibe  Kamodzi kapena kawiri  Mwezi ndi mwezi (kamodzi fukikira katatu pa mwezi)  Sabata ndi sabata (kamozi kufikira katatu pa sabata)  Tsiku ndi tsiku kapena pafupifupi tsiku lili lonse (kanai kapena kupotsera apo pa sabata)  Refused to answer | Mu miyezi yitatu yajumpha iyi,nkhanandi wuli apo mukagwiliska ntchito vyakupangika kufuma ku hona (nge ndudu, hona wakusumba, hona wamphuno, ngilimbo, kyuba, chikololo, chingambwe na vinyake)? | Palije  Kamoza panji kawiri  Mwezi na mwezi (kamoza kufikira katatu pa mwezi)  Sabata na sabata (kamoza kufikira katatu pa sabata)  Zuwa na zuwa panji pafupifupi zuwa lililose (kanayi panji kujumphizga apa pa sabata)  Refused to answer | Record as responded. Read out options to participants. |
| *CANNABIS3MON* | In the past three months, how often have you used Cannabis (marijuana, pot, grass, hash, etc.) | 0  1  2  3  4  6 | Never  Once or Twice  Monthly (1-3 TIMES PER MONTH)  Weekly (1-3 TIMES PER WEEK)  Daily or almost daily (4 OR MORE TIMES A WEEK)  Refused to answer | Mu miyezi itatu yathayi, ndimowirikiza bwanji pamene munagwiritsa ntchito chamba ndi zinthu zomwe zimapangidwa zochokera ku chamba? | Palibe  Kamodzi kapena kawiri  Mwezi ndi mwezi (kamodzi fukikira katatu pa mwezi)  Sabata ndi sabata (kamozi kufikira katatu pa sabata)  Tsiku ndi tsiku kapena pafupifupi tsiku lili lonse (kanai kapena kupotsera apo pa sabata)  Refused to answer | Mu miyezi yitatu yajumpha, nkhanandi wuli apo mukagwiliska ntchito chamba na vyakufuma ku chamba? | Palije  Kamoza panji kawiri  Mwezi na mwezi (kamoza kufikira katatu pa mwezi)  Sabata na sabata (kamoza kufikira katatu pa sabata)  Zuwa na zuwa panji pafupifupi zuwa lililose (kanayi panji kujumphizga apa pa sabata)  Refused to answer | Record as responded. Read out options to participants. |
| *COCAINE3MON* | In the past three months, how often have you used Cocaine (coke, crack, etc.) | 0  1  2  3  4  6 | Never  Once or Twice  Monthly (1-3 TIMES PER MONTH)  Weekly (1-3 TIMES PER WEEK)  Daily or almost daily (4 OR MORE TIMES A WEEK)  Refused to answer | Mu miyezi itatu yathayi, ndimowirikiza bwanji pamene munagwiritsa ntchito kokeni ? | Palibe  Kamodzi kapena kawiri  Mwezi ndi mwezi (kamodzi fukikira katatu pa mwezi)  Sabata ndi sabata (kamozi kufikira katatu pa sabata)  Tsiku ndi tsiku kapena pafupifupi tsiku lili lonse (kanai kapena kupotsera apo pa sabata)  Refused to answer | Mu miyezi yitatu yajumpha, nkhanandi wuli apo mukagwiliska ntchito kokeni? | Palije  Kamoza panji kawiri  Mwezi na mwezi (kamoza kufikira katatu pa mwezi)  Sabata na sabata (kamoza kufikira katatu pa sabata)  Zuwa na zuwa panji pafupifupi zuwa lililose (kanayi panji kujumphizga apa pa sabata)  Refused to answer | Record as responded. Read out options to participants. |
| *AMPHET3MON* | In the past three months, how often have you used Amphetamine type stimulants (e.g. speed, ecstacy, tik)? | 0  1  2  3  4  6 | Never  Once or Twice  Monthly (1-3 TIMES PER MONTH)  Weekly (1-3 TIMES PER WEEK)  Daily or almost daily (4 OR MORE TIMES A WEEK)  Refused to answer | Mu miyezi itatu yathayi, ndimowirikiza bwanji pamene munagwiritsa ntchito mankhwala ochangamutsa (e.g. speed, ecstacy, tik)? | Palibe  Kamodzi kapena kawiri  Mwezi ndi mwezi (kamodzi fukikira katatu pa mwezi)  Sabata ndi sabata (kamozi kufikira katatu pa sabata)  Tsiku ndi tsiku kapena pafupifupi tsiku lili lonse (kanai kapena kupotsera apo pa sabata)  Refused to answer | Mu miyezi yitatu yajumpha, nkhanandi wuli apo mukagwiliska ntchito mulikugwiliskapo ntchito mankhwala yakuchentchemuska munthu (e.g. speed, ecstacy, tik)? | Palije  Kamoza panji kawiri  Mwezi na mwezi (kamoza kufikira katatu pa mwezi)  Sabata na sabata (kamoza kufikira katatu pa sabata)  Zuwa na zuwa panji pafupifupi zuwa lililose (kanayi panji kujumphizga apa pa sabata)  Refused to answer | Record as responded. Read out options to participants. |
| *INHALANT3MON* | In the past three months, how often have you used Inhalants (glue, petrol etc.)? | 0  1  2  3  4  6 | Never  Once or Twice  Monthly (1-3 TIMES PER MONTH)  Weekly (1-3 TIMES PER WEEK)  Daily or almost daily (4 OR MORE TIMES A WEEK)  Refused to answer | Mu miyezi itatu yathayi, ndimowirikiza bwanji pamene munagwiritsa ntchito zofwenthera (monga glue, petrol ndi zina zotero)? | Palibe  Kamodzi kapena kawiri  Mwezi ndi mwezi (kamodzi fukikira katatu pa mwezi)  Sabata ndi sabata (kamozi kufikira katatu pa sabata)  Tsiku ndi tsiku kapena pafupifupi tsiku lili lonse (kanai kapena kupotsera apo pa sabata)  Refused to answer | Mu miyezi yitatu yajumpha, nkhanandi wuli apo mukagwiliska ntchito vyakukhweba (nge glue, petrol na vinyakhe)? | Palije  Kamoza panji kawiri  Mwezi na mwezi (kamoza kufikira katatu pa mwezi)  Sabata na sabata (kamoza kufikira katatu pa sabata)  Zuwa na zuwa panji pafupifupi zuwa lililose (kanayi panji kujumphizga apa pa sabata)  Refused to answer | Record as responded. Read out options to participants. |
| *SEDATIVE3MON* | In the past three months, how often have you used Sedatives or Sleeping Pills (Valium, Diazepam etc.)? | 0  1  2  3  4  6 | Never  Once or Twice  Monthly (1-3 TIMES PER MONTH)  Weekly (1-3 TIMES PER WEEK)  Daily or almost daily (4 OR MORE TIMES A WEEK)  Refused to answer | Mu miyezi itatu yathayi, ndimowirikiza bwanji pamene munagwiritsa ntchito mankhwala ogonetsa kuposera mulingo womwe anakulemberani adotolo (monga Valium kapena kuti Diazepam)? | Palibe  Kamodzi kapena kawiri  Mwezi ndi mwezi (kamodzi fukikira katatu pa mwezi)  Sabata ndi sabata (kamozi kufikira katatu pa sabata)  Tsiku ndi tsiku kapena pafupifupi tsiku lili lonse (kanai kapena kupotsera apo pa sabata)  Refused to answer | Mu miyezi yitatu yajumpha, nkhanandi wuli apo mukagwiliska ntchito mankhwala yakwiziska tulo mwakujumphizga mulingo uwo wakamulembelani badokotala (nge Valium panji Diazepam)? | Palije  Kamoza panji kawiri  Mwezi na mwezi (kamoza kufikira katatu pa mwezi)  Sabata na sabata (kamoza kufikira katatu pa sabata)  Zuwa na zuwa panji pafupifupi zuwa lililose (kanayi panji kujumphizga apa pa sabata)  Refused to answer | Record as responded. Read out options to participants. |
| *HALLUC3MON* | In the past three months, how often have you used Hallucinogens (magic mushrooms etc.)? | 0  1  2  3  4  6 | Never  Once or Twice  Monthly (1-3 TIMES PER MONTH)  Weekly (1-3 TIMES PER WEEK)  Daily or almost daily (4 OR MORE TIMES A WEEK)  Refused to answer | Mu miyezi itatu yathayi, ndimowirikiza bwanji pamene munagwiritsa ntchito mankhwala ena opangisa kuona zinthu zomwe anthu ena sakuziwona ndi maso awo (bowa wa poyizoni ndi zina zotero)? | Palibe  Kamodzi kapena kawiri  Mwezi ndi mwezi (kamodzi fukikira katatu pa mwezi)  Sabata ndi sabata (kamozi kufikira katatu pa sabata)  Tsiku ndi tsiku kapena pafupifupi tsiku lili lonse (kanai kapena kupotsera apo pa sabata)  Refused to answer | Mu miyezi yitatu yajumpha, nkhanandi wuli apo mukagwiliska ntchito mankhwala yanyake ayo yakupangiska kuti muwone vinthu vyakuti banthu banyakhe bakuviwona yayi na maso (vikanje na vinyakhe)? | Palije  Kamoza panji kawiri  Mwezi na mwezi (kamoza kufikira katatu pa mwezi)  Sabata na sabata (kamoza kufikira katatu pa sabata)  Zuwa na zuwa panji pafupifupi zuwa lililose (kanayi panji kujumphizga apa pa sabata)  Refused to answer | Record as responded. Read out options to participants. |
| *OPIOID3MON* | In the past three months, how often have you used illegal opioids (e.g. heroin) or opioids (e.g. morphine, pethidine, methadone, codeine, cough syrup with codeine etc) for reasons other than prescription, or taken them more frequently or at higher doses than prescribed by a doctor? | 0  1  2  3  4  6 | Never  Once or Twice  Monthly (1-3 TIMES PER MONTH)  Weekly (1-3 TIMES PER WEEK)  Daily or almost daily (4 OR MORE TIMES A WEEK)  Refused to answer | Mu miyezi itatu yathayi, ndimowirikiza bwanji pamene munagwiritsa ntchito mankhwala olesedwa a heroin, kapena makhwala amphamvu oletsa ululu waukulu monga morphine, perthidine, methadone, codeine, cough syrup wophatikizako codeine ndi zina zotero pa zifukwa zina kupatula zomwe anakulemberani adotolo kapena kumwa mowirikiza kapenanso kuposera mulingo omwe munapatsidwa? | Palibe  Kamodzi kapena kawiri  Mwezi ndi mwezi (kamodzi fukikira katatu pa mwezi)  Sabata ndi sabata (kamozi kufikira katatu pa sabata)  Tsiku ndi tsiku kapena pafupifupi tsiku lili lonse (kanai kapena kupotsera apo pa sabata)  Refused to answer | Mu miyezi yitatu yajumpha, nkhanandi wuli apo mukagwiliska ntchito mankhwala yakukanizgika ya heroin panyakhe makhwala yankhongono yakulekeska wurwirwi wukulu nge morphine, pethidine, methadone, codeine, cough syrup wakuskazikako codeine na vinyakhe pavifukwa vinyakhe vyakuperekeka na badokotala yayi, panji para mukamwa mankhwala aya mwakujumphizga ndondomeko? | Palije  Kamoza panji kawiri  Mwezi na mwezi (kamoza kufikira katatu pa mwezi)  Sabata na sabata (kamoza kufikira katatu pa sabata)  Zuwa na zuwa panji pafupifupi zuwa lililose (kanayi panji kujumphizga apa pa sabata)  Refused to answer | Record as responded. Read out options to participants. |
| *OTH3MON* | In the past three months, how often have you used any other illegal drug or used drugs for reasons other than prescription, or taken them more frequently or at higher doses than prescribed by a doctor? | 0  1  2  3  4  6 | Never  Once or Twice  Monthly (1-3 TIMES PER MONTH)  Weekly (1-3 TIMES PER WEEK)  Daily or almost daily (4 OR MORE TIMES A WEEK)  Refused to answer | Mu miyezi itatu yathayi, ndimowirikiza bwanji pamene munagwiritsa ntchito mankhwala ena aliwonse oletsedwa kapena kugwiritsa ntchito makhwala pa zifukwa zina kupatula zomwe anakulemberani adotolo kapena kumwa mowirikiza kapenanso kuposera mulingo omwe munapatsidwa? | Palibe  Kamodzi kapena kawiri  Mwezi ndi mwezi (kamodzi fukikira katatu pa mwezi)  Sabata ndi sabata (kamozi kufikira katatu pa sabata)  Tsiku ndi tsiku kapena pafupifupi tsiku lili lonse (kanai kapena kupotsera apo pa sabata)  Refused to answer | Mu miyezi yitatu yajumpha, nkhanandi wuli apo mulikugwiliska ntchito mankhwala yanyake yaliyose yakukanizgika panji kugwiliska mankhwala pavifukwa vinyakhe vyakuperekeka na badokotala yayi, panji para mukamwa mankhwala aya mwakujumphizga ndondomeko? | Palije  Kamoza panji kawiri  Mwezi na mwezi (kamoza kufikira katatu pa mwezi)  Sabata na sabata (kamoza kufikira katatu pa sabata)  Zuwa na zuwa panji pafupifupi zuwa lililose (kanayi panji kujumphizga apa pa sabata)  Refused to answer | Record as responded. Read out options to participants. |
| ***ASSISTQ1*** | **ASSIST Q 1** |  |  | **ASSIST Q 1** |  | **ASSIST Q 1** |  |  |
| *ASSISTQ1_INTRO* | I am now going to ask you some questions about alcohol, tobacco products and other drugs. Some of these substances may be prohibited by law, but please be assured that all information we will collect will be kept private and confidential. |  |  | Ndikufunsani mafunso okhudzana ndi mowa, fodya, ndi mankhwala ena.    Ena mwa mankhwala antha kukhala oletsedwa, chonde dziwani kuti uthenga omwe utengedwe ukasungida motetezedwa ndi mwa chinsinsi. |  | Sono ndimufumbaninge mafumbo yakukhwaskana na mowa, hona, na mankhwala yanyakhe.   Yanyakhe mwa mankhwala aya yangaba yakukanizgika, chonde manyani kuti wanduzi wose uwo utolekenge wamusungika mwa kuvikilirika na mwa chisisi. |  | Read note exactly as written. Emphasize to participants about confidentiality of the information collected. If participants agree to taking any substance, confirm if they understand what the substance is. |
| *ALCEVER* | In your life, have you ever used alcohol? Examples of alcoholic beverages are bottled beer (Carlsberg), spirits, chibuku and locally brewed alcohol. | 1  0  8  6 | Yes  No  Don't Know  Refused to answer | Mu moyo wanu, munamwako mowa? Mu gulu la zokumwa zoledzeletsa (mowa), muli mowa wa m’botolo (monga Carlsberg, castel) mowa wa masacheti, maspiriti, wayini, Chibuku, kachasu, mowa wina wophikidwa mmudzi ndi mowa ina yosiyanasiyana. | Eya  Ayi  Sindikudziwa  Refused to answer | Mu moyo winu, muli kumwapo moba? Mu gulu la vyakumwa vyakulowereska (moba), muli moba wa ma botolo (nge Carlsberg, Castel), moba wa masacheti, masipiriti, wayini, Chibuku, kachasu, moba wakuphikika mchikaya, na moba inyakhe yakupambanapambana. | Enya  Yayi  Nkhumanya yayi  Refused to answer | Record as responded. |
| *TOBACEVER* | In your life, have you ever used tobacco products (e.g. cigarettes, chewing tobacco, kuber, cigars, labu etc.)? | 1  0  8  6 | Yes  No  Don't Know  Refused to answer | Mu moyo wanu munagwiritsapo ntchito zopangidwa kuchoka ku fodya (monga ndundu, fodya wotafuna/fodya wamphuno, kyuba, chikololo (paipi), chingambwe ndi zina zotero)? | Eya  Ayi  Sindikudziwa  Refused to answer | Pamoyo winu, kasi mulikugwiliskapo ntchito vyakupangika kufuma ku hona (nge ndudu, hona wakusumba, hona wamphuno, ngilimbo, kyuba, chikololo, chingambwe na vinyake)? | Enya  Yayi  Nkhumanya yayi  Refused to answer | Record as responded. |
| *CANNABISEVER* | In your life, have you ever used cannabis (marijuana, pot, grass, hash etc.)? | 1  0  8  6 | Yes  No  Don't Know  Refused to answer | Mu moyo wanu, munagwiritsapo ntchito chamba ndi zinthu zomwe zimapangidwa kuchokera ku chamba? | Eya  Ayi  Sindikudziwa  Refused to answer | Pamoyo winu, kasi mulikugwiliskapo ntchito chamba na vyakupangika kufuma ku chamba? | Enya  Yayi  Nkhumanya yayi  Refused to answer | Record as responded. |
| *COCAINEEVER* | In your life, have you ever used cocaine (coke, crack, etc.)? | 1  0  8  6 | Yes  No  Don't Know  Refused to answer | Mu moyo wanu, munagwiritsapo ntchito kokeni? | Eya  Ayi  Sindikudziwa  Refused to answer | Pamoyo winu, kasi mulikugwiliskapo ntchito kokeni? | Enya  Yayi  Nkhumanya yayi  Refused to answer | Record as responded. |
| *AMPHETEVER* | In your life, have you ever used amphetamine type stimulants? Names of these drugs include speed, ecstasy and tik. | 1  0  8  6 | Yes  No  Don't Know  Refused to answer | Mu moyo wanu, munagwiritsapo ntchito mankhwala ochangamutsa? Maina a makhwala awa ndimonga speed, ecstasy and tik. | Eya  Ayi  Sindikudziwa  Refused to answer | Pamoyo winu, kasi mulikugwiliskapo ntchito mankhwala yakuchentchemuska munthu? Mazina ya makhwala aya ningati speed, ecstasy and tik. | Enya  Yayi  Nkhumanya yayi  Refused to answer | Record as responded. |
| *INHALANTEVER* | In your life, have you ever used inhalants (glue, petrol, etc.)? | 1  0  8  6 | Yes  No  Don't Know  Refused to answer | Mu moyo wanu, munagwiritsapo ntchito zofwenthera (monga glue, petrol ndi zina zotero)? | Eya  Ayi  Sindikudziwa  Refused to answer | Pamoyo winu, kasi mulikugwiliskapo ntchito vyakukhweba (nge glue, petrol na vinyakhe)? | Enya  Yayi  Nkhumanya yayi  Refused to answer | Record as responded. |
| *SEDATIVEEVER* | In your life, have you ever used sedatives or sleeping pills more than you were prescribed by a doctor (e.g Valium or Diazepam)? | 1  0  8  6 | Yes  No  Don't Know  Refused to answer | Mu moyo wanu, munagwiritsapo ntchito mankhwala ogonetsa kuposera mulingo womwe anakulemberani adotolo (monga Valium kapena kuti Diazepam)? | Eya  Ayi  Sindikudziwa  Refused to answer | Pamoyo winu, kasi mulikugwiliskapo ntchito mankhwala yakwiziska tulo mwakujumphizga mulingo uwo wakamulembelani badokotala (nge Valium panji Diazepam)? | Enya  Yayi  Nkhumanya yayi  Refused to answer | Record as responded. |
| *HALLUCEVER* | In your life, have you ever used hallucinogens (magic mushrooms etc.)? | 1  0  8  6 | Yes  No  Don't Know  Refused to answer | Mu moyo wanu, munagwiritsapo ntchito mankhwala ena opangisa kuona zinthu zomwe anthu ena sakuziwona ndi maso awo (bowa wa poyizoni ndi zina zotero)? | Eya  Ayi  Sindikudziwa  Refused to answer | Pamoyo winu, kasi mulikugwiliskapo ntchito mankhwala yanyake ayo yakuwoneska vinthu vyakuti banthu banyakhe bakuviwona yayi na maso (vikanje na vinyakhe)? | Enya  Yayi  Nkhumanya yayi  Refused to answer | Record as responded. |
| *OPIOIDEVER* | In your life, have you ever used illegal opiods (e.g. heroin) or other opiods (e.g. morphine, pethidine, methadone, codeine, cough syrup with codeine etc) for reasons other than prescription, or taken them more frequently or at higher doses than prescribed by a doctor? | 1  0  8  6 | Yes  No  Don't Know  Refused to answer | Mu moyo wanu, munagwiritsapo ntchito mankhwala oletsedwa a heroin, kapena mankhwala amphamvu oletsa ululu waukulu monga morphine, pethidine, methadone, codeine, cough syrup wophatikizako codeine ndi zina zotero pa zifukwa zina kupatula zomwe anakulemberani adotolo kapena kumwa mowirikiza kapenanso kuposera mulingo omwe munapatsidwa? | Eya  Ayi  Sindikudziwa  Refused to answer | Pamoyo winu, kasi mulikugwiliskapo ntchito mankhwala yakukanizgika ya heroin panyakhe mankhwala yankhongono yakulekeska wurwirwi wukulu nge morphine, pethidine, methadone, codeine, cough syrup wakuskazikako codeine na vinyakhe pavifukwa vinyakhe vyakuperekeka na badokotala yayi, panji para mukamwa mankhwala aya mwakujumphizga ndondomeko? | Enya  Yayi  Nkhumanya yayi  Refused to answer | Record as responded. |
| *OTHEVER* | In your life, have you ever used any other illegal drug or used drugs for reasons other than prescription, or taken them more frequently or at higher doses than prescribed by a doctor? | 1  0  8  6 | Yes  No  Don't Know  Refused to answer | Mu moyo wanu, munagwiritsapo ntchito mankhwala ena aliwonse oletsedwa kapena kugwiritsa ntchito mankhwala pa zifukwa zina kupatula zomwe anakulemberani adotolo kapena kumwa mowirikiza kapenanso kuposera mulingo omwe munapatsidwa? | Eya  Ayi  Sindikudziwa  Refused to answer | Pamoyo winu, mulikugwiliskapo ntchito mankhwala yanyake yaliyose yakukanizgika panji kugwiliska mankhwala pavifukwa vinyakhe vyakuperekeka na badokotala yayi, panji para mukamwa mankhwala aya mwakujumphizga ndondomeko? | Enya  Yayi  Nkhumanya yayi  Refused to answer | Record as responded. |
| *OTHEVERSPEC* | If so, what was it? |  |  | Ngati eya, mankhwala anji? |  | Para enya, mankhwala uli? |  | Specify any other drug used if yes to above. |
| ***AUDIT*** | **AUDIT** |  |  | **AUDIT** |  | **AUDIT** |  |  |
| *INTRO* | Now I am going to ask you some questions about your use of alcoholic beverages during this past 12 months |  |  | Pano ndikufunsani mafunso okhudzana ndi kamwedwe kanu ka zokumwa zoledzeretsa mu m'miyezi 12 yapitayi. |  | Sono ndimufumbaninge mafumbo yakukhwaskana na kwamwelo kinu ka vyakumwa vyakulowereska mu miyezi 12 yajumpha iyi. |  | These questions are asked to respondents who accepted ever taking alcohol in their life. Read out note as written to participant. |
| *ALCFREQ* | During this past year how often did you have a drink containing alcohol? | 0  1  2  3  4  6 | Never  Monthly or less  2 to 4 times a month  2 to 3 times a week  4 or more times a week  Refused to answer | Mu m'miyezi 12 yapitayi ndi kowilikiza bwanji pamene mumamwa chakumwa cholezeletsa? | Palibe  Kamodzi pamwezi kapena kucheperapo  Kawiri kufikira kanayi pa mwezi  Kawiri kufikira katatu pa sabata  Kanayi kapena koposera apo pa sabata  Refused to answer | Mu miyezi 12 yajumpha iyi, nkhanandi wuli ako mukamwanga chakumwa chakuloweleska? | Palije  Kamoza pa mwezi panji kucheperapo  Kawiri kufikira kanayi pa mwezi  Kawiri panji katatu pa sabata  Kanayi panji kujumpha apo pa sabata  Refused to answer | Record as responded. |
| *DRINKTYP_1* | On a typical day when you are drinking, what is the main type of alcohol that you drink? | 1  2  3  4  5  6  7  96 | Local beer (chipumu, khangala, chikokeyani, masese)  Local fruit wine "Wayini"  Kachasu  Chibuku  Bottled beer or cider (e.g. Carlsberg, Castel, Savannah)  Industrial/imported spirit (rum, whisky, gin, vodka etc)  Imported wine  Refused to answer | Pa tsiku pamene mukumwa mowa, ndimtundu wanji wa mowa womwe mumakonda kumwa? | Local beer (chipumu, khangala, chikokeyani, masese)  Local fruit wine "Wayini"  Kachasu  Chibuku  Bottled beer or cider (e.g. Carlsberg, Castel, Savannah)  Industrial/imported spirit (rum, whisky, gin, vodka etc)  Imported wine  Refused to answer | Pa zuba ilo mukumwa moba, kasi nimoba wamutundu uli uwo mukutemwa kumwa? | Local beer (chipumu, khangala, chikokeyani, masese)  Local fruit wine "Wayini"  Kachasu  Chibuku  Bottled beer or cider (e.g. Carlsberg, Castel, Savannah)  Industrial/imported spirit (rum, whisky, gin, vodka etc)  Imported wine  Refused to answer | Record as responded. If a participants mentions more than one drink, inquire which one of these is the does the participant drink on a typical day. |
| *DRINKTYPICDAY_1* | How much of this do you drink on a typical day? [REFER TO STANDARD DRINK TABLE] |  |  | Kodi mumamwa mowa wochuluka bwanji watundu umenewu pa tsiku? [REFER TO STANDARD DRINK TABLE] |  | Kasi mukumwa moba unandi uli wamutundu uwu pa zuba? [REFER TO STANDARD DRINK TABLE] |  | Refer to standard drink table to convert to intake to a standard drink. Use “GM ASSIST/AUDIT FORM USER GUIDE” on how to use standard drink table. |
| *ALCOTH1_2* | In addition to ${drinktyp_1_str}, do you also drink another type of alcohol on the same typical day? | 1  0  6 | Yes  No  Refused to answer | Moonjezerapo ${drinktyp_1_str}, mumamwanso mtundu wina wa mowa pa tsiku lomweri? | Eya  Ayi  Refused to answer | Mwakusazgilapo ${drinktyp_1_str}, mkumwaso mtundu unyakhe wamoba pa zuba leneliri? | Enya  Yayi  Refused to answer | Ask if in addition to the typical drink, participant takes another type of drink. |
| *DRINKTYP_2* | What type of alcohol is it? | 1  2  3  4  5  6  7  96 | Local beer (chipumu, khangala, chikokeyani, masese)  Local fruit wine "Wayini"  Kachasu  Chibuku  Bottled beer or cider (e.g. Carlsberg, Castel, Savannah)  Industrial/imported spirit (rum, whisky, gin, vodka etc)  Imported wine  Refused to answer | Kodi ndi mowa wanji? | Local beer (chipumu, khangala, chikokeyani, masese)  Local fruit wine "Wayini"  Kachasu  Chibuku  Bottled beer or cider (e.g. Carlsberg, Castel, Savannah)  Industrial/imported spirit (rum, whisky, gin, vodka etc)  Imported wine  Refused to answer | Kasi ni moba wuli? | Local beer (chipumu, khangala, chikokeyani, masese)  Local fruit wine "Wayini"  Kachasu  Chibuku  Bottled beer or cider (e.g. Carlsberg, Castel, Savannah)  Industrial/imported spirit (rum, whisky, gin, vodka etc)  Imported wine  Refused to answer | Record as responded.  Refer to standard drink table to convert to intake to a standard drink. Use “GM ASSIST/AUDIT FORM USER GUIDE” on how to use standard drink table. |
| *DRINKTYPICDAY_2* | How much of this do you drink on the same typical day? [REFER TO STANDARD DRINK TABLE] |  |  | Kodi mumamwa mowa wochuluka bwanji watundu umenewu pa tsiku lomweri? [REFER TO STANDARD DRINK TABLE] |  | Kasi mukumwa moba unandi uli wamutundu uwu pa zuba leneliri? [REFER TO STANDARD DRINK TABLE] |  |  |
| *ALCOTH2_3* | In addition to ${drinktyp_1_str} and ${drinktyp_2_str}, do you also drink another type of alcohol on the same typical day? | 1  0  6 | Yes  No  Refused to answer | Moonjera pa ${drinktyp_1_str} ndi ${drinktyp_2_str}, mumamwanso mtundu wina wa mowa pa tsiku lomweri? | Eya  Ayi  Refused to answer | Mwakusazgilapo pa ${drinktyp_1_str} na ${drinktyp_2_str}, mkumwaso mtundu unyakhe wamoba pa zuba leneliri? | Enya  Yayi  Refused to answer | Ask if in addition to the typical drink, participant takes another type of drink. |
| *DRINKTYP_3* | What type of alcohol is it? | 1  2  3  4  5  6  7  96 | Local beer (chipumu, khangala, chikokeyani, masese)  Local fruit wine "Wayini"  Kachasu  Chibuku  Bottled beer or cider (e.g. Carlsberg, Castel, Savannah)  Industrial/imported spirit (rum, whisky, gin, vodka etc)  Imported wine  Refused to answer | Kodi ndi mowa wanji? | Local beer (chipumu, khangala, chikokeyani, masese)  Local fruit wine "Wayini"  Kachasu  Chibuku  Bottled beer or cider (e.g. Carlsberg, Castel, Savannah)  Industrial/imported spirit (rum, whisky, gin, vodka etc)  Imported wine  Refused to answer | Kasi ni moba wuli? | Local beer (chipumu, khangala, chikokeyani, masese)  Local fruit wine "Wayini"  Kachasu  Chibuku  Bottled beer or cider (e.g. Carlsberg, Castel, Savannah)  Industrial/imported spirit (rum, whisky, gin, vodka etc)  Imported wine  Refused to answer | Record as responded. |
| *DRINKTYPICDAY_3* | How much of this do you drink on the same typical day? [REFER TO STANDARD DRINK TABLE] |  |  | Kodi mumamwa mowa wochuluka bwanji watundu umenewu pa tsiku lomweri? [REFER TO STANDARD DRINK TABLE] |  | Kasi mukumwa moba unandi uli wamutundu uwu pa zuba leneliri? [REFER TO STANDARD DRINK TABLE] |  | Refer to standard drink table to convert to intake to a standard drink. Use “GM ASSIST/AUDIT FORM USER GUIDE” on how to use standard drink table. |
| *SUMMARY_NOTE* | Total number of "standard drinks" on a typical day:  ${stddrinktotal}   [If incorrect, go back and change previous answers.] |  |  | Total number of "standard drinks" on a typical day  ${stddrinktotal}   [If incorrect, go back and change previous answers] |  | Total number of "standard drinks" on a typical day  ${stddrinktotal}  [If incorrect, go back and change previous answers.] |  | The tablet will calculate total number of standard drinks based on intake. |
| *ALC6FREQ1* | In the last year, have there been some occasions when you have had at a lot of alcohol. I mean at least 5 bottles of Carlsberg, OR a 200ml bottle of kachasu/sprit OR 2 cartons of chibuku OR 2.5 bottles of “wayini” OR almost 1 bottle of imported wine OR a large number of drinks of different kinds? [NOTE: IF PERSON DOESN’T UNDERSTAND THEN REFER SPECIFICALLY TO THE TYPE(S) OF DRINK THAT THE PERSON SAID THAT THEY DRINK TYPICALLY] | 1  0  6 | Yes  No  Refused to answer | Mu miyezi khumi ndi iwiri yapitayi, panali nthawi zina pomwe mukamwa zakumwa zolezeletsa zambiri panthawi imodzi? Ndikuthanthauza kumwa mabotolo 5 kapena kupotsera apa a Carlsberg, kapena botololo la 200 ml la kachasu/sipiriti, makatoni awiri a chibuku, kapena mabotolo awiri ndi hafu ''wayini'' kapena pafupifupi botolo limodzi la wayini wopangidwa kuja kapena zakumwa zambiri zolezeletsa zosiyanasiyana? [NOTE: IF PERSON DOESN’T UNDERSTAND THEN REFER SPECIFICALLY TO THE TYPE(S) OF DRINK THAT THE PERSON SAID THAT THEY DRINK TYPICALLY] | Eya  Ayi  Refused to answer | Mu miyezi 12 yajumpha iyi pakaba nyengo izo mukamwa vyakumwa vyakuloweleska vinandi panyengo imoza? Nkhung'anamula kumwa mabotolo 5 ya Carlsberg panji kujumpha, panyakhe botolo la 200ml la kachasu/masipiriti, panji makatoni yawiri ya chiBuku, panji mabotolo ya wiri na hafu ya ''wayini'' panji pafupifupi botolo limoza la wayini wakupangika kuwalo panji kumwa vyakumwa vyakuloweleska vyakupAmbanapambana vinandi? [NOTE: IF PERSON DOESN’T UNDERSTAND THEN REFER SPECIFICALLY TO THE TYPE(S) OF DRINK THAT THE PERSON SAID THAT THEY DRINK TYPICALLY] | Enya  Yayi  Refused to answer | Read as written. Refer to their typical drink if they do not understand question. |
| *ALC6FREQ2* | How often do you do this? | 1  2  3  4  6 | Less than monthly (occasionally)  Monthly (1-3 times per month)  Weekly (1-3 times per week)  Daily or almost daily (4 or more times per week)  Refused to answer | Izi mumapanga mowirikiza bwanji? | Kochepera mwezi ndi mwezi (mwapanthawi)  Mwezi ndi mwezi (kamodzi fukikira katatu pa mwezi)  Sabata ndi sabata(kamozi kufikira katatu pa sabata)  Tsiku ndi tsiku kapena pafupifupi tsiku lili lonse (kanai kapena kupotsera apo pa sabata)  Refused to answer | Ivi mukuchitanga mwapafupipafupi uli? | Kuchepera mwezi na mwezi (panyengo)  Mwezi na mwezi (kamoza kufikira katatu pa mwezi)  Sabata na sabata (kamoza kufikira katatu pa sabata)  Zuwa na zuwa panji pafupifupi zuwa lililose (kanayi panji kujumphizga apa pa sabata)  Refused to answer | Questions below are asked to participants who agreed to taking alcohol in excess. Record as responded. |
| *ALCNOSTOP* | How often during the last year have you found that you were not able to stop drinking once you had started? | 0  1  2  3  4  6 | Never  Less than monthly (occasionally)  Monthly (1-3 times per month)  Weekly (1-3 times per week)  Daily or almost daily (4 or more times per week)  Refused to answer | Ndimowirikiza bwanji mu m'miyezi 12 yapitayi pamene mumapezeka kuti mukangoyamba kumwa zakumwa zoledzeretsa simumatha kusiya? | Palibe  Kochepera mwezi ndi mwezi (mwapanthawi)  Mwezi ndi mwezi (kamodzi fukikira katatu pa mwezi)  Sabata ndi sabata(kamozi kufikira katatu pa sabata)  Tsiku ndi tsiku kapena pafupifupi tsiku lili lonse (kanai kapena kupotsera apo pa sabata)  Refused to answer | Mu miyezi 12 yajumpha iyi, nkhanandi wuli apomukasanga kuti pala mwayamba kumwa mukatondekanga kulekelezga? | Palije  Kuchepera mwezi na mwezi (panyengo)  Mwezi na mwezi (kamoza kufikira katatu pa mwezi)  Sabata na sabata (kamoza kufikira katatu pa sabata)  Zuwa na zuwa panji pafupifupi zuwa lililose (kanayi panji kujumphizga apa pa sabata)  Refused to answer | Record as responded. |
| *ALCFAILEXP* | How often during the last year have you failed to do what was normally expected from you because of drinking? | 0  1  2  3  4  6 | Never  Less than monthly (occasionally)  Monthly (1-3 times per month)  Weekly (1-3 times per week)  Daily or almost daily (4 or more times per week)  Refused to answer | Ndimowirikiza bwanji, mu m'miyezi 12 yapitayi pamene munalephera kuchita zimene mumayenera kuchita nthawi zonse chifukwa cha kumwa kwanu kwa mowa? | Palibe  Kochepera mwezi ndi mwezi (mwapanthawi)  Mwezi ndi mwezi (kamodzi fukikira katatu pa mwezi)  Sabata ndi sabata(kamozi kufikira katatu pa sabata)  Tsiku ndi tsiku kapena pafupifupi tsiku lili lonse (kanai kapena kupotsera apo pa sabata)  Refused to answer | Mu miyezi 12 yajumpha iyi, nkhanandi wuli apo mukatondekanga kupanga ivyo ŵanthu ŵakukhazga kufuma kwa imwe chifukwa cha kumwa kwinu kwa moba? | Palije  Kuchepera mwezi na mwezi (panyengo)  Mwezi na mwezi (kamoza kufikira katatu pa mwezi)  Sabata na sabata (kamoza kufikira katatu pa sabata)  Zuwa na zuwa panji pafupifupi zuwa lililose (kanayi panji kujumphizga apa pa sabata)  Refused to answer | Record as responded. |
| *ALCMORN* | How often during the last year have you needed a first drink in the morning to get yourself going after a heavy drinking session? | 0  1  2  3  4  6 | Never  Less than monthly (occasionally)  Monthly (1-3 times per month)  Weekly (1-3 times per week)  Daily or almost daily (4 or more times per week)  Refused to answer | Ndimowirikiza bwanji, mu m'miyezi 12 yapitayi pamene choyambirira m’mawa mumafuna kumwa chakumwa choledzeretsa kuti muthe kukwaniritsa kuchita zinthu chifukwa dzulo lake munamwa kwambiri? | Palibe  Kochepera mwezi ndi mwezi (mwapanthawi)  Mwezi ndi mwezi (kamodzi fukikira katatu pa mwezi)  Sabata ndi sabata(kamozi kufikira katatu pa sabata)  Tsiku ndi tsiku kapena pafupifupi tsiku lili lonse (kanai kapena kupotsera apo pa sabata)  Refused to answer | Mu miyezi 12 yajumpha iyi, nkhanandi wuli apo mukakhumbanga chakumwa chakuloweleska chakwamba namlenji kuti mukwaniliske kuchita vinthu chifukwa chakuti mayilo yakhe mukamwa chomene? | Palije  Kuchepera mwezi na mwezi (panyengo)  Mwezi na mwezi (kamoza kufikira katatu pa mwezi)  Sabata na sabata (kamoza kufikira katatu pa sabata)  Zuwa na zuwa panji pafupifupi zuwa lililose (kanayi panji kujumphizga apa pa sabata)  Refused to answer | Record as responded. |
| *ALCGUILT* | How often during the last year have you had a feeling of guilt or remorse after drinking? | 0  1  2  3  4  6 | Never  Less than monthly (occasionally)  Monthly (1-3 times per month)  Weekly (1-3 times per week)  Daily or almost daily (4 or more times per week)  Refused to answer | Ndimowirikiza bwanji, mu m'miyezi 12 yapitayi pamene munamva kutsutsika kapena kudzimvera chisoni pamapeto pa kumwa? | Palibe  Kochepera mwezi ndi mwezi (mwapanthawi)  Mwezi ndi mwezi (kamodzi fukikira katatu pa mwezi)  Sabata ndi sabata(kamozi kufikira katatu pa sabata)  Tsiku ndi tsiku kapena pafupifupi tsiku lili lonse (kanai kapena kupotsera apo pa sabata)  Refused to answer | Mu miyezi 12 yajumpha iyi, nkhanandi wuli apo mukaŵapo na kapulikiro kakuti mwananga panji kujiyeruzga pamanyuma pakumwa? | Palije  Kuchepera mwezi na mwezi (panyengo)  Mwezi na mwezi (kamoza kufikira katatu pa mwezi)  Sabata na sabata (kamoza kufikira katatu pa sabata)  Zuwa na zuwa panji pafupifupi zuwa lililose (kanayi panji kujumphizga apa pa sabata)  Refused to answer | Record as responded. |
| *ALCREMEM* | How often during the last year have you been unable to remember what happened the night before because you had been drinking? | 0  1  2  3  4  6 | Never  Less than monthly (occasionally)  Monthly (1-3 times per month)  Weekly (1-3 times per week)  Daily or almost daily (4 or more times per week)  Refused to answer | Ndimowirikiza bwanji, mu m'miyezi 12 yapitayi pamene simumatha kukumbukira zimene zinachitika usiku wathawo chifukwa munali mutamwa mowa? | Palibe  Kochepera mwezi ndi mwezi (mwapanthawi)  Mwezi ndi mwezi (kamodzi fukikira katatu pa mwezi)  Sabata ndi sabata(kamozi kufikira katatu pa sabata)  Tsiku ndi tsiku kapena pafupifupi tsiku lili lonse (kanai kapena kupotsera apo pa sabata)  Refused to answer | Mu miyezi 12 yajumpha iyi, nkhanandi wuli apo mukatondeka kukumbuka ivyo vikachitika mu usiku wamala chifukwa mukaŵa kuti mukamwa moba? | Palije  Kuchepera mwezi na mwezi (panyengo)  Mwezi na mwezi (kamoza kufikira katatu pa mwezi)  Sabata na sabata (kamoza kufikira katatu pa sabata)  Zuwa na zuwa panji pafupifupi zuwa lililose (kanayi panji kujumphizga apa pa sabata)  Refused to answer | Record as responded. |
| *ALCPASTPRESNTNOTE* | Now I am going to ask you some more questions related to your use of alcoholic beverages in the PAST or PRESENT. If you no longer use alcohol at all, please think back to your past use of alcohol and answer the next questions based on that. |  |  | Tsopano ndikufunsani mafunso ena wokhudzana ndi kumwa mowa kwanu M’MBUYOMU kapena PAKALI PANO. Ngati pakali pano simumamwanso wowa, ganizirani m’mbuyomu pamene mumamwa mowa ndi kuyankha mafunso wotsatira potengera zimenezo. |  | Sono ndimfumbaninge mafumbo yakukhwaskana naumo mukamwelanga moba mu nyengo za KUMASINDA panji KWASONO. Usange muli kulekelathu, chonde ghanaghanani umo mukamwelanga moba kale na kuzgola mafumbo yakulondezgapo kugwiriska ntchito vyenevivyo. |  | Read note exactly as written. Remind participant that this includes a time when they used to take alcohol, if they no longer take alcohol now. |
| ***GRISKRESIL*** | **RISK AND RESILIENCE** |  |  | **RISK AND RESILIENCE** |  | **RISK AND RESILIENCE** |  |  |
| ***EPQRA*** | **EPQR-A Neuroticism Scale** |  |  | **EPQR-A Neuroticism Scale** |  | **EPQR-A Neuroticism Scale** |  |  |
| *EPQRA_INTRO* | We would now like to find out about your personality. Personality is the unique tendency to feel and behave in a certain manner that is relatively stable over time. For example, some people are outgoing and like to spend a lot of time doing social activities whereas other people prefer to stay quiet and to be with only their family or by themselves. Some people follow routines and would always to go to church every week for example, whereas other people may be reckless. Some individuals may tend to worry or be nervous whereas others don’t get bothered by problems in their life and are optimistic. These are examples of different personality traits.  I am now going to ask you some questions about your usual personality. |  |  | Tsopano tikufuna tidziwe zakhalidwe lanu. Khalidwe ndi umo tichitira/tikhalira pa moyo wathu wa tsiku ndi tsiku. Mwachitsanzo anthu ena ndi ochezeka ndipo amakonda nthawi yochuluka kuchita za mchezo pomwe ena ndi a phee ndipo amakonda kukhala ndi abale awo okha kapenanso kukhala okha. Anthu ena amachita zinthu mwandondomeko nthawi zonse monga amapita ku tchalitchi sabata lirilonse pomwe ena salabadira kanthu. Anthu ena amakhala odandauladandaula kapena a mantha, kusowa mtendere pomwe ena salabadira za mabvuto m’moyo mwawo, amakhala achiyembekezo. Izi ndiye zitsanzo zamakhalidwe osiyanasiyana.  Tsopano ndikufunsani mafunso ena okhuza khalidwe lanu lanthawi zonse. |  | Sono tikukhumba kumanya vya wunthu winu . Wunthu (nkhalo zamunthu) nikabilo kamunthu kapadela ako kakumupangiska munthu kupulika nakuchita vinthu munthowa yinyakhe kwanyengo yitaliko. Mwachiyezgelelo banthu wanyakhe bakuba nantchezgo ndipo bakutemwa kutaya nyengo yawo pa vyakuchitika vya ntchezgo apo banthu wanyakhe bakutemwa kuwa chete waka nakutemwa kukhala pafupi na wa bale wawo pela panji kujikhalira pawekha. Banthu wanyakhe wakulondezga ndondomeko nyengo zose mwachiyezgelelo kuluta kutchalitchi sabata na sabata apo wanji wakukhala naumoyo wachisawawa. Wanji wakuya naumoyo wakudandaula panji kuwavya mtende apo wanyakhe bakupwelera yayi namasuzgo paumoyo wawo ndipo bakuwa nachikhazgo.  Sono nimufumbeninge mafumbo ghanyakhe yakukhwaskana na nkhalo yinu yanyengo zose. |  | This section asks participants about neuroticism, which is a personality trait where one has a tendency of being in a negative emotional state including worrying. Read note exactly as written. |
| *EPQRAMOOD* | Does your mood often go up and down? | 1  0  8  6 | Yes  No  Don't Know  Refused to answer | Kodi khalidwe (mudi) lanu limasinthasintha, kukwera ndi ndikutsika kawirikawiri? | Eya  Ayi  Sindikudziwa  Refused to answer | Kasi kabilo kinu kakusinthasintha, kukwiya na kuzika kawirikawiri? | Enya  Yayi  Nkhumanya yayi  Refused to answer | Record as responded. |
| *EPQRANERVOUSPERS* | Would you call yourself a nervous person? | 1  0  8  6 | Yes  No  Don't Know  Refused to answer | Kodi munganene kuti ndinu munthu wa mantha, nkhawa, wamanjenje, nthumazi kapena wosowa mtendele? | Eya  Ayi  Sindikudziwa  Refused to answer | Kasi mungajiyowoya kuti muli wa wofi, wa kudandaula, panji wambula mtende? | Enya  Yayi  Nkhumanya yayi  Refused to answer | Record as responded. |
| *EPQRALONELY* | Do you often feel lonely, as if you are without friends or family? | 1  0  8  6 | Yes  No  Don't Know  Refused to answer | Kodi kawirikawiri mumamva kusungulumwa kapena kuti muli nokha opanda mbale kapena mzanu? | Eya  Ayi  Sindikudziwa  Refused to answer | Kasi ni mwakawirikawiri mukujipulika kuti muli mwekha nga mulije wabale panji wabwezi? | Enya  Yayi  Nkhumanya yayi  Refused to answer | Record as responded. |
| *EPQRANERVES* | Do you suffer from "nerves"? | 1  0  8  6 | Yes  No  Don't Know  Refused to answer | Kodi mumavutika ndikuchita mantha, nkhawa, kapena kusowa mtendere? | Eya  Ayi  Sindikudziwa  Refused to answer | Kasi mukusugzikanga na wofi, kudandaula, panji kuwavya mtende? | Enya  Yayi  Nkhumanya yayi  Refused to answer | Record as responded. |
| *EPQRAFEDUP* | Do you often feel "fed-up"? | 1  0  8  6 | Yes  No  Don't Know  Refused to answer | Kodi mumatopetsedwa ndi zochitika kapena kutaya mtima kawirikawiri? | Eya  Ayi  Sindikudziwa  Refused to answer | Kasi mukuvukanga navyochitika nakukwenyerezgeka pafupi pafupi? | Enya  Yayi  Nkhumanya yayi  Refused to answer | Record as responded. |
| *EPQRAWORRIER* | Are you a worrier? | 1  0  8  6 | Yes  No  Don't Know  Refused to answer | Kodi ndinu odandauladandaula (kapena oliralira)? | Eya  Ayi  Sindikudziwa  Refused to answer | Kasi ndimwe munthu wakudandauladandaula? | Enya  Yayi  Nkhumanya yayi  Refused to answer | Record as responded. |
| ***MSPSS*** | **Multi-dimensional Scale of Perceived Social Support (MSPSS)** |  |  | **Multi-dimensional Scale of Perceived Social Support (MSPSS)** |  | **Multi-dimensional Scale of Perceived Social Support (MSPSS)** |  |  |
| *MSPSS_INTRO* | Now, I would like to ask you questions about support that you get from other people. For these questions, you should give the answer that best applies to you using this picture. [SHOW THE RATING CARD] This face means totally disagree, this face means partially disagree, the one in the middle means neutral, this one means partially agree and the last one means totally agree that the statement is true for you.  Some of the questions ask about a “Special Person”. This is someone to whom you feel very close emotionally. It usually refers to spouse or boyfriend/girlfriend, though could be another person to whom you are particularly emotionally close. |  |  | Tsopano ndikufuna ndikufunseni mafunso okhuzana ndi chithandizo chomwe mwakhala mukulandira kuchokela kwa anthu ena. Pa funso lirilonse, munene m’mene mukuonera kuti ndi zoona kwainuyo pogwiritsa ntchito zithunzi izi. NDIKUONETSANI NKHOPE: Nkhope iyi ikuimila kuti simukugwirizana nazo kwathunthu kuti ndi zoona, iyi ikuimila kuti simukugwirizana nazo pang’ono, yapakatiyi ikuimila kuti mulibe mbali (simukugwirizana nazo kapena kugwirizana nazo), iyi ikuimila kuti mukugwirizana nazo pang’ono ndipo iyi ikuimila kuti mukugwirizana nazo kwambiri, kuti yankho la funsolo ndi loona kwa inuyo.  Mafunso ena afunsa za "munthu wapaderadera". Uyu ndi munthu yemwe mumamva kuti ndi wapafupi kwambiri kwa inu. Kawirikawiri amakhala mwamuna/mkazi wanu kapena wachibwenzi, athanso kukhala munthu wina wapafupi nanu. |  | Sono ndimufumbeninge mafumbo yakukhwaskana na wovwiri uwo mwakhala mukupokera kufuma kwa banthu. Pa fumbo lililose, muyowoyenge naumo mukuonera unenesko wake kwa imwe. NDIMULONGOLANINGE VYISKO: Chisko ichi chikwimilila kuleka kuzomerezgana navyo nkhanila, ichi chikwimilila kuleka kuzomerezgana navyo pachoko, chapakati ichi chikwimilila kuti mulije lwande (mukuzomerezga panji kukana yayi). Ichi chikwimilila kuzomerezgana navyo pachoko ndipo chaumaliro ichi chikwimilila kuzomerezga chomene, kuti zgolo la fumbo ndaunenesko kwa imwe.  Mafumbo yanyakhe yafumbenge “wakukhumbikila". Uyu ni munthu uyo ngwapafupi chomene muvyakuchitika kwaimwe. Angaba mfumu/muwoli winu panji chibwezi chinu, nanga wuli angawa munthu yunji uyo angawa wapafupi chomene naimwe. |  | This section to ask participants about their perceived social support. Interviewer should assess whether a participant will be able to use the visual and explain to how the facial rating card is used. If the interviewer thinks the participant will have difficulty using the visual aid, the options should be read out to them. Read the note exactly as written. |
| *INNEED1MON* | How much do you agree or disagree with the following statement: In the last month there has been a special person who is around when I am in need? | 0  1  2  3  4  6 | Strongly Disagree  Mildly Disagree  Neutral  Mildly Agree  Strongly Agree  Refused to answer | Mukugwirizana nazo kapena mukukana motani pa chiganizo ichi: Mwezi wathawu panali munthu wapaderadera m’moyo mwanga amene angathe kundithandiza pamene ndikufuna chithandizo? | Sindikugwirizana nazo kwatunthu  Sindikugwirizana nazo pang’ono  Ndiribe mbali  Ndikugwirizana nazo pang’ono  Ndikugwirizana nazo kwambiri  Refused to answer | Mukuzomelezgana navyo panji mukukana wuli pafundo iyi: Mumwezi wamala uwu pakaba munthu wakukhumbikira m’moyo wane uyo wangamanya kundovwira usange pakukhumbikila wovwiri? | Nkhuzomerezgana navyo yayi nkhanila  Nkhuzomerezgana navyo yayi pachoko  Nilije lwande/chigaba  Nkhuzomemerezgana navyo pachoko  Nkhuzomemerezgana navyo chomene  Refused to answer | Record as reported. |
| *JOYSORRW1MON* | How much do you agree or disagree with the following statement: In the last month there has been a special person with whom I can share joys and sorrows? | 0  1  2  3  4  6 | Strongly Disagree  Mildly Disagree  Neutral  Mildly Agree  Strongly Agree  Refused to answer | Mukugwirizana nazo kapena mukukana motani pa chiganizo ichi: Mwezi wathawu panali munthu wapaderadera m’moyo mwanga amene ndingathe kugawana naye chisangalalo ndi chisoni changa? | Sindikugwirizana nazo kwatunthu  Sindikugwirizana nazo pang’ono  Ndiribe mbali  Ndikugwirizana nazo pang’ono  Ndikugwirizana nazo kwambiri  Refused to answer | Mukuzomelezgana navyo panji mukukana wuli pafundo iyi: Mumwezi wamala uwu pakaba munthu wakukhumbikira m’moyo mwane uyo ningagawana nayo kukondwa kwane na vitima vyane? | Nkhuzomerezgana navyo yayi nkhanila  Nkhuzomerezgana navyo yayi pachoko  Nilije lwande/chigaba  Nkhuzomemerezgana navyo pachoko  Nkhuzomemerezgana navyo chomene  Refused to answer | Record as responded. |
| *FAMHELP1MON* | How much do you agree or disagree with the following statement: In the last month, my family has really tried to help me? | 0  1  2  3  4  6 | Strongly Disagree  Mildly Disagree  Neutral  Mildly Agree  Strongly Agree  Refused to answer | Mukugwirizana nazo kapena mukukana motani pa chiganizo ichi: Mwezi wathawu abale anga amayesetsa ndithu kundithandiza? | Sindikugwirizana nazo kwatunthu  Sindikugwirizana nazo pang’ono  Ndiribe mbali  Ndikugwirizana nazo pang’ono  Ndikugwirizana nazo kwambiri  Refused to answer | Mukuzomelezgana navyo panji mukukana wuli pafundo iyi: Mumwezi wamala uwu babale bane bakayezgeska kundovwira? | Nkhuzomerezgana navyo yayi nkhanila  Nkhuzomerezgana navyo yayi pachoko  Nilije lwande/chigaba  Nkhuzomemerezgana navyo pachoko  Nkhuzomemerezgana navyo chomene  Refused to answer | Record as responded. |
| *EMOTHELP1MON* | How much do you agree or disagree with the following statement: In the last month, I have got the emotional help & support I need from my family? | 0  1  2  3  4  6 | Strongly Disagree  Mildly Disagree  Neutral  Mildly Agree  Strongly Agree  Refused to answer | Mukugwirizana nazo kapena mukukana motani pa chiganizo ichi: Mwezi wathawu ndimapeza mtendere wa mumtima kuchokera kwa abale anga? | Sindikugwirizana nazo kwatunthu  Sindikugwirizana nazo pang’ono  Ndiribe mbali  Ndikugwirizana nazo pang’ono  Ndikugwirizana nazo kwambiri  Refused to answer | Mukuzomelezgana navyo panji mukukana wuli pafundo iyi: Mumwezi wamala uwu nkhasanga mtende wa mu mtima kufumira kwa wabale wane? | Nkhuzomerezgana navyo yayi nkhanila  Nkhuzomerezgana navyo yayi pachoko  Nilije lwande/chigaba  Nkhuzomemerezgana navyo pachoko  Nkhuzomemerezgana navyo chomene  Refused to answer | Record as responded. |
| *COMFORT1MON* | How much do you agree or disagree with the following statement: In the last month I have had a special person who is a real source of comfort to me? | 0  1  2  3  4  6 | Strongly Disagree  Mildly Disagree  Neutral  Mildly Agree  Strongly Agree  Refused to answer | Mukugwirizana nazo kapena mukukana motani pa chiganizo ichi: Mwezi wathawu ndinali ndi munthu wapaderadera m’moyo mwanga amene amanditonthoza? | Sindikugwirizana nazo kwatunthu  Sindikugwirizana nazo pang’ono  Ndiribe mbali  Ndikugwirizana nazo pang’ono  Ndikugwirizana nazo kwambiri  Refused to answer | Mukuzomelezgana navyo panji mukukana wuli pafundo iyi: Mumwezi wamala uwu nkhawa namunthu wakukhumbikira m’moyo wane uyo wakunipembuzganga? | Nkhuzomerezgana navyo yayi nkhanila  Nkhuzomerezgana navyo yayi pachoko  Nilije lwande/chigaba  Nkhuzomemerezgana navyo pachoko  Nkhuzomemerezgana navyo chomene  Refused to answer | Record as responded. |
| *FRNDHELP1MON* | How much do you agree or disagree with the following statement: In the last month, my friends have really tried to help me? | 0  1  2  3  4  6 | Strongly Disagree  Mildly Disagree  Neutral  Mildly Agree  Strongly Agree  Refused to answer | Mukugwirizana nazo kapena mukukana motani pa chiganizo ichi: Mwezi wathawu anzanga amayesetsa ndithu kundithandiza? | Sindikugwirizana nazo kwatunthu  Sindikugwirizana nazo pang’ono  Ndiribe mbali  Ndikugwirizana nazo pang’ono  Ndikugwirizana nazo kwambiri  Refused to answer | Mukuzomelezgana navyo panji mukukana wuli pafundo iyi: Mumwezi wamala uwu banyane bakayezgeska kundovwira? | Nkhuzomerezgana navyo yayi nkhanila  Nkhuzomerezgana navyo yayi pachoko  Nilije lwande/chigaba  Nkhuzomemerezgana navyo pachoko  Nkhuzomemerezgana navyo chomene  Refused to answer | Record as responded. |
| *FRNDWRONG1MON* | How much do you agree or disagree with the following statement: In the last month, I have been able to count on my friends when things go wrong? | 0  1  2  3  4  6 | Strongly Disagree  Mildly Disagree  Neutral  Mildly Agree  Strongly Agree  Refused to answer | Mukugwirizana nazo kapena mukukana motani pa chiganizo ichi: Mwezi wathawu ndimatha kudalira anzanga zinthu zikavuta? | Sindikugwirizana nazo kwatunthu  Sindikugwirizana nazo pang’ono  Ndiribe mbali  Ndikugwirizana nazo pang’ono  Ndikugwirizana nazo kwambiri  Refused to answer | Mukuzomelezgana navyo panji mukukana wuli pafundo iyi: Mumwezi wamala uwu nkhagomezganga banyane pala vinthu vyasuzga? | Nkhuzomerezgana navyo yayi nkhanila  Nkhuzomerezgana navyo yayi pachoko  Nilije lwande/chigaba  Nkhuzomemerezgana navyo pachoko  Nkhuzomemerezgana navyo chomene  Refused to answer | Record as responded. |
| *FAMTLKPROB1MON* | How much do you agree or disagree with the following statement: In the last month, I have been able to talk about my problems with my family? | 0  1  2  3  4  6 | Strongly Disagree  Mildly Disagree  Neutral  Mildly Agree  Strongly Agree  Refused to answer | Mukugwirizana nazo kapena mukukana motani pa chiganizo ichi: Mwezi wathawu ndimatha kukamba za mavuto anga ndi abale anga? | Sindikugwirizana nazo kwatunthu  Sindikugwirizana nazo pang’ono  Ndiribe mbali  Ndikugwirizana nazo pang’ono  Ndikugwirizana nazo kwambiri  Refused to answer | Mukuzomelezgana navyo panji mukukana wuli pafundo iyi: Mumwezi wamala uwu nkhakwaniskanga kubaphalira wabale wane za masuzgo yane? | Nkhuzomerezgana navyo yayi nkhanila  Nkhuzomerezgana navyo yayi pachoko  Nilije lwande/chigaba  Nkhuzomemerezgana navyo pachoko  Nkhuzomemerezgana navyo chomene  Refused to answer | Record as responded. |
| *FRNDJOYSORRW1MON* | How much do you agree or disagree with the following statement: In the last month, I have had friends with whom I can share my joys and sorrows? | 0  1  2  3  4  6 | Strongly Disagree  Mildly Disagree  Neutral  Mildly Agree  Strongly Agree  Refused to answer | Mukugwirizana nazo kapena mukukana motani pa chiganizo ichi: Mwezi wathawu ndinali ndi anzanga omwe ndingagawane nawo chisangalalo changa ndi chisoni changa? | Sindikugwirizana nazo kwatunthu  Sindikugwirizana nazo pang’ono  Ndiribe mbali  Ndikugwirizana nazo pang’ono  Ndikugwirizana nazo kwambiri  Refused to answer | Mukuzomelezgana navyo panji mukukana wuli pafundo iyi: Mumwezi wamala uwu nkhaba nawo banyane awo nkhagabananga nawo kukondwa kwane na vitima vyane? | Nkhuzomerezgana navyo yayi nkhanila  Nkhuzomerezgana navyo yayi pachoko  Nilije lwande/chigaba  Nkhuzomemerezgana navyo pachoko  Nkhuzomemerezgana navyo chomene  Refused to answer | Record as responded. |
| *FEELINGS1MON* | How much do you agree or disagree with the following statement: In the last month, there has been a special person in my life who cares about my feelings? | 0  1  2  3  4  6 | Strongly Disagree  Mildly Disagree  Neutral  Mildly Agree  Strongly Agree  Refused to answer | Mukugwirizana nazo kapena mukukana motani pa chiganizo ichi: Mwezi wathawu panali munthu wapaderadera m’moyo mwanga amene amasamala za momwe ndikumvera? | Sindikugwirizana nazo kwatunthu  Sindikugwirizana nazo pang’ono  Ndiribe mbali  Ndikugwirizana nazo pang’ono  Ndikugwirizana nazo kwambiri  Refused to answer | Mukuzomelezgana navyo panji mukukana wuli pafundo iyi: Mumwezi wamala uwu wakabapo munthu wakukhumbikira m’moyo wane uyo wakukhwaskika na vya umo nkhujipulikila? | Nkhuzomerezgana navyo yayi nkhanila  Nkhuzomerezgana navyo yayi pachoko  Nilije lwande/chigaba  Nkhuzomemerezgana navyo pachoko  Nkhuzomemerezgana navyo chomene  Refused to answer | Record as responded. |
| *DECISIONS1MON* | How much do you agree or disagree with the following statement: In the last month, my family has been willing to help me make decisions? | 0  1  2  3  4  6 | Strongly Disagree  Mildly Disagree  Neutral  Mildly Agree  Strongly Agree  Refused to answer | Mukugwirizana nazo kapena mukukana motani pa chiganizo ichi: Mwezi wathawu abale anga amakhala okonzeka kundithangata kupanga ziganizo? | Sindikugwirizana nazo kwatunthu  Sindikugwirizana nazo pang’ono  Ndiribe mbali  Ndikugwirizana nazo pang’ono  Ndikugwirizana nazo kwambiri  Refused to answer | Mukuzomelezgana navyo panji mukukana wuli pafundo iyi: Mumwezi wamala uwu babale wane bakaba bakunozgeka kundovwira kupanga viganizo? | Nkhuzomerezgana navyo yayi nkhanila  Nkhuzomerezgana navyo yayi pachoko  Nilije lwande/chigaba  Nkhuzomemerezgana navyo pachoko  Nkhuzomemerezgana navyo chomene  Refused to answer | Record as responded. |
| *FRNDTLKPROB1MON* | How much do you agree or disagree with the following statement: In the last month, I have been able to talk about my problems with my friends? | 0  1  2  3  4  6 | Strongly Disagree  Mildly Disagree  Neutral  Mildly Agree  Strongly Agree  Refused to answer | Mukugwirizana nazo kapena mukukana motani pa chiganizo ichi: Mwezi wathawu ndimatha kuwauza anzanga za mavuto anga? | Sindikugwirizana nazo kwatunthu  Sindikugwirizana nazo pang’ono  Ndiribe mbali  Ndikugwirizana nazo pang’ono  Ndikugwirizana nazo kwambiri  Refused to answer | Mukuzomelezgana navyo panji mukukana wuli pafundo iyi: Mumwezi wamala uwu nkhakwaniskanga kubaphalira banyane vya masugzo yane? | Nkhuzomerezgana navyo yayi nkhanila  Nkhuzomerezgana navyo yayi pachoko  Nilije lwande/chigaba  Nkhuzomemerezgana navyo pachoko  Nkhuzomemerezgana navyo chomene  Refused to answer | Record as responded. |
| *SPECPERSON* | If you said that there is a “special person” in your life, what is the relationship to you of this person? | 0  1  2  3  4  5  6  7  8  96 | No special person  Spouse (married)  Boyfriend/Girlfriend  Mother  Father  Brother  Sister  Other relative  Other non-relative  Refused to answer | Ngati munanena kuti pali munthu wapaderadera m’moyo wanu, Kodi ndindani wanu? | Palibe munthu wapaderadera  Mwamuna/mkazi wanga (banja)  Chibwenzi  Amayi  Atate  Mchimwene  Mchemwali  Wachibale wina  Wina koma osati wachibale  Refused to answer | Pala mwanguyowoya kuti walipo munthu wakukhumbikira mumoyo winu, kasi mbanjani winu? | Palije munthu movwili chomene kwaine apa  Mfumu/muwoli (nthengwa)  Chibwezi  Mama  Dada  Mwanamunyane (mwanalume)  Mwana munyane (mwanakazi)  Mubale munyakhe  Munyakhe kwenu mubale yayi  Refused to answer | Record as responded. |
| ***HFIAS*** | **FOOD INSECURITY (HFIAS)** |  |  | **FOOD INSECURITY (HFIAS)** |  | **FOOD INSECURITY (HFIAS)** |  |  |
| *HFIAS_INTRO* | The following questions ask about whether or not you have had enough food in your houshold in the last 4 weeks. |  |  | Mafunso otsatirawa akufunsa ngati munali kapena munalibe chakudya chokwanira munyumba mwanu m’masabata anayi apitawa. |  | Mafumbo yakulondezga aya yafumbenge usange mukawa nacho panji mukabavye chakulya chakukwanila panyumba yinu mumasabata yanayi ayo yajumpha. |  | This section asks about food security in the participants household. Read note exactly as written. |
| *WORRYFOOD* | In the past 4 weeks, did you worry that your household would not have enough food? | 1  0  8  6 | Yes  No  Don't Know  Refused to answer | Pa masabata anayi omwe apitawa, kodi mudakhalapo ndi nkhawa chifukwa choti mukhala ndi chakudya choperewera panyumba yanu pano? | Eya  Ayi  Sindikudziwa  Refused to answer | Pamasabata yanayi ayo yajumpha, kasi mukabapo wakwenjerwa kuti chakurya chichepenge panyumba pinu? | Enya  Yayi  Nkhumanya yayi  Refused to answer | Record as responded. |
| *WORRYFOODFREQ* | How often did this happen? | 1  2  3  6 | Rarely (once or twice in the past 4 weeks)  Sometimes (three to ten times in the past 4 weeks)  Often (more than ten times in the past 4 weeks)  Refused to answer | Izi zinachitika mowirikiza bwanji? | Mwa apo ndi apo (Kamodzi kapena kawiri mu masabata anayi apitawa)  Nthawi zina (Kuchokera katatu kufikira ka teni mu masabata anayi apitawa)  Pafupi pafupi (Kuposera ka teni mu masabata anayi apitawa )  Refused to answer | Ivi vikachitikanga mwapafupi-pafupi wuli? | Munyengo munyengo/mwa apo na apo (kamoza panji kawili mumasabata yanayi ayo yajumpha)  Nyengo zinyakhe (katatu mupaka kateni mumasabata yanayi ayo yajumpha)  Mwapafupipafupi (kujumpha ka teni pamasabata yanayi ayo yajumpha)  Refused to answer | If yes to above question, record as responded. |
| *FOODPREFLACK* | In the past 4 weeks, were you or any household member not able to eat the kinds of foods you preferred because of a lack of resources? | 1  0  8  6 | Yes  No  Don't Know  Refused to answer | Pa masabata anayi omwe apitawa, alipo munyumba yanu yemwe adalephera kudya chakudya chomwe amafuna chifukwa chosowa zofunikira? | Eya  Ayi  Sindikudziwa  Refused to answer | Pamasabata yanayi ayo yajumpha, kasi imwe panji munyake walipo munyumba yinu uyo wakatondeka kurya chakurya icho wakachikhumbanga chifukwa cha kusowerwa kwa kasangilo? | Enya  Yayi  Nkhumanya yayi  Refused to answer | Record as responded. |
| *FOODPREFLACKFREQ* | How often did this happen? | 1  2  3  6 | Rarely (once or twice in the past 4 weeks)  Sometimes (three to ten times in the past 4 weeks)  Often (more than ten times in the past 4 weeks)  Refused to answer | Izi zinachitika mowirikiza bwanji? | Mwa apo ndi apo (Kamodzi kapena kawiri mu masabata anayi apitawa)  Nthawi zina (Kuchokera katatu kufikira ka teni mu masabata anayi apitawa)  Pafupi pafupi (Kuposera ka teni mu masabata anayi apitawa )  Refused to answer | Ivi vikachitikanga mwapafupi-pafupi wuli? | Munyengo munyengo/mwa apo na apo (kamoza panji kawili mumasabata yanayi ayo yajumpha)  Nyengo zinyakhe (katatu mupaka kateni mumasabata yanayi ayo yajumpha)  Mwapafupipafupi (kujumpha ka teni pamasabata yanayi ayo yajumpha)  Refused to answer | If yes to above question, record as responded. |
| *LIMITFOOD* | In the past 4 weeks, did you or any household member have to eat a limited variety of foods due to a lack of resources? | 1  0  8  6 | Yes  No  Don't Know  Refused to answer | Pa masabata anayi omwe apitawa, alipo munyumba yanu amene analephera kudya zakudya zosiyanasiyana chifukwa chopereweredwa pakhomo? | Eya  Ayi  Sindikudziwa  Refused to answer | Pamasabata yanayi ayo yajumpha, kasi imwe panji munyake walipo munyumba yinu uyo wakatondeka kurya vyakurya vyakupambanapambana chifukwa cha kusowerwa ndalama panji vinthu vinyakhe vyakusangira chakulya? | Enya  Yayi  Nkhumanya yayi  Refused to answer | Record as responded. |
| *LIMITFOODFREQ* | How often did this happen? | 1  2  3  6 | Rarely (once or twice in the past 4 weeks)  Sometimes (three to ten times in the past 4 weeks)  Often (more than ten times in the past 4 weeks)  Refused to answer | Izi zinachitika mowirikiza bwanji? | Mwa apo ndi apo (Kamodzi kapena kawiri mu masabata anayi apitawa)  Nthawi zina (Kuchokera katatu kufikira ka teni mu masabata anayi apitawa)  Pafupi pafupi (Kuposera ka teni mu masabata anayi apitawa )  Refused to answer | Ivi vikachitikanga mwapafupi-pafupi uli? | Munyengo munyengo/mwa apo na apo (kamoza panji kawili mumasabata yanayi ayo yajumpha)  Nyengo zinyakhe (katatu mupaka kateni mumasabata yanayi ayo yajumpha)  Mwapafupipafupi (kujumpha ka teni pamasabata yanayi ayo yajumpha)  Refused to answer | If yes to above question, record as responded. |
| *FOODNOTWANT* | In the past 4 weeks, did you or any household member have to eat some foods that you really did not want to eat because of a lack of resources to obtain other types of food? | 1  0  8  6 | Yes  No  Don't Know  Refused to answer | Pa masabata anayi omwe apitawa, alipo munyumba yanu amene anadya chakudya chomwe sanachifune chifukwa chosowa ndalama kapena zinthu zina zopezera zakudya zamitundu yina? | Eya  Ayi  Sindikudziwa  Refused to answer | Pamasabata yanayi ayo yajumpha, kasi imwe panji munyake walipo munyumba yinu uyo wakarya chakurya icho iyo wakhumbanga yayi kuti warye chifukwa cha kusoberwa kwa ndalama panji vinthu vinyakhe vyakusangira chakurya cha mitundu yinyakhe? | Enya  Yayi  Nkhumanya yayi  Refused to answer | Record as responded. |
| *FOODNOTWANTFREQ* | How often did this happen? | 1  2  3  6 | Rarely (once or twice in the past 4 weeks)  Sometimes (three to ten times in the past 4 weeks)  Often (more than ten times in the past 4 weeks)  Refused to answer | Izi zinachitika mowirikiza bwanji? | Mwa apo ndi apo (Kamodzi kapena kawiri mu masabata anayi apitawa)  Nthawi zina (Kuchokera katatu kufikira ka teni mu masabata anayi apitawa)  Pafupi pafupi (Kuposera ka teni mu masabata anayi apitawa )  Refused to answer | Ivi vikachitikanga mwapafupi-pafupi uli? | Munyengo munyengo/mwa apo na apo (kamoza panji kawili mumasabata yanayi ayo yajumpha)  Nyengo zinyakhe (katatu mupaka kateni mumasabata yanayi ayo yajumpha)  Mwapafupipafupi (kujumpha ka teni pamasabata yanayi ayo yajumpha)  Refused to answer | If yes to above question, record as responded. |
| *SMALLERMEAL* | In the past 4 weeks, did you or any household member have to eat a smaller meal than you felt you needed because there was not enough food? | 1  0  8  6 | Yes  No  Don't Know  Refused to answer | Pa masabata anayi omwe apitawa, alipo munyumba yanu amene anadya chakudya chochepera momwe chimayenera kukhalira chifukwa panalibe chakudya chokwanira? | Eya  Ayi  Sindikudziwa  Refused to answer | Pamasabata yanayi ayo yajumpha, kasi imwe panji munyake walipo munyumba yinu uyo wakalya chakulya chakuchepa kulusha umo chikenelela kubila kuti iyo warye chifukwa chakuti chakurya chikawa chichoko? | Enya  Yayi  Nkhumanya yayi  Refused to answer | Record as responded. |
| *SMALLERMEALFREQ* | How often did this happen? | 1  2  3  6 | Rarely (once or twice in the past 4 weeks)  Sometimes (three to ten times in the past 4 weeks)  Often (more than ten times in the past 4 weeks)  Refused to answer | Izi zinachitika mowirikiza bwanji? | Mwa apo ndi apo (Kamodzi kapena kawiri mu masabata anayi apitawa)  Nthawi zina (Kuchokera katatu kufikira ka teni mu masabata anayi apitawa)  Pafupi pafupi (Kuposera ka teni mu masabata anayi apitawa )  Refused to answer | Ivi vikachitikanga mwapafupi-pafupi uli? | Munyengo munyengo/mwa apo na apo (kamoza panji kawili mumasabata yanayi ayo yajumpha)  Nyengo zinyakhe (katatu mupaka kateni mumasabata yanayi ayo yajumpha)  Mwapafupipafupi (kujumpha ka teni pamasabata yanayi ayo yajumpha)  Refused to answer | If yes to above question, record as responded. |
| *FEWERMEAL* | In the past 4 weeks, did you or any household member have to eat fewer meals in a day because there was not enough food? | 1  0  8  6 | Yes  No  Don't Know  Refused to answer | Pa masabata anayi omwe apitawa, alipo munyumba yanu amene anadya chakudya kochepa (number of meals) pa tsiku chifukwa choti chakudya chinali chosakwanira? | Eya  Ayi  Sindikudziwa  Refused to answer | Pamasabata yanayi ayo yajumpha, kasi imwe panji munyake walipo munyumba yinu uyo wakalya chakurya mwakuchepa (number of meals) pa zuwa chifukwa chakuti chakurya chikawa chichoko? | Enya  Yayi  Nkhumanya yayi  Refused to answer | Record as responded. |
| *FEWERMEALFREQ* | How often did this happen? | 1  2  3  6 | Rarely (once or twice in the past 4 weeks)  Sometimes (three to ten times in the past 4 weeks)  Often (more than ten times in the past 4 weeks)  Refused to answer | Izi zinachitika mowirikiza bwanji? | Mwa apo ndi apo (Kamodzi kapena kawiri mu masabata anayi apitawa)  Nthawi zina (Kuchokera katatu kufikira ka teni mu masabata anayi apitawa)  Pafupi pafupi (Kuposera ka teni mu masabata anayi apitawa )  Refused to answer | Ivi vikachitikanga mwapafupi-pafupi uli? | Munyengo munyengo/mwa apo na apo (kamoza panji kawili mumasabata yanayi ayo yajumpha)  Nyengo zinyakhe (katatu mupaka kateni mumasabata yanayi ayo yajumpha)  Mwapafupipafupi (kujumpha ka teni pamasabata yanayi ayo yajumpha)  Refused to answer | If yes to above question, record as responded. |
| *NOFOOD* | In the past 4 weeks, was there ever no food to eat of any kind in your household because of lack of resources to get food? | 1  0  8  6 | Yes  No  Don't Know  Refused to answer | Pa masabata anayi omwe apitawa, yinalipo nthawi yoti panalibiletu chakudya chili chonse panyumba yanu chifukwa choti panalibe ndalama kapena zinthu zopezera chakudya? | Eya  Ayi  Sindikudziwa  Refused to answer | Pamasabata yanayi ayo yajumpha, vilikuchitikapo kuti mukawavya chakulya chili chose chifukwa chakusowerwa ndalama panji vinthu vinyakhe vyakusangila chakulya? | Enya  Yayi  Nkhumanya yayi  Refused to answer | Record as responded. |
| *NOFOODFREQ* | How often did this happen? | 1  2  3  6 | Rarely (once or twice in the past 4 weeks)  Sometimes (three to ten times in the past 4 weeks)  Often (more than ten times in the past 4 weeks)  Refused to answer | Izi zinachitika mowirikiza bwanji? | Mwa apo ndi apo (Kamodzi kapena kawiri mu masabata anayi apitawa)  Nthawi zina (Kuchokera katatu kufikira ka teni mu masabata anayi apitawa)  Pafupi pafupi (Kuposera ka teni mu masabata anayi apitawa )  Refused to answer | Ivi vikachitikanga mwapafupi-pafupi uli? | Munyengo munyengo/mwa apo na apo (kamoza panji kawili mumasabata yanayi ayo yajumpha)  Nyengo zinyakhe (katatu mupaka kateni mumasabata yanayi ayo yajumpha)  Mwapafupipafupi (kujumpha ka teni pamasabata yanayi ayo yajumpha)  Refused to answer | If yes to above question, record as responded. |
| *SLEEPHUNGRY* | In the past 4 weeks, did you or any household member go to sleep at night hungry because there was not enough food? | 1  0  8  6 | Yes  No  Don't Know  Refused to answer | Pa masabata anayi omwe apitawa, alipo panyumba yanu pano, amene anagona ndi njala chifukwa choti panalibe chakudya chokwanira? | Eya  Ayi  Sindikudziwa  Refused to answer | Pamasabata yanayi ayo yajumpha, kasi imwe panji munyake walipo munyumba yinu uyo walikugonapo nanjala chifukwa chakuti chakulya chikaba chambura kukwana? | Enya  Yayi  Nkhumanya yayi  Refused to answer | Record as responded. |
| *SLEEPHUNGRYFREQ* | How often did this happen? | 1  2  3  6 | Rarely (once or twice in the past 4 weeks)  Sometimes (three to ten times in the past 4 weeks)  Often (more than ten times in the past 4 weeks)  Refused to answer | Izi zinachitika mowirikiza bwanji? | Mwa apo ndi apo (Kamodzi kapena kawiri mu masabata anayi apitawa)  Nthawi zina (Kuchokera katatu kufikira ka teni mu masabata anayi apitawa)  Pafupi pafupi (Kuposera ka teni mu masabata anayi apitawa )  Refused to answer | Ivi vikachitikanga mwapafupi-pafupi uli? | Munyengo munyengo/mwa apo na apo (kamoza panji kawili mumasabata yanayi ayo yajumpha)  Nyengo zinyakhe (katatu mupaka kateni mumasabata yanayi ayo yajumpha)  Mwapafupipafupi (kujumpha ka teni pamasabata yanayi ayo yajumpha)  Refused to answer | If yes to above question, record as responded. |
| *NOFOOD24HR* | In the past 4 weeks, did you or any household member go a whole day and night without eating anything because there was not enough food? | 1  0  8  6 | Yes  No  Don't Know  Refused to answer | Pa masabata anayi omwe apitawa, alipo panyumba yanu pano, amene anaswera ndi kugona ndi njala chifukwa choti chakudya chinali chosakwanira? | Eya  Ayi  Sindikudziwa  Refused to answer | Pamasabata yanayi ayo yajumpha, kasi imwe panji munyake walipo munyumba yinu uyo wakatandala nakugona na njala zuwa lose chifukwa chakuti chakulya chikaba chambula kukwana? | Enya  Yayi  Nkhumanya yayi  Refused to answer | Record as responded. |
| *NOFOOD24HRFREQ* | How often did this happen? | 1  2  3  6 | Rarely (once or twice in the past 4 weeks)  Sometimes (three to ten times in the past 4 weeks)  Often (more than ten times in the past 4 weeks)  Refused to answer | Izi zinachitika mowirikiza bwanji? | Mwa apo ndi apo (Kamodzi kapena kawiri mu masabata anayi apitawa)  Nthawi zina (Kuchokera katatu kufikira ka teni mu masabata anayi apitawa)  Pafupi pafupi (Kuposera ka teni mu masabata anayi apitawa )  Refused to answer | Ivi vikachitikanga mwapafupi-pafupi uli? | Munyengo munyengo/mwa apo na apo (kamoza panji kawili mumasabata yanayi ayo yajumpha)  Nyengo zinyakhe (katatu mupaka kateni mumasabata yanayi ayo yajumpha)  Mwapafupipafupi (kujumpha ka teni pamasabata yanayi ayo yajumpha)  Refused to answer | If yes to above question, record as responded. |
| ***LIFEEVENTS*** | **LIFE EVENTS** |  |  | **LIFE EVENTS** |  | **LIFE EVENTS** |  |  |
| *LIFEEVENTS_INTRO* | Now I would like to ask you about whether any of the following difficult situations have happened to you in the last 12 months. |  |  | Pano ndikufuna ndikufunseni mafunso ngati zochitika zovuta zotsatirazi zinakuchitikiranipo m’miyezi khumi ndi iwiiri yapitayi. |  | Sono nimufumbeninge mafumbo yakukhwaskana nanyengo zinonono zakulondezga izi pala zikamuchitikiranipo mu miyezi 12 yajumpha iyi. |  | This section asks about life events that happened in the past 12 months. |
| *DIED12MONTH_NOTE* | Did any of the following people in your life die in the last 12 months? |  |  | Kodi anthu awa anamwalirapo mu moyo mwanu m’miyezi khumi ndi iwiri yapitayi? |  | Kasi wanthu awa watayikapo mu moyo winu mu miyezi 12 yajumpha iyi? |  | Select appropriate answers from options below. |
| *SPOUSEDIED* | Your spouse | 1  0  8  6 | Yes  No  Don't Know  Refused to answer | Amuna anu/akazi anu | Eya  Ayi  Sindikudziwa  Refused to answer | Mfumu/muwoli | Enya  Yayi  Nkhumanya yayi  Refused to answer | Record as responded. |
| *SPOUSEDIEDPREG* | Did this happen before you fell pregnant or since you fell pregnant? | 1  2  6 | Before pregnant  Since pregnant  Refused to answer | Kodi zimenezi zinachitika musanatenge mimba kapena kuchokera pamene munatenga mimba iyi? | Musanatenge mimba  Kuchokera pamene munatenga mimba iyi  Refused to answer | Kasi ivi vikachitika pambere mundapoke nthumbo iyi panji kufuma apo mukapoka nthumbo iyi? | Pambere mundapoke nthumbo  Kufuma apo mukapoka nthumbo iyi  Refused to answer | *For women only:* if yes to above question, record as responded. |
| *PARENTDIED* | Your own parent | 1  0  8  6 | Yes  No  Don't Know  Refused to answer | Kholo lanu | Eya  Ayi  Sindikudziwa  Refused to answer | Mpapi winu | Enya  Yayi  Nkhumanya yayi  Refused to answer | Record as responded. |
| *PARENTDIEDPREG* | Did this happen before you fell pregnant or since you fell pregnant? | 1  2  6 | Before pregnant  Since pregnant  Refused to answer | Kodi zimenezi zinachitika musanatenge mimba kapena kuchokera pamene munatenga mimba iyi? | Musanatenge mimba  Kuchokera pamene munatenga mimba iyi  Refused to answer | Kasi ivi vikachitika pambere mundapoke nthumbo iyi panji kufuma apo mukapoka nthumbo iyi? | Pambere mundapoke nthumbo  Kufuma apo mukapoka nthumbo iyi  Refused to answer | *For women only:* if yes to above question, record as responded. |
| *SIBLINGDIED* | Your real brother or sister | 1  0  8  6 | Yes  No  Don't Know  Refused to answer | Mchimwene kapena mchemwali wanu weniweni | Eya  Ayi  Sindikudziwa  Refused to answer | Mwana munyinu mwanalume / mwanakazi mwenechomwenecho | Enya  Yayi  Nkhumanya yayi  Refused to answer | Record as responded. |
| *SIBLINGDIEDPREG* | Did this happen before you fell pregnant or since you fell pregnant? | 1  2  6 | Before pregnant  Since pregnant  Refused to answer | Kodi zimenezi zinachitika musanatenge mimba kapena kuchokera pamene munatenga mimba iyi? | Musanatenge mimba  Kuchokera pamene munatenga mimba iyi  Refused to answer | Kasi ivi vikachitika pambere mundapoke nthumbo iyi panji kufuma apo mukapoka nthumbo iyi? | Pambere mundapoke nthumbo  Kufuma apo mukapoka nthumbo iyi  Refused to answer | *For women only:* if yes to above question, record as responded. |
| *CHILDDIED* | Your child | 1  0  8  6 | Yes  No  Don't Know  Refused to answer | Mwana wanu | Eya  Ayi  Sindikudziwa  Refused to answer | Mwana winu | Enya  Yayi  Nkhumanya yayi  Refused to answer | Record as responded. |
| *CHILDDIEDPREG* | Did this happen before you fell pregnant or since you fell pregnant? | 1  2  6 | Before pregnant  Since pregnant  Refused to answer | Kodi zimenezi zinachitika musanatenge mimba kapena kuchokera pamene munatenga mimba iyi? | Musanatenge mimba  Kuchokera pamene munatenga mimba iyi  Refused to answer | Kasi ivi vikachitika pambere mundapoke nthumbo iyi panji kufuma apo mukapoka nthumbo iyi? | Pambere mundapoke nthumbo  Kufuma apo mukapoka nthumbo iyi  Refused to answer | *For women only:* if yes to above question, record as responded. |
| *CLOSEFAMDIED* | Any other close family member | 1  0  8  6 | Yes  No  Don't Know  Refused to answer | Wachibale wapafupi wina aliyense | Eya  Ayi  Sindikudziwa  Refused to answer | Wachibale mwenechomwenecho waliyose | Enya  Yayi  Nkhumanya yayi  Refused to answer | Record as responded. |
| *CLOSEFAMDIEDPREG* | Did this happen before you fell pregnant or since you fell pregnant? | 1  2  6 | Before pregnant  Since pregnant  Refused to answer | Kodi zimenezi zinachitika musanatenge mimba kapena kuchokera pamene munatenga mimba iyi? | Musanatenge mimba  Kuchokera pamene munatenga mimba iyi  Refused to answer | Kasi ivi vikachitika pambere mundapoke nthumbo iyi panji kufuma apo mukapoka nthumbo iyi? | Pambere mundapoke nthumbo  Kufuma apo mukapoka nthumbo iyi  Refused to answer | *For women only:* if yes to above question, record as responded. |
| *CLOSEFRNDDIED* | Any close friend | 1  0  8  6 | Yes  No  Don't Know  Refused to answer | Mzanu wapafupi wina aliyense | Eya  Ayi  Sindikudziwa  Refused to answer | Mnyinu mwenechomwenecho waliyose | Enya  Yayi  Nkhumanya yayi  Refused to answer | Record as responded. |
| *CLOSEFRNDDIEDPREG* | Did this happen before you fell pregnant or since you fell pregnant? | 1  2  6 | Before pregnant  Since pregnant  Refused to answer | Kodi zimenezi zinachitika musanatenge mimba kapena kuchokera pamene munatenga mimba iyi? | Musanatenge mimba  Kuchokera pamene munatenga mimba iyi  Refused to answer | Kasi ivi vikachitika pambere mundapoke nthumbo iyi panji kufuma apo mukapoka nthumbo iyi? | Pambere mundapoke nthumbo  Kufuma apo mukapoka nthumbo iyi  Refused to answer | *For women only:* if yes to above question, record as responded. |
| *ILLINJ* | In the past 12 months, did you have any serious illness or have you been seriously injured? | 1  0  8  6 | Yes  No  Don't Know  Refused to answer | M’miyezi khumi ndi iwiri yapitayi munakhalapo ndi matenda aakulu/oopsya kapena munavulalapo kwambiri? | Eya  Ayi  Sindikudziwa  Refused to answer | Mmiyezi 12 yajumpa iyi, mulikukhalapo na matenda yakulu/yakofya panji kupwetekeka chomene? | Enya  Yayi  Nkhumanya yayi  Refused to answer | Record as responded. |
| *ILLINJPREG* | Did this happen before you fell pregnant or since you fell pregnant? | 1  2  3  6 | Before pregnant  Since pregnant  Both  Refused to answer | Kodi zimenezi zinachitika musanatenge mimba kapena kuchokera pamene munatenga mimba iyi? | Musanatenge mimba  Kuchokera pamene munatenga mimba iyi  Zonse ziwiri  Refused to answer | Kasi ivi vikachitika pambere mundapoke nthumbo iyi panji kufuma apo mukapoka nthumbo iyi? | Pambere mundapoke nthumbo  Kufuma apo mukapoka nthumbo iyi  Vyose vibiri  Refused to answer | *For women only:* if yes to above question, record as responded. |
| *FAMILLINJ* | In the past 12 months, have any close family members had a serious illness or injury? | 1  0  8  6 | Yes  No  Don't Know  Refused to answer | M’miyezi khumi ndi iwiri yapitayi, achibale apafupi, alipo anakhala ndi nthenda yayikulu/yowopsya kapena kuchita ngozi yayikulu? | Eya  Ayi  Sindikudziwa  Refused to answer | Mmiyezi 12 yajumpa iyi, bachibale benechobenecho, balipo awo bakabapo na nthenda yikulu/yakofya panji kuchita ngozi yikulu? | Enya  Yayi  Nkhumanya yayi  Refused to answer | Record as responded. |
| *FAMILLINJPREG* | Did this happen before you fell pregnant or since you fell pregnant? | 1  2  3  6 | Before pregnant  Since pregnant  Both  Refused to answer | Kodi zimenezi zinachitika musanatenge mimba kapena kuchokera pamene munatenga mimba iyi? | Musanatenge mimba  Kuchokera pamene munatenga mimba iyi  Zonse ziwiri  Refused to answer | Kasi ivi vikachitika pambere mundapoke nthumbo iyi panji kufuma apo mukapoka nthumbo iyi? | Pambere mundapoke nthumbo  Kufuma apo mukapoka nthumbo iyi  Vyose vibiri  Refused to answer | *For women only:* if yes to above question, record as responded. |
| *ATTCKASSLT* | In the past 12 months, were you physically attacked or assaulted or had your life been threatened? | 1  0  8  6 | Yes  No  Don't Know  Refused to answer | M’miyezi khumi ndi iwiri yapitayi, kodi munachitidwapo nkhanza kapena kuvurazidwa kapena moyo wanu unali pa chiopsezo? | Eya  Ayi  Sindikudziwa  Refused to answer | Mmiyezi 12 yajumpa iyi, kasi muli kupwetekeka panji kuchitilikapo nkhaza panjiso umoyo winu kofyeka? | Enya  Yayi  Nkhumanya yayi  Refused to answer | Record as responded. |
| *ATTCKASSLTPREG* | Did this happen before you fell pregnant or since you fell pregnant? | 1  2  3  6 | Before pregnant  Since pregnant  Both  Refused to answer | Kodi zimenezi zinachitika musanatenge mimba kapena kuchokera pamene munatenga mimba iyi? | Musanatenge mimba  Kuchokera pamene munatenga mimba iyi  Zonse ziwiri  Refused to answer | Kasi ivi vikachitika pambere mundapoke nthumbo iyi panji kufuma apo mukapoka nthumbo iyi? | Pambere mundapoke nthumbo  Kufuma apo mukapoka nthumbo iyi  Vyose vibiri  Refused to answer | *For women only:* if yes to above question, record as responded. |
| *ROBBED* | In the past 12 months, were you robbed or was your home burglarized? | 1  0  8  6 | Yes  No  Don't Know  Refused to answer | M’miyezi khumi ndi iwiri yapitayi, kodi munaberedwa kapena nyumba yanu inathyoledwa? | Eya  Ayi  Sindikudziwa  Refused to answer | Mmiyezi 12 yajumpa iyi, kasi mulikwibilikapo panji nyumba yinu yikibilikapo? | Enya  Yayi  Nkhumanya yayi  Refused to answer | Record as responded. |
| *ROBBEDPREG* | Did this happen before you fell pregnant or since you fell pregnant? | 1  2  3  6 | Before pregnant  Since pregnant  Both  Refused to answer | Kodi zimenezi zinachitika musanatenge mimba kapena kuchokera pamene munatenga mimba iyi? | Musanatenge mimba  Kuchokera pamene munatenga mimba iyi  Zonse ziwiri  Refused to answer | Kasi ivi vikachitika pambere mundapoke nthumbo iyi panji kufuma apo mukapoka nthumbo iyi? | Pambere mundapoke nthumbo  Kufuma apo mukapoka nthumbo iyi  Vyose vibiri  Refused to answer | *For women only:* if yes to above question, record as responded. |
| *UNSAFENGHBHD* | In the past 12 months, have you felt unsafe in your neighborhood? | 1  0  8  6 | Yes  No  Don't Know  Refused to answer | M’miyezi khumi ndi iwiri yapitayi, kodi munazimva kuti ndinu wosatetezedwa ku dera kwanu? | Eya  Ayi  Sindikudziwa  Refused to answer | Mmiyezi 12 yajumpha iyi, kasi mulikujipulikapo kuti muli ŵambula kuvikilirika mu uzengezgani winu? | Enya  Yayi  Nkhumanya yayi  Refused to answer | Record as responded. |
| *UNSAFENGHBHDPREG* | Did this happen before you fell pregnant or since you fell pregnant? | 1  2  3  6 | Before pregnant  Since pregnant  Both  Refused to answer | Kodi zimenezi zinachitika musanatenge mimba kapena kuchokera pamene munatenga mimba iyi? | Musanatenge mimba  Kuchokera pamene munatenga mimba iyi  Zonse ziwiri  Refused to answer | Kasi ivi vikachitika pambere mundapoke nthumbo iyi panji kufuma apo mukapoka nthumbo iyi? | Pambere mundapoke nthumbo  Kufuma apo mukapoka nthumbo iyi  Vyose vibiri  Refused to answer | *For women only:* if yes to above question, record as responded. |
| *PROBRELATSHP* | In the past 12 months, have you had problems in your relationship with your spouse? | 1  0  8  6 | Yes  No  Don't Know  Refused to answer | M’miyezi khumi ndi iwiri yapitayi, kodi mnakhalapo ndi mabvuto amubanja apakati pa inu ndi amuna anu/akazi anu? | Eya  Ayi  Sindikudziwa  Refused to answer | Mmiyezi 12 yajumpha iyi, kasi mukabapo na masuzgo yamunthengwa pakati pa imwe na wawoli/bafumu binu? | Enya  Yayi  Nkhumanya yayi  Refused to answer | Record as responded. |
| *PROBRELATSHPPREG* | Did this happen before you fell pregnant or since you fell pregnant? | 1  2  3  6 | Before pregnant  Since pregnant  Both  Refused to answer | Kodi zimenezi zinachitika musanatenge mimba kapena kuchokera pamene munatenga mimba iyi? | Musanatenge mimba  Kuchokera pamene munatenga mimba iyi  Zonse ziwiri  Refused to answer | Kasi ivi vikachitika pambere mundapoke nthumbo iyi panji kufuma apo mukapoka nthumbo iyi? | Pambere mundapoke nthumbo  Kufuma apo mukapoka nthumbo iyi  Vyose vibiri  Refused to answer | *For women only:* if yes to above question, record as responded. |
| *SEPDIVORCD* | In the past 12 months, have you become separated or divorced? | 1  0  8  6 | Yes  No  Don't Know  Refused to answer | M’miyezi khumi ndi iwiri yapitayi, kodi munasudzulidwa kapena banja kutha? | Eya  Ayi  Sindikudziwa  Refused to answer | Mmiyezi 12 yajumpha iyi, kasi nthengwa yinu yili Kumalapo panji kupatukana? | Enya  Yayi  Nkhumanya yayi  Refused to answer | Record as responded. |
| *SEPDIVORCDPREG* | Did this happen before you fell pregnant or since you fell pregnant? | 1  2  6 | Before pregnant  Since pregnant  Refused to answer | Kodi zimenezi zinachitika musanatenge mimba kapena kuchokera pamene munatenga mimba iyi? | Musanatenge mimba  Kuchokera pamene munatenga mimba iyi  Refused to answer | Kasi ivi vikachitika pambere mundapoke nthumbo iyi panji kufuma apo mukapoka nthumbo iyi? | Pambere mundapoke nthumbo  Kufuma apo mukapoka nthumbo iyi  Refused to answer | *For women only:* if yes to above question, record as responded. |
| *LE_WORKDIFF_INTRO* | The following questions are about significant work-related difficulties. |  |  | Mafunso otsatirawa ndiokhudzana ndi zovuta zowonekera zokhudzana ndi kuntchito. |  | Mafumbo yakulondezgapo aya, yakukhwaskana na maunonono yakukhwaskana na ntchito |  | Read note exactly as written. |
| *FINDWORK* | In the past 12 months, have you been unable to find work even though you were looking for it? | 1  0  8  6 | Yes  No  Don't Know  Refused to answer | M’miyezi khumi ndi iwiri yapitayi, Mwavutikapo ndi kusapeza ntchito ngakhale mumayifunafuna? | Eya  Ayi  Sindikudziwa  Refused to answer | Mmiyezi 12 yajumpha iyi, kasi mwasuzikapo nakutondeka kusanga ntchito nangauli mukayipenjanga? | Enya  Yayi  Nkhumanya yayi  Refused to answer | Record as responded. |
| *FINDWORKPREG* | Did this happen before you fell pregnant or since you fell pregnant? | 1  2  3  6 | Before pregnant  Since pregnant  Both  Refused to answer | Kodi zimenezi zinachitika musanatenge mimba kapena kuchokera pamene munatenga mimba iyi? | Musanatenge mimba  Kuchokera pamene munatenga mimba iyi  Zonse ziwiri  Refused to answer | Kasi ivi vikachitika pambere mundapoke nthumbo iyi panji kufuma apo mukapoka nthumbo iyi? | Pambere mundapoke nthumbo  Kufuma apo mukapoka nthumbo iyi  Vyose vibiri  Refused to answer | *For women only:* if yes to above question, record as responded. |
| *LOSTWORKINC* | In the past 12 months, have you lost your work or source of income, or been in danger of losing your work or source of income? | 1  0  8  6 | Yes  No  Don't Know  Refused to answer | M’miyezi khumi ndi iwiri yapitayi, Kodi munataya ntchito yanu kapena zinthu zimene zimakupezetsani ndalama, kapenanso kukhala pa chiopsezo chotaya ntchito kapena zinthu zimene zimakupezetsani ndalama? | Eya  Ayi  Sindikudziwa  Refused to answer | Mmiyezi 12 yajumpha iyi, Kasi mukataya ntchito panji icho mukasangilangapo ndalama, panji kuŵa pa chofyo cha kutaya ntchito panji icho mukusangilapo ndalama? | Enya  Yayi  Nkhumanya yayi  Refused to answer | Record as responded. |
| *LOSTWORKINCPREG* | Did this happen before you fell pregnant or since you fell pregnant? | 1  2  3  6 | Before pregnant  Since pregnant  Both  Refused to answer | Kodi zimenezi zinachitika musanatenge mimba kapena kuchokera pamene munatenga mimba iyi? | Musanatenge mimba  Kuchokera pamene munatenga mimba iyi  Zonse ziwiri  Refused to answer | Kasi ivi vikachitika pambere mundapoke nthumbo iyi panji kufuma apo mukapoka nthumbo iyi? | Pambere mundapoke nthumbo  Kufuma apo mukapoka nthumbo iyi  Vyose vibiri  Refused to answer | *For women only:* if yes to above question, record as responded. |
| *PROBEMPLOY* | In the past 12 months, have you had trouble with your employer such as being suspended or demoted, experiencing discrimination, or any other major problems with your job? | 1  0  8  6 | Yes  No  Don't Know  Refused to answer | M’miyezi khumi ndi iwiri yapitayi, kodi munakhalapo ndi vuto ndi wokulembani ntchito monga kuimitsidwa pa ntchito kapena kutsitsidwa, kusalidwa kapena mavuto ena aakulu wokhudza ntchito yanu? | Eya  Ayi  Sindikudziwa  Refused to answer | Mmiyezi 12 yajumpha iyi, Kasi mukaŵapo nasuzgo na uyo walikumulembani ntchito ngati kwimikika ntchito panji kukhizgika udindo, kusalika, panji suzgo lirilose likulu na ntchito yinu? | Enya  Yayi  Nkhumanya yayi  Refused to answer | Record as responded. |
| *PROBEMPLOYPREG* | Did this happen before you fell pregnant or since you fell pregnant? | 1  2  3  6 | Before pregnant  Since pregnant  Both  Refused to answer | Kodi zimenezi zinachitika musanatenge mimba kapena kuchokera pamene munatenga mimba iyi? | Musanatenge mimba  Kuchokera pamene munatenga mimba iyi  Zonse ziwiri  Refused to answer | Kasi ivi vikachitika pambere mundapoke nthumbo iyi panji kufuma apo mukapoka nthumbo iyi? | Pambere mundapoke nthumbo  Kufuma apo mukapoka nthumbo iyi  Vyose vibiri  Refused to answer | *For women only:* if yes to above question, record as responded. |
| *DEBT* | In the past 12 months, have you or your immediate family who live with you been in debt? | 1  0  8  6 | Yes  No  Don't Know  Refused to answer | M’miyezi khumi ndi iwiri yapitayi, kodi inu kapena wachibale wapafupi amene mumakhala naye anali mungongole? | Eya  Ayi  Sindikudziwa  Refused to answer | Mmiyezi 12 yajumpha iyi, kasi imwe panji mubale winu mwenechomwenecho uyo mukukhala nayo wakawapo nangongole? | Enya  Yayi  Nkhumanya yayi  Refused to answer | Record as responded. |
| *DEBTPREG* | Did this happen before you fell pregnant or since you fell pregnant? | 1  2  3  6 | Before pregnant  Since pregnant  Both  Refused to answer | Kodi zimenezi zinachitika musanatenge mimba kapena kuchokera pamene munatenga mimba iyi? | Musanatenge mimba  Kuchokera pamene munatenga mimba iyi  Zonse ziwiri  Refused to answer | Kasi ivi vikachitika pambere mundapoke nthumbo iyi panji kufuma apo mukapoka nthumbo iyi? | Pambere mundapoke nthumbo  Kufuma apo mukapoka nthumbo iyi  Vyose vibiri  Refused to answer | *For women only:* if yes to above question, record as responded. |
| ***NOTE_1*** | **Coercive control and IPV (FOR WOMEN ONLY)** |  |  | **Coercive control and IPV (FOR WOMEN ONLY)** |  | **Coercive control and IPV (FOR WOMEN ONLY)** |  |  |
| ***NOTE_2*** | **READ TO THE PARTICIPANT: The next questions ask about things that are done to many women by their partner(s). We will use the tablet computer that asks the questions via headphones so that nobody can hear, including me (the fieldworker). The answers you give will be completely confidential.** |  |  | **READ TO THE PARTICIPANT: Mafunso otsatirawa akufunsa za zinthu zomwe zimawachitikira azimayi ochuluka kuchokera kwa amuna awo kapena chibwenzi chawo. Tigwilitsa ntchito kompyuta ya pa manja yomwe yizikufunsani mafunso ndipo inuyo muzimvetsera kuzera ma hedifoni kuti pasakhale munthu wina aliyense kuphatikizapo ineyo yemwe azimvetsera nawo.Mayankho omwe mutapereke akhala achinsinsi mwantheradi.** |  | **READ TO THE PARTICIPANT: Mafumbo yakulondezgapo yakufumba za vinthu ivyo vyaŵachitikilapo ŵanakazi ŵanandi kufuma kuŵafumu ŵawo panji chibwezi. Titigwiriskilenge ntchito kompyuta yamumawoko iyo yimufumbaninge mafumbo ndipo imwe mutiyapulikizgenge kwizila muma hedifoni mwakuti pawavyenge ine panji waliyose wakupulikako mafumbo aya. Mazgolo ayo mupelekenge yawenge ya chisisi mwakufikapo.** |  | **Read out loud to the participant as written in the note. Show participant how to hold tablet and use headsets. Assure participants of confidentiality, particularly if unsure after reading the note.** |
| *PRACTICE_1* | *1st Practice Questions* |  |  | *1st Practice Questions* |  | *1st Practice Questions* |  | *These practise questions are for interviewer to go through with the participant.* |
| ***NOTE_3*** | **READ TO THE PARTICIPANT: This tablet computer reads out each question and you then use the shapes to give the answer that applies to you. Firstly, here are examples of the shapes that you will see and what answer they mean. When you have given your answer press the NEXT button at the bottom of the screen to move to the next question.** |  |  | **READ TO THE PARTICIPANT: Kompyuta ya tabuleti iyi izikuwerengerani mafunso ndipo inu mugwiritsa ntchito zojambula popereka mayankho oyenera kwa inuyo. Choyamba, izi ndizitsanzo za zojambula zomwe muzione komanso ndi matanthauzo amayankho ake. Mukapereka yankho dinani batani la NEXT pamunsipa kuti mupite pa funso lotsatira.** |  | **READ TO THE PARTICIPANT: Kompyuta ya tabuleti iyi yimubelengeraninge mafumbo ndipo imwe mugwiliskenge ntchito vyakujambula pakupeleka mazgolo ya kwenelera kwa imwe. Chakwamba, ivi niviyezgelero vya vyakujambula ivyo muviwonenge kweniso na ving’anamulo vya mazgolo yakhe. Para mwazgola dofyani batani la NEXT pasika apa kuti mulute pa fumbo linyakhe.** |  | Read note exactly as written. Show the participant the NEXT button and let them press the button to move on to the next question. The tablet will then start reading out the meanings of the shape. PLEASE DO NOT USE THE HEADSET FOR PRACTISE QUESTIONS. |
| *NOTE_TICK* | This is a tick. It means YES. |  |  | Ichi ndichichongi. Chikutanthauza kubvomeleza. |  | Ichi ntchichongero. Chikung’anamula kuzomerezga. |  | Work together with participants and show the shape. |
| *NOTE_CROSS* | This is a cross. It means NO. |  |  | Ichi ndi ekesi. Akutanthauza kukana. |  | Ichi ntchikomero. Chikung’anamula kukana. |  | Work together with participants and show the shape. |
| *NOTE_CIRCLE* | This is a circle. It means ONCE |  |  | Iyi ndi seko (chozungilira). Ikutanthauza kamodzi |  | Iyi ndi seko (chizingizgo). Ikung’anamula kamoza |  | Work together with participants and show the shape. |
| *NOTE_TRIANGLE* | This is a triangle. It means A FEW TIMES |  |  | Iyi ndi thirayango (chamakona atatu). Ikutanthauza mochulukirapo. |  | Iyi ndi thirayango (chamakona yatatu). Ikung’anumula kanandiko. |  | Work together with participants and show the shape. |
| *NOTE_SQUARE* | This is a square. It means MANY TIMES |  |  | Ili ndi bokosi (sikweya). Likutanthauza kambiri. |  | Ili ndi bokosi (sikweya). Likung’amula kanandi |  | Work together with participants and show the shape. |
| ***NOTE_4*** | **Let’s practice pressing the buttons.** |  |  | **Tiyeni tiyeselere kudina mabatani.** |  | **Tiyeni tiyezgelere kudofya mabatani.** |  | **This lets the participant practise pressing the tablet. Work together with participants, but let them press the buttons on the computer.** |
| *SELECT_ONE_A* | Press tick. |  |  | Dinani chichongi. |  | Dofyani chichongero. |  | Let participant press shape on the tablet. |
| *SELECT_ONE_B* | Press cross. |  |  | Dinani ekesi. |  | Dofyani chikomero. |  | Let participant press shape on the tablet. |
| *SELECT_ONE_C* | Press circle. |  |  | Dinani seko (chozungilira). |  | Dofyani seko (chizingizgo). |  | Let participant press shape on the tablet. |
| *SELECT_ONE_D* | Press triangle. |  |  | Dinani thirayango (chamakona atatu). |  | Dofyani thirayango (chamakona yatatu). |  | Let participant press shape on the tablet |
| *SELECT_ONE_E* | Press square. |  |  | Dinani bokosi (sikweya). |  | Dofyani bokosi (sikweya). |  | Let participant press shape on the tablet |
| *PRACTICE_2* | *2nd Practice Questions* |  |  | *2nd Practice Questions* |  | *2nd Practice Questions* |  |  |
| ***NOTE_5*** | **Now we will practice using the buttons to answer some questions. This time you will use the headphones. The following questions are just practice questions to show you how this tablet computer works and to help you learn how to answer the questions. When you have given your answer press the NEXT button at the bottom of the screen to move to the next question.** |  |  | **Panopa tiyeselera kuyankha mafunso podina mabatani. Tsopano mugwiritsa ntchito ma hedifoni. Mafunso osatirawa ndiongoyeselelapo kuti tikuonetseni momwe kompyuta ya tabuleti iyi ikugwilira ntchito kuti tikuthandizeni momwe mungayankhire mafunso. Mukapeleka mayankho anu dinani batani la NEXT pamunsipa kuti mupite ku funso lotsatira.** |  | **Pasono tiyezgelerenge kuzgola mafumbo pakudofya mabatani. Sono mugwiriskenge ntchito ma hedifoni. Mafumbo yakulondezga aya ngakuyezgelapo waka kuti timuwoneskani umo kompyuta ya tabuleti iyi yikugwilira ntchito kuti timovwirani umo mungazgolera mafumbo. Para mwapeleka mazgolo yinu dofyani batani la NEXT pasika kuti mulute pa fumbo la kulondezgapo.** |  | Read the note and instructions exactly as written. Use headphones for this practise question. Tell participant you will go through the practice questions together until s/he is comfortable doing it by him/herself. |
| *EATEN_NSIMA* | Have you ever eaten nsima? If YES press the tick; if NO press the cross. | 1  0 | Yes  No | Kodi munadyapo nsima? Ngati EYA dinani chichongi; ngati AYI dinani ekesi | Eya  Ayi | Kasi mulikulyapo sima? Para ENYA dofyani chichongero; para YAYI dofyani chikomero. | Enya  Yayi | Confirm the answer with the participant. Acknowledge when the participant has selected the correct answer and go through the shapes again if not. |
| *HAPPEN_NSIMA* | Has this happened in the past 3 months? If YES press the tick; if NO press the cross. | 1  0 | Yes  No | Kodi izi zachitika m’miyezi itatu yapitayi? Ngati EYA dinani chichongi; ngati AYI dinani ekesi | Eya  Ayi | Kasi ivi vikachitika m’miyezi yitatu yajumpha iyi? Para ENYA dofyani chichongero; para YAYI dofyani chikomero. | Enya  Yayi | Confirm the answer with the participant. Acknowledge when the participant has selected the correct answer and go through the shapes again if not. |
| *FREQUENT_NSIMA* | In the past 3 months, how often has this happened? If once, press Circle; if a few times, press Triangle; if many times, press Square. | 1  2  3 | Once  Few Times  Many Times | Mu miyezi itatu yapitayi, munganene kuti zimenezi zinachitika mowirikiza bwanji? Ngati ndi kamodzi, dinani seko (chozungilira); ngati ndi mochulukirapo, thirayango (chamakona atatu); ngati ndi kambiri, dinani bokosi (sikweya). | Kamodzi  Mochulukirapo  Kambiri | Mu miyezi itatu yajumpha, kasi mungayowoya kuti ivi vikachitika kanandi wuli? Pala nkhamoza dofyani seko (chizingizgo); pala nikanandiko dofyani thirayango (chamakona yatatu); pala nikanandi dofyani bokosi (sikweya). | Kamoza  Kanandiko  Kanandi | Confirm the answer with the participant. Acknowledge when the participant has selected the correct answer and go through the shapes again if not. |
| ***NOTE_6*** | **That is the end of the practice questions. Please tell the interviewer you have finished.** |  |  | **Apa ndimamatsiriziro pa mafunso oyesezera. Chonde muwuzeni ofunsa mafunso kuti mwamaliza.** |  | **Apa mphaumalilo pa mafumbo yakuyezelerapo. Chonde muphalilani wakufumba mafumbo kuti mwamala.** |  | **Let the participant know that this is the end of the practise questions.** |
| ***NOTE_7*** | **READ TO THE PARTICIPANT: Are you happy to continue to the main questions? The answers you give will be completely confidential and you will now use headphones so that nobody can hear, including me (the fieldworker).** |  |  | **READ TO THE PARTICIPANT: Kodi mungakondwe kuti tipitilize ndimafunsowa? Mayankho omwe mutapeleke akhala achinsisi mwantheradi komanso inuyo muzimvetsera mafunso kuzera ma hedifoni kuti pasakhale munthu wina aliyense kuphatikizapo ineyo yemwe azimvetsera nawo.** |  | **READ TO THE PARTICIPANT: Kasi mungakondwa kuti tilutilizge kumufumbani mafumbo agha? Mazgolo ayo mupelekenge ya benge ya chinsisi mwakufikapo ndipo mafumbo mutiyapulikizgenge kwizila muma hedifoni mwakuti pawavyenge ine panji waliyose wakupulikako mafumbo aya.** |  | Read exactly as written. Remind participants that this is confidential. Do not distract the participant. Observe if there is any trouble with using the tablet. |
| ***NOTE_8*** | **COERSIVE CONTROL** |  |  | **COERSIVE CONTROL** |  | **COERSIVE CONTROL** |  |  |
| *ACASI_COERSIVE_CONTROL* | ACASI - COERSIVE CONTROL |  |  | ACASI - COERSIVE CONTROL |  | ACASI - COERSIVE CONTROL |  |  |
| *CC1* | Does your spouse try to stop you from seeing your friends? If YES press the tick; if NO press the cross | 1  0 | Yes  No | Kodi chibwenzi chanu kapena amuna anu amayesera kukuletsani kuonana ndi anzanu? Ngati Eya ndinani chichongi; ngati Ayi ndinani ekesi. | Eya  Ayi | Kasi chibwezi chinu panji bafumu binu bakuyezga kumukanizgani kuwonana na wanyinu? Pala Enya ndofyani chichongelo; pala Yayi ndofyani chikomelo. | Enya  Yayi | For participant to respond. |
| *CC2* | Does your spouse try to stop you contacting your family of birth? If YES press the tick; if NO press the cross | 1  0 | Yes  No | Kodi chibwenzi chanu kapena amuna anu amayesera kukuletsani kulumikizana ndi apabanja anu enieni kapena chibale chamagazi? Ngati Eya ndinani chichongi; ngati Ayi ndinani ekesi. | Eya  Ayi | Kasi chibwezi chinu panji bafumu binu bakuyezga kumukanizgani kubapamoza na bakubanja linu uko mukababikila? Pala Enya ndofyani chichongelo; pala Yayi ndofyani chikomelo. | Enya  Yayi | For participant to respond. |
| *CC3* | Does your spouse insist on knowing where you are all the time? If YES press the tick; if NO press the cross | 1  0 | Yes  No | Kodi chibwenzi chanu kapena amuna anu amakakamira kudziwa komwe muli nthawi ndi nthawi? Ngati Eya ndinani chichongi; ngati Ayi ndinani ekesi. | Eya  Ayi | Kasi chibwezi chinu panji bafumu binu bakumuchichizgani kuti bamanye uko muli nyengo zose? Pala Enya ndofyani chichongelo; pala Yayi ndofyani chikomelo. | Enya  Yayi | For participant to respond. |
| *CC4* | Does your spouse ignore you and treat you indifferently? If YES press the tick; if NO press the cross | 1  0 | Yes  No | Kodi chibwenzi chanu kapena amuna anu amakhala osakulabadirani kapena kusaonetsa chidwi nanu? Ngati Eya ndinani chichongi; ngati Ayi ndinani ekesi. | Eya  Ayi | Kasi chibwezi chinu panji bafumu binu bakumusulaninge panji kuleka kuwoneska chitemwa kwa imwe? Pala Enya ndofyani chichongelo; pala Yayi ndofyani chikomelo. | Enya  Yayi | For participant to respond. |
| *CC5* | Does your spouse get angry if you speak to another man? If YES press the tick; if NO press the cross | 1  0 | Yes  No | Kodi chibwenzi chanu kapena amuna anu amakwiya mukamayankhula ndi mzibambo wina? Ngati Eya ndinani chichongi; ngati Ayi ndinani ekesi. | Eya  Ayi | Kasi chibwezi chinu panji bafumu binu bakukwiya pala mukuyowoyeskana na mwanalume munyakhe? Pala Enya ndofyani chichongelo; pala Yayi ndofyani chikomelo. | Enya  Yayi | For participant to respond. |
| *CC6* | Is your spouse often suspicious that you are unfaithful? If YES press the tick; if NO press the cross | 1  0 | Yes  No | Kodi chibwenzi chanu kapena amuna anu amakukayikirani kuti ndinu osakhulupilirika pa chibwenzi kapena pa banja? Ngati Eya ndinani chichongi; ngati Ayi ndinani ekesi. | Eya  Ayi | Kasi chibwezi chinu panji bafumu binu bakumukayikilani kuti ndimwe bambula kugomwezgeka pa chibwezi panji pa nthengwa? Pala Enya ndofyani chichongelo; pala Yayi ndofyani chikomelo. | Enya  Yayi | For participant to respond. |
| *CC7* | Does your spouse expect you to ask his permission before seeking health care for yourself? If YES press the tick; if NO press the cross | 1  0 | Yes  No | Kodi chibwenzi chanu kapena amuna anu amayembekezera kuwapempha chilorezo chofuna thandizo la zaumoyo?Ngati Eya ndinani chichongi; ngati Ayi ndinani ekesi. | Eya  Ayi | Kasi chibwezi chinu panji bafumu binu bakukhumba mupemphe dankha chizomelezgo chawo pala mukukhumba wovwiri wa vyawumoyo? Pala Enya ndofyani chichongelo; pala Yayi ndofyani chikomelo. | Enya  Yayi | For participant to respond. |
| *ACASI_IPV* | ACASI - IPV |  |  | ACASI - IPV |  | ACASI - IPV |  |  |
| ***NOTE_9*** | **I want you to tell me if your current husband/partner, or any other partner has done the following things to you.** |  |  | **Ndikufuna mundidziwitse ngati amuna anu kapena omwe muli nawo pachibwenzi anakuchitaniponi zinthu zotsatirazi.** |  | **Nkhukhumba muniphalile pala bafumu winu panji uyo muli nayo pachibwezi wakamuchitilanipo vinthu ivi.** |  |  |
| *IPV1* | Has your current husband/partner, or any other partner, ever insulted you or made you feel bad about yourself? If YES press Tick; if NO press Cross. | 1  0 | Yes  No | Kodi amuna anu kapena omwe muli nawo pachibwenzi anakunyozanipo kapena kukupangitsani kusamva bwino za inu mwini? Ngati ndi EYA, dinani chichongi; ngati ndi AYI dinani ekesi. | Eya  Ayi | Kasi bafumu winu panji awo muli nawo pachibwezi wali kumunenanipo panji kumupangiskani kujipulika uheni vya imwe mwawene? Pala ENYA dofyani chichongero; pala YAYI dofyani chikomero. | Enya  Yayi | For participant to respond. |
| *IPV1A* | Has this happened in the past 3 months? If YES press Tick; if NO press Cross. | 1  0 | Yes  No | Kodi izi zinachitikapo miyezi itatu yapitayi? Ngati ndi EYA, dinani chichongi; ngati ndi AYI dinani ekesi. | Eya  Ayi | Kasi ivi vilikuchitikapo mu miyezi yitatu yajumpha iyi? Pala ENYA dofyani chichongero; pala YAYI dofyani chikomero. | Enya  Yayi | For participant to respond. |
| *IPV1B* | In the past 3 months, how often has this happened? If once, press Circle; if a few times, press Triangle; if many times, press Square. | 1  2  3 | Once  Few Times  Many Times | [Mu miyezi itatu yapitayi, munganene kuti zimenezi zinachitika mowirikiza bwanji? Ngati ndi kamodzi, dinani seko (chozungilira); ngati ndi mochulukirapo, thirayango (chamakona atatu); ngati ndi kambiri, dinani bokosi (sikweya). | Kamodzi  Mochulukirapo  Kambiri | Mu miyezi itatu yajumpha, kasi mungayowoya kuti ivi vikachitika kanandi wuli? Pala nkhamoza dofyani seko (chizingizgo); pala nikanandiko dofyani thirayango (chamakona yatatu); pala nikanandi dofyani bokosi (sikweya). | Kamoza  Kanandiko  Kanandi | For participant to respond. |
| *IPV2* | Has your current husband/partner, or any other partner ever belittled or humiliated you in front of other people? If YES press Tick; if NO press Cross. | 1  0 | Yes  No | Kodi amuna anu kapena omwe muli nawo pachibwenzi anakupeputsanipo kapena kukuchititsani manyazi pa maso pa anthu ena? Ngati ndi EYA, dinani chichongi; ngati ndi AYI dinani ekesi. | Eya  Ayi | Kasi bafumu winu panji awo muli nawo pachibwezi wali kumupepuskanipo panji kumuyuyulanipo imwe pamaso pa banthu? Pala ENYA dofyani chichongero; pala YAYI dofyani chikomero. | Enya  Yayi | For participant to respond. |
| *IPV2A* | Has this happened in the past 3 months? If YES press Tick; if NO press Cross. | 1  0 | Yes  No | Kodi izi zinachitikapo miyezi itatu yapitayi? Ngati ndi EYA, dinani chichongi; ngati ndi ayi dinani ekesi. | Eya  Ayi | Kasi ivi vilikuchitikapo mu miyezi yitatu yajumpha iyi? Pala ENYA dofyani chichongero; pala YAYI dofyani chikomero. | Enya  Yayi | For participant to respond. |
| *IPV2B* | In the past 3 months, how often has this happened? If once, press Circle; if a few times, press Triangle; if many times, press Square. | 1  2  3 | Once  Few Times  Many Times | Mu miyezi itatu yapitayi, munganene kuti zimenezi zinachitika mowirikiza bwanji? Ngati ndi kamodzi, dinani seko (chozungilira); ngati ndi mochulukirapo, thirayango (chamakona atatu); ngati ndi kambiri, dinani bokosi (sikweya). | Kamodzi  Mochulukirapo  Kambiri | Mu miyezi yitatu iyo ghajumpha, kasi mungayowoya kuti ivi vikachitika kanandi wuli? Pala nkhamoza dofyani seko (chizingizgo); pala nikanandiko dofyani thirayango (chamakona yatatu); pala nikanandi dofyani bokosi (sikweya). | Kamoza  Kanandiko  Kanandi | For participant to respond. |
| *IPV3* | Has your current husband/partner, or any other partner ever done things to scare or intimidate you on purpose (e.g. by the way he looked at you, by yelling and smashing things)? If YES press Tick; if NO press Cross. | 1  0 | Yes  No | Kodi amuna anu kapena omwe muli nawo pachibwenzi, anachita zinthu zokuchititsani mantha kapena kukuopsyezani (monga momwe anakuyang’anirani, kukalipa, ndikuphwanya zinthu) mwadaladala? Ngati ndi eya, dinani chichongi; ngati ndi ayi dinani ekesi. | Eya  Ayi | Kasi bafumu winu panji awo muli nawo pachibwezi wali kumuofyanipo ( ngeti kumubekani mwawukali, panji kuswa vinthu) mwadala waka? Pala ENYA dofyani chichongero; pala YAYI dofyani chikomero. | Enya  Yayi | For participant to respond. |
| *IPV3A* | Has this happened in the past 3 months? If YES press Tick; if NO press Cross. | 1  0 | Yes  No | Kodi izi zinachitikapo miyezi itatu yapitayi? Ngati ndi EYA, dinani chichongi; ngati ndi AYI dinani ekesi. | Eya  Ayi | Kasi ivi vilikuchitikapo mu miyezi yitatu yajumpha iyi? Pala ENYA dofyani chichongero; pala YAYI dofyani chikomero. | Enya  Yayi | For participant to respond. |
| *IPV3B* | In the past 3 months, how often has this happened? If once, press Circle; if a few times, press Triangle; if many times, press Square. | 1  2  3 | Once  Few Times  Many Times | Mu miyezi itatu yapitayi, munganene kuti zimenezi zinachitika mowirikiza bwanji? Ngati ndi kamodzi, dinani seko (chozungilira); ngati ndi mochulukirapo, thirayango (chamakona atatu); ngati ndi kambiri, dinani bokosi (sikweya). | Kamodzi  Mochulukirapo  Kambiri | Mu miyezi itatu yajumpha, kasi mungayowoya kuti ivi vikachitika kanandi wuli? Pala nkhamoza dofyani seko (chizingizgo); pala nikanandiko dofyani thirayango (chamakona yatatu); pala nikanandi dofyani bokosi (sikweya). | Kamoza  Kanandiko  Kanandi | For participant to respond. |
| *IPV4* | Has your current husband/partner, or any other partner ever threatened to hurt you or someone you care about? If YES press Tick; if NO press Cross. | 1  0 | Yes  No | Kodi amuna anu kapena omwe muli nawo pachibwenzi anakuopsyezanipo kuti akupwetekani inuyo kapena munthu wina wapamtima panu? Ngati ndi EYA, dinani chichongi; ngati ndi AYI dinani ekesi. | Eya  Ayi | Kasi bafumu winu panji awo muli nawo pachibwezi wali kumuwofyanipo kuti wamupwetekeninge panji kupweteka munthu uyo mukumutemwa? Pala ENYA dofyani chichongero; pala YAYI dofyani chikomero. | Enya  Yayi | For participant to respond. |
| *IPV4A* | Has this happened in the past 3 months? If YES press Tick; if NO press Cross. | 1  0 | Yes  No | Kodi izi zinachitikapo miyezi itatu yapitayi? Ngati ndi EYA, dinani chichongi; ngati ndi AYI dinani ekesi. | Eya  Ayi | Kasi ivi vilikuchitikapo mu miyezi yitatu yajumpha iyi? Pala ENYA dofyani chichongero; pala YAYI dofyani chikomero. | Enya  Yayi | For participant to respond. |
| *IPV4B* | In the past 3 months, how often has this happened? If once, press Circle; if a few times, press Triangle; if many times, press Square. | 1  2  3 | Once  Few Times  Many Times | Mu miyezi itatu yapitayi, munganene kuti zimenezi zinachitika mowirikiza bwanji? Ngati ndi kamodzi, dinani seko (chozungilira); ngati ndi mochulukirapo, thirayango (chamakona atatu); ngati ndi kambiri, dinani bokosi (sikweya). | Kamodzi  Mochulukirapo  Kambiri | Mu miyezi yitatu yajumpha, kasi mungayowoya kuti ivi vikachitika kanandi wuli? Pala nkhamoza dofyani seko (chizingizgo); pala nikanandiko dofyani thirayango (chamakona yatatu); pala nikanandi dofyani bokosi (sikweya). | Kamoza  Kanandiko  Kanandi | For participant to respond. |
| *IPV5* | Has your current husband/partner, or any other partner ever slapped you or thrown something at you that could hurt you? If YES press Tick; if NO press Cross. | 1  0 | Yes  No | Kodi amuna anu kapena omwe muli nawo pachibwenzi anakumenyani mbama kapena kukugendani? Ngati ndi eya, dinani chichongi; ngati ndi ayi dinani ekesi. | Eya  Ayi | Kasi bafumu winu panji awo muli nawo pachibwezi wali kumutimbanipo khofi panji kumupozani? Pala ENYA dofyani chichongero; pala YAYI dofyani chikomero. | Enya  Yayi | For participant to respond. |
| *IPV5A* | Has this happened in the past 3 months? If YES press Tick; if NO press Cross. | 1  0 | Yes  No | Kodi izi zinachitikapo miyezi itatu yapitayi? Ngati ndi EYA, dinani chichongi; ngati ndi AYI dinani ekesi. | Eya  Ayi | Kasi ivi vilikuchitikapo mu miyezi yitatu yajumpha iyi? Pala ENYA dofyani chichongero; pala YAYI dofyani chikomero. | Enya  Yayi | For participant to respond. |
| *IPV5B* | In the past 3 months, how often has this happened? If once, press Circle; if a few times, press Triangle; if many times, press Square. | 1  2  3 | Once  Few Times  Many Times | Mu miyezi itatu yapitayi, munganene kuti zimenezi zinachitika mowirikiza bwanji? Ngati ndi kamodzi, dinani seko (chozungilira); ngati ndi mochulukirapo, thirayango (chamakona atatu); ngati ndi kambiri, dinani bokosi (sikweya) | Kamodzi  Mochulukirapo  Kambiri | Mu miyezi yitatu yajumpha, kasi mungayowoya kuti ivi vikachitika kanandi wuli? Pala nkhamoza dofyani seko (chizingizgo); pala nikanandiko dofyani thirayango (chamakona yatatu); pala nikanandi dofyani bokosi (sikweya). | Kamoza  Kanandiko  Kanandi | For participant to respond. |
| *IPV6* | Has your current husband/partner, or any other partner ever pushed you or shoved you? If YES press Tick; if NO press Cross. | 1  0 | Yes  No | Kodi amuna anu kapena omwe muli nawo pachibwenzi anakukankhanipo? Ngati ndi eya, dinani chichongi; ngati ndi ayi dinani ekesi. | Eya  Ayi | Kasi bafumu winu panji awo muli nawo pachibwezi wali kumukankhanipo  ? Pala ENYA dofyani chichongero; pala YAYI dofyani chikomero. | Enya  Yayi | For participant to respond. |
| *IPV6A* | Has this happened in the past 3 months? If YES press Tick; if NO press Cross. | 1  0 | Yes  No | Kodi izi zinachitikapo miyezi itatu yapitayi? Ngati ndi EYA, dinani chichongi; ngati ndi AYI dinani ekesi. | Eya  Ayi | Kasi ivi vilikuchitikapo mu miyezi yitatu yajumpha iyi? Pala ENYA dofyani chichongero; pala YAYI dofyani chikomero. | Enya  Yayi | For participant to respond. |
| *IPV6B* | In the past 3 months, how often has this happened? If once, press Circle; if a few times, press Triangle; if many times, press Square. | 1  2  3 | Once  Few Times  Many Times | Mu miyezi itatu yapitayi, munganene kuti zimenezi zinachitika mowirikiza bwanji? Ngati ndi kamodzi, dinani seko (chozungilira); ngati ndi mochulukirapo, thirayango (chamakona atatu); ngati ndi kambiri, dinani bokosi (sikweya). | Kamodzi  Mochulukirapo  Kambiri | Mu miyezi yitatu yajumpha, kasi mungayowoya kuti ivi vikachitika kanandi wuli? Pala nkhamoza dofyani seko (chizingizgo); pala nikanandiko dofyani thirayango (chamakona yatatu); pala nikanandi dofyani bokosi (sikweya). | Kamoza  Kanandiko  Kanandi | For participant to respond. |
| *IPV7* | Has your current husband/partner, or any other partner ever hit you with his fist or with something else that could hurt you? If YES press Tick; if NO press Cross. | 1  0 | Yes  No | Kodi amuna anu kapena omwe muli nawo pachibwenzi anakumenyanipo ndi chibakera kapena chida choti chinakatha kukupwetekani? Ngati ndi EYA, dinani chichongi; ngati ndi AYI dinani ekesi. | Eya  Ayi | Kasi bafumu winu panji awo muli nawo pachibwezi wali kumutimbanipo na nkhonyo panji chinthu chinyakhe chakuti chingamuvulazani? Pala ENYA dofyani chichongero; pala YAYI dofyani chikomero. | Enya  Yayi | For participant to respond. |
| *IPV7A* | Has this happened in the past 3 months? If YES press Tick; if NO press Cross. | 1  0 | Yes  No | Kodi izi zinachitikapo miyezi itatu yapitayi? Ngati ndi EYA, dinani chichongi; ngati ndi AYI dinani ekesi. | Eya  Ayi | Kasi ivi vilikuchitikapo mu miyezi yitatu yajumpha iyi? Pala ENYA dofyani chichongero; pala YAYI dofyani chikomero. | Enya  Yayi | For participant to respond. |
| *IPV7B* | In the past 3 months, how often has this happened? If once, press Circle; if a few times, press Triangle; if many times, press Square. | 1  2  3 | Once  Few Times  Many Times | Mu miyezi itatu yapitayi, munganene kuti zimenezi zinachitika mowirikiza bwanji? Ngati ndi kamodzi, dinani seko (chozungilira); ngati ndi mochulukirapo, thirayango (chamakona atatu); ngati ndi kambiri, dinani bokosi (sikweya). | Kamodzi  Mochulukirapo  Kambiri | Mu miyezi yitatu yajumpha, kasi mungayowoya kuti ivi vikachitika kanandi wuli?  Pala nkhamoza dofyani seko (chizingizgo); pala nikanandiko dofyani thirayango (chamakona yatatu); pala nikanandi dofyani bokosi (sikweya). | Kamoza  Kanandiko  Kanandi | For participant to respond. |
| *IPV8* | Has your current husband/partner, or any other partner ever kicked you, dragged you or beaten you up? If YES press Tick; if NO press Cross. | 1  0 | Yes  No | Kodi amuna anu kapena omwe muli nawo pachibwenzi anakupatsanipo mateche kapena kukukokani kapena kukumenyani? Ngati ndi EYA, dinani chichongi; ngati ndi ayi dinani ekesi. | Eya  Ayi | Kasi bafumu winu panji awo muli nawo pachibwezi bali kumutimbanipo na bakela, panji kumukwekwetani, panji kumutimbani? Pala ENYA dofyani chichongero; pala YAYI dofyani chikomero. | Enya  Yayi | For participant to respond. |
| *IPV8A* | Has this happened in the past 3 months? If YES press Tick; if NO press Cross | 1  0 | Yes  No | Kodi izi zinachitikapo miyezi itatu yapitayi? Ngati ndi eya, dinani chichongi; ngati ndi ayi dinani ekesi. | Eya  Ayi | Kasi ivi vilikuchitikapo mu miyezi yitatu yajumpha iyi? Pala ENYA dofyani chichongero; pala YAYI dofyani chikomero. | Enya  Yayi | For participant to respond. |
| *IPV8B* | In the past 3 months, how often has this happened? If once, press Circle; if a few times, press Triangle; if many times, press Square. | 1  2  3 | Once  Few Times  Many Times | Mu miyezi itatu yapitayi, munganene kuti zimenezi zinachitika mowirikiza bwanji? Ngati ndi kamodzi, dinani seko (chozungilira); ngati ndi mochulukirapo, thirayango (chamakona atatu); ngati ndi kambiri, dinani bokosi (sikweya). | Kamodzi  Mochulukirapo  Kambiri | Mu miyezi yitatu yajumpha, kasi mungayowoya kuti ivi vikachitika kanandi wuli? Pala nkhamoza dofyani seko (chizingizgo); pala nikanandiko dofyani thirayango (chamakona yatatu); pala nikanandi dofyani bokosi (sikweya). | Kamoza  Kanandiko  Kanandi | For participant to respond. |
| *IPV9* | Has your current husband/partner, or any other partner ever choked or burnt you on purpose? If YES press Tick; if NO press Cross. | 1  0 | Yes  No | Kodi amuna anu kapena omwe muli nawo pachibwenzi anakufinyani pakhosi kapena kukuotchani mwadaladala? Ngati ndi eya, dinani chichongi; ngati ndi ayi dinani ekesi. | Eya  Ayi | Kasi bafumu winu panji awo muli nawo pachibwezi wali kumufyenyanipo pasingo panji kumotchani mwadala waka? Pala ENYA dofyani chichongero; pala YAYI dofyani chikomero. | Enya  Yayi | For participant to respond. |
| *IPV9A* | Has this happened in the past 3 months? If YES press Tick; if NO press Cross. | 1  0 | Yes  No | Kodi izi zinachitikapo miyezi itatu yapitayi? Ngati ndi EYA, dinani chichongi; ngati ndi AYI dinani ekesi. | Eya  Ayi | Kasi ivi vilikuchitikapo mu miyezi yitatu yajumpha iyi? Pala ENYA dofyani chichongero; pala YAYI dofyani chikomero. | Enya  Yayi | For participant to respond. |
| *IPV9B* | In the past 3 months, how often has this happened? If once, press Circle; if a few times, press Triangle; if many times, press Square. | 1  2  3 | Once  Few Times  Many Times | Mu miyezi itatu yapitayi, munganene kuti zimenezi zinachitika mowirikiza bwanji? Ngati ndi kamodzi, dinani seko (chozungilira); ngati ndi mochulukirapo, thirayango (chamakona atatu); ngati ndi kambiri, dinani bokosi (sikweya). | Kamodzi  Mochulukirapo  Kambiri | Mu miyezi yitatu yajumpha, kasi mungayowoya kuti ivi vikachitika kanandi wuli? Pala nkhamoza dofyani seko (chizingizgo); pala nikanandiko dofyani thirayango (chamakona yatatu); pala nikanandi dofyani bokosi (sikweya). | Kamoza  Kanandiko  Kanandi | For participant to respond. |
| *IPV10* | Has your current husband/partner, or any other partner ever threatened to use or actually used a gun or other weapon against you? If YES press Tick; if NO press Cross. | 1  0 | Yes  No | Kodi amuna anu kapena omwe muli nawo pachibwenzi anakuopsyezanipo ndi mfuti kapena kugwiritsa ntchito mfuti kapena chida china pa inu? Ngati ndi EYA, dinani chichongi; ngati ndi AYI dinani ekesi. | Eya  Ayi | Kasi bafumu winu panji awo muli nawo pachibwezi wali kumuofyanipo na futi panji kugwiriska ntchito futi na chida chinyakhe pa imwe? Pala ENYA dofyani chichongero; pala YAYI dofyani chikomero. | Enya  Yayi | For participant to respond. |
| *IPV10A* | Has this happened in the past 3 months? If YES press Tick; if NO press Cross. | 1  0 | Yes  No | Kodi izi zinachitikapo miyezi itatu yapitayi? Ngati ndi EYA, dinani chichongi; ngati ndi AYI dinani ekesi. | Eya  Ayi | Kasi ivi vilikuchitikapo mu miyezi yitatu yajumpha iyi? Pala ENYA dofyani chichongero; pala YAYI dofyani chikomero. | Enya  Yayi | For participant to respond. |
| *IPV10B* | In the past 3 months, how often has this happened? If once, press Circle; if a few times, press Triangle; if many times, press Square. | 1  2  3 | Once  Few Times  Many Times | Mu miyezi itatu yapitayi, munganene kuti zimenezi zinachitika mowirikiza bwanji? Ngati ndi kamodzi, dinani seko (chozungilira); ngati ndi mochulukirapo dinanithirayango (chamakona atatu), ngati ndi kambiri, dinani bokosi (sikweya). | Kamodzi  Mochulukirapo  Kambiri | Mu miyezi yitatu _yajumpha, kasi mungayowoya kuti ivi vikachitika kanandi wuli? Pala nkhamoza dofyani seko (chizingizgo); pala nikanandiko dofyani thirayango (chamakona yatatu); pala nikanandi dofyani bokosi (sikweya). | Kamoza  Kanandiko  Kanandi | For participant to respond. |
| *IPV11* | Has your current husband/partner, or any other partner ever physically forced you to have sexual intercourse when you did not want to? If YES press Tick; if NO press Cross. | 1  0 | Yes  No | Kodi amuna anu kapena omwe muli nawo pachibwenzi anakukakamizanipo kuchita zogonana pomwe inu musakufuna? Ngati ndi eya, dinani chichongi; ngati ndi ayi dinani ekesi. | Eya  Ayi | Kasi bafumu winu panji awo muli nawo pachibwezi wali kumuchichizganipo kuti mugonane nawo imwe mundakhumbe? Pala ENYA dofyani chichongero; pala YAYI dofyani chikomero. | Enya  Yayi | For participant to respond. |
| *IPV11A* | Has this happened in the past 3 months? If YES press Tick; if NO press Cross. | 1  0 | Yes  No | Kodi izi zinachitikapo miyezi itatu yapitayi? Ngati ndi EYA, dinani chichongi; ngati ndi AYI dinani ekesi. | Eya  Ayi | Kasi ivi vilikuchitikapo mu miyezi yitatu yajumpha iyi? Pala ENYA dofyani chichongero; pala YAYI dofyani chikomero. | Enya  Yayi | For participant to respond. |
| *IPV11B* | In the past 3 months, how often has this happened? If once, press Circle; if a few times, press Triangle; if many times, press Square. | 1  2  3 | Once  Few Times  Many Times | Mu miyezi itatu yapitayi, munganene kuti zimenezi zinachitika mowirikiza bwanji? Ngati ndi kamodzi, dinani seko (chozungilira); ngati ndi mochulukirapo, thirayango (chamakona atatu); ngati ndi kambiri, dinani bokosi (sikweya). | Kamodzi  Mochulukirapo  Kambiri | Mu miyezi yitatu yajumpha, kasi mungayowoya kuti ivi vikachitika kanandi wuli? Pala nkhamoza dofyani seko (chizingizgo); pala nikanandiko dofyani thirayango (chamakona yatatu); pala nikanandi dofyani bokosi (sikweya). | Kamoza  Kanandiko  Kanandi | For participant to respond. |
| *IPV12* | Did you ever have sexual intercourse you did not want because you were afraid of what your current husband/partner, or any other partner might do? If YES press Tick; if NO press Cross. | 1  0 | Yes  No | Kodi munachitapo zogonana musakufuna chifukwa chowopa zomwe amuna anu kapena omwe muli nawo pachibwenzi angakupangeni mukakana? Ngati ndi EYA, dinani chichongi; ngati ndi AYI dinani ekesi. | Eya  Ayi | Kasi muli kugonanapo kwambula kukhumba chifukwa cha wofyi wa ivyo bafumu winu panji awo muli nawo pachibwezi wangakuchitani usange mwakana? Pala ENYA dofyani chichongero; pala YAYI dofyani chikomero. | Enya  Yayi | For participant to respond. |
| *IPV12A* | Has this happened in the past 3 months? If YES press Tick; if NO press Cross. | 1  0 | Yes  No | Kodi izi zinachitikapo miyezi itatu yapitayi? Ngati ndi EYA, dinani chichongi; ngati ndi AYI dinani ekesi. | Eya  Ayi | Kasi ivi vilikuchitikapo mu miyezi yitatu yajumpha iyi? Pala ENYA dofyani chichongero; pala YAYI dofyani chikomero. | Enya  Yayi | For participant to respond. |
| *IPV12B* | In the past 3 months, how often has this happened? If once, press Circle; if a few times, press Triangle; if many times, press Square. | 1  2  3 | Once  Few Times  Many Times | Mu miyezi itatu yapitayi, munganene kuti zimenezi zinachitika mowirikiza bwanji? Ngati ndi kamodzi, dinaniseko (chozungilira); ngati ndi mochulukirapo, thirayango (chamakona atatu); ngati ndi kambiri, dinani bokosi (sikweya). | Kamodzi  Mochulukirapo  Kambiri | Mu miyezi yitatu yajumpha, kasi mungayowoya kuti ivi vikachitika kanandi wuli? Pala nkhamoza dofyani seko (chizingizgo); pala nikanandiko dofyani thirayango (chamakona yatatu); pala nikanandi dofyani bokosi (sikweya). | Kamoza  Kanandiko  Kanandi | For participant to respond. |
| *IPV13* | Did your current husband/partner, or any other partner ever force you to do something sexual that you found degrading or humiliating? If YES press Tick; if NO press Cross. | 1  0 | Yes  No | Kodi amuna anu kapena omwe muli nawo pachibwenzi anakukakamizanipo kuchita zinthu zina zokhudza kugonana zomwe mudazipeza kuti ndi zochotsa umunthu wanu kapena zochititsa manyazi? Ngati ndi eya, dinani chichongi; ngati ndi ayi dinani ekesi. | Eya  Ayi | Kasi bafumu winu panji awo muli nawo pachibwezi bakamuchichizganipo kuchita vinthu vinyakhe vyakukhwaskana nakugonana ivyo mukasanga kuti vikawa vyakumukhozgani soni panji kumuyuyulani? Pala ENYA dofyani chichongero; pala YAYI dofyani chikomero. | Enya  Yayi | For participant to respond. |
| *IPV13A* | Has this happened in the past 3 months? If YES press Tick; if NO press Cross. | 1  0 | Yes  No | Kodi izi zinachitikapo miyezi itatu yapitayi? Ngati ndi EYA, dinani chichongi; ngati ndi AYI dinani ekesi. | Eya  Ayi | Kasi ivi vilikuchitikapo mu miyezi yitatu yajumpha iyi? Pala ENYA dofyani chichongero; pala YAYI dofyani chikomero. | Enya  Yayi | For participant to respond. |
| *IPV13B* | In the past 3 months, how often has this happened? If once, press Circle; if a few times, press Triangle; if many times, press Square. | 1  2  3 | Once  Few Times  Many Times | Mu miyezi itatu yapitayi, munganene kuti zimenezi zinachitika mowirikiza bwanji? Ngati ndi kamodzi, dinani seko (chozungilira); ngati ndi mochulukirapo thirayango (chamakona atatu); ngati ndi kambiri, dinani bokosi (sikweya). | Kamodzi  Mochulukirapo  Kambiri | Mu miyezi yitatu yajumpha, kasi mungayowoya kuti ivi vikachitika kanandi wuli? Pala nkhamoza dofyani seko (chizingizgo); pala nikanandiko dofyani thirayango (chamakona yatatu); pala nikanandi dofyani bokosi (sikweya). | Kamoza  Kanandiko  Kanandi | For participant to respond. |
| ***NOTE_10*** | **Non-spousal abuse** |  |  | **Non-spousal abuse** |  | **Non-spousal abuse** |  |  |
| *NONSPABUSE* | In the last 3 months, have you been physically beaten, sexually abused or mistreated in any way by someone other than your spouse? If YES press Tick; if NO press Cross. | 1  0 | Yes  No | Mu miyezi itatu yapitayi, munayamba mwamenyedwapo, kuchitiridwa nkhanza zogonana, kapena kuchitiridwa nkhanza munjira ina ndi munthu wina osati mamuna wanu? Ngati ndi EYA, dinani chichongi; ngati ndi AYI dinani ekesi. | Eya  Ayi | Mu miyezi yitatu iyo yajumpha, muli kutimbikapo, kuchitilika nkhaza zakugonana, panji nkhaza munthowa yiliyose na munthu munyakhe kupatula mfumu winu? Pala ENYA dofyani chichongero; pala YAYI dofyani chikomero. | Enya  Yayi | For participant to respond. |
| *NONSPABUSEHOME* | Does that person live with you in your home? If YES press Tick; if NO press Cross. | 1  0 | Yes  No | Kodi munthu ameneyu mumakhala naye munyumba imodzi? Ngati ndi EYA, dinani chichongi; ngati ndi AYI dinani ekesi. | Eya  Ayi | Kasi munthu uyu mukukhala nayo munyumba yimoza? Pala ENYA dofyani chichongero; pala YAYI dofyani chikomero. | Enya  Yayi | For participant to respond. |
| *ACASI_IPV* |  |  |  |  |  |  |  |  |
| ***NOTE_11*** | **That is the end of this section. Please tell the interviewer you have finished** |  |  | **Apa ndi pamatsiriziro pa mafunso a gawo ili. Chonde muuzeni ofunsa mafunso kuti mwamaliza** |  | **Apa mphaumaliro pa mafumbo ya chigawa ichi. Chonde, muphalilani wakufumba mafumbo kuti mwamalizga.** |  |  |
| ***NOTE_12*** | **READ TO THE PARTICIPANT:  As I said, I do not know what answers you have given. However, if you are experiencing, or has experienced, violence or abuse, I can offer advice on how to get help. Do you want me to tell you about this help?  If no: If you change your mind, you can come to CRH/Area 25 and ask to speak to MEIRU counsellor.  If yes: If you feel you are at severe risk today and you wish to go somewhere safe right now, I can arrange for you to come to CRH/Area 25 H.C. to talk to someone who can help you. Do you want to do that? [If yes]: ACTION [If no] If you want to get help but not today, I can arrange an appointment for you to speak to a MEIRU counsellor. Do you want to do that?  [If yes]: ACTION Do you want me to give I can give you a leaflet with advice and phone numbers you can call? Will you be able to keep this leaflet secret? If yes: Give leaflet and give advice to keep it safe/secret. [If no to any]: If you change your mind, come to CRH/Area 25 and ask to speak to MEIRU counsellor or do you want me to give you a phone number that you can call. If YES: ACTION; give number.  If NO: ACTION; If you change your mind, come to CRH/Area 25 and ask to speak to MEIRU counsellor.** |  |  | **READ TO THE PARTICIPANT:  Monga m’mene ndanenera kale, sindikudziwa mayankho omwe mwapereka. Komabe, ngati mukuchitiridwa nkhanza kapena zinakuchitikiraniponi ndintha kukupasani langizo lakomwe mungapeze thandizo.. Kodi mukufuna kuti ndikuwuzeni za thandizo limeneri?  [IF NO]: ngati mwasintha maganizo anu mutha kubwela ku chipatala ku Area 25 ndikuzapempha kuti mulankhulane ndi mphungu wa MEIRU.  [IF YES]: ngati mukuzimva kuti muli pachiopsezo chachikulu kwa lero ndipo mukulakalaka mutapita kwina kwake koti mukapeze thandizo, nditha kukupangilani ndondomeko kuti mubwere ku chipatala ku Area 25 kuti muzalankhulane ndi munthu yemwe angakuthandizeni. Mukufuna zitakhala mwachoncho? [IF YES]: ACTION; [IF NO]: ngati mukufunabe thandizo koma osati kwalero, nditha kukonza ndondomeko kuti muzalankhule ndi mphungu wa MEIRU. Mukufuna zitakhala mwachoncho? [IF YES]:ACTION; Nditha kukupatsani chipepala chomwe chili ndi malangizo komanso manambala a foni omwe mutha kuyimbilako, kodi mukufuna ndikupatseni? [IF YES]: pelekani chipepala ndi malangizo akuti achisunge bwinobwino komanso mwachinsinsi. [If NO to any]: ngati mwasintha maganizo anu mutha kubwela ku chipatala ku Area 25 ndi kuzafunsa kuti mulankhulane ndi mphunguwa MEIRU kapena mukufuna ndikupaseni nambala ya foni yomwe mungayimbe.  If YES: ACTION; perekani nambala ya foni.  If NO: ACTION; ngati mwasintha maganizo anu mutha kubwela ku chipatala ku Area 25 ndi kuzafunsa kuti mulankhulane ndi mphunguwa MEIRU** |  | **READ TO THE PARTICIPANT:  Nga umo ndanguyowoyela, nkhumanya chala mazgolo ayo mwazgola. Nangawuli, pala mukuchitilika panji mulikuchitilikapo nkhaza, ndingamupasani malangizo ya umo mungasangila wovwili. Kasi mukukhumba kuti ndimuphalirani za wovwili uwu?  [IF NO]: pala mwasintha maghanoghano yinu, mungiza ku chipatala cha ku Chilumba ndipo muzafumbe kuti muyowoyeskane na mulangizi wa MEIRU.  [IFYES]: pala mukujipulika kuti muli pachofyo chikulu muhanyauno ndipo mukukhumba kuluta kuwunyakhe uko mungakasanga wovwili, ine ndingamunozgelani ndondomeko yakuti mwize ku chipatala cha ku Chilumba kuti muzakayowoyeskane na mulangizi. Mukukhumba kuti viŵe mwantheula? [PIF YES]: ACTION [IF NO]: pala mukuwukhumba wovwili kweni kwamuhanyauno chala, ndingamunozgelani ndondomeko kuti muzakayowoyeskane na mulangizi wa ku MEIRU. Mukukhumba kuti viŵe mwantheula? [IF YES]: ACTION Ndingamupani chipepala icho chinamalangizo na manambala ya foni yakuti mungayimbilako, kasi mukukhumba kuti ndimupasani? [IF YES]: pelekani chipepala, ndakuŵalangiza kuti wachisunge makola kweniso mwachsisi. [IF NO TO ANY]: pala mwasintha maghanoghano yinu, mungiza ku chipatala cha ku Chilumba ndipo muzakafumbe kuti muyowoyeskane na mulangizi wa MEIRU panyakhe nimupasani nambala ya foni yoti muzakayimbe.  If YES: ACTION; pelekani nambala ya foni.  If NO: ACTION; pala mwasintha maghanoghano yinu, mungiza ku chipatala cha ku Chilumba ndipo muzakafumbe kuti muyowoyeskane na mulangizi wa MEIRU.** |  | **The participant will at this point let you know they are done. Read the note exactly as written.**  **The note also includes what actions to take depending on the participant’s responses.** |
| *CONTCODE* | Enter 4-digit code to continue |  |  | Enter 4-digit code to continue |  | Enter 4-digit code to continue |  | The tablet will now ask for your 4 digit code to continue into the next session. |
| ***GBPAA*** | **BLOOD PRESSURE & ANTHROPOMETRY** |  |  | **BLOOD PRESSURE & ANTHROPOMETRY** |  | **BLOOD PRESSURE & ANTHROPOMETRY** |  | **This session records BP and anthropometric measurements by the interviewer. Refer to “GM BP and anthropometry SOP” for specific guidelines on procedures.** |
| *BPRARM* | Blood pressure taken on right arm? | 1  0 | Yes  No | Blood pressure taken on right arm? | Eya  Ayi | Blood pressure taken on right arm? | Enya  Yayi | Record as appropriate.  Note: BP to be taken on the right hand unless otherwise, e.g. handicap participant. |
| *BPNOT* | If no, specify reason |  |  | If no, specify reason |  | If no, specify reason |  | Specify why BP taken on the left. |
| *BPLARM* | Blood pressure taken on left arm? | 1  0 | Yes  No | Blood pressure taken on left arm? | Eya  Ayi | Blood pressure taken on left arm? | Enya  Yayi | This question is asked when question above is “no”. |
| *BPLEFTNOT* | If no, specify reason |  |  | If no, specify reason |  | If no, specify reason |  | ? |
| *CIRARM* | BP cuff size [cm] | 1  2  3 | <24cm  24-32cm  33-41cm | BP cuff size [cm] | <24cm  24-32cm  33-41cm | BP cuff size [cm] | <24cm  24-32cm  33-41cm | Record appropriate cuff used based on arm circumference measurement. |
| *BPDEID* | Device ID for blood pressure |  |  | Device ID for blood pressure |  | Device ID for blood pressure |  | Record the standard device ID used in GM. |
| *S_16.5_A* | First measure |  |  | First measure |  | First measure |  | Enter first measurements. BP measurements are done three times after every 5 minutes.  Note: keep participant calm and inactive between the measurements. |
| *SYSBPF* | A. Systolic BP [brachial] |  |  | A. Systolic BP [brachial] |  | A. Systolic BP [brachial] |  |  |
| *DIABPF* | B. Diastolic BP [brachial] |  |  | B. Diastolic BP [brachial] |  | B. Diastolic BP [brachial] |  |  |
| *PRATEF* | C. Pulse rate |  |  | C. Pulse rate |  | C. Pulse rate |  |  |
| *S_16.5_B* | Second measure |  |  | Second measure |  | Second measure |  | Enter the second measurements (5 mins after first one). |
| *SYSBPS* | A. Systolic BP [brachial] |  |  | A. Systolic BP [brachial] |  | A. Systolic BP [brachial] |  |  |
| *DIABPS* | B. Diastolic BP [brachial] |  |  | B. Diastolic BP [brachial] |  | B. Diastolic BP [brachial] |  |  |
| *PRATES* | C. Pulse rate |  |  | C. Pulse rate |  | C. Pulse rate |  |  |
| *S_16.5_C* | Third measure |  |  | Third measure |  | Third measure |  | Enter the third measurement (5 minutes after second one). |
| *SYSBPT* | A. Systolic BP [brachial] |  |  | A. Systolic BP [brachial] |  | A. Systolic BP [brachial] |  |  |
| *DIABPT* | B. Diastolic BP [brachial] |  |  | B. Diastolic BP [brachial] |  | B. Diastolic BP [brachial] |  |  |
| *SYSMEAN* |  |  |  |  |  |  |  | The tablet will give you a systolic mean after third measurement and entry. |
| *DIAMEAN* |  |  |  |  |  |  |  | The tablet will give you a diastolic mean after third measurement and entry. |
| *S_16.8_I* | *Blood Pressure Referral* |  |  | *Blood Pressure Referral* |  | *Blood Pressure Referral* |  | *The tablet will give you a note on the measured BP interpretations.*  *If SBP <140 and/or DBP<90, the participants BP is normal.*  *If SBP 140-179 and/or DBP 90-109, the participants BP is* ***HIGH.***  *If SBP ≥180 and/or DBP ≥110, the participants BP is* ***SEVERE OR VERY HIGH.*** |
| *REBPNOTE4* | On medication for High BP |  |  | On medication for High BP |  | On medication for High BP |  | This will show up if participant is on medication for high BP. |
| *REFBP* | Referral slip given for high blood pressure assessment? | 1  0 | Yes  No | Referral slip given for high blood pressure assessment? | Eya  Ayi | Referral slip given for high blood pressure assessment? | Enya  Yayi | Record as appropriate for participants with an abnormal BP. |
| *DEVICEIDH* | Device ID for height |  |  | Device ID for height |  | Device ID for height |  | Enter device used for height measurement (must contain 3 digits and a letter) |
| *S_16.8_B* | Standing Height |  |  | Standing Height |  | Standing Height |  |  |
| *HEIGF* | B. Standing height [no shoes] -- Accuracy to 1 mm [0.1cm]: First measure |  |  | B. Muyezo watalika atayima [wopanda nsapato] -- Accuracy to 1 mm [0.1cm]: Mulingo woyamba |  | B. Standing Height [no shoes] -- Utali kwambula syapato 1 mm [0.1cm]: Kupima kwa kwamba |  | Enter standing first height. |
| *HEIGS* | B. Standing height [no shoes] -- Accuracy to 1 mm [0.1cm]: Second measure |  |  | B. Muyezo watalika atayima [wopanda nsapato] -- Accuracy to 1 mm [0.1cm]: Mulingo wachiwiri |  | B. Standing Height [no shoes] -- Utali kwambula syapato 1 mm [0.1cm]: Kupima kwa chibili |  | Enter second standing height. |
| *HEIGHTDIFF* | Height difference display |  |  | Height difference display |  | Height difference display |  | The prompt you to retake the height if there is a difference of >5% between the two entered heights. |
| *DEVICEIDW* | Device ID for weight |  |  | Device ID for weight |  | Device ID for weight |  | Enter device used for weight measurement (must contain 3 digits and a letter) |
| *S_16.8_A* | Weight |  |  | Weight |  | Weight |  |  |
| *WEIGF* | A. Weight [no shoes] kg -- Accuracy to 0.1 kg [100gm]: First measure |  |  | A. Weight [no shoes] kg -- Accuracy to 0.1 kg [100gm]: Mulingo woyamba |  | A. Weight [no shoes] kg --Uzito (kwambula syapato) 01.kg (100g): Kupima kwa kwamba |  | Enter first weight. |
| *WEIGS* | A. Weight [no shoes] kg -- Accuracy to 0.1 kg [100gm]: Second measure |  |  | A. Weight [no shoes] kg -- Accuracy to 0.1 kg [100gm]:Mulingo wachiwiri |  | A. Weight [no shoes] kg --Uzito (kwambula syapato) 01.kg (100g): Kupima kwa chibili |  | Enter second weight. |
| *WEIGHTDIFF* | Weight difference display |  |  | Weight difference display |  | Weight difference display |  | The prompt you to retake the weight if there is a difference of >5% between the two entered weights. |
| *BMIDISPLAY* | BMI |  |  | BMI |  | BMI |  | Auto calculated from weight and height and shown on tablet display. |
| *BPBMI* | Blood pressure and Body Mass Index (BMI) recorded in Health Passport? | 1  0 | Yes  No | Mwalemba kuthamanga kwa magazi (blood presure) ndi Body Mass Index (BMI) mu Health Passport? | Eya  Ayi | Kasi matenda ya kutchimbila ndopa (BP) na Body Mass Index (BMI) yalembeka mu Health Passport? | Enya  Yayi | Record results in health passport. |
| *S_16.8_C* | Body composition first measurements |  |  | Body composition first measurements |  | Body composition first measurements |  | Enter body mass composition measurements as shown in Body Composition Monitor display. See “GM BLOOD PRESSURE AND ANTHROPOMETRY STANDARD OPERATING PROCEDURE” on how to measure body mass composition. |
| *PBFF* | Percent body fat % : First measure |  |  | Percent body fat %: Mulingo woyamba |  | Percent body fat %: Kupima kwa kwamba |  | Enter body fat percentage. |
| *SMPF* | Skeletal Muscle % : First measure |  |  | Skeletal Muscle % : Mulingo woyamba |  | Skeletal Muscle % : Kupima kwa kwamba |  | Enter skeletal mass percentage. |
| *RMF* | Resting metabolism (kcal) : First measure |  |  | Resting metabolism (kcal) : Mulingo woyamba |  | Resting metabolism (kcal) : Kupima kwa kwamba |  | Resting metabolism (kcal). |
| *S_16.8_C5* | Body composition second measurements |  |  | Body composition second measurements |  | Body composition second measurements |  | Enter second measurements for body fat composition. The tablet will prompt you to re-enter any of the body composition difference is >5%. |
| *PBFS* | Body fat %: Second measure |  |  | Percent body fat %: Mulingo wachiwiri |  | Percent body fat %: Kupima kwa chibili |  | Enter second measurements for body fat composition. |
| *SMPS* | Skeletal Muscle % : Second measure |  |  | Skeletal Muscle % : Mulingo wachiwiri |  | Skeletal Muscle % : Kupima kwa chibili |  | Enter second measurement for skeletal muscle percentage. |
| *RMS* | Resting metabolism (kcal) : Second measure |  |  | Resting metabolism (kcal) : Mulingo wachiwiri |  | Resting metabolism (kcal) : Kupima kwa chibili |  | Enter second measurement for resting metabolism. |
| *VFLS* | Visceral fat level : Second measure |  |  | Visceral fat level : Mulingo wachiwiri |  | Visceral fat level : Kupima kwa chibili |  | Enter second measurement for visceral fat percentage. |
| *HANDEDNESS* | Are you Right handed or Left handed? | 1  2 | Right-handed  Left-handed | Kodi mumagwilitsa ntchito kwambiri dzanja la manja kapena lamanzere? | Right-handed  Left-handed | Kasi ndimwe wakalizere panji muli wamalyelo? | Right-handed  Left-handed | This question asks which is the dominant hand for the participant. the interviewer should measure MUAC in the non-dominant hand as described in the “GM BLOOD PRESSURE AND ANTHROPOMETRY STANDARD OPERATING PROCEDURE” |
| *MIDACIF* | G. Mid-upper arm circumference -- Accuracy to 1 mm [0.1cm]: First measure |  |  | G. Mulingo wozungulira pakati pa mkono -- Accuracy to 1 mm [0.1cm]: Mulingo woyamba |  | G. Mid-arm circumference -- Kututuba pakati pa kaboko 1 mm [0.1cm]: Kupima kwa kwamba |  | Enter first MUAC. |
| *MIDACIS* | G. Mid-upper arm circumference -- Accuracy to 1 mm [0.1cm]: Second measure |  |  | G. Mulingo wozungulira pakati pa mkono -- Accuracy to 1 mm [0.1cm]: Mulingo wachiwiri |  | G. Mid-arm circumference -- Kututuba pakati pa kaboko 1 mm [0.1cm]: Kupima kwa chibili |  | Enter second MUAC. The tablet will prompt you to retake MUAC if the difference between first and second muac is >5%. |
| *S_16.8_J* | Diabetes Referal |  |  | Diabetes Referal |  | Diabetes Referal |  | This is done at dissemination of RBS/FBS results, after measurements. |
| *REDIANOTE3* | On medication for Diabetes |  |  | On medication for Diabetes |  | On medication for Diabetes |  |  |
| *REFDIB* | Referral slip given for diabetes assessment? | 1  0 | Yes  No | Mwapereka chikalata chokaonetsa kuchipatara cha matenda a shuga (referral slip given for diabetes)? | Eya  Ayi | Kasi mwawapa chikalata chakukalongola ku chipatala na shuga | Enya  Yayi |  |
| *SUICIDESAFETY_NOTE* | [NOTE TO FIELDWORKER] The next section will be the suicide safety action section |  |  | [NOTE TO FIELDWORKER] The next section will be the suicide safety action section |  | [NOTE TO FIELDWORKER] The next section will be the suicide safety action section |  | This is done for participants who have admitted to having suicidal thoughts during “MENTAL HEALTH” section. |
| *GSUICIDERISK* | SUICIDE RISK |  |  | SUICIDE RISK |  | SUICIDE RISK |  | The tablet will alert you the severity of the risk of suicide based on the participants’ responses in the “MENTAL HEALTH” section |
| *SRSSCOREHIGH* | HIGH RISK ON SRS: FOR URGENT ASSESSMENT BY MENTAL HEALTH NURSE/CLINICIAN  The SRS result is high: this means that the participant has said that he/she has the thought of suicide or of harming him/herself now or in the near future. Therefore he/she needs immediate further assessment and support to remain safe. You will help the participant contact a SUPPORT PERSON and refer the participant to the mental health nurse to be seen urgently at CRH/AREA 25HC, or to have an immediate telephone consultation.   IF AT ANY STAGE YOU ARE NOT SURE WHAT TO DO CALL THE NURSE, CLINICIAN OR FIELD SUPERVISOR FOR ADVICE.  ANSWERS TO SUICIDE RISK SCREEN:  Can I check, in the last 2 weeks have you had these thoughts many times or just once or twice? ${suiciderisk1slctd}  In the last 2 weeks, have you made any plans or considered a method that you might use to end your life or harm yourself? ${suiciderisk2slctd}  Have you ever attempted to harm yourself or end your life? ${suiciderisk3slctd}  Do you think there is any risk that you might harm yourself or end your life now or in the near future? ${suiciderisk4slctd} |  |  | HIGH RISK ON SRS: FOR URGENT ASSESSMENT BY MENTAL HEALTH NURSE/CLINICIAN  The SRS result is high: this means that the participant has said that he/she has the thought of suicide or of harming him/herself now or in the near future. Therefore he/she needs immediate further assessment and support to remain safe. You will help the participant contact a SUPPORT PERSON and refer the participant to the mental health nurse to be seen urgently at CRH/AREA 25HC, or to have an immediate telephone consultation.   IF AT ANY STAGE YOU ARE NOT SURE WHAT TO DO CALL THE NURSE, CLINICIAN OR FIELD SUPERVISOR FOR ADVICE.  ANSWERS TO SUICIDE RISK SCREEN:  Can I check, in the last 2 weeks have you had these thoughts many times or just once or twice? ${suiciderisk1slctd}  In the last 2 weeks, have you made any plans or considered a method that you might use to end your life or harm yourself? ${suiciderisk2slctd}  Have you ever attempted to harm yourself or end your life? ${suiciderisk3slctd}  Do you think there is any risk that you might harm yourself or end your life now or in the near future? ${suiciderisk4slctd} |  | HIGH RISK ON SRS: FOR URGENT ASSESSMENT BY MENTAL HEALTH NURSE/CLINICIAN  The SRS result is high: this means that the participant has said that he/she has the thought of suicide or of harming him/herself now or in the near future. Therefore he/she needs immediate further assessment and support to remain safe. You will help the participant contact a SUPPORT PERSON and refer the participant to the mental health nurse to be seen urgently at CRH/AREA 25HC, or to have an immediate telephone consultation.   IF AT ANY STAGE YOU ARE NOT SURE WHAT TO DO CALL THE NURSE, CLINICIAN OR FIELD SUPERVISOR FOR ADVICE.  ANSWERS TO SUICIDE RISK SCREEN:  Can I check, in the last 2 weeks have you had these thoughts many times or just once or twice? ${suiciderisk1slctd}  In the last 2 weeks, have you made any plans or considered a method that you might use to end your life or harm yourself? ${suiciderisk2slctd}  Have you ever attempted to harm yourself or end your life? ${suiciderisk3slctd}  Do you think there is any risk that you might harm yourself or end your life now or in the near future? ${suiciderisk4slctd} |  | This section is for a person at high risk of suicide (i.e. has the thought of suicide or of harming him/herself now or in the near future) should be referred immediately to mental health team for further assessment as described in the “?”  The tablet will require you to check the replies given to the different questions asked when assessing suicide risk. |
| *SRSHIGHREAD* | Read the following to participant:  You have said that you have the thought of ending your life or hurting yourself now or in the near future. I want to make sure you are safe and to help you get further assessment from a trained nurse today. Whilst I am arranging this, is there someone who could come and support you now? Please can you give me the name and phone number for that person so we can call them if needed? |  |  | Read the following to participant:  Munanena kuti muli ndimaganizo ofuna kuchotsa moyo wanukapena kuzivulaza nokha pakali pano kapena mtsogolo. Ndikufuna nditsimikize kuti muli ndi chitetezo chokwanira komanso ndikuthandizeni kupeza thandizo lofunikira kuchoka kwanamwinolero. Kodi pamene ndikukodzai ndongosololi, alipo aliyense yemwe angabwere kukuthandizani panopa? Chonde ndiuzeni dzina lake ndi nambala yake ya foni kuti tiyimbe ngati pangafunike kutero? |  | Read the following to participant:  Mwanguyowoya kuti muli na maghanoghano yakudumula moyo winu panji yakujipweteteka mwabene sono panji munthazi. Nkhukhumba nipanikizge kuti mwavikililika na kumovwilani kuti msange wovwiri wakukhumbikira wa vyaumoyo kufuma kwa nesi mwahuno. Apo nkhumunozgerani dongosolo ili kasi walipo munyake wakuti wangamuvwirani panyengo yasono? Chonde ndiphalirani zina lakhe na nambara yakhe ya foni usange nkhwakukhumbikwira? |  | Read exactly as written to participant. |
| *SPERSONIDNTFD* | Support person Identified | 1  0 | Yes  No | Support person Identified | Eya  Ayi | Support person Identified | Enya  Yayi | Record as appropriate. |
| *SUPORTNOTE* | RECORD SUPPORT PERSON NAME AND NUMBER |  |  | RECORD SUPPORT PERSON NAME AND NUMBER |  | RECORD SUPPORT PERSON NAME AND NUMBER |  | Record name and phone number of support person if identified.  . |
| *SUPORTPERSONNAMEH* | A. Name |  |  | A. Name |  | A. Name |  |  |
| *SUPORTPERSONPHONEH* | B. Phone |  |  | B. Phone |  | B. Phone |  |  |
| *FETCHSUPPPRSON* | If SUPPORT PERSON is present or living nearby, fetch them or arrange someone to fetch them. If the SUPPORT PERSON is not present or local, with permission of the participant, call this person on the phone. |  |  | If SUPPORT PERSON is present or living nearby, fetch them or arrange someone to fetch them. If the SUPPORT PERSON is not present or local, with permission of the participant, call this person on the phone. |  | If SUPPORT PERSON is present or living nearby, fetch them or arrange someone to fetch them. If the SUPPORT PERSON is not present or local, with permission of the participant, call this person on the phone. |  |  |
| *READFORSUPORTPRSON* | Read the following to the SUPPORT PERSON (in person or by phone):  I am [field worker name] working with MEIRU and today I am interviewing ${fullname} who is taking part in GENERATION MALAWI STUDY. ${fullname} has confidence in you, that is why I am talking to you, so please remember that this information we are discussing is confidential between you, ${fullname}, and any health professionals. ${fullname} has shared with me that they feel like they might harm themselves. This is a mental health issue that they need to speak to a health professional about. It is important for their safety that they speak to a mental health professional today to be evaluated. Would you be able to help them go to CRH/AREA 25HC today? | 1  0  8  6 | Yes  No  Don't Know  Refused to answer | Read the following to the SUPPORT PERSON (in person or by phone):  Ine ndine[ field worker name] ndikugwira ntchito ndi a MEIRU lero ndikumufunsa mafunso ${fullname} yemwe akutenga nawo mbali mukafukufuku wa GENERATION MALAWI STUDY. ${fullname} alindichikhulupilo mwa inu, ndichifukwa ndikulakhulana nanu, chonde dziwani kuti tikukambilanazi ndi za chinsisi pakati pa inu, ${fullname}, ndi azaumoyo. ${fullname}, afotokoza kuti atha kuzivulaza yenkha. Izi ndi zokhuza matenda a malingaliro angwiro zoyenera kuwafotokozera a zaumoyo. Ndizofunika pa chitetezo chawo kuti ayankhule ndi akatswiri owona za matenda a malingaliro a ngwiro kuti awapime. Kodi mungathe kuwathandiza kuti apite ku chipatalacha ku AREA 25 lero? | Eya  Ayi  Sindikudziwa  Refused to answer | Read the following to the SUPPORT PERSON (in person or by phone):  Ndine[ field worker name] nkhugwira ntchito ku MEIRU mwahuno nafumbanga mafumbo ${fullname} uyo wakutola lwande mukafukufuku wa GENERATION MALAWI STUDY. ${fullname} wanachigomezgo mwa imwe lekani ntchifukwa nkhuyowoyeskana na imwe, chonde manyani kuti wanduzi uwu tikudumbiskana ngwa chisisi pakati pa imwe,${fullname} na waliyose wa vyaumoyo. Wakutolapo lwande wawoneska kutiwali na maghanoghano yakuti wangajipweteka yenkha. Iyi ninkhani yakukhwaskana na na matende yamaghanoghano yakuti wayowoyeskane na wavyaumoyo. Ntchakuzirwa ku chivikiliro chabo kuti bandumbilane na badakotala wa maghanoghano gha wemi. Kasi mungabavwira kuti balute ku chipatala cha Chilumba mwahuno? | Enya  Yayi  Nkhumanya yayi  Refused to answer |  |
| *NOSUPORTPRSON* | If NO SUPPORT PERSON is available or identified. - Stay with the person whilst you contact the mental health nurse  Read the following to the participant: I now need to call the mental health nurse to arrange for them to talk with you.    If the person objects, stress that it is your responsibility to call the mental health nurse in order to make a plan to ensure that the person is safe. |  |  | If NO SUPPORT PERSON is available or identified. - Stay with the person whilst you contact the mental health nurse  Read the following to the participant: Pano ndiimbira a namwino owona za matende a malingaliro a ngwiro kuti akonze nthawi yoti muyankhulane nawo.   [If the person objects] Ndiudindo wanga kuwayimbira foni anamwino/dotolo owona za matenda a malingaliro agwiro kuti akonze dongosolo loti inu mukhale otetezeka. |  | If NO SUPPORT PERSON is available or identified. - Stay with the person whilst you contact the mental health nurse  Read the following to the participant: Sono ndibayimbilenge ba nesi bakuwona vya maghanaghaniro ya wemi kuti banozgere nyengo yakuti bayowoyeskane namwe   [If the person objects,]Ndiudindo wane kuwayimbira foni a wadokotala wa vya maghanoghano yawemi kuti banozge ndondomeko yakuwoneskeska kuti imwe muli bakuvikililika. |  | If no support person, contact MH team. STAY with the person until contacted the team. Read the note to the participant exactly as written. |
| *GOTOCLINIC* | Read the following to the participant:  I can arrange transport to take you to CRH/Area 25HC to meet with the nurse.  Are you willing to go to the clinic? | 1  0  8  6 | Yes  No  Don't Know  Refused to answer | Read the following to the participant:  Nditha kukonza mayendedwe okutengelani ku chipatala cha ku Area 25 kuti mukakumane ndi namwino/dotolo. Kodi mukufuna kupita ku chipatala. | Eya  Ayi  Sindikudziwa  Refused to answer | Read the following to the participant:  Ndinganozgera mendero yakuti imwe mulute ku chipatala cha Chilumba kuti kukakumane na ba nesi/ dokotala. Kasi muli bakunweka kuluta ku chipatala? | Enya  Yayi  Nkhumanya yayi  Refused to answer | Read note exactly as written to arrange transport for participant. |
| *YESCLINIC* | If participant agrees to go to the clinic:  Call the mental health nurse and advise that participant is high risk on SRS. If nurse not available call MEIRU clinician.  Give the nurse/clinician the name and phone number of PARTICIPANT and of SUPPORT PERSON  Participant name: ${fullname} Participant's phone number 1: ${phonea} Participant's phone number 2: ${phoneb}  Support person's name: ${suportpersonnameh} Support person's phone number: ${suportpersonphoneh}  Arrange transport. Complete the Counsellor Referral Section on the referral form and give to the participant. Include the PHQ9, GAD7, PC-PTSD, and AUDIT scores as shown here: |  |  | If participant agrees to go to the clinic:  Call the mental health nurse and advise that participant is high risk on SRS. If nurse not available call MEIRU clinician.  Give the nurse/clinician the name and phone number of PARTICIPANT and of SUPPORT PERSON  Participant name: ${fullname} Participant's phone number 1: ${phonea} Participant's phone number 2: ${phoneb}  Support person's name: ${suportpersonnameh} Support person's phone number: ${suportpersonphoneh}  Arrange transport. Complete the Counsellor Referral Section on the referral form and give to the participant. Include the PHQ9, GAD7, PC-PTSD, and AUDIT scores as shown here: |  | If participant agrees to go to the clinic:  Call the mental health nurse and advise that participant is high risk on SRS. If nurse not available call MEIRU clinician.  Give the nurse/clinician the name and phone number of PARTICIPANT and of SUPPORT PERSON  Participant name: ${fullname} Participant's phone number 1: ${phonea} Participant's phone number 2: ${phoneb}  Support person's name: ${suportpersonnameh} Support person's phone number: ${suportpersonphoneh}  Arrange transport. Complete the Counsellor Referral Section on the referral form and give to the participant. Include the PHQ9, GAD7, PC-PTSD, and AUDIT scores as shown here: |  | Inform MH nurse or MEIRU clinician that you are making a referral for a patient at high risk of suicide. Arrange transport and refer participant with the referral form. Complete counsellor field form. Include the PHQ9, GAD7, PC-PTSD, and AUDIT scores as shown on the tablet display. |
| *NOTOCLINIC* | If participant does not agree to go to the clinic:  Call the mental health nurse and arrange for him to call back straight away. If the participant has own phone, then the mental health nurse should call on that number, or that of the support person. If neither has a phone then lend the participant your phone. Wait with participant until further instructions from mental health nurse. Complete the section on the referral form as advised by the nurse and give to the participant and give to the participant. Show them that the clinic phone number is on the form |  |  | If participant does not agree to go to the clinic:  Call the mental health nurse and arrange for him to call back straight away. If the participant has own phone, then the mental health nurse should call on that number, or that of the support person. If neither has a phone then lend the participant your phone. Wait with participant until further instructions from mental health nurse. Complete the section on the referral form as advised by the nurse and give to the participant and give to the participant. Show them that the clinic phone number is on the form |  | If participant does not agree to go to the clinic:  Call the mental health nurse and arrange for him to call back straight away. If the participant has own phone, then the mental health nurse should call on that number, or that of the support person. If neither has a phone then lend the participant your phone. Wait with participant until further instructions from mental health nurse. Complete the section on the referral form as advised by the nurse and give to the participant and give to the participant. Show them that the clinic phone number is on the form |  | If participant does not agree to go to for immediate assessment, inform mental health team, who will make a phone call to the speak to the participant immediately. Wait with participant until further instructions from MH nurse. Complete counsellor field form. Include the PHQ9, GAD7, PC-PTSD, and AUDIT scores as shown on the tablet display.and MH nurse phone number. |
| *NOTEHIGH* | Show the participant that the clinic phone number is on the form if needed. When transport arrives, alert the MH nurse that participant and support person are on their way. |  |  | Show the participant that the clinic phone number is on the form if needed. When transport arrives, alert the MH nurse that participant and support person are on their way. |  | Show the participant that the clinic phone number is on the form if needed. When transport arrives, alert the MH nurse that participant and support person are on their way. |  |  |
| *MODERATERISKSUICIDE* | MODERATE RISK ON SRS: FOR SOON ASSESSMENT BY MENTAL HEALTH NURSE/CLINICIAN  The SRS result is MODERATE: this means that the participant has said that he/she has one or more of: frequent thoughts of hurting him/herself, has thought of a method, or has done it before. Therefore he/she needs further assessment and support within the next few days. You will help the participant contact a SUPPORT PERSON and refer them to a counsellor/nurse to be seen at CRH/AREA 25HC, or to have a telephone consultation.   IF AT ANY STAGE YOU ARE NOT SURE WHAT TO DO CALL THE NURSE, CLINICIAN OR FIELD SUPERVISOR FOR ADVICE.  ANSWERS TO SUICIDE RISK SCREEN:  Can I check, in the last 2 weeks have you had these thoughts many times or just once or twice? ${suiciderisk1slctd}  In the last 2 weeks, have you made any plans or considered a method that you might use to end your life or harm yourself? ${suiciderisk2slctd}  Have you ever attempted to harm yourself or end your life? ${suiciderisk3slctd}  Do you think there is any risk that you might harm yourself or end your life now or in the near future? ${suiciderisk4slctd} |  |  | MODERATE RISK ON SRS: FOR SOON ASSESSMENT BY MENTAL HEALTH NURSE/CLINICIAN  The SRS result is MODERATE: this means that the participant has said that he/she has one or more of: frequent thoughts of hurting him/herself, has thought of a method, or has done it before. Therefore he/she needs further assessment and support within the next few days. You will help the participant contact a SUPPORT PERSON and refer them to a counsellor/nurse to be seen at CRH/AREA 25HC, or to have a telephone consultation.   IF AT ANY STAGE YOU ARE NOT SURE WHAT TO DO CALL THE NURSE, CLINICIAN OR FIELD SUPERVISOR FOR ADVICE.  ANSWERS TO SUICIDE RISK SCREEN:  Can I check, in the last 2 weeks have you had these thoughts many times or just once or twice? ${suiciderisk1slctd}  In the last 2 weeks, have you made any plans or considered a method that you might use to end your life or harm yourself? ${suiciderisk2slctd}  Have you ever attempted to harm yourself or end your life? ${suiciderisk3slctd}  Do you think there is any risk that you might harm yourself or end your life now or in the near future? ${suiciderisk4slctd} |  | MODERATE RISK ON SRS: FOR SOON ASSESSMENT BY MENTAL HEALTH NURSE/CLINICIAN  The SRS result is MODERATE: this means that the participant has said that he/she has one or more of: frequent thoughts of hurting him/herself, has thought of a method, or has done it before. Therefore he/she needs further assessment and support within the next few days. You will help the participant contact a SUPPORT PERSON and refer them to a counsellor/nurse to be seen at CRH/AREA 25HC, or to have a telephone consultation.   IF AT ANY STAGE YOU ARE NOT SURE WHAT TO DO CALL THE NURSE, CLINICIAN OR FIELD SUPERVISOR FOR ADVICE.  ANSWERS TO SUICIDE RISK SCREEN:  Can I check, in the last 2 weeks have you had these thoughts many times or just once or twice? ${suiciderisk1slctd}  In the last 2 weeks, have you made any plans or considered a method that you might use to end your life or harm yourself? ${suiciderisk2slctd}  Have you ever attempted to harm yourself or end your life? ${suiciderisk3slctd}  Do you think there is any risk that you might harm yourself or end your life now or in the near future? ${suiciderisk4slctd} |  | This is for participants with a moderate risk of suicide (i.e participant has said that he/she has one or more of: frequent thoughts of hurting him/herself, has thought of a method, or has done it before). Participants with moderate risk if suicide need further assessment within the next few days at the clinics. Involve the mental health nurse or clinician if not sure what to do.  The tablet will require you to check the replies given to the different questions asked when assessing suicide risk. |
| *MODRTESUPPRSN* | Read the following to the participant:  You have said that you have the thought of hurting yourself frequently, had thought of how you would do it, or have done it before. I want to make sure you are safe and to help you get further assessment from a counsellor/nurse. As well as help from a professional, is there someone who you can talk to about your feelings and who can support you? Please can you give me the name and phone number for that person so we can call them if needed? |  |  | Read the following to the participant:   Mwanena kuti mukumakhala ndimaganizo ofuna kuzivulaza nokha mwapafupipafupi, muli ndi malingaliro oti muntha kuzivulaza bwanji kapena mudayetselapo m'mbuyomu. Ndikufuna nditsimikize kuti muli ndi chitetezo chokwanira komanso ndikuthandizeni kupeza thandizo lofunikira kuchoka kwa alangizi/ namwino lero. Komanso kupeza thandizo kuchoka kwa akatswiri, kodi pamene alipo aliyense yemwe mungamuudze zamomwe mukumvera mtima komanso angabwere kukuthandizani? Chonde ndiuzeni dzina lake ndi nambala yake ya foni kuti tiyimbe ngati pangafunike kutero? |  | Read the following to the participant:  Mwanguyowoya kuti mukuba na maghanoghano yakudumula moyo winu panji yakujipweteteka mwabene mwapafupipafupi, muli kughanaghanira umo mungajipwetekera, panji mulikuyezgapo kale kujipweteka mekha. Nkhukhumba nipanikizge kuti mwavikililika na kumovwilani kuti msange wovwiri wakukhumbikira wa vyaumoyo kufuma kwa ba phungu/nesi mwahuno. Kweniso wovwiri kufuma ku nkhwantha, kasi walipo munyake wakuti munga mumanyska zaumo mukujipulikira mtima ndipo wangamovwirani ? Chonde ndiphalirani zina lakhe na nambara yakhe ya foni usange nkhwakukhumbikwira? |  | Read note exactly as written to participant. |
| *MDRTSUPPRSN* | Support person Identified | 1  0 | Yes  No | Support person Identified | Eya  Ayi | Support person Identified | Enya  Yayi | Record as responded. |
| *SUPORTNOTE* | RECORD SUPPORT PERSON NAME AND NUMBER |  |  | RECORD SUPPORT PERSON NAME AND NUMBER |  | RECORD SUPPORT PERSON NAME AND NUMBER |  | Record support person name and phone number. |
| *SUPORTPERSONNAMEM* | A. Name |  |  | A. Name |  | A. Name |  |  |
| *SUPORTPERSONPHONEM* | B. Phone |  |  | B. Phone |  | B. Phone |  |  |
| *SPEAKSUPPRSN* | Read the following to the participant:   Do you want me to speak to your SUPPORT PERSON right now? | 1  0  8  6 | Yes  No  Don't Know  Refused to answer | Read the following to the participant:   Kodi mukufuna ndiyankhule ndi [ SUPPORT PERSON NAME] pakali pano? | Eya  Ayi  Sindikudziwa  Refused to answer | Read the following to the participant:   Kasi mukukhumba kuti niyowoye nayo[ SUPPORT PERSON NAME] sono nthena? | Enya  Yayi  Nkhumanya yayi  Refused to answer | Read note exactly as written. Record as responded. |
| *SPEAKSUPPRSNYES* | If the participant agrees, speak to or call the SUPPORT PERSON:  Read the following to the SUPPORT PERSON (in person or by phone): ${fullname} has confidence in you, that is why I am talking to you, so please remember that this information we are discussing is confidential between you, ${fullname}, and any health professionals. ${fullname} has shared with me that they feel like they might harm themselves. This is a mental health issue that they need to speak to a health professional about. It is important for their safety that they speak to a mental health professional to be evaluated. Would you be able to provide some support to them? | 1  0  8  6 | Yes  No  Don't Know  Refused to answer | If the participant agrees, speak to or call the SUPPORT PERSON:  Read the following to the SUPPORT PERSON (in person or by phone): Ine ndine[ field worker name] ndikugwira ntchito ndi a MEIRU lero ndikumufunsa mafunso ${fullname} yemwe akutenga nawo mbali mukafukufuku wa Generation Malawi Study. ${fullname} alindichikhulupilo mwa inu, ndichifukwa ndikulakhulana nanu, chonde dziwani kuti tikukambilanazi ndi za chinsisi pakati pa inu, ${fullname}, ndi azaumoyo. ${fullname}, afotokoza kuti atha kuzivulaza yenkha. Izi ndi zokhuza matenda a malingaliro angwiro zoyenera kuwafotokozera a zaumoyo. Ndizofunika pa chitetezo chawo kuti ayankhule ndi akatswiri owona za matenda a malingaliro a ngwiro kuti awapime. Kodi mungathe kuwathandiza? | Eya  Ayi  Sindikudziwa  Refused to answer | If the participant agrees, speak to or call the SUPPORT PERSON:  Read the following to the SUPPORT PERSON (in person or by phone): Ndine[ field worker name] nkhugwira ntchito ku MEIRU mwahuno nafumbanga mafumbo ${fullname} uyo wakutola lwande mukafukufuku wa Generation Malawi Study. ${fullname} wanachigomezgo mwa imwe lekani ntchifukwa nkhuyowoyeskana na imwe, chonde manyani kuti wanduzi uwu tikudumbiskana ngwa chisisi pakati pa imwe, ${fullname} na waliyose wa vyaumoyo. Wakutolapo lwande wawoneska kutiwali na maghanoghano yakuti wangajipweteka yenkha. Iyi ninkhani yakukhwaskana na na matende yamaghanoghano yakuti wayowoyeskane na wavyaumoyo. Ntchakuzirwa ku chivikiliro chabo kuti bandumbilane na badakotala wa maghanoghano gha wemi. Kasi mungabavwira? | Enya  Yayi  Nkhumanya yayi  Refused to answer | If agrees to question above, read note as written Record as responded. |
| *MODERATECALLNURSE* | Read the following to the participant:  I now need to call the counsellor/nurse to arrange an appointment for you. It is best if this is a face-to-face appointment at CRH/AREA 25 HC. If you are unable to travel there, then we can arrange a telephone call. Are you able to go to CRH/Area 25HC to meet with the counsellor or would you prefer a phone call? | 1  2  3  6 | Meet with counsellor  Prefer phone call  Participant does not agree to any referral  Refused to answer | Read the following to the participant:  Pano ndiwaimbira phungu/namwino/dotolo kuti akonze nthawi yokumana nanu. Zingakhale zofunikira kwambiri kukumana maso ndi maso ku chipatala cha kuAREA 25 . Ngati mungakanike kupita , atha kukuyimbirani foni/lamya. Mutha kukwanitsa kupita ku chipatala cha ku AREA 25 kuti mukakumane ndi aphungu/anamwino/adotolo.? Kapena mungakonde angokuyimbirani foni? | Meet with counsellor  Prefer phone call  Participant does not agree to any referral  Refused to answer | Read the following to the participant:  Sono ndibayimbilenge ba lanizi/ nesi/dokotala kuti banozgere nyengo yakuti bayowoyeskane namwe. Chingaba makola chomene para mungandumbiskana nawo maso na maso ku chipata cha ku Chilumba. Para mungakwaniska cha mendero, tinganozgera kuti muyowoye nabo kwizira pa foni. Kasi mungakwaniska kuluta ku chipatala cha chilumba kuti mukakumane na balangizi? panyekhe mungatemwa kuti kuyowoyeskana nawo pa foni? | Meet with counsellor  Prefer phone call  Participant does not agree to any referral  Refused to answer | Read note exactly as written and record as responded. |
| *MODRTENOTOCLINIC* | If participant does not agree to any referral to the clinic:  Call the mental health nurse, discuss the situation and get instructions from the nurse. He may want to talk to the participant. If so arrange for him to call back straight away. If the participant has own phone, then the mental health nurse should call on that number, or that of the support person. If neither has a phone then lend the participant your phone. Wait with participant until further instructions from the nurse. If the participant now agrees to referral, complete the referral form. |  |  | If participant does not agree to any referral to the clinic:  Call the mental health nurse, discuss the situation and get instructions from the nurse. He may want to talk to the participant. If so arrange for him to call back straight away. If the participant has own phone, then the mental health nurse should call on that number, or that of the support person. If neither has a phone then lend the participant your phone. Wait with participant until further instructions from the nurse. If the participant now agrees to referral, complete the referral form. |  | If participant does not agree to any referral to the clinic:  Call the mental health nurse, discuss the situation and get instructions from the nurse. He may want to talk to the participant. If so arrange for him to call back straight away. If the participant has own phone, then the mental health nurse should call on that number, or that of the support person. If neither has a phone then lend the participant your phone. Wait with participant until further instructions from the nurse. If the participant now agrees to referral, complete the referral form. |  | If participant denies any referral, call mental health nurse for advice and instruction on further actions. |
| *COUNSELORDETAILS* | Contact the counsellor.  Give the counsellor the participant details and arrange an in person or telephone appointment within next 2-3 days.  Participant name: ${fullname} Participant's phone number 1: ${phonea} Participant's phone number 2: ${phoneb}  Complete the Counsellor Referral Section on the referral form and give to the participant. Include the PHQ9, GAD7, PC-PTSD, and AUDIT scores as shown here: |  |  | Contact the counsellor.  Give the counsellor the participant details and arrange an in person or telephone appointment within next 2-3 days.  Participant name: ${fullname} Participant's phone number 1: ${phonea} Participant's phone number 2: ${phoneb}  Complete the Counsellor Referral Section on the referral form and give to the participant. Include the PHQ9, GAD7, PC-PTSD, and AUDIT scores as shown here: |  | Contact the counsellor.  Give the counsellor the participant details and arrange an in person or telephone appointment within next 2-3 days.  Participant name: ${fullname} Participant's phone number 1: ${phonea} Participant's phone number 2: ${phoneb}  Complete the Counsellor Referral Section on the referral form and give to the participant. Include the PHQ9, GAD7, PC-PTSD, and AUDIT scores as shown here: |  | If participant opts to meet counsellor, contact counsellor and arrange a meeting with the participant. Complete referral form. Include the PHQ9, GAD7, PC-PTSD, and AUDIT. |
| *NOTEPHQ9DAD7* | Show the participant that the clinic phone number is on the form. Read the following to the participant: If you feel worse, please call this number. If you are unable to phone this number you should go to CRH/AREA 25 HC. |  |  | Show the participant that the clinic phone number is on the form. Read the following to the participant: Ngati mungapitilire kusamva bwino chonde imbani foni ku nambala iyi. Ngati simungakwanitse kuimba foni/lamya pitani ku chipatala cha ku AREA 25. |  | Show the participant that the clinic phone number is on the form. Read the following to the participant: Para mungapitilira kujipulika makola chala chone,chonde yimbani kunambara iyi. Para mwakwaniska chala kwimba ku nambala ya foni iyi ipo mulute ku chipatala cha Chilumba. |  | Show participant clinic phone number to be used when need. |
| *LOWRISKSUICIDE* | LOW RISK ON SRS: FOR ADVICE, SIGNPOSTING AND ROUTINE APPT IF WANTED  The SRS result is LOW: this means that the participant has said that he/she only has occasional thoughts of ending life or hurting him/herself, and no intent to do so. They may benefit from getting support from friends/family and should be made aware of the option to see a counsellor if they wish.  IF AT ANY STAGE YOU ARE NOT SURE WHAT TO DO CALL THE NURSE, CLINICIAN OR FIELD SUPERVISOR FOR ADVICE.  ANSWERS TO SUICIDE RISK SCREEN:  Can I check, in the last 2 weeks have you had these thoughts many times or just once or twice? ${suiciderisk1slctd}  In the last 2 weeks, have you made any plans or considered a method that you might use to end your life or harm yourself? ${suiciderisk2slctd}  Have you ever attempted to harm yourself or end your life? ${suiciderisk3slctd}  Do you think there is any risk that you might harm yourself or end your life now or in the near future? ${suiciderisk4slctd} |  |  | LOW RISK ON SRS: FOR ADVICE, SIGNPOSTING AND ROUTINE APPT IF WANTED  The SRS result is LOW: this means that the participant has said that he/she only has occasional thoughts of ending life or hurting him/herself, and no intent to do so. They may benefit from getting support from friends/family and should be made aware of the option to see a counsellor if they wish.  IF AT ANY STAGE YOU ARE NOT SURE WHAT TO DO CALL THE NURSE, CLINICIAN OR FIELD SUPERVISOR FOR ADVICE.  ANSWERS TO SUICIDE RISK SCREEN:  Can I check, in the last 2 weeks have you had these thoughts many times or just once or twice? ${suiciderisk1slctd}  In the last 2 weeks, have you made any plans or considered a method that you might use to end your life or harm yourself? ${suiciderisk2slctd}  Have you ever attempted to harm yourself or end your life? ${suiciderisk3slctd}  Do you think there is any risk that you might harm yourself or end your life now or in the near future? ${suiciderisk4slctd} |  | LOW RISK ON SRS: FOR ADVICE, SIGNPOSTING AND ROUTINE APPT IF WANTED  The SRS result is LOW: this means that the participant has said that he/she only has occasional thoughts of ending life or hurting him/herself, and no intent to do so. They may benefit from getting support from friends/family and should be made aware of the option to see a counsellor if they wish.  IF AT ANY STAGE YOU ARE NOT SURE WHAT TO DO CALL THE NURSE, CLINICIAN OR FIELD SUPERVISOR FOR ADVICE.  ANSWERS TO SUICIDE RISK SCREEN:  Can I check, in the last 2 weeks have you had these thoughts many times or just once or twice? ${suiciderisk1slctd}  In the last 2 weeks, have you made any plans or considered a method that you might use to end your life or harm yourself? ${suiciderisk2slctd}  Have you ever attempted to harm yourself or end your life? ${suiciderisk3slctd}  Do you think there is any risk that you might harm yourself or end your life now or in the near future? ${suiciderisk4slctd} |  | This section is for participants with low risk (i.e. participant has said that he/she only has occasional thoughts of ending life or hurting him/herself, and no intent to do so). These will be given advice as highlighted in the mental health referral form. They are also given an option to meet a counsellor if they wish to do so. |
| *APPT* | Read the following to the participant:  Is there someone you can talk to about how you are feeling? I suggest you speak to that person for support. If you feel you would like to speak to a counsellor about your problems, you can attend the counselling clinic at CRH/AREA25 HC or call the counsellor by phone. The clinic at CRH takes place on every Thursday morning. | 1  0  8  6 | Yes  No  Don't Know  Refused to answer | Read the following to the participant:  Kodi alipo yemwe mungayakhule naye za momwe mukuzimvera? Ngati ndi choncho Yankhulani nawo kuti akuthandizeni. Ngati mungafune kuyakhula ndi aphungu za mavuto anu, mutha kupita kukalandira uphungu ku chipatala cha AREA25 kapena kuwaimbira lamya aphungu. Timatsegula kiliniki pachipatala cha Chilumba lachinayi lirilonse m’mawa. | Eya  Ayi  Sindikudziwa  Refused to answer | Read the following to the participant:  Kasi pali munthu yunji uyo mungayowoyeskana naye za umo mukujipulikira? Para enya Nkhughanaghana kuti muyoyeskane nayo kuti wamovwilani. Para mukujipulika kuti muyowoye na balangizi vya suzgo ili, mungaluta ku kakapokera ulangizi ku chipatala cha Chilumba panji kubayimbira foni balangizi. Tikujulanga kiliniki pachipatala cha Chilumba pachinayi chilichose namulenji. | Enya  Yayi  Nkhumanya yayi  Refused to answer | Read the note as written to participant. Record as responded. |
| *LOWCALLCOUNSLR* | Complete the Counsellor Referral Section on the referral form and give to the participant. Include the PHQ9, GAD7, PC-PTSD, and AUDIT scores as shown here: |  |  | Complete the Counsellor Referral Section on the referral form and give to the participant. Include the PHQ9, GAD7, PC-PTSD, and AUDIT scores as shown here: |  | Complete the Counsellor Referral Section on the referral form and give to the participant. Include the PHQ9, GAD7, PC-PTSD, and AUDIT scores as shown here: |  | Complete counsellor field form. Include the PHQ9, GAD7, PC-PTSD, and AUDIT scores as shown on the tablet display. |
| *NOTEPHQ9DAD7* | Show the participant that the clinic phone number is on the form. |  |  | Show the participant that the clinic phone number is on the form. |  | Show the participant that the clinic phone number is on the form. |  | Show participant the MH clinic phone number to use when required. |
| *NOAPPT* | Read the following to the participant: Even if you don’t want to see someone now, if you feel worse and any thoughts of harming yourself get stronger, then I particularly recommend that you visit the counsellor office at CRH/AREA 25HC or call the counsellor number. |  |  | Read the following to the participant: Ngakhale muthakhala kuti simukufuna kuonana ndi aliyese panopa, ngati zikupitilira kuti maganizowa akutchulukira , ndikukulangizani kuti mukawonane ndi aphungu ku ofesi yawo ku chipatala ku Area 25 kapena muwayimbire pa nambala iyi. |  | Read the following to the participant: Nanga uli mungakhumba yayi kukumana na waliyose kwasono, pala mukupitilira kujipulika makola yayi kweniso maghanoghano aya yakupitilira chomene, nkhumuchiskani kuti mukawonane na balangizi kuchipatala cha ku chilumaba panji mubayimbile balangizi pa nambala ya foni iyi. |  | Read note as written to participant. |
| *REFMENT* | Referral given for mental health assessment? | 1  0 | Yes  No | Referral given for mental health assessment? | Eya  Ayi | Referral given for mental health assessment? | Enya  Yayi | Record as responded. |
| *REFMENTPHOTO* | Instruction to fieldworker: Photograph the mental health referral form |  |  | Instruction to fieldworker: Photograph the mental health referral form |  | Instruction to fieldworker: Photograph the mental health referral form |  | Photograph mental health referral form. |
| *PHOTOTAKENMENT* | Was photo/s taken? | 1  0 | Yes  No | Was photo/s taken? | Eya  Ayi | Was photo/s taken? | Enya  Yayi | Record as responded. |
| ***REFERALSUMMARY*** | **PHYSICAL HEALTH REFERAL SUMMARY** |  |  | **PHYSICAL HEALTH REFERAL SUMMARY** |  | **PHYSICAL HEALTH REFERAL SUMMARY** |  | **This section will show you the physical measurement summary.** |
| *BPSUMMARYNOTE* | Blood pressure Systolic: ${sysmean} mmHg (average of reading 2 and 3) Blood pressure Diastolic: ${diamean} mmHg (average of reading 2 and 3) Body mass index (BMI): ${bmi} kg/m2 |  |  | Blood pressure Systolic: ${sysmean} mmHg (average of reading 2 and 3) Blood pressure Diastolic: ${diamean} mmHg (average of reading 2 and 3) Body mass index (BMI): ${bmi} kg/m2 |  | Blood pressure Systolic: ${sysmean} mmHg (average of reading 2 and 3) Blood pressure Diastolic: ${diamean} mmHg (average of reading 2 and 3) Body mass index (BMI): ${bmi} kg/m2 |  | Summary of BP and BMI. The tablet will also display whether participanst already on high BP or diabetes medication or not (based on previous replies). |
| *BPYESDIABYESSUMMARY* | Already on medication for Hypertension (high blood pressure): Yes Already on medication for Diabetes(High blood sugar: Yes |  |  | Already on medication for Hypertension (high blood pressure): Yes Already on medication for Diabetes(High blood sugar: Yes |  | Already on medication for Hypertension (high blood pressure): Yes Already on medication for Diabetes(High blood sugar: Yes |  |  |
| *BPNODIABNOSUMMARY* | Already on medication for Hypertension (high blood pressure): No Already on medication for Diabetes(High blood sugar: No |  |  | Already on medication for Hypertension (high blood pressure): No Already on medication for Diabetes(High blood sugar: No |  | Already on medication for Hypertension (high blood pressure): No Already on medication for Diabetes(High blood sugar: No |  |  |
| *BPYESDIABNOSUMMARY* | Already on medication for Hypertension (high blood pressure): Yes Already on medication for Diabetes(High blood sugar: No |  |  | Already on medication for Hypertension (high blood pressure): Yes Already on medication for Diabetes(High blood sugar: No |  | Already on medication for Hypertension (high blood pressure): Yes Already on medication for Diabetes(High blood sugar: No |  |  |
| *BPNODIABYESSUMMARY* | Already on medication for Hypertension (high blood pressure): No Already on medication for Diabetes(High blood sugar: Yes |  |  | Already on medication for Hypertension (high blood pressure): No Already on medication for Diabetes(High blood sugar: Yes |  | Already on medication for Hypertension (high blood pressure): No Already on medication for Diabetes(High blood sugar: Yes |  |  |
| *END_LTC* | Thank you for taking part in the survey! |  |  | Thank you for taking part in the survey! |  | Thank you for taking part in the survey! |  | Thank Participant for taking part in study. |
| *RCDR* | INTERVIEWER STAFF CODE |  |  | INTERVIEWER STAFF CODE |  | INTERVIEWER STAFF CODE |  | Enter interviewer code. |
| *COMDATE* | DATE FORM COMPLETED |  |  | DATE FORM COMPLETED |  | DATE FORM COMPLETED |  | Enter date |
| *OTHERNOTES* | ANY OTHER NOTES |  |  | ANY OTHER NOTES |  | ANY OTHER NOTES |  | Add any notes. |
| *END_LTC* |  |  |  |  |  |  |  | Save as finalised data. |
